# An Efficient Synthesis of 2-CF_3_-3-Benzylindoles

**DOI:** 10.3390/molecules26165084

**Published:** 2021-08-22

**Authors:** Vasiliy M. Muzalevskiy, Zoia A. Sizova, Valentine G. Nenajdenko

**Affiliations:** Department of Chemistry, Lomonosov Moscow State University, 119899 Moscow, Russia; muzvas@mail.ru (V.M.M.); syzova@mail.ru (Z.A.S.)

**Keywords:** CF_3_-group, enone, nitro, reduction, ammonium formate, indole, fluorine

## Abstract

The reaction of α-CF_3_-β-(2-nitroaryl) enamines with benzaldehydes afforded effectively α,β-diaryl-CF_3_-enones having nitro group. Subsequent reduction of nitro group by NH_4_HCO_2_-Pd/C system initiated intramolecular cyclization to give 2-CF_3_-3-benzylindoles. Target products can be prepared in up to quantitative yields. Broad synthetic scope of the reaction was shown. Probable mechanism of indole formation is proposed.

## 1. Introduction

Organofluorine chemistry is now hot topic area of modern organic chemistry. A lot of attention has been paid to elaboration of novel synthetic approaches towards fluorine-containing compounds as well as investigation of their chemical properties. Such concern of the chemists about these compounds is a result of their unique physicochemical and biological properties [1,2,3,4,5]. Fluorinated compounds are widely used as construction materials, components of liquid crystalline compositions, agrochemicals [6,7,8,9] and pharmaceuticals [10,11,12]. It was reported recently, that about 20% (more than 300 compounds) of currently used drugs [13,14,15,16,17,18,19,20] contain at least one fluorine atom [21]. Moreover, last years revealed the tendency of increasing of these values. Thus, share of fluoropharmaceuticals among new small-molecule drugs was 45% in 2018 [22], and 41% in 2019 [23]. On the other hand, about 59% of small-molecule drugs are the derivatives of nitrogen heterocyclic compounds [24]. As a result, novel approaches to fluorinated heterocycles are highly attractive [25,26,27,28,29,30,31].

**Figure 1:**
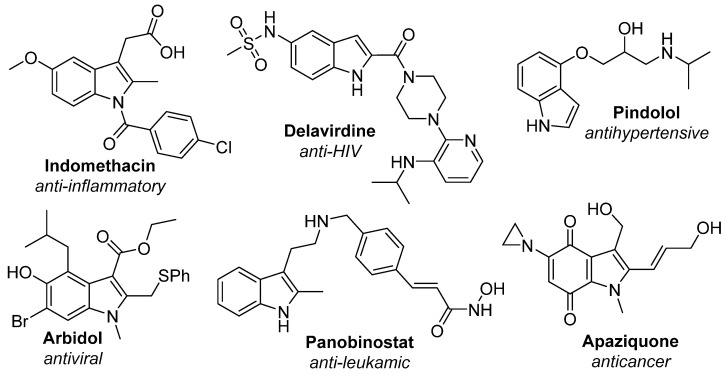
Indole based marketed drugs.

Indole [32,33,34,35,36,37,38] is a “privileged structure” in drug discovery [39] and can be frequently found in pharmaceuticals and natural products [24]. The derivatives of 2-arylindoles exhibit antibacterial, anticancer, anti-oxidant, anti-inflammatory, anti-diabetic, antiviral, antiproliferative, antituberculosis and antiparkinsonian activities [40]. The amino acid tryptophan is an essential amino acid that is necessary for normal growth in infants and for nitrogen balance in adults. The biogenic amines tryptamine and serotonin as well as the mammalian hormone melatonin are important regulators of psychiatric health [41]. Indole derived marketed drugs include the nonsteroidal anti-inflammatory drug Indomethacin [42,43], anti-HIV drug Delavirdine [44,45], beta-blocker Pindolol [46,47], antintineoplastic drugs Panobinostat [48,49] and Apaziquone [50,51] (Figure 1).

One of the most reliable strategies for the synthesis of fluorinated heterocycles is using of fluorinated building blocks, highly reactive small-molecules. For example, α,β-unsaturated CF_3_-ketones were shown to possess a great potential in the synthesis of various organofluorine compounds, including carbo- and heterocycles [52,53,54,55,56,57,58,59,60,61]. Our group has been deeply involved in this chemistry. Recently, we have reported an efficient approach towards α,β-diaryl-CF_3_-enones—a new type of fluorinated building block. The reaction of arylaldehydes with α-CF_3_-β-aryl enamines gave the corresponding α,β-diaryl-CF_3_-enones in good to high yields at heating in acetic acid. Based on the reactions with hydrazines a convenient pathway to exhaustingly substituted fluorinated pyrazolines and pyrazoles were elaborated, including derivatives of Celecoxib, Mavocoxib (nonsteroidal anti-inflammatory drugs) and SC-560 (anti-cancer drug) [62]. Using reduction of α-aryl-β-(2-nitroaryl)-CF_3_-enones a novel synthetic approach towards 2-CF_3_-3-arylquinolines was developed [63]. Shifting nitro group to α-aryl ring-opened a pathway to various functionalized 2-CF_3_-indoles by the reduction with ammonium formate followed by reactions with various nucleophiles [64].

**Figure 2:**
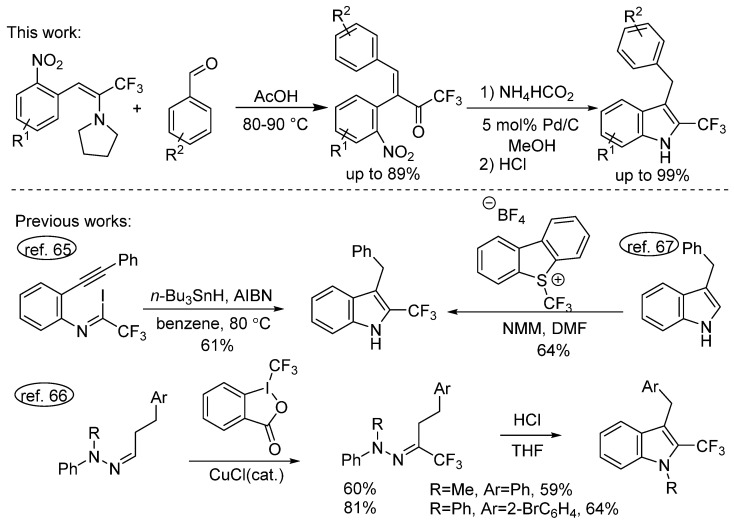
Approaches to 2-CF_3_-3-benzylindoles.

In continuation of the investigation of α,β-diaryl-CF_3_-enones chemistry, in this article, we report synthesis of 2-CF_3_-3-benzylindoles by reduction of nitro group in α-(2-nitroaryl)-β-aryl-CF_3_-enones followed by intramolecular cyclization (Figure 2).

It should be noted, that 2-CF_3_-3-benzylindoles are quite a rare type of indoles. The approaches to the synthesis of these indoles were not studied systematically and have been not in the main focus of the publications. As a result, syntheses of only few 2-CF_3_-3-benzylindoles were reported. Thus, prepared in three steps, *N*-[2-(1-alkynyl)phenyl]trifluoroacetimidoyl iodides were transformed into desired indoles by the tin-radical promoted cyclization of *N*-[2-(1-alkynyl)phenyl]trifluoroacetimidoyl iodides as reported by Uneyama [65]. The copper-catalyzed C(sp2)-H trifluoromethylation of *N*,*N*-disubstituted hydrazones using the Togni’s reagent followed by Fischer indole cyclization of CF_3_-hydrazones formed was described by Monteiro and Bouyssi [66]). *N*-Methylmorpholine mediated direct trifluoromethylation of 3-benzylindole with Umemoto’s reagent was reported by Ma and Yu [67]. In spite of the mentioned methods allowed to prepare 2-CF_3_-indoles in good yields (59–64%), low atom efficiency and high price of some used reagent should be taken into account (Figure 2).

## 2. Results

**Scheme 1:**
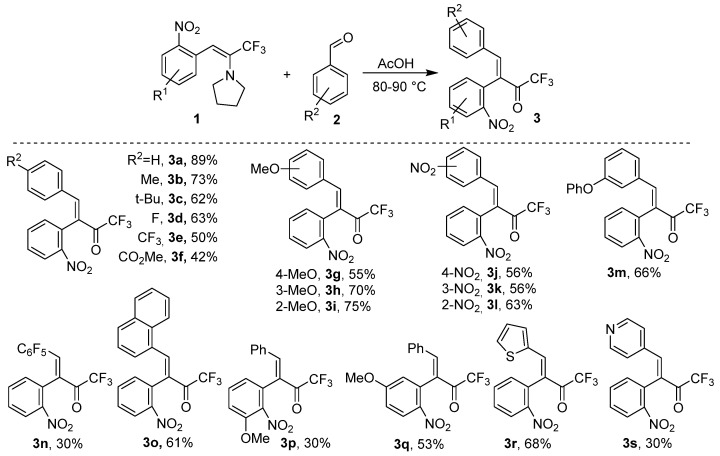
Synthesis of α-(2-nitroaryl)-β-aryl-CF_3_-enones **3**.

To start our investigation, we prepared a set of α-(2-nitroaryl)-β-aryl-CF_3_-enones using recently elaborated by us synthetic protocol [64]. Condensation of α-CF_3_-β-(2-nitroaryl)enamines **1** with arylaldehydes **2** in acetic acid at 80–90 °C led to the corresponding α-(2-nitroaryl)-β-aryl-CF_3_-enones **3** in good to high yields. The reaction is very general, almost no limitations were found to give variety of such enones with a possibility to have different substituents in both aromatic rings. Moreover, some heterocyclic derivatives can be prepared as well (Scheme 1).

**Scheme 2:**
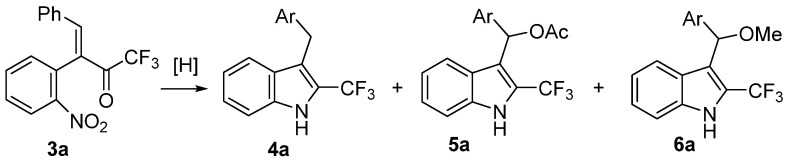
Reduction of ketone **3a** in various conditions.

**Scheme 3:**
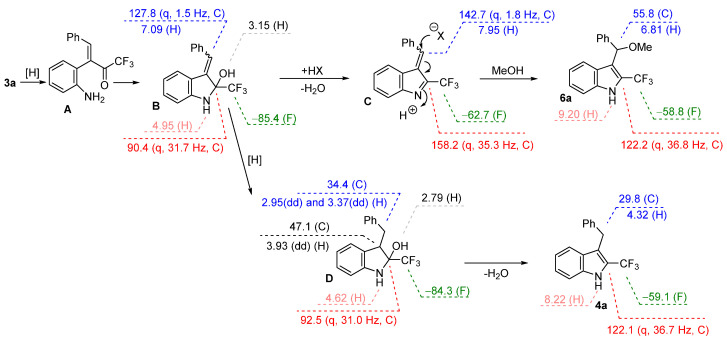
Mechanism of transformation of **3a** into indoles **4a** and **6a**.

Next, we investigated the reductive cyclization of ketone **3a** in various conditions (Scheme 2). Firstly, we employed standard conditions of Leimgruber–Batcho [68] and Reissert [69] synthesis of indoles, which involve the reduction of nitro group followed by intramolecular cyclization of aniline formed. Thus, heating of ketone **3a** using Fe-AcOH-H_2_O, Zn-EtOH-HCl and SnCl_2•_2H_2_O-EtOH systems led to the formation of a variety of hardly identifiable products, in which we were able to identify only 2-CF_3_-3-benzylindole **4a** and its acetoxy-derivative **5a** (by ^19^F NMR, Scheme 2). Better results were achieved when Zn-AcOH system was used. In this case, indoles **4a** and **5a** were isolated in 20% and 47% yield correspondingly (Table 1, entry 1). Further heating of this reaction mixture with additional amount of Zn led to a partial transformation of acetoxy indole **5a** into indole **4a** (Table 1, entry 2). In Zn-AcOH-MeOH system methoxy-indol **6a** became the main product, which was isolated in 77% yield (Table 1, entry 3). Further improvements in terms of chemoselectivity were made using catalytic hydrogenation on Pd/C in MeOH. Thus, reduction using H_2_ at room temperature or NH_4_HCO_2_ (hydrogen surrogate) at 65 °C afforded 2-CF_3_-3-benzylindole **4a** in about 90% yield. In both cases methoxy-substituted indole **6a** was formed as a byproduct in less than 1% yield (Table 1, entries 4,5). Ultimate selectivity of the reaction was achieved by the reduction with 5 equivalents of NH_4_HCO_2_ on Pd/C in MeOH at room temperature. In this conditions 2-CF_3_-3-benzylindole **4a** was isolated in almost quantitative yield while byproduct **6a** was not formed at all (Table 1, entry 6). It is worth noting that the reaction with NH_4_HCO_2_ (Table 1, entries 5,6) leads to a mixture of indole **4a** and indolinol **D**, which structure is proved by NMR spectra of the reaction mixture. However, indolinol **D** eliminates water instantly followed by aromatization after addition of an acid (Scheme 2 and Scheme 3).

Careful analysis of results of experiments (Table 1) forced us to propose that the reaction can proceed via the formation of cyclic hemiaminal **B** (Scheme 3). To confirm our preposition, we performed the reduction of **3a** with 3.3 equivalents of NH_4_HCO_2_ (the precise amount needed for NO_2_ reduction only). Heating of the reaction mixture for 1h at 60 °C led highly selectively to assembling of methoxy-substituted indole **6a** in 86% yield (Table 1, entry 7). We have also found, that using THF as a solvent instead of methanol allowed to stop the reaction at the step of intermediate unsaturated indolinol **B**. Compound **B** is stable enough to be isolated in crude form (by evaporation of the solvent). The structure of **B** was confirmed by NMR and HRMS spectra (Scheme 3). It was also found that compound **B** eliminates water slowly at standing in CDCl_3_ solution (directly in NMR tube). Thus, NMR spectra of this solution measured after about a month (36 days) showed the complete transformation of **B** into **C** (Scheme 3). An attempt to perform acid catalyzed elimination of water from **B** in THF the solution and isolate **C** was failed. Thus, the addition of pTSA to the THF solution of **B** followed by evaporation of the solvent led to severe tarring immediately. However, the addition of pTSA to solution of **B** in methanol led to desired elimination of water followed by the conjugated addition of methanol to form methoxy-indole **5a** (Table 1, entry 8). Similarly, the addition of methanol to CDCl_3_ solution of **C** (obtained by standing in NMR tube, see above) led to the transformation of **C** into **5a** (by ^19^F NMR). So, we have successfully confirmed the mechanism of the reaction. Thus, reduction of the nitro group in indole **3a** led to aniline **A**, which cyclizes to unsaturated indolinol **B**. Elimination of water from **B** afforded conjugated imine **C**, which is a strong Michael acceptor due to aromatization facilitating addition of nucleophiles. Hydrogenation of the double bond of **B** leads to saturated indolinol **D**. Elimination of water from **D** finalizes the process to afford indole **4a**.

**Scheme 4:**
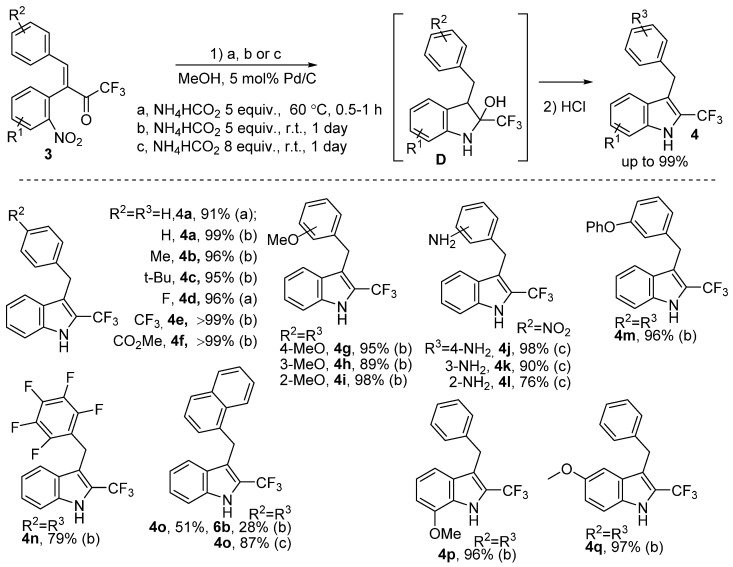
Synthesis of 2-CF_3_-3-benzylindoles **4**.

Next, we investigated the synthetic scope of the synthesis of CF_3_-indoles **4**. Using the optimal reaction conditions, we performed a reduction of a number of ketones **3** to afford corresponding indoles **4** in high to quantitative yields (Scheme 4.).

The reaction has a wide synthetic scope, allowing preparing indoles having both electron-donating and electron-withdrawing groups as well as bulky *ortho*-substituents and naphthyl fragment. It should be noted, that ketones **3j**–**l** bearing the additional nitro groups were transformed into amino-substituted indoles **4j**–**l**. These indoles are interesting objects for the further modifications at NH_2_-group to give promising derivatives in terms of drug design. In the case of bulky ketone **3o** having 1-naphthyl substituent reduction in standard conditions (5 equivalents of NH_4_HCO_2_) led to the formation of admixture of methoxy-indole **6b** (about 28%). Probably, the rate of hydrogenation of the double bond of unsaturated indolinol **B** is lower due to its steric hindrance and the reaction cannot be completed because of full decomposition of NH_4_HCO_2_ on Pd/C during the reaction course. Nevertheless, using of 8 equivalents of NH_4_HCO_2_ allowed to overcome this obstacle to give selectively indole **4o** in 87% yield. The reduction of ketones **3p** and **3q** having additional methoxy group in nitro-aryl fragment led to 5- and 8-methoxyindoles correspondingly.

**Scheme 5:**
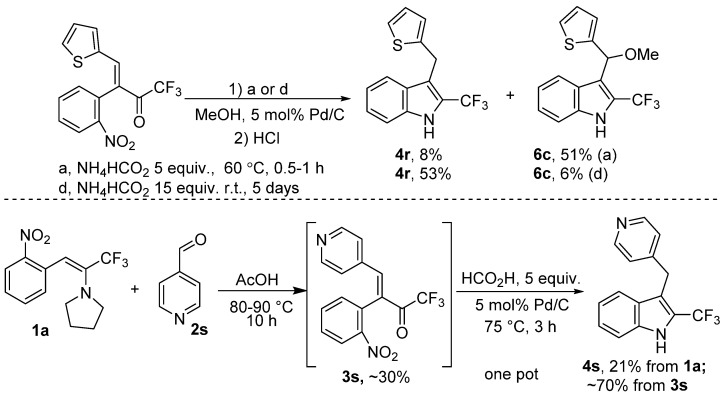
Reduction of ketones **3**, having heterocyclic substituents.

Ketones **3r,s** having heterocyclic substituents were also involved in the transformation. It should be noted that reduction of thiophene derivative **3r** proceeded much more slowly compared to other substrates, which can be explained by poisoning of palladium by thiophene moiety [70]. Thus, attempt to perform the reaction in standard conditions led mostly to methoxy-indole **6c**. However, increasing of the amount of NH_4_HCO_2_ to 15 equivalents and prolongation of the reaction time to 5 days allowed to prepare desired indole **4r** in good yield. Separation of admixture of **6c** from target indole **4r** was carried out by column chromatography. It should be noted, that it is one of few cases, then column chromatography was used for purification of the products (**4l**,**r**,**s**). All other indoles were isolated in pure form just after separation from the inorganic admixtures (Pd/C and NH_4_Cl). Due to the low stability of pyridine derived ketone **3s** the reduction of this compound was performed without its isolation. An attempt to use NH_4_HCO_2_ in AcOH afforded a complex mixture of products. However, using HCO_2_H instead of NH_4_HCO_2_ showed much better results. Indole **4s** having pyridine substituent was isolated in 21% yield from enamine **1a**. Taking into account moderate yield at first step of the reaction sequence (30% for the formation of **3v**) the yield at the reduction step can be estimated as 70% (Scheme 5).

## 3. Materials and Methods

### General Remarks

^1^H, ^13^C and ^19^F NMR spectra were recorded on Bruker AVANCE 400 MHz spectrometer (Bruker Corp., Carlsruhe, Germany) in CD_3_CN and CDCl_3_ at 400, 100 and 376 MHz respectively. Chemical shifts (*δ*) in ppm are reported with the use of the residual CHD_2_CN and chloroform signals (1.94 and 7.25 for ^1^H and 1.30, 77.0 for ^13^C) as internal reference. The ^19^F chemical shifts were referenced to C_6_F_6_, (−162.9 ppm). ESI-MS spectra were measured with an Orbitrap Elite instrument (Thermo Fisher Scientific, Waltham, MA USA). TLC analysis was performed on “Merck 60 F_254_” plates (Merck, Darmstadt, Germany). Column chromatography was performed on silica gel. Melting points were determined on an Electrothermal 9100 apparatus (Electrothermal, Stone, Staffordshire, UK). All reagents were of reagent grade and were used as such or were distilled prior to use. Starting α-CF_3_-β-aryl enamines **1** were synthesized using previously reported procedures by the reaction with 10 equivalents of pyrrolidine in neat [71].

**Synthesis of α-CF_3_-β-(2-nitroaryl)enamines 1 by the Reaction with Pyrrolidine in Neat (General Procedure).** One neck 25 mL round-bottomed flask was charged with dry pyrrolidine (8.5 mL, 100 mmol), cooled down to −18 °C and the corresponding styrene (10 mmol) was added in one portion with vigorous stirring. The reaction mixture was stirred at room temperature for 1-3 h until starting styrene was consumed (TLC or NMR monitoring). The excess of pyrrolidine was evaporated in a vacuum, the viscous residue was dissolved in CH_2_Cl_2_ (50 mL), washed with 10% K_2_CO_3_ solution (2 × 50 mL) and dried over Na_2_SO_4_. CH_2_Cl_2_ was removed in vacuo to give crude enamine, which was used without further purification. For characterization data of enamines **1** see [64].

**Synthesis of ketones 3 by the reactions of α-(trifluoromethyl)enamines with aromatic aldehydes (general procedure).** One-necked 50-mL round bottom flask (or 12 mL vial) was charged with enamine **1** (5 mmol), aromatic aldehyde **2** (5.75 mmol) and glacial acetic acid (15 mL or 5 mL for reaction in the vial). Reaction mixture was kept at 80–90 °C (hotplate stirrer) under stirring for 6-10 h until consumption of aldehyde and corresponding benzyl ketone formed by the hydrolysis of enamine (^1^H NMR control). Volatiles were evaporated in vacuo, the residue was dissolved in CH_2_Cl_2_ (50 mL), washed with water (2 × 20 mL) and dried over Na_2_SO_4_. Volatiles were evaporated in vacuo, the residue was purified by column chromatography, using mixtures of hexane and CH_2_Cl_2_ (3:1, 1:1), CH_2_Cl_2_, mixture of CH_2_Cl_2_ and MeOH (100:1) as eluents. For characterization data of ketones **3** see [64].

**Reductive cyclization of nitro-ketones 3 to 2-CF_3_-indoles 4.** 12 mL vial with a screw cap was charged with ketone **4** (0.2 mmol), NH_4_HCO_2_ (0.063 g, 1.00 mmol, 5 equiv.), Pd/C (10%, 0.0108 g, 0.01 mmol, 5 mol%) and methanol (1.2 mL). Next, the reaction mixture was kept under stirring at 60 °C for 0.5-1 h (conditions a) or at room temperature for 1 day (conditions b). After that, 6M HCl (0.25 mL, 1.5 mmol) was added in 4-5 portions (evolution of CO_2_!). The reaction mixture was filtered through a short celite pad and dispersed between water (10 mL) and CH_2_Cl_2_ (20 mL). Aqueous layer was separated and extracted with CH_2_Cl_2_ (3 × 10 mL). Combined organic phases were dried over Na_2_SO_4_, volatiles were evaporated in vacuo, to give pure indole **4**. In the case of indoles **4l**, **4n, 4r**, **4s** additional purification by column chromatography on silica gel was performed. Reduction of ketones **3j**–**l**, having additional nitro group, was performed using 8 equivalents of NH_4_HCO_2_ at room temperature (conditions c).

*3-Benzyl-2-(trifluoromethyl)-1H-indole* (**4a**). Obtained using conditions a (0.108 g, 0.34 mmol of **3a**) or conditions b (0.055 g, 0.171 mmol of **3a**). Pale brown crystals, m.p. 103–104 °C, yield 0.084 g (91%, A) 0.0465 g (99%, B). ^1^H NMR (CDCl_3_, 400.1 MHz): δ 8.25 (br.s, 1H), 7.57 (d, 1H, ^3^*J* = 8.1 Hz), 7.39 (d, 1H, ^3^*J* = 8.2 Hz), 7.27–7.37 (m, 5H), 7.20–7.26 (m, 1H), 7.13–7.19 (m, 1H), 4.32 (s, 2H). ^13^C{^1^H} NMR (CDCl_3_, 100.6 MHz): δ 139.9, 135.3, 128.4, 128.3, 127.4, 126.1, 124.8, 122.03 (q, ^2^*J*_CF_ = 36.5 Hz), 122.01 (q, ^1^*J*_CF_ = 269.0 Hz), 120.8, 120.7, 116.8 (q, ^3^*J*_CF_ = 2.8 Hz), 111.7, 29.8. ^19^F NMR (CDCl_3_, 376.5 MHz): δ −59.1 (s, 3F). NMR data are in agreement with those in the literature [67].

*3-(4-Methylbenzyl)-2-(trifluoromethyl)-1H-indole* (**4b**). Obtained using conditions a (0.109 g, 0.325 mmol of **3b**). Pale brown solid, m.p. 88–90 °C, yield 0.090 g (96%). ^1^H NMR (CDCl_3_, 400.1 MHz): δ 8.24 (br.s, 1H), 7.63 (d, 1H, ^3^*J* = 8.1 Hz), 7.35–7.44 (m, 2H), 7.25 (d, 2H, ^3^*J* = 8.0 Hz), 7.19–7.23 (m, 1H), 7.17 (d, 2H, ^3^*J* = 7.9 Hz), 4.33 (s, 2H), 2.39 (s, 3H). ^13^C{^1^H} NMR (CDCl_3_, 100.6 MHz): δ 136.9, 135.6, 135.3, 129.1, 128.1, 127.4, 124.7, 122.1(q, ^1^*J*_CF_ = 269.0 Hz), 121.9 (q, ^2^*J*_CF_ = 36.5 Hz), 120.8, 120.6, 117.0 (q, ^3^*J*_CF_ = 2.8 Hz), 111.6, 29.3, 20.9. ^19^F NMR (CDCl_3_, 376.5 MHz): δ −59.1 (s, 3F). HRMS (ESI-TOF): *m*/*z* [M − H]^−^ Calcd for C_17_H_13_F_3_N^−^: 288.1006; found: 288.1009.

*3-(4-(tert-Butyl)benzyl)-2-(trifluoromethyl)-1H-indole* (**4c**). Obtained using conditions b (0.120 g, 0.318 mmol of **3c**). Pale brown solid, m.p. 85–87 °C, yield 0.100 g (95%). ^1^H NMR (CDCl_3_, 400.1 MHz): δ 8.23 (br.s, 1H), 7.64 (d, 1H, ^3^*J* = 8.1 Hz), 7.33–7.42 (m, 4H), 7.25–7.32 (m, 2H), 7.19 (ddd, 1H, ^3^*J* = 8.0 Hz, ^3^*J* = 6.6 Hz, ^4^*J* = 1.4 Hz), 4.32 (s, 2H), 1.36 (s, 9H). ^13^C{^1^H} NMR (CDCl_3_, 100.6 MHz): δ 148.9, 136.9, 135.3, 127.9, 127.5, 125.3, 124.8, 122.0 (q, ^1^*J*_CF_ = 269.1 Hz), 121.9 (q, ^2^*J*_CF_ = 36.7 Hz), 120.9, 120.6, 117.1 (q, ^3^*J*_CF_ = 2.6 Hz), 111.6, 34.3, 31.3, 29.2. ^19^F NMR (CDCl_3_, 376.5 MHz): δ −59.0 (s, 3F). HRMS (ESI-TOF): *m*/*z* [M − H]^−^ Calcd for C_20_H_19_F_3_N^−^: 330.1475; found: 330.1472.

*3-(4-Fluorobenzyl)-2-(trifluoromethyl)-1H-indole* (**4d**). Obtained using conditions a (0.126 g, 0.372 mmol of **3d**). Brown viscous oil, yield 0.105 g (96%). ^1^H NMR (CDCl_3_, 400.1 MHz): δ 8.30 (br.s, 1H), 7.56 (d, 1H, ^3^*J* = 8.1 Hz), 7.33–7.45 (m, 2H), 7.16–7.28 (m, 3H), 6.94–7.05 (m, 2H), 4.29 (s, 2H). ^13^C{^1^H} NMR (CDCl_3_, 100.6 MHz): δ 161.3 (d, ^1^*J*_CF_ = 243.8 Hz), 135.6 (d, ^4^*J*_CF_ = 2.8 Hz), 135.3, 129.6 (d, ^3^*J*_CF_ = 7.9 Hz), 127.3, 124.9, 122.0 (q, ^2^*J*_CF_ = 36.4 Hz), 121.9 (q, ^1^*J*_CF_ = 269.0 Hz), 120.8, 120.6, 116.6 (q, ^3^*J*_CF_ = 2.6 Hz), 115.1 (d, ^2^*J*_CF_ = 21.3 Hz), 111.7, 28.9. ^19^F NMR (CDCl_3_, 376.5 MHz): δ −59.1 (s, 3F), −118.19 – −118.55 (m, 1F). HRMS (ESI-TOF): *m*/*z* [M − H]^−^ Calcd for C_16_H_10_F_4_N^−^: 292.0755; found: 292.0749.

*2-(Trifluoromethyl)-3-(4-(trifluoromethyl)benzyl)-1H-indole* (**4e**). Obtained using conditions b (0.147 g, 0.378 mmol of **3e**). Pale brown solid, m.p. 54–56 °C, yield 0.129 g (>99%). ^1^H NMR (CDCl_3_, 400.1 MHz): δ 8.36 (br.s, 1H), 7.50–7.66 (m, 3H), 7.40–7.49(m, 1H), 7.39-7.40 (m, 3H), 7.19 (t, 1H, ^3^*J* = 7.5 Hz),4.35 (s, 2H). ^13^C{^1^H} NMR (CDCl_3_, 100.6 MHz): δ 144.0, 135.3, 128.49 (q, ^2^*J*_CF_ = 32.3 Hz), 128.51, 127.2, 125.4 (q, ^3^*J*_CF_ = 3.7 Hz), 124.3 (q, ^1^*J*_CF_ = 271.9 Hz), 122.4 (q, ^2^*J*_CF_ = 36.8 Hz), 121.9 (q, ^1^*J*_CF_ = 269.1 Hz), 121.0, 120.4, 115.6 (q, ^3^*J*_CF_ = 2.6 Hz), 111.9, 29.5. ^19^F NMR (CDCl_3_, 376.5 MHz): δ −59.1 (s, 3F), −63.4 (s, 3F). HRMS (ESI-TOF): *m*/*z* [M − H]^−^ Calcd for C_17_H_10_F_6_N^−^: 342.0723; found: 342.0714.

*Methyl 4-((2-(trifluoromethyl)-1H-indol-3-yl)methyl)benzoate* (**4f**). Obtained using conditions b (0.085 g, 0.224 mmol of **3f**). Pale yellow solid, m.p. 109–111 °C, yield 0.074 g (>99%). ^1^H NMR (CDCl_3_, 400.1 MHz): δ 8.69 (br.s, 1H), 7.95 (d, 2H, ^3^*J* = 8.3 Hz), 7.48 (d, 2H, ^3^*J* = 8.1 Hz), 7.41 (d, 1H, ^3^*J* = 8.3 Hz), 7.27–7.35 (m, 3H), 7.10–7.16 (m, 1H), 4.32 (s, 2H), 3.90 (s, 3H). ^13^C{^1^H} NMR (CDCl_3_, 100.6 MHz): δ 167.2, 145.5, 135.4, 129.8, 128.3, 128.0, 127.2, 124.9, 122.3 (q, ^2^*J*_CF_ = 36.8 Hz), 121.9 (q, ^1^*J*_CF_ = 269.0 Hz), 120.8, 120.4, 115.5 (q, ^3^*J*_CF_ = 2.9 Hz), 111.8, 52.0, 29.8. ^19^F NMR (CDCl_3_, 376.5 MHz): δ −59.1 (s, 3F). HRMS (ESI-TOF): *m*/*z* [M − H]^−^ Calcd for C_18_H_13_F_3_NO_2_^−^: 332.0904; found: 332.0904.

*3-(4-Methoxybenzyl)-2-(trifluoromethyl)-1H-indole* (**4g**). Obtained using conditions b (0.057 g, 0.161 mmol of **3g**). White powder, m.p. 116–118 °C, yield 0.047 g (95%). ^1^H NMR (CDCl_3_, 400.1 MHz): δ 8.34 (br.s, 1H), 7.56 (d, 1H, ^3^*J* = 8.1 Hz), 7.37 (d, 1H, ^3^*J* = 8.2 Hz), 7.32 (t, 1H, ^3^*J* = 7.5 Hz), 7.20 (d, 2H, ^3^*J* = 8.5 Hz), 7.11–7.17 (m, 1H), 6.84 (d, 2H, ^3^*J* = 8.6 Hz), 4.24 (s, 2H), 3.79 (s, 3H). ^13^C{^1^H} NMR (CDCl_3_, 100.6 MHz): δ 157.8, 135.3, 132.1, 129.2, 127.4, 124.8, 122.0 (q, ^1^*J*_CF_ = 268.9 Hz), 121.9 (q, ^2^*J*_CF_ = 36.7 Hz), 120.8, 120.6, 117.2 (q, ^3^*J*_CF_ = 2.8 Hz), 113.8, 111.7, 55.2, 28.9. ^19^F NMR (CDCl_3_, 376.5 MHz): δ −59.1 (s, 3F). HRMS (ESI-TOF): *m*/*z* [M − H]^−^ Calcd for C_17_H_13_F_3_NO^−^: 304.0955; found: 304.0945.

*3-(3-Methoxybenzyl)-2-(trifluoromethyl)-1H-indole* (**4h**). Obtained using conditions b (0.104 g, 0.296 mmol of **3h**). Pale brown solid, m.p. 55–57 °C, yield 0.080 g (89%). ^1^H NMR (CDCl_3_, 400.1 MHz): δ 8.35 (br.s, 1H), 7.57 (d, 1H, ^3^*J* = 8.1 Hz), 7.28–7.38 (m, 2H), 7.21 (d, 1H, ^3^*J* = 7.9 Hz), 7.12–7.17 (m, 1H), 6.90 (d, 1H, ^3^*J* = 7.7 Hz), 6.85 (*pseudo*-s, 1H), 6.77 (dd, 1H, ^3^*J* = 8.2 Hz, ^4^*J* = 2.3 Hz), 4.28 (s, 2H), 3.77 (s, 3H). ^13^C{^1^H} NMR (CDCl_3_, 100.6 MHz): δ 159.6, 141.6, 135.3, 129.3, 127.4, 124.8, 122.01 (q, ^2^*J*_CF_ = 36.5 Hz), 121.99 (q, ^1^*J*_CF_ = 269.1 Hz), 120.8, 120.70, 120.65, 116.5 (q, ^3^*J*_CF_ = 2.9 Hz), 114.3, 111.7, 111.2, 55.0, 29.7. ^19^F NMR (CDCl_3_, 376.5 MHz): δ −59.0 (s, 3F). HRMS (ESI-TOF): *m*/*z* [M − H]^−^ Calcd for C_17_H_13_F_3_NO^−^: 304.0955; found: 304.0953.

*3-(2-Methoxybenzyl)-2-(trifluoromethyl)-1H-indole* (**4i**). Obtained using conditions b (0.116 g, 0.330 mmol of **3i**). Pale yellow solid, m.p. 67–69 °C, yield 0.099 g (98%). ^1^H NMR (CDCl_3_, 400.1 MHz): δ 8.28 (br.s, 1H), 7.58 (d, 1H, ^3^*J* = 8.1 Hz), 7.31–7.41 (m, 2H), 7.20–7.25 (m, 1H), 7.15 (ddd, 1H, ^3^*J* = 8.0 Hz, ^3^*J* = 6.8 Hz, ^4^*J* = 1.2 Hz), 6.96–7.02 (m, 1H), 6.94 (dd, 1H, ^3^*J* = 8.2 Hz, ^4^*J* = 0.7 Hz), 6.85 (td, 1H, ^3^*J* = 7.5 Hz, ^4^*J* = 1.0 Hz), 4.34 (s, 2H), 3.94 (s, 3H). ^13^C{^1^H} NMR (CDCl_3_, 100.6 MHz): δ 157.0, 135.3, 129.2, 128.2, 127.7, 127.2, 124.7 122.3 (q, ^2^*J*_CF_ = 36.7 Hz), 122.1 (q, ^1^*J*_CF_ = 269.0 Hz), 121.0, 120.5, 120.4, 116.5 (q, ^3^*J*_CF_ = 2.7 Hz), 111.5, 109.9, 55.2, 23.3. ^19^F NMR (CDCl_3_, 376.5 MHz): δ −59.3 (s, 3F). HRMS (ESI-TOF): *m*/*z* [M − H]^−^ Calcd for C_17_H_13_F_3_NO^−^: 304.0955; found: 304.0951.

*4-((2-(Trifluoromethyl)-1H-indol-3-yl)methyl)aniline* (**4j**). Obtained using conditions c (0.112 g, 0.306 mmol of **3j**). Pale brown solid, m.p. 175–177 °C, yield 0.087 g (98%). ^1^H NMR (CDCl_3_, 400.1 MHz): δ 9.87 (br.s, 1H), 7.55 (d, 1H, ^3^*J* = 8.1 Hz), 7.46 (d, 1H, ^3^*J* = 8.3 Hz), 7.28 (t, 1H, ^3^*J* = 7.6 Hz), 7.09 (ddd, 1H, ^3^*J* = 8.0 Hz, ^3^*J* = 7.1 Hz, ^4^*J* = 0.9 Hz), 6.95 (d, 2H, ^3^*J* = 8.4 Hz), 6.54 (d, 2H, ^3^*J* = 8.5 Hz), 4.11 (s, 2H), 3.98 (br.s, 2H). ^13^C{^1^H} NMR (CDCl_3_, 100.6 MHz): δ 146.9, 136.8, 130.0, 129.7, 127.9, 125.4, 123.4 (q, ^1^*J*_CF_ = 268.1 Hz), 122.1 (q, ^2^*J*_CF_ = 36.6 Hz), 121.4, 121.0, 115.4, 112.9, 29.2. ^19^F NMR (CDCl_3_, 376.5 MHz): δ −56.8 (s, 3F). HRMS (ESI-TOF): *m*/*z* [M + H]^+^ Calcd for C_16_H_14_F_3_N_2_^+^: 291.1104; found: 291.1110.

*3-((2-(Trifluoromethyl)-1H-indol-3-yl)methyl)aniline* (**4k**). Obtained using conditions c (0.120 g, 0.328 mmol of **3k**). Pale yellow solid, m.p. 138–140 °C, yield 0.086 g (90%). ^1^H NMR (CDCl_3_, 400.1 MHz): δ 8.56 (br.s, 1H), 7.56 (d, 1H, ^3^*J* = 8.1 Hz), 7.26–7.35 (m, 2H), 7.05–7.15 (m, 2H), 6.72 (d, 2H, ^3^*J* = 7.6 Hz), 6.57 (br.s, 1H), 6.53 (dd, 1H, ^3^*J* = 7.9 Hz, ^3^*J* = 1.7 Hz), 4.20 (s, 2H), 3.54 (br.s, 2H). ^13^C{^1^H} NMR (CDCl_3_, 100.6 MHz): δ 146.2, 141.3, 135.3, 129.2, 127.5, 124.7, 121.97 (q, ^2^*J*_CF_ = 36.5 Hz), 122.02 (q, ^1^*J*_CF_ = 268.7 Hz), 120.8, 120.5,118.9, 116.6 (q, ^3^*J*_CF_ = 2.4 Hz), 115.2, 113.2, 111.6, 29.65. ^19^F NMR (CDCl_3_, 376.5 MHz): δ −58.9 (s, 3F). HRMS (ESI-TOF): *m*/*z* [M + H]^+^ Calcd for C_16_H_14_F_3_N_2_^+^: 291.1104; found: 291.1111.

*2-((2-(Trifluoromethyl)-1H-indol-3-yl)methyl)aniline* (**4l**). Obtained using conditions c (0.160 g, 0.437 mmol of **3l**). Purified by column chromatography, using gradient elution by CH_2_Cl_2_ followed by mixture CH_2_Cl_2_-MeOH (100:1, 30:1). Pale yellow solid, m.p. 136–138 °C, yield 0.097 g (76%). ^1^H NMR (CDCl_3_, 400.1 MHz): δ 8.50 (br.s, 1H), 7.40 (d, 1H, ^3^*J* = 8.1 Hz), 7.35 (d, 1H, ^3^*J* = 8.2 Hz), 7.29 (t, 1H, ^3^*J* = 7.5 Hz), 7.08–7.12 (m, 1H), 7.04–7.08 (m, 1H), 6.93 (d, 1H, ^3^*J* = 7.5 Hz), 6.67–6.75 (m, 2H), 4.13 (s, 2H), 3.53 (br.s, 1H). ^13^C{^1^H} NMR (CDCl_3_, 100.6 MHz): δ 144.2, 135.3, 129.5, 127.5, 127.4, 124.9, 123.9, 122.4 (q, ^2^*J*_CF_ = 36.8 Hz), 121.9 (q, ^1^*J*_CF_ = 269.0 Hz), 120.9, 120.7, 118.8, 115.7, 115.0 (q, ^3^*J*_CF_ = 2.9 Hz), 111.7, 25.9. ^19^F NMR (CDCl_3_, 376.5 MHz): δ −59.3 (s, 3F). HRMS (ESI-TOF): *m*/*z* [M + H]^+^ Calcd for C_16_H_14_F_3_N_2_^+^: 291.1104; found: 291.1104.

*3-(3-Phenoxybenzyl)-2-(trifluoromethyl)-1H-indole* (**4m**). Obtained using conditions b (0.126 g, 0.305 mmol of **3m**). Pale yellow solid, m.p. 71–73 °C, yield 0.107 g (96%). ^1^H NMR (CDCl_3_, 400.1 MHz): δ 8.33 (br.s, 1H), 7.56 (d, 1H, ^3^*J* = 8.1 Hz), 7.31–7.41 (m, 4H), 7.22-7.27 (m, 1H), 7.10–7.20 (m, 2H), 6.97-7.07 (m, 4H), 6.86 (dd, 1H, ^3^*J* = 8.1 Hz, ^4^*J* = 1.7 Hz), 4.29 (s, 2H). ^13^C{^1^H} NMR (CDCl_3_, 100.6 MHz): δ 157.14, 157.11, 142.1, 135.3, 129.6, 127.3, 124.8, 123.3, 123.1, 122.1 (q, ^2^*J*_CF_ = 36.7 Hz), 121.9 (q, ^1^*J*_CF_ = 269.0 Hz), 120.69, 120.65, 119.1, 118.7, 116.5, 116.3 (q, ^3^*J*_CF_ = 2.5 Hz), 111.7, 29.6. ^19^F NMR (CDCl_3_, 376.5 MHz): δ −59.1 (s, 3F). HRMS (ESI-TOF): *m*/*z* [M − H]^−^ Calcd for C_22_H_15_F_3_NO^−^: 366.1111; found: 366.1107.

*3-((Perfluorophenyl)methyl)-2-(trifluoromethyl)-1H-indole* (**4n**). Obtained using conditions b (0.117 g, 0.285 mmol of **3n**). Purified by column chromatography, using gradient elution by mixture hexane-CH_2_Cl_2_ (4:1) followed by mixture hexane-CH_2_Cl_2_ (2:1). Pale brown solid, m.p. 131–133 °C, yield 0.082 g (79%). ^1^H NMR (CDCl_3_, 400.1 MHz): δ 8.32 (br.s, 1H), 7.56 (d, 1H, ^3^*J* = 8.1 Hz), 7.37–7.42 (m, 1H), 7.29–7.36 (m, 1H), 7.19 (ddd, 1H, ^3^*J* = 8.1 Hz, ^3^*J* = 6.9 Hz, ^4^*J* = 1.1 Hz), 4.32 (s, 2H). ^13^C{^1^H} NMR (CDCl_3_, 100.6 MHz): δ 145.3 (dddt, ^1^*J*_CF_ = 246.8 Hz, ^3^*J*_CF_ = 11.8 Hz, ^4^*J*_CF_ = 7.8 Hz, ^5^*J*_CF_ = 3.8 Hz, CF), 140.04 (dm, ^1^*J*_CF_ = 258.6 Hz, m_1_ 141.5-141.1, m_2_ 138.9-138.6, CF), 137.5 (dm, ^1^*J*_CF_ = 257.8 Hz, m_1_ 138.9–138.6, m_2_ 136.5–136.1, CF), 135.0, 126.6, 125.1, 122.3 (q, ^2^*J*_CF_ = 37.4 Hz), 121.7 (q, ^1^*J*_CF_ = 269.1 Hz), 121.1, 119.7, 113.1, 112.9, 111.9, 29.8 (d, ^3^*J*_CF_ = 20.6 Hz, CF). ^19^F NMR (CDCl_3_, 376.5 MHz): δ −59.7 (s, 3F), −142.98 – −143.23 (m, 2F), −157.9 (t, 1F, *J* = 20.8 Hz), −163.47 – −163.67 (m, 2F). HRMS (ESI-TOF): *m*/*z* [M − H]^−^ Calcd for C_16_H_6_F_8_N^−^: 364.0378; found: 364.0373.

*3-(Naphthalen-1-ylmethyl)-2-(trifluoromethyl)-1H-indole* (**4o**). Obtained using conditions b (0.043 g, 0.116 mmol of **3o**) and 8 equivalents of NH_4_HCO_2_ (0.059 g, 0.94 mmol, 8 equiv.). White solid, m.p. 69–71 °C, yield 0.0328 g (87%). ^1^H NMR (CDCl_3_, 400.1 MHz): δ 8.37 (br.s, 1H), 8.29 (d, 1H, ^3^*J* = 8.4 Hz), 7.90–7.98 (m, 1H), 7.76 (d, 1H, ^3^*J* = 8.2 Hz), 7.59–7.66 (m, 1H), 7.53-7.59(m, 1H), 7.43 (d, 1H, ^3^*J* = 8.3 Hz), 07.29-7.39 (m, 3H), 7.08 (ddd, 1H, ^3^*J* = 8.0 Hz, ^3^*J* = 7.1 Hz, ^4^*J* = 0.9 Hz), 7.04 (dd, 1H, ^3^*J* = 7.1 Hz, ^4^*J* = 0.9 Hz), 4.78 (s, 2H). ^13^C{^1^H} NMR (CDCl_3_, 100.6 MHz): δ 135.4, 135.3, 133.6, 131.9, 128.8, 127.7, 126.9, 126.1, 125.6, 125.5, 125.4, 124.9, 123.2, 122.7 (q, ^2^*J*_CF_ = 36.7 Hz), 122.0 (q, ^1^*J*_CF_ = 269.3 Hz), 120.9, 120.7, 115.7 (q, ^3^*J*_CF_ = 2.9 Hz), 111.7, 26.6. ^19^F NMR (CDCl_3_, 376.5 MHz): δ −59.7 (s, 3F). HRMS (ESI-TOF): *m*/*z* [M − H]^−^ Calcd for C_20_H_13_F_3_N^−^: 324.1006; found: 324.1002.

*3-(Methoxy(naphthalen-1-yl)methyl)-2-(trifluoromethyl)-1H-indole* (**6b**). Obtained using conditions b (0.153 g, 0.412 mmol of **3o**) as a mixture with indole **4o** (yield 0.069 g (51%) for **4o**). Purified by column chromatography, using mixture of hexane and CH_2_Cl_2_ (1:1) as an eluent. Yellow powder, m.p. 65–67 °C, yield 0.041 g (28%). ^1^H NMR (CDCl_3_, 400.1 MHz): δ 8.53 (br.s, 1H), 8.17 (d, 1H, ^3^*J* = 8.5 Hz), 7.88 (dd, 1H, ^3^*J* = 8.3 Hz, ^4^*J* = 0.9 Hz), 7.81 (d, 1H, ^3^*J* = 8.0 Hz), 7.74 (d, 1H, ^3^*J* = 8.2 Hz), 7.53–7.60 (m, 1H), 7.47–7.53 (m, 1H), 7.40 (d, 2H, ^3^*J* = 8.2 Hz), 7.36 (d, 1H, ^3^*J* = 7.9 Hz), 7.28–7.34 (m, 1H), 7.06-7.13 (m, 1H), 6.51 (s, 1H), 3.54 (s, 3H). ^13^C{^1^H} NMR (CDCl_3_, 100.6 MHz): δ 135.6, 135.3, 134.0, 131.6, 128.9, 128.7, 126.5, 126.3, 125.6, 125.3, 124.98, 124.94, 123.8, 123.3 (q, ^2^*J*_CF_ = 37.4 Hz), 123.0, 121.7 (q, ^1^*J*_CF_ = 269.5 Hz), 121.2, 116.5 (q, ^3^*J*_CF_ = 2.6 Hz), 111.7, 75.5, 57.2. ^19^F NMR (CDCl_3_, 376.5 MHz): δ −59.0 (s, 3F). HRMS (ESI-TOF): *m*/*z* [M − OMe]^−^ Calcd for C_20_H_14_F_3_N^−^: 324.1002; found: 324.1006.

*3-Benzyl-7-methoxy-2-(trifluoromethyl)-1H-indole* (**4p**). Obtained using conditions b (0.053 g, 0.151 mmol of **3p**). Green-yellowish viscous oil, yield 0.044 g (96%). ^1^H NMR (CDCl_3_, 400.1 MHz): δ 8.57 (br.s, 1H), 7.23–7.30 (m, 4H), 7.15–7.22 (m,1H), 7.11 (d, 1H, ^3^*J* = 8.1 Hz), 7.04 (t, 1H, ^3^*J* = 7.6 Hz), 6.72 (d, 1H, ^3^*J* = 7.6 Hz), 4.26 (s, 2H), 3.97 (s, 3H). ^13^C{^1^H} NMR (CDCl_3_, 100.6 MHz): δ 146.3, 140.0, 128.7, 128.4, 128.3, 126.3, 126.0, 122.0 (q, ^1^*J*_CF_ = 268.9 Hz), 121.8 (q, ^2^*J*_CF_ = 36.8 Hz), 121.2, 117.1 (q, ^3^*J*_CF_ = 2.8 Hz), 113.1, 103.9, 55.4, 30.0. ^19^F NMR (CDCl_3_, 376.5 MHz): δ −59.1 (s, 3F). HRMS (ESI-TOF): *m*/*z* [M − H]^−^ Calcd for C_17_H_13_F_3_NO^−^: 304.0955; found: 304.0960.

*3-Benzyl-5-methoxy-2-(trifluoromethyl)-1H-indole* (**4q**). Obtained using conditions b (0.098 g, 0.279 mmol of **3q**). Pale brown solid, m.p. 102–104 °C, yield 0.0826 g (97%). ^1^H NMR (CDCl_3_, 400.1 MHz): δ 8.29 (br.s, 1H), 7.24–7.33 (m, 5H), 7.19–7.24 (m,1H), 6.99 (dd, 1H, ^3^*J* = 8.9 Hz, ^4^*J* = 2.4 Hz), 6.92 (d, 1H, ^4^*J* = 2.4 Hz), 4.27 (s, 2H), 3.78 (s,3H). ^13^C{^1^H} NMR (CDCl_3_, 100.6 MHz): δ 154.5, 139.9, 130.5, 128.4, 128.3, 127.9, 126.1, 122.6 (q, ^2^*J*_CF_ = 36.7 Hz), 121.9 (q, ^1^*J*_CF_ = 269.0 Hz), 116.2 (q, ^3^*J*_CF_ = 2.4 Hz), 115.6, 112.6, 101.6, 55.7, 29.8. ^19^F NMR (CDCl_3_, 376.5 MHz): δ −59.2 (s, 3F). HRMS (ESI-TOF): *m*/*z* [M − H]^−^ Calcd for C_17_H_13_F_3_NO^−^: 304.0955; found: 304.0946.

**Reduction of ketone 3r.** Using conditions a: 12 mL vial with a screw cap was charged with ketone **3r** (0.072 g, 0.220 mmol), NH_4_HCO_2_ (0.069 g, 1.10 mmol, 5 equiv.), Pd/C (10%, 0.012 g, 0.011 mmol, 5 mol%) and methanol (1.5 mL). Next, the reaction mixture was kept under stirring at 60 °C for 1 h. After that, 6M HCl (0.25 mL, 1.5 mmol) was added in 4-5 portions (evolution of CO_2_!). The reaction mixture was filtered through a short celite pad and dispersed between water (10 mL) and CH_2_Cl_2_ (20 mL). Aqueous layer was separated and extracted with CH_2_Cl_2_ (3 × 10 mL). Combined organic phases were dried over Na_2_SO_4_, volatiles were evaporated in vacuo, the residue was purified by column chromatography on silica gel, using gradient elution by mixture hexane-CH_2_Cl_2_ (4:1) followed by mixture hexane-CH_2_Cl_2_ (2:1) to give 3-(thiophen-2-ylmethyl)-2-(trifluoromethyl)-1H-indole **(4r)**, yield 0.0048 g, (8%) and 3-(methoxy(thiophen-2-yl)methyl)-2-(trifluoromethyl)-1H-indole **(5a)**, yield 0.035 g, (51%)**.**

Using conditions d: 12 mL vial with a screw cap was charged with ketone **3r** (0.068 g, 0.208 mmol), NH_4_HCO_2_ (0.188 g, 2.98 mmol, ~15 equiv.), Pd/C (10%, 0.011 g, 0.0104 mmol, 5 mol%) and methanol (3 mL). Next, the reaction mixture was kept under stirring for 1 day. After that, 6M HCl (0.5 mL, 3 mmol) was added in 4-5 portions (evolution of CO_2_!). The reaction mixture was filtered through a short celite pad and dispersed between water (10 mL) and CH_2_Cl_2_ (20 mL). Aqueous layer was separated and extracted with CH_2_Cl_2_ (3 × 10 mL). Combined organic phases were dried over Na_2_SO_4_, volatiles were evaporated in vacuo, the residue was purified by column chromatography on silica gel, using gradient elution by mixture hexane-CH_2_Cl_2_ (4:1) followed by mixture hexane-CH_2_Cl_2_ (2:1) to give 3-(thiophen-2-ylmethyl)-2-(trifluoromethyl)-1H-indole **(4r)**, yield 0.031 g, (53%) and 3-(methoxy(thiophen-2-yl)methyl)-2-(trifluoromethyl)-1H-indole **(5a)**, yield 0.0038 g, (6%)**.**

*3-(Thiophen-2-ylmethyl)-2-(trifluoromethyl)-1H-indole* (**4r**). White powder, m.p. 88–90 °C. ^1^H NMR (CDCl_3_, 400.1 MHz): δ 8.26 (br.s, 1H), 7.63 (d, 1H, ^3^*J* = 8.1 Hz), 7.37–7.42 (m, 1H), 7.29-7.35 (m, 1H), 7.17 (ddd, 1H, ^3^*J* = 8.0 Hz, ^3^*J* = 7.0 Hz, ^4^*J* = 1.0 Hz), 7.10 (dd, 1H, ^3^*J* = 5.1 Hz, ^4^*J* = 1.2 Hz), 6.89 (dd, 1H, ^3^*J* = 5.1 Hz, ^4^*J* = 3.5 Hz), 6.80-6.86 (m,1H), 4.44 (s, 2H). ^13^C{^1^H} NMR (CDCl_3_, 100.6 MHz): δ 143.0, 135.2, 127.0, 126.7, 125.0, 124.8, 123.6, 121.8 (q, ^1^*J*_CF_ = 269.0 Hz), 121.7 (q, ^2^*J*_CF_ = 36.8 Hz), 120.8, 120.5, 116.4 (q, ^3^*J*_CF_ = 2.7 Hz), 111.7, 24.2. ^19^F NMR (CDCl_3_, 376.5 MHz): δ −59.3 (s, 3F). HRMS (ESI-TOF): *m*/*z* [M − H]^+^ Calcd for C_14_H_9_F_3_NOS^+^: 280.0402; found: 280.0404.

*3-(Methoxy(thiophen-2-yl)methyl)-2-(trifluoromethyl)-1H-indole* (**6a**). Grey solid, m.p. 110–112 °C. ^1^H NMR (CDCl_3_, 400.1 MHz): δ 8.43 (br.s, 1H), 7.90 (d, 1H, ^3^*J* = 8.1 Hz), 7.39 (d, 1H, ^3^*J* = 8.3 Hz), 7.32 (t, 1H, ^3^*J* = 7.6 Hz), 7.23 (d, 1H, ^3^*J* = 5.0 Hz), 7.15 (t, 1H, ^3^*J* = 7.5 Hz), 6.86–6.92 (m, 1H), 6.83 (d, 1H, ^4^*J* = 3.4 Hz), 6.03 (s, 1H), 3.41 (s, 3H). ^13^C{^1^H} NMR (CDCl_3_, 100.6 MHz): δ 145.1, 135.3, 126.4, 125.2, 125.1, 125.0, 124.7, 123.0, 122.9 (q, ^2^*J*_CF_ = 37.3 Hz), 121.6 (q, ^1^*J*_CF_ = 269.4 Hz), 121.1, 117.3 (q, ^3^*J*_CF_ = 2.6 Hz), 111.7, 74.3, 56.8. ^19^F NMR (CDCl_3_, 376.5 MHz): δ −58.5 (s, 3F). HRMS (ESI-TOF): *m*/*z* [M + Na]^+^ Calcd for C_15_H_12_F_3_NOS^+^: 334.0484; found: 334.0475.

**Synthesis of 3-(pyridin-4-ylmethyl)-2-(trifluoromethyl)-1H-indole (4s)**. 12 mL vial was charged with enamine **1a** (0.5 mmol), isonicotinaldehyde **2s** (0.0669 g, 0.625 mmol) and glacial acetic acid (1 mL). Reaction mixture was kept at 80–90 °C (hotplate stirrer) under stirring for 10 h. The reaction mixture was cooled down to room temperature. Next, Pd/C (10%, 0.027 g, 0.025 mmol, 5 mol%) and formic acid (0.115 g, 2.5 mmol) was added and the reaction mixture was heated at 75 °C under stirring for 3 h. The reaction mixture was filtered through a short celite pad and dispersed between water (10 mL) and CH_2_Cl_2_ (20 mL). Aqueous layer was separated and extracted with CH_2_Cl_2_ (3×10 mL). Combined organic phases were dried over Na_2_SO_4_, volatiles were evaporated in vacuo, the residue was purified by column chromatography on silica gel, using gradient elution by mixture hexane-CH_2_Cl_2_ (1:1) followed by CH_2_Cl_2_ and CH_2_Cl_2_-MeOH (100:1) as eluents. Pale yellow-brown powder, m.p. 185–187 °C, yield 0.029 g (21%). ^1^H NMR (CDCl_3_, 400.1 MHz): δ 9.03 (br.s, 1H), 8.42–8.50 (m, 2H),7.46 (d, 1H, ^3^*J* = 8.1 Hz), 7.43 (d, 2H, ^3^*J* = 8.3 Hz), 7.32 (t, 1H, ^3^*J* = 7.6 Hz), 7.11–7.18 (m, 3H), 4.26 (s, 2H). ^13^C{^1^H} NMR (CDCl_3_, 100.6 MHz): δ 150.5, 136.8, 127.8, 125.7, 124.4, 123.2 (q, ^1^*J*_CF_ = 268.2 Hz), 123.1 (q, ^2^*J*_CF_ = 36.5 Hz), 121.5, 121.0, 115.1 (q, ^3^*J*_CF_ = 2.7 Hz), 113.1, 29.5. ^19^F NMR (CDCl_3_, 376.5 MHz): δ −59.3 (s, 3F). HRMS (ESI-TOF): *m*/*z* [M + H]^+^ Calcd for C_15_H_12_F_3_N_2_^+^: 277.0947; found: 277.0950. See Supplementary Materials.

## 4. Conclusions

In conclusion, we elaborated a novel two-step pathway towards 2-CF_3_-3-benzylindoles. Based on condensation of α-CF_3_-β-(2-nitroaryl) enamines with benzaldehydes the first step leads effectively to nitro-substituted α,β-diaryl-CF_3_-enones. The second one is a reduction of nitro group by NH_4_HCO_2_-Pd/C system followed by intramolecular cyclization to 2-CF_3_-3-benzylindoles in up to quantitative yields. High selectivity and the reaction yield of all steps are the distinct advantages of the method. Combining the experimental observations and the data of the NMR monitoring of the reaction mixtures, possible scheme of the transformation is evaluated and discussed.

## Figures and Tables

**Table 1:** Reduction of ketone **3a** in various conditions.

Title 1	Reaction Conditions	Yield of 4a, %	Yield of 5a, %	Yield of 6a, %
entry 1	6 eq. Zn, AcOH, 80 °C, 4h	20	47	-
entry 2	12 eq. Zn, AcOH, 80 °C, 14h	43	3	-
entry 3	6 eq. Zn, AcOH-MeOH, 65 °C, 8h	8	2	77
entry 4	H_2_, MeOH, 5 mol% Pd/C, r.t., 1 day	89	-	<1
entry 5	5 eq. NH_4_HCO_2_, MeOH, 5 mol% Pd/C, r.t., 60 °C, 1 h	91	-	<1
entry 6	5 eq. NH_4_HCO_2_, MeOH, 5 mol% Pd/C, r.t., 1 day	99	-	-
entry 7	3.3 equiv. NH_4_HCO_2_, MeOH, 5 mol% Pd/C, 60 °C, 1 h	<1	-	86
entry 8	3.3 equiv. NH_4_HCO_2_, THF, 5 mol% Pd/C, r.t., 1 day; then *p*TSA, MeOH	traces	-	81

## Data Availability

The data presented in this study are available in Supplementary Materials.

## Supplementary Materials

Copy of all ^1^H, ^13^C and ^19^F NMR spectra are available online.

## Sample Availability

Samples of the compounds **3** and **4** are available from the authors.
