# Supplementary material for: An Efficient Synthesis of 2-CF3-3-Benzylindoles
Source: Molecules. 2021 Aug 22;26(16):5084. doi: 10.3390/molecules26165084 (PMC8401147; doi:10.3390/molecules26165084)
Supplement: Supplementary file 1 [file molecules-26-05084-s001.zip › molecules-1330858-supplementary.pdf]

Supporting Information

An efficient synthesis of 2-CF_3-3-benzylindoles

Vasiliy M. Muzalevskiy,[a] Zoia A. Sizova,[a] and Valentine G. Nenajdenko[a,*]

[a] M. V. Lomonosov Moscow State University, Department of Chemistry, Leninskie Gory 1, Moscow, 119991 Russia

Corresponding Author **nenajdenko@org.chem.msu.ru.*

Table of contents

General remarks **S2**

Synthesis of α-CF_3-β-(2-nitroaryl)enamines 2 by the reaction with pyrrolidine in neat (general procedure) **S2**

Synthesis of ketones 3 by the reactions of α-(trifluoromethyl)enamines with aromatic aldehydes (general procedure) **S3**

Reductive cyclization of nitro-ketone 3a to 2-CF_3-indoles in various conditions (a-h) **S4-S6**

Reductive cyclization of nitro-ketones 3 to 2-CF_3-indoles 4 **S7-S14**

NMR data of the reaction intermediates **S15**

Copies of all NMR spectra **S16-S95**

Experimental section

General remarks. ^{1}H, ^{13}C and ^{19}F NMR spectra were recorded on Bruker AVANCE 400 MHz spectrometer in CD_3CN and $CDCl_3$ at 400, 100 and 376 MHz respectively. Chemical shifts (δ) in ppm are reported with the use of the residual CHD_2CN and chloroform signals (1.94 and 7.25 for ^{1}H and 1.30, 77.0 for ^{13}C) as internal reference. The ^{19}F chemical shifts were referenced to C_6F_6, (-162.9 ppm). ESI-MS spectra were measured with an Orbitrap Elite instrument. TLC analysis was performed on "Merck 60 F_{254}" plates. Column chromatography was performed on silica gel. Melting points were determined on an Electrothermal 9100 apparatus. All reagents were of reagent grade and were used as such or were distilled prior to use. Starting α-CF_3-β-aryl enamines **1** were synthesized using previously reported procedures by the reaction with 10 equivalents of pyrrolidine in neat. [1. Muzalevskiy, V. M.; Nenajdenko, V. G.; Rulev, A. Yu.; Ushakov, I. A.; Romanenko, G. V.; Shastin, A. V.; Balenkova, E. S.; Haufe, G. Selective synthesis of α-trifluoromethyl-β-arylenamines or vinylogous guanidinium salts by treatment of β-halo-β-trifluoromethylstyrenes with secondary amines under different conditions. *Tetrahedron* **2009,** *65* (34), 6991 - 7000.]

Synthesis of α-CF_3-β-(2-nitroaryl)enamines 1 by the reaction with pyrrolidine in neat (general procedure). A one neck 25 mL round bottomed flask was charged with dry pyrrolidine (8.5 mL, 100 mmol), cooled down to -18 ℃ and the corresponding styrene (10 mmol) was added in one portion with vigorous stirring. The reaction mixture was stirred at room temperature for 1-3 h until starting styrene was consumed (TLC or NMR monitoring). The excess of pyrrolidine was evaporated in vacuum, the viscous residue was dissolved in CH_2Cl_2 (50 mL), washed with 10% K_2CO_3 solution (2×50 mL) and dried over Na_2SO_4. CH_2Cl_2 was removed in vacuo to give crude enamine, which was used without further purification. For characterization data of enamines **1** see [2] (Muzalevskiy, V. M.; Sizova, Z. A.; Nenajdenko, V. G. Modular Construction of Functionalized 2-CF_3-Indoles. *Org. Lett.*, **2021**, ASAP, DOI: 10.1021/acs.orglett.1c02061).

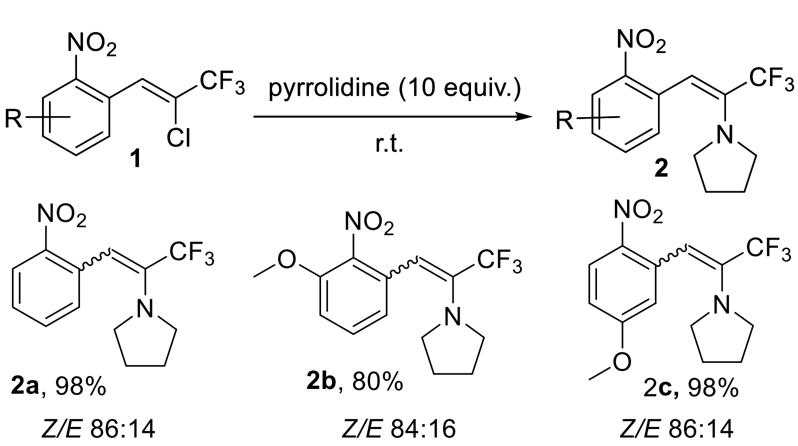

Scheme S1. Synthesis of CF_3-enamines **1**.

Synthesis of ketones 3 by the reactions of α-(trifluoromethyl)enamines with aromatic aldehydes (general procedure). One-necked 50-mL round bottom flask (or 12 mL vial) was charged with enamine **1** (5 mmol), aromatic aldehyde **2** (5.75 mmol) and glacial acetic acid (15 mL or 5 mL for reaction in vial). Reaction mixture was kept at 80-90 ℃ (hotplate stirrer) under stirring for 6-10 hours until consumption of aldehyde and corresponding benzyl ketone formed by the hydrolisys of enamine (^{1}H NMR control). Volatiles were evaporated in vacuo, the residue was dissolved in CH_2Cl_2 (50 mL), washed with water (2×20 mL) and dried over Na_2SO_4. Volatiles were evaporated Please confirm if this should be deleted., the residue was purified by column chromatography, using mixtures of hexane and CH_2Cl_2 (3:1, 1:1), CH_2Cl_2, mixture of CH_2Cl_2 and MeOH (100:1) as eluents. For characterization data of ketones **3** see [2] (Muzalevskiy, V. M.; Sizova, Z. A.; Nenajdenko, V. G. Modular Construction of Functionalized 2‑CF_3‑Indoles. *Org. Lett.*, **2021**, ASAP, DOI: 10.1021/acs.orglett.1c02061).

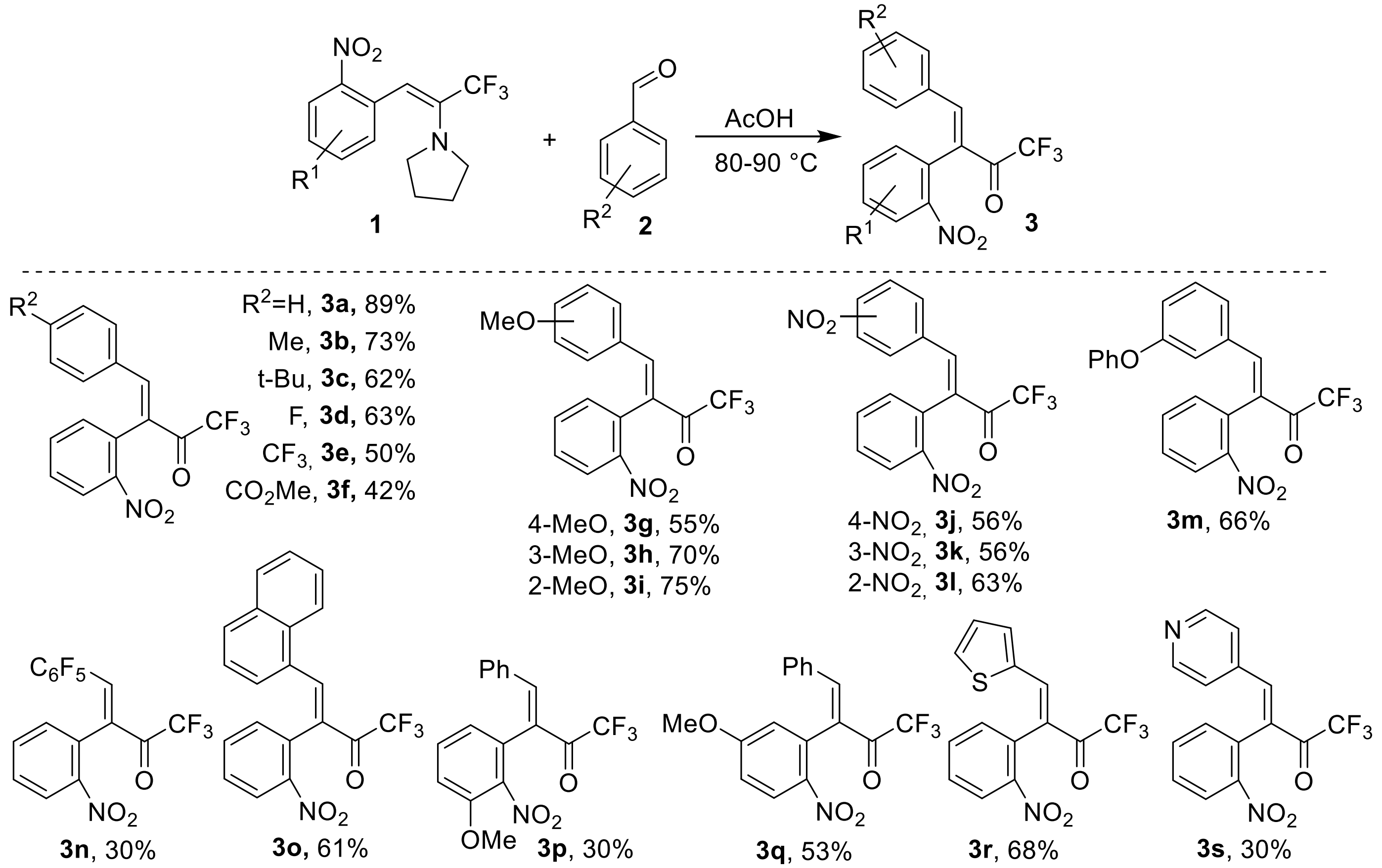

Scheme S2.

Reductive cyclization of nitro-ketone 3a to 2-CF_3-indoles in various conditions (a-h)

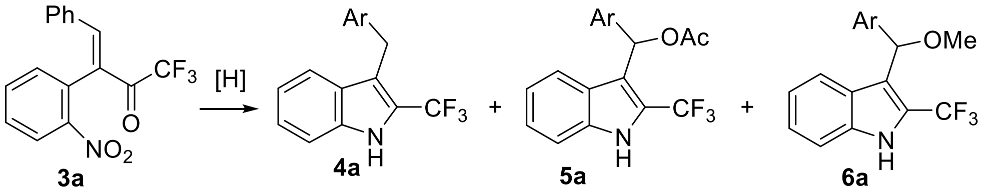

	4a	5a	6a
a, 6 eq. Zn, AcOH, 80 °C, 4h	20%	47%	-
b, 12 eq. Zn, AcOH, 80 °C, 14h	43%	3%	-
c, 6 eq. Zn, AcOH-MeOH, 65 °C, 8h	8%	2%	77%
d, H_2, MeOH, 5 mol% Pd/C, rt, 1 day	89%	-	<1%
e, 5 eq. NH_4HCO_2, MeOH, 5 mol% Pd/C, r.t., 60 °C, 1 h	91%	-	<1%
f, 5 eq. NH_4HCO_2, MeOH, 5 mol% Pd/C, r.t., 1 day	99%	-	-
g, 3.3 equiv. NH_4HCO_2, MeOH, 5 mol% Pd/C, 60 °C, 1 h	<1%	-	86%
h, 3.3 equiv. NH_4HCO_2, THF, 5 mol% Pd/C, r.t., 1 day; then pTSA, MeOH	trases	-	81%

Scheme S3.

Conditions (a,b), Zn in AcOH. 12 mL vial with a screw cap was charged with ketone **3a** (0.0551 g, 0.172 mmol), Zn (0.067 g, 1.03 mmol, 6 equiv.) and AcOH (2 mL). Next, the reaction mixture was kept at 80 ℃ (hotplate stirrer) under stirring for 4 hours.* The reaction mixture was dispersed between water (10-15 mL) and CH_2Cl_2 (10 mL). Aqeous layer was separated and extracted with CH_2Cl_2 (3×10 mL). Combined organic phases were dried over Na_2SO_4, volatiles were evaporated in vacuo, and passed through a short sicagel pad, using mixture of hexane and CH_2Cl_2 (1:1) as an eluent. Evaporation of the solvents gave a mixture of indoles **4a** and **5a**. Conditions a: for the mixture of **4a** and **5a**: brown oil, 0.036 g, yield of **5a** - 47%, yield of **4a** - 20%. For characterization data of **4a** and **5a** see below. Conditions b: for the mixture of **4a** and **5a**: brown oil, 0.025 g, yield of **5a** - 3%, yield of **4a** - 43%.

*-In case of condtions **b** at that point additional amount of Zn (0.067 g, 1.03 mmol, 6 equiv.) was added and the reaction mixture was kept at 80 ℃ (hotplate stirrer) under stirring for another 10 hours

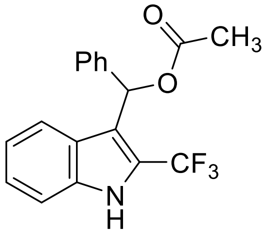

Phenyl(2-(trifluoromethyl)-1*H*-indol-3-yl)methyl acetate (5a). Pale brown solid, m.p. 48-50 ℃. ^{1}H NMR ($CDCl_3$, 400.1 MHz): δ 8.55 (br.s, 1H), 7.70 (d, 1H, ^3J = 8.2 Hz), 7.43 (s, 1H), 7.36-7.42 (m, 3H), 7.24-7.34 (m, 4H), 7.09-7.16 (m, 1H), 2.17 (s, 3H). $^{13}C\{^1H\}$ NMR ($CDCl_3$, 100.6 MHz): δ 170.0, 139.2, 135.2, 128.4, 127.9, 126.5, 125.6, 125.0, 122.4 (q, $^2J_{CF}$ = 37.9 Hz), 122.1, 121.4 (q, $^1J_{CF}$ = 268.9 Hz), 121.3, 116.1 (q, $^3J_{CF}$ = 2.5 Hz), 111.9, 70.0, 21.0. ^{19}F NMR ($CDCl_3$, 376.5 MHz): δ -59.0 (s, 3F). HRMS (ESI-TOF): m/z $[M-Ac]^-$ Calcd for $C_{16}H_{12}F_3NO^-$: 290.0798; found: 290.0797.

Conditions (c), Zn in AcOH-MeOH. 12 mL vial with a screw cap was charged with ketone **3a** (0.0482 g, 0.15 mmol), Zn (0.059 g, 0.9 mmol, 6 equiv.), AcOH (1 mL) and MeOH (1 mL). Next, the reaction mixture was kept at 65 ℃ (hotplate stirrer) under stirring for 8 hours. The reaction mixture was dispersed between water (10-15 mL) and CH_2Cl_2 (10 mL). Aqeous layer was separated and extracted with CH_2Cl_2 (3×10 mL). Combined organic phases were dried over Na_2SO_4, volatiles were evaporated in vacuo, and passed through a short sicagel pad, using mixture of hexane and CH_2Cl_2 (1:1) as an eluent. Evaporation of the solvents gave a mixture of indoles **4a, 5a** and **6a**. For the mixture of **4a, 5a** and **6a**: brown oil, 0.039 g, yield of **5a** - 2%, yield of **4a** - 8%, yield of **6a** - 77%. For characterization data of **4a, 5a** and **6a** see below.

Conditions (d), H_2 in MeOH (on Pd/C). 25 mL three-neck round bottom flask equipped with two glass stop corks, and gas inlet was charged with ketone **3a** (0.095 g, 0.3 mmol), Pd/C (10%, 0.013 g, 0.012 mmol, 5 mol%), methanol (5 mL) and evacuated in vacuo. A balloon with hydrogen was connected to the gas inlet and the flask was filled with hydrogen. The reaction mixture was magnetically stirred for 1 day, the hydrogen balloon was disconnected, the flask was evacuated in vacuo through gas inlet and filled carefully with air. The reaction mixture filtered through celite, volatiles were evaporated in vacuo, the residue was purified by column chromatography, using mixture of hexane and CH_2Cl_2 (3:1) as an eluent. Evaporation of the solvents afforded indole **4a** as a pale brown solid, yield 0.0735 g (89%). For characterization data of **4a** see below.

Conditions (e), NH_4HCO_2 (5 equiv.) in MeOH, Pd/C (10%), 60 ℃**.** 12 mL vial with a screw cap was charged with ketone **3a** (0.108 g, 0.336 mmol), NH_4HCO_2 (0.105 g, 1.67 mmol, 5 equiv.), Pd/C (10%, 0.016 g, 0.015 mmol, 5 mol%) and methanol (1 mL). Next, the reaction mixture was kept at 60 ℃ (hotplate stirrer) under stirring at room temperature for 1 h. After that, 6M HCl (0.3 mL, 1.8 mmol) was added in 4-5 portions (evolution of CO_2!). The reaction mixture was filtred through a short celite pad and dispersed between water (10 mL) and CH_2Cl_2 (20 mL). Aqeous layer was separated and extracted with CH_2Cl_2 (3×10 mL). Combined organic phases were dried over Na_2SO_4, volatiles were evaporated in vacuo, to give pure indole **4a**. Pale brown crystals. For characterization data of **4a** see below.

Conditions (f), NH_4HCO_2 (5 equiv.) in MeOH, Pd/C (10%). 12 mL vial with a screw cap was charged with ketone **3a** (0.055 g, 0.171 mmol), NH_4HCO_2 (0.49 g, 0.78 mmol, 5 equiv.), Pd/C (10%, 0.009 g, 0.085 mmol, 5 mol%) and methanol (1 mL). Next, the reaction mixture was kept under stirring at room temperature for 1 day.* After that, 6M HCl (0.25 mL, 1.5 mmol) was added in 4-5 portions (evolution of CO_2!). The reaction mixture was filtred through a short celite pad and dispersed between water (10 mL) and CH_2Cl_2 (20 mL). Aqeous layer was separated and extracted with CH_2Cl_2 (3×10 mL). Combined organic phases were

dried over Na_2SO_4, volatiles were evaporated in vacuo, to give pure indole **4a**. *-Evaporation of MeOH at this step afforded crude indolinol **D** as a mixture with indole **6** 2:1. Pale brown oil. For NMR data see below.

Conditions (g), NH_4HCO_2 (3.3 equiv.) in MeOH, Pd/C (10%). 12 mL vial with a screw cap was charged with ketone **3a** (0.055 g, 0.171 mmol), NH_4HCO_2 (0.036 g, 0.571 mmol, 3.3 equiv.), Pd/C (10%, 0.009 g, 0.084 mmol, 5 mol%) and methanol (1 mL). Next, the reaction mixture was kept under stirring at room temperature for 1 day. After that, 6M HCl (0.25 mL, 1.5 mmol) was added in 4-5 portions (evolution of CO_2!). The reaction mixture was filtred through a short celite pad and dispersed between water (10 mL) and CH_2Cl_2 (20 mL). Aqeous layer was separated and extracted with CH_2Cl_2 (3×10 mL). Combined organic phases were dried over Na_2SO_4, volatiles were evaporated in vacuo, the residue was purified by column chromatography, using mixture of hexane and CH_2Cl_2 (3:1) as eluent. Evaporation of the solvents afforded indole **6a** as a slightly brown solid, m.p. 86-88 ℃, yield 0.045 g (86%).

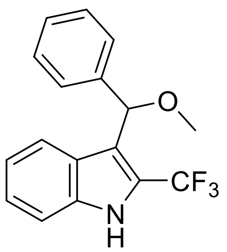

3-(Methoxy(phenyl)methyl)-2-(trifluoromethyl)-1*H*-indole (6a). ^{1}H NMR ($CDCl_3$, 400.1 MHz): δ 8.40 (br.s, 1H), 7.79 (d, 1H, ^3J = 8.2 Hz), 7.49 (d, 2H, ^3J = 7.4 Hz), 7.26-7.39 (m, 4H), 7.20-7.25 (m, 1H), 7.06-7.13 (m, 1H), 5.83 (s, 1H), 3.42 (s, 3H). ^{13}C{^{1}H} NMR ($CDCl_3$, 100.6 MHz): δ 141.3, 135.4, 128.2, 127.2, 126.4, 125.4, 124.9, 123.0 (q, $^2J_{CF}$ = 37.0 Hz), 122.9, 121.8 (q, $^1J_{CF}$ = 269.2 Hz), 121.0, 117.9 (q, $^3J_{CF}$ = 2.6 Hz), 111.6, 77.5, 56.9. ^{19}F NMR ($CDCl_3$, 376.5 MHz): δ -58.2 (s, 3F). HRMS (ESI-TOF): m/z [M-H]$^-$ Calcd for $C_{17}H_{13}F_3NO^-$: 304.0955; found: 304.0945.

Conditions (h) NH_4HCO_2 (3.3 equiv.) in MeOH, Pd/C (10%). 1) Preparation of solution of indolinol B. 20 mL vial with a screw cap was charged with ketone **3a** (0.963 g, 3 mmol), NH_4HCO_2 (0.623 g, 9.9 mmol, 3.3 equiv.), Pd/C (10%, 0.162 g, 0.15 mmol, 5 mol%) and THF (12 mL). Next, the reaction mixture was kept under stirring at room temperature for about 1 day (20 h, ^{19}F NMR control). The reaction mixture was filtred through a short celite pad, celite was washed with THF (3×2 mL) to obtain 9.47 g of solution of **B** (0.1 mmol of B in approximately 0.316 g of the solution). Evaporation of THF led to a crude indolinol **B** as a light yellow-green solid, m.p. 50-55 °C. For NMR data see below. **2) Reaction with MeOH under p-TSA×H_2O catalysis.** 12 mL vial with a screw cap was charged with solution of **B** (0.632 g, ~0.2 mmol), MeOH (0.200 g, 6.25 mmol), stirred for 5 minutes and then *p*-TSA (0.038 g, 0.2 mmol, 1 equiv.) was added. The reaction mixture was stirred overnight, volatiles were evaporated in vacuo, the residue was purified by column chromatography, using mixture of hexane and CH_2Cl_2 (3:1) as an eluent. Evaporation of the solvents afforded indole **6a** as a slightly brown solid, yield 0.049 g (81%).

Reductive cyclization of nitro-ketones 3 to 2-CF_3-indoles 4. 12 mL vial with a screw cap was charged with ketone **4** (0.2 mmol), NH_4HCO_2 (0.063 g, 1.00 mmol, 5 equiv.), Pd/C (10%, 0.0108 g, 0.01 mmol, 5 mol%) and methanol (1.2 mL). Next, the reaction mixture was kept under stirring at 60 °C for 0.5-1 h (conditions A) or at room temperature for 1 day (conditions B). After that, 6M HCl (0.25 mL, 1.5 mmol) was added in 4-5 portions (evolution of CO_2!). The reaction mixture was filtred through a short celite pad and dispersed between water (10 mL) and CH_2Cl_2 (20 mL). Aqeous layer was separated and extracted with CH_2Cl_2 (3×10 mL). Combined organic phases were dried over Na_2SO_4, volatiles were evaporated in vacuo, to give pure indole **4**. In case of indoles **4l**, **4n, 4r**, **4s** additional purification by column chromatography on silica gel was performed. Reduction of ketones **3j-l**, having additional nitro group, was performed using 8 equivalents of NH_4HCO_2 at room temperature (conditions C).

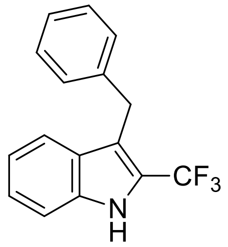

3-Benzyl-2-(trifluoromethyl)-1*H*-indole (4a). Obtained using conditions A (0.108 g, 0.34 mmol of **3a**) or conditions B (0.055 g, 0.171 mmol of **3a**). Pale brown crystals, m.p. 103-104 ℃, yield 0.084 g (91%, A) 0.0465 g (99%, B). ^{1}H NMR ($CDCl_3$, 400.1 MHz): δ 8.25 (br.s, 1H), 7.57 (d, 1H, ^3J = 8.1 Hz), 7.39 (d, 1H, ^3J = 8.2 Hz), 7.27-7.37 (m, 5H), 7.20-7.26 (m, 1H), 7.13-7.19 (m, 1H), 4.32 (s, 2H). ^{13}C{^{1}H} NMR ($CDCl_3$, 100.6 MHz): δ 139.9, 135.3, 128.4, 128.3, 127.4, 126.1, 124.8, 122.03 (q, $^2J_{CF}$ = 36.5 Hz), 122.01 (q, $^1J_{CF}$ = 269.0 Hz), 120.8, 120.7, 116.8 (q, $^3J_{CF}$ = 2.8 Hz), 111.7, 29.8. ^{19}F NMR ($CDCl_3$, 376.5 MHz): δ -59.1 (s, 3F). NMR data are in agreement with those in the literature [3. Cheng, Y., Yuan, X., Ma, J. and Yu, S. Direct Aromatic C-H Trifluoromethylation via an Electron-Donor–Acceptor Complex. *Chem. Eur. J.*, **2015**, *21*, 8355-8359.]

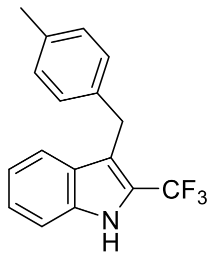

3-(4-Methylbenzyl)-2-(trifluoromethyl)-1H-indole (4b). Obtained using conditions A (0.109 g, 0.325 mmol of **3b**). Pale brown solid, m.p. 88-90 ℃, yield 0.090 g (96%). ^{1}H NMR ($CDCl_3$, 400.1 MHz): δ 8.24 (br.s, 1H), 7.63 (d, 1H, ^3J = 8.1 Hz), 7.35-7.44 (m, 2H), 7.25 (d, 2H, ^3J = 8.0 Hz), 7.19-7.23 (m, 1H), 7.17 (d, 2H, ^3J = 7.9 Hz), 4.33 (s, 2H), 2.39 (s, 3H). ^{13}C{^{1}H} NMR ($CDCl_3$, 100.6 MHz): δ 136.9, 135.6, 135.3, 129.1, 128.1, 127.4, 124.7, 122.1(q, $^1J_{CF}$ = 269.0 Hz), 121.9 (q, $^2J_{CF}$ = 36.5 Hz), 120.8, 120.6, 117.0 (q, $^3J_{CF}$ = 2.8 Hz), 111.6, 29.3, 20.9. ^{19}F NMR ($CDCl_3$, 376.5 MHz): δ -59.1 (s, 3F). HRMS (ESI-TOF): m/z [M-H]$^-$ Calcd for $C_{17}H_{13}F_3N^-$: 288.1006; found: 288.1009.

C17H14F3N

3-(4-(*tert*-Butyl)benzyl)-2-(trifluoromethyl)-1H-indole (4c). Obtained using conditions B (0.120 g, 0.318 mmol of **3c**). Pale brown solid, m.p. 85-87 ℃, yield 0.100 g (95%). ^{1}H NMR ($CDCl_3$, 400.1 MHz): δ 8.23 (br.s, 1H), 7.64 (d, 1H, ^{3}J = 8.1 Hz), 7.33-7.42 (m, 4H), 7.25-7.32 (m, 2H), 7.19 (ddd, 1H, ^{3}J = 8.0 Hz, ^{3}J = 6.6 Hz, ^{4}J = 1.4 Hz), 4.32 (s, 2H), 1.36 (s, 9H). ^{13}C{^{1}H} NMR ($CDCl_3$, 100.6 MHz): δ 148.9, 136.9, 135.3, 127.9, 127.5, 125.3, 124.8, 122.0 (q, $^{1}J_{CF}$ = 269.1 Hz), 121.9 (q, $^{2}J_{CF}$ = 36.7 Hz), 120.9, 120.6, 117.1 (q, $^{3}J_{CF}$ = 2.6 Hz), 111.6, 34.3, 31.3, 29.2. ^{19}F NMR ($CDCl_3$, 376.5 MHz): δ -59.0 (s, 3F). HRMS (ESI-TOF): m/z [M-H]$^-$ Calcd for $C_{20}H_{19}F_3N^-$: 330.1475; found: 330.1472.

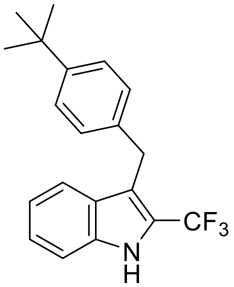

3-(4-Fluorobenzyl)-2-(trifluoromethyl)-1H-indole (4d). Obtained using conditions A (0.126 g, 0.372 mmol of **3d**). Brown viscous oil, yield 0.105 g (96%). ^{1}H NMR ($CDCl_3$, 400.1 MHz): δ 8.30 (br.s, 1H), 7.56 (d, 1H, ^{3}J = 8.1 Hz), 7.33-7.45 (m, 2H), 7.16-7.28 (m, 3H), 6.94-7.05 (m, 2H), 4.29 (s, 2H). ^{13}C{^{1}H} NMR ($CDCl_3$, 100.6 MHz): δ 161.3 (d, $^{1}J_{CF}$ = 243.8 Hz), 135.6 (d, $^{4}J_{CF}$ = 2.8 Hz), 135.3, 129.6 (d, $^{3}J_{CF}$ = 7.9 Hz), 127.3, 124.9, 122.0 (q, $^{2}J_{CF}$ = 36.4 Hz), 121.9 (q, $^{1}J_{CF}$ = 269.0 Hz), 120.8, 120.6, 116.6 (q, $^{3}J_{CF}$ = 2.6 Hz), 115.1 (d, $^{2}J_{CF}$ = 21.3 Hz), 111.7, 28.9. ^{19}F NMR ($CDCl_3$, 376.5 MHz): δ -59.1 (s, 3F), -118.19 - -118.55 (m, 1F). HRMS (ESI-TOF): m/z [M-H]$^-$ Calcd for $C_{16}H_{10}F_4N^-$: 292.0755; found: 292.0749.

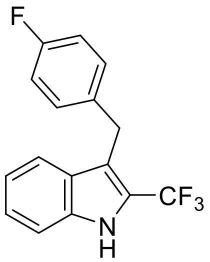

2-(Trifluoromethyl)-3-(4-(trifluoromethyl)benzyl)-1H-indole (4e). Obtained using conditions B (0.147 g, 0.378 mmol of **3e**). Pale brown solid, m.p. 54-56 ℃, yield 0.129 g (>99%). ^{1}H NMR ($CDCl_3$, 400.1 MHz): δ 8.36 (br.s, 1H), 7.50-7.66 (m, 3H), 7.40-7.49(m, 1H), 7.39-7.40 (m, 3H), 7.19 (t, 1H, ^{3}J = 7.5 Hz),4.35 (s, 2H). ^{13}C{^{1}H} NMR ($CDCl_3$, 100.6 MHz): δ 144.0, 135.3, 128.49 (q, $^{2}J_{CF}$ = 32.3 Hz), 128.51, 127.2, 125.4 (q, $^{3}J_{CF}$ = 3.7 Hz), 124.3 (q, $^{1}J_{CF}$ = 271.9 Hz), 122.4 (q, $^{2}J_{CF}$ = 36.8 Hz), 121.9 (q, $^{1}J_{CF}$ = 269.1 Hz), 121.0, 120.4, 115.6 (q, $^{3}J_{CF}$ = 2.6 Hz), 111.9, 29.5. ^{19}F NMR ($CDCl_3$, 376.5 MHz): δ -59.1 (s, 3F), -63.4 (s, 3F). HRMS (ESI-TOF): m/z [M-H]$^-$ Calcd for $C_{17}H_{10}F_6N^-$: 342.0723; found: 342.0714.

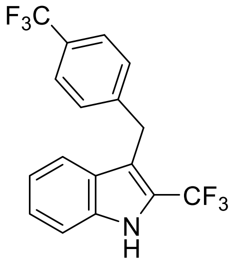

Methyl 4-((2-(trifluoromethyl)-1H-indol-3-yl)methyl)benzoate (4f). Obtained using conditions B (0.085 g, 0.224 mmol of **3f**). Pale yellow solid, m.p. 109-111 ℃, yield 0.074 g (>99%). ^{1}H NMR ($CDCl_3$, 400.1 MHz): δ 8.69 (br.s, 1H), 7.95 (d, 2H, ^{3}J = 8.3 Hz), 7.48 (d, 2H, ^{3}J = 8.1 Hz), 7.41 (d, 1H, ^{3}J = 8.3 Hz), 7.27-7.35 (m, 3H), 7.10-7.16 (m, 1H), 4.32 (s, 2H), 3.90 (s, 3H). ^{13}C{^{1}H} NMR ($CDCl_3$, 100.6 MHz): δ 167.2, 145.5, 135.4, 129.8, 128.3, 128.0, 127.2, 124.9, 122.3 (q, $^{2}J_{CF}$ = 36.8 Hz), 121.9 (q,

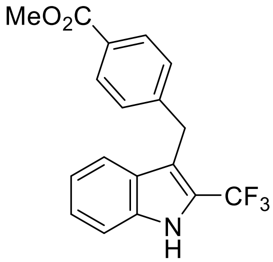

$^{1}J_{CF}$ = 269.0 Hz), 120.8, 120.4, 115.5 (q, $^{3}J_{CF}$ = 2.9 Hz), 111.8, 52.0, 29.8. ^{19}F NMR ($CDCl_3$, 376.5 MHz): δ -59.1 (s, 3F). HRMS (ESI-TOF): m/z [M-H]$^-$ Calcd for $C_{18}H_{13}F_3NO_2^-$: 332.0904; found: 332.0904.

3-(4-Methoxybenzyl)-2-(trifluoromethyl)-1H-indole (4g). Obtained using conditions B (0.057 g, 0.161 mmol of **3g**). White powder, m.p. 116-118 ℃, yield 0.047 g (95%). ^{1}H NMR ($CDCl_3$, 400.1 MHz): δ 8.34 (br.s, 1H), 7.56 (d, 1H, ^{3}J = 8.1 Hz), 7.37 (d, 1H, ^{3}J = 8.2 Hz), 7.32 (t, 1H, ^{3}J = 7.5 Hz), 7.20 (d, 2H, ^{3}J = 8.5 Hz), 7.11-7.17 (m, 1H), 6.84 (d, 2H, ^{3}J = 8.6 Hz), 4.24 (s, 2H), 3.79 (s, 3H). ^{13}C{^{1}H} NMR ($CDCl_3$, 100.6 MHz): δ 157.8, 135.3, 132.1, 129.2, 127.4, 124.8, 122.0 (q, $^{1}J_{CF}$ = 268.9 Hz), 121.9 (q, $^{2}J_{CF}$ = 36.7 Hz), 120.8, 120.6, 117.2 (q, $^{3}J_{CF}$ = 2.8 Hz), 113.8, 111.7, 55.2, 28.9. ^{19}F NMR ($CDCl_3$, 376.5 MHz): δ -59.1 (s, 3F). HRMS (ESI-TOF): m/z [M-H]$^-$ Calcd for $C_{17}H_{13}F_3NO^-$: 304.0955; found: 304.0945.

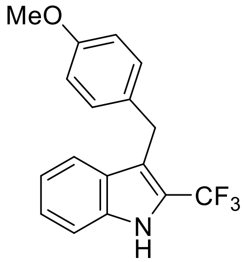

3-(3-Methoxybenzyl)-2-(trifluoromethyl)-1H-indole (4h). Obtained using conditions B (0.104 g, 0.296 mmol of **3h**). Pale brown solid, m.p. 55-57 ℃, yield 0.080 g (89%). ^{1}H NMR ($CDCl_3$, 400.1 MHz): δ 8.35 (br.s, 1H), 7.57 (d, 1H, ^{3}J = 8.1 Hz), 7.28-7.38 (m, 2H), 7.21 (d, 1H, ^{3}J = 7.9 Hz), 7.12-7.17 (m, 1H), 6.90 (d, 1H, ^{3}J = 7.7 Hz), 6.85 (*pseudo*-s, 1H), 6.77 (dd, 1H, ^{3}J = 8.2 Hz, ^{4}J = 2.3 Hz), 4.28 (s, 2H), 3.77 (s, 3H). ^{13}C{^{1}H} NMR ($CDCl_3$, 100.6 MHz): δ 159.6, 141.6, 135.3, 129.3, 127.4, 124.8, 122.01 (q, $^{2}J_{CF}$ = 36.5 Hz), 121.99 (q, $^{1}J_{CF}$ = 269.1 Hz), 120.8, 120.70, 120.65, 116.5 (q, $^{3}J_{CF}$ = 2.9 Hz), 114.3, 111.7, 111.2, 55.0, 29.7. ^{19}F NMR ($CDCl_3$, 376.5 MHz): δ -59.0 (s, 3F). HRMS (ESI-TOF): m/z [M-H]$^-$ Calcd for $C_{17}H_{13}F_3NO^-$: 304.0955; found: 304.0953.

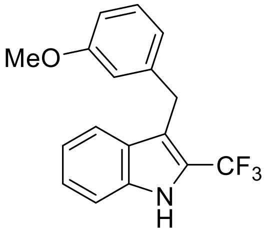

3-(2-Methoxybenzyl)-2-(trifluoromethyl)-1H-indole (4i). Obtained using conditions B (0.116 g, 0.330 mmol of **3i**). Pale yellow solid, m.p. 67-69 ℃, yield 0.099 g (98%). ^{1}H NMR ($CDCl_3$, 400.1 MHz): δ 8.28 (br.s, 1H), 7.58 (d, 1H, ^{3}J = 8.1 Hz), 7.31-7.41 (m, 2H), 7.20-7.25 (m, 1H), 7.15 (ddd, 1H, ^{3}J = 8.0 Hz, ^{3}J = 6.8 Hz, ^{4}J = 1.2 Hz), 6.96-7.02 (m, 1H), 6.94 (dd, 1H, ^{3}J = 8.2 Hz, ^{4}J = 0.7 Hz), 6.85 (td, 1H, ^{3}J = 7.5 Hz, ^{4}J = 1.0 Hz), 4.34 (s, 2H), 3.94 (s, 3H). ^{13}C{^{1}H} NMR ($CDCl_3$, 100.6 MHz): δ 157.0, 135.3, 129.2, 128.2, 127.7, 127.2, 124.7 122.3 (q, $^{2}J_{CF}$ = 36.7 Hz), 122.1 (q, $^{1}J_{CF}$ = 269.0 Hz), 121.0, 120.5, 120.4, 116.5 (q, $^{3}J_{CF}$ = 2.7 Hz), 111.5, 109.9, 55.2, 23.3. ^{19}F NMR ($CDCl_3$, 376.5 MHz): δ -59.3 (s, 3F). HRMS (ESI-TOF): m/z [M-H]$^-$ Calcd for $C_{17}H_{13}F_3NO^-$: 304.0955; found: 304.0951.

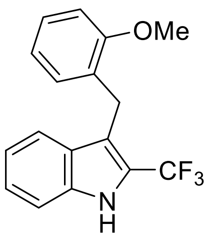

4-((2-(Trifluoromethyl)-1H-indol-3-yl)methyl)aniline (4j). Obtained using conditions C (0.112 g, 0.306 mmol of **3j**). Pale brown solid, m.p. 175-177 °C, yield 0.087 g (98%). ^{1}H NMR ($CDCl_3$, 400.1 MHz): δ 9.87 (br.s, 1H), 7.55 (d, 1H, ^3J = 8.1 Hz), 7.46 (d, 1H, ^3J = 8.3 Hz), 7.28 (t, 1H, ^3J = 7.6 Hz), 7.09 (ddd, 1H, ^3J = 8.0 Hz, ^3J = 7.1 Hz, ^4J = 0.9 Hz), 6.95 (d, 2H, ^3J = 8.4 Hz), 6.54 (d, 2H, ^3J = 8.5 Hz), 4.11 (s, 2H), 3.98 (br.s, 2H). ^{13}C{^{1}H} NMR ($CDCl_3$, 100.6 MHz): δ 146.9, 136.8, 130.0, 129.7, 127.9, 125.4, 123.4 (q, $^1J_{CF}$ = 268.1 Hz), 122.1 (q, $^2J_{CF}$ = 36.6 Hz), 121.4, 121.0, 115.4, 112.9, 29.2. ^{19}F NMR ($CDCl_3$, 376.5 MHz): δ -56.8 (s, 3F). HRMS (ESI-TOF): m/z $[M+H]^+$ Calcd for $C_{16}H_{14}F_3N_2^+$: 291.1104; found: 291.1110.

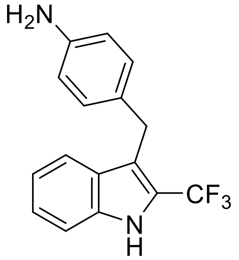

3-((2-(Trifluoromethyl)-1H-indol-3-yl)methyl)aniline (4k). Obtained using conditions C (0.120 g, 0.328 mmol of **3k**). Pale yellow solid, m.p. 138-140 °C, yield 0.086 g (90%). ^{1}H NMR ($CDCl_3$, 400.1 MHz): δ 8.56 (br.s, 1H), 7.56 (d, 1H, ^3J = 8.1 Hz), 7.26-7.35 (m, 2H), 7.05-7.15 (m, 2H), 6.72 (d, 2H, ^3J = 7.6 Hz), 6.57 (br.s, 1H), 6.53 (dd, 1H, ^3J = 7.9 Hz, ^3J = 1.7 Hz), 4.20 (s, 2H), 3.54 (br.s, 2H). ^{13}C{^{1}H} NMR ($CDCl_3$, 100.6 MHz): δ 146.2, 141.3, 135.3, 129.2, 127.5, 124.7, 121.97 (q, $^2J_{CF}$ = 36.5 Hz), 122.02 (q, $^1J_{CF}$ = 268.7 Hz), 120.8, 120.5,118.9, 116.6 (q, $^3J_{CF}$ = 2.4 Hz), 115.2, 113.2, 111.6, 29.65. ^{19}F NMR ($CDCl_3$, 376.5 MHz): δ -58.9 (s, 3F). HRMS (ESI-TOF): m/z $[M+H]^+$ Calcd for $C_{16}H_{14}F_3N_2^+$: 291.1104; found: 291.1111.

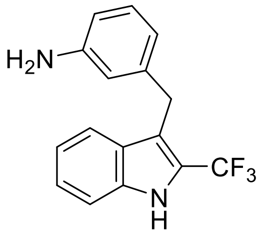

2-((2-(Trifluoromethyl)-1H-indol-3-yl)methyl)aniline (4l). Obtained using conditions C (0.160 g, 0.437 mmol of **3l**). Purified by column chromatography, using gradient elution by CH_2Cl_2 followed by mixture CH_2Cl_2-MeOH (100:1, 30:1). Pale yellow solid, m.p. 136-138 °C, yield 0.097 g (76%). ^{1}H NMR ($CDCl_3$, 400.1 MHz): δ 8.50 (br.s, 1H), 7.40 (d, 1H, ^3J = 8.1 Hz), 7.35 (d, 1H, ^3J = 8.2 Hz), 7.29 (t, 1H, ^3J = 7.5 Hz), 7.08-7.12 (m, 1H), 7.04-7.08 (m, 1H), 6.93 (d, 1H, ^3J = 7.5 Hz), 6.67-6.75 (m, 2H), 4.13 (s, 2H), 3.53 (br.s, 1H). ^{13}C{^{1}H} NMR ($CDCl_3$, 100.6 MHz): δ 144.2, 135.3, 129.5, 127.5, 127.4, 124.9, 123.9, 122.4 (q, $^2J_{CF}$ = 36.8 Hz), 121.9 (q, $^1J_{CF}$ = 269.0 Hz), 120.9, 120.7, 118.8, 115.7, 115.0 (q, $^3J_{CF}$ = 2.9 Hz), 111.7, 25.9. ^{19}F NMR ($CDCl_3$, 376.5 MHz): δ -59.3 (s, 3F). HRMS (ESI-TOF): m/z $[M+H]^+$ Calcd for $C_{16}H_{14}F_3N_2^+$: 291.1104; found: 291.1104.

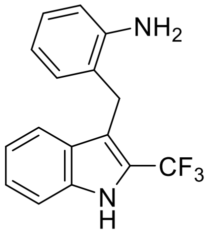

3-(3-Phenoxybenzyl)-2-(trifluoromethyl)-1H-indole (4m). Obtained using conditions B (0.126 g, 0.305 mmol of **3m**). Pale yellow solid, m.p. 71-73 °C, yield

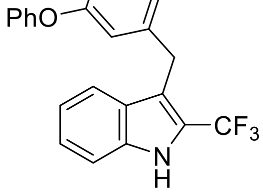

0.107 g (96%). ^{1}H NMR ($CDCl_3$, 400.1 MHz): δ 8.33 (br.s, 1H), 7.56 (d, 1H, ^3J = 8.1 Hz), 7.31-7.41 (m, 4H), 7.22-7.27 (m, 1H), 7.10-7.20 (m, 2H), 6.97-7.07 (m, 4H), 6.86 (dd, 1H, ^3J = 8.1 Hz, ^4J = 1.7 Hz), 4.29 (s, 2H). ^{13}C{^{1}H} NMR ($CDCl_3$, 100.6 MHz): δ 157.14, 157.11, 142.1, 135.3, 129.6, 127.3, 124.8, 123.3, 123.1, 122.1 (q, $^2J_{CF}$ = 36.7 Hz), 121.9 (q, $^1J_{CF}$ = 269.0 Hz), 120.69, 120.65, 119.1, 118.7, 116.5, 116.3 (q, $^3J_{CF}$ = 2.5 Hz), 111.7, 29.6. ^{19}F NMR ($CDCl_3$, 376.5 MHz): δ -59.1 (s, 3F). HRMS (ESI-TOF): m/z $[M-H]^-$ Calcd for $C_{22}H_{15}F_3NO^-$: 366.1111; found: 366.1107.

3-((Perfluorophenyl)methyl)-2-(trifluoromethyl)-1H-indole (4n). Obtained using conditions B (0.117 g, 0.285 mmol of **3n**). Purified by column chromatography, using gradient elution by mixture hexane-CH_2Cl_2 (4:1) followed by mixture hexane-CH_2Cl_2 (2:1). Pale brown solid, m.p. 131-133 °C, yield 0.082 g (79%). ^{1}H NMR ($CDCl_3$, 400.1 MHz): δ 8.32 (br.s, 1H), 7.56 (d, 1H, ^3J = 8.1 Hz), 7.37-7.42 (m, 1H), 7.29-7.36 (m, 1H), 7.19 (ddd, 1H, ^3J = 8.1 Hz, ^3J = 6.9 Hz, ^4J = 1.1 Hz), 4.32 (s, 2H). ^{13}C{^{1}H} NMR ($CDCl_3$, 100.6 MHz): δ 145.3 (dddt, $^1J_{CF}$ = 246.8 Hz, $^3J_{CF}$ = 11.8 Hz, $^4J_{CF}$ = 7.8 Hz, $^5J_{CF}$ = 3.8 Hz, CF), 140.04 (dm, $^1J_{CF}$ = 258.6 Hz, m_1 141.5-141.1, m_2 138.9-138.6, CF), 137.5 (dm, $^1J_{CF}$ = 257.8 Hz, m_1 138.9-138.6, m_2 136.5-136.1, CF), 135.0, 126.6, 125.1, 122.3 (q, $^2J_{CF}$ = 37.4 Hz), 121.7 (q, $^1J_{CF}$ = 269.1 Hz), 121.1, 119.7, 113.1, 112.9, 111.9, 29.8 (d, $^3J_{CF}$ = 20.6 Hz, CF). ^{19}F NMR ($CDCl_3$, 376.5 MHz): δ -59.7 (s, 3F), -142.98 - -143.23 (m, 2F), -157.9 (t, 1F, J = 20.8 Hz), -163.47 - -163.67 (m, 2F). HRMS (ESI-TOF): m/z $[M-H]^-$ Calcd for $C_{16}H_6F_8N^-$: 364.0378; found: 364.0373.

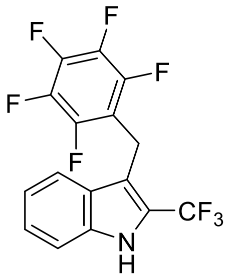

C16H7F8N

3-(Naphthalen-1-ylmethyl)-2-(trifluoromethyl)-1H-indole (4o). Obtained using conditions B (0.043 g, 0.116 mmol of **3o**) and 8 equivalents of NH_4HCO_2 (0.059 g, 0.94 mmol, 8 equiv.). White solid, m.p. 69-71 °C, yield 0.0328 g (87%). ^{1}H NMR ($CDCl_3$, 400.1 MHz): δ 8.37 (br.s, 1H), 8.29 (d, 1H, ^3J = 8.4 Hz), 7.90-7.98 (m, 1H), 7.76 (d, 1H, ^3J = 8.2 Hz), 7.59-7.66 (m, 1H), 7.53-7.59(m, 1H), 7.43 (d, 1H, ^3J = 8.3 Hz), 07.29-7.39 (m, 3H), 7.08 (ddd, 1H, ^3J = 8.0 Hz, ^3J = 7.1 Hz, ^4J = 0.9 Hz), 7.04 (dd, 1H, ^3J = 7.1 Hz, ^4J = 0.9 Hz), 4.78 (s, 2H). ^{13}C{^{1}H} NMR ($CDCl_3$, 100.6 MHz): δ 135.4, 135.3, 133.6, 131.9, 128.8, 127.7, 126.9, 126.1, 125.6, 125.5, 125.4, 124.9, 123.2, 122.7 (q, $^2J_{CF}$ = 36.7 Hz), 122.0 (q, $^1J_{CF}$ = 269.3 Hz), 120.9, 120.7, 115.7 (q, $^3J_{CF}$ = 2.9 Hz), 111.7, 26.6. ^{19}F NMR ($CDCl_3$, 376.5 MHz): δ -59.7 (s, 3F). HRMS (ESI-TOF): m/z $[M-H]^-$ Calcd for $C_{20}H_{13}F_3N^-$: 324.1006; found: 324.1002.

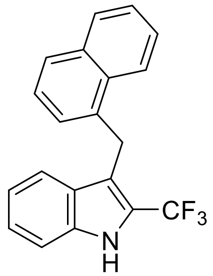

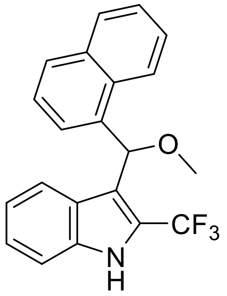

3-(Methoxy(naphthalen-1-yl)methyl)-2-(trifluoromethyl)-1*H*-indole (6b). Obtained using conditions B (0.153 g, 0.412 mmol of **3o**) as a mixture with indole **4o** **(**yield 0.069 g (51%) for **4o**). Purified by column chromatography, using mixture of hexane and CH_2Cl_2 (1:1) as an eluent. Yellow powder, m.p. 65-67 ℃, yield 0.041 g (28%). ^{1}H NMR ($CDCl_3$, 400.1 MHz): δ 8.53 (br.s, 1H), 8.17 (d, 1H, ^3J = 8.5 Hz), 7.88 (dd, 1H, ^3J = 8.3 Hz, ^4J = 0.9 Hz), 7.81 (d, 1H, ^3J = 8.0 Hz), 7.74 (d, 1H, ^3J = 8.2 Hz), 7.53-7.60 (m, 1H), 7.47-7.53 (m, 1H), 7.40 (d, 2H, ^3J = 8.2 Hz), 7.36 (d, 1H, ^3J = 7.9 Hz), 7.28-7.34 (m, 1H), 7.06-7.13 (m, 1H), 6.51 (s, 1H), 3.54 (s, 3H). ^{13}C{^{1}H} NMR ($CDCl_3$, 100.6 MHz): δ 135.6, 135.3, 134.0, 131.6, 128.9, 128.7, 126.5, 126.3, 125.6, 125.3, 124.98, 124.94, 123.8, 123.3 (q, $^2J_{CF}$ = 37.4 Hz), 123.0, 121.7 (q, $^1J_{CF}$ = 269.5 Hz), 121.2, 116.5 (q, $^3J_{CF}$ = 2.6 Hz), 111.7, 75.5, 57.2. ^{19}F NMR ($CDCl_3$, 376.5 MHz): δ -59.0 (s, 3F). HRMS (ESI-TOF): m/z [M-OMe]$^-$ Calcd for $C_{20}H_{14}F_3N^-$: 324.1002; found: 324.1006.

3-Benzyl-7-methoxy-2-(trifluoromethyl)-1H-indole (4p). Obtained using conditions B (0.053 g, 0.151 mmol of **3p**). Green-yellowish viscous oil, yield 0.044 g (96%). ^{1}H NMR ($CDCl_3$, 400.1 MHz): δ 8.57 (br.s, 1H), 7.23-7.30 (m, 4H), 7.15-7.22 (m,1H), 7.11 (d, 1H, ^3J = 8.1 Hz), 7.04 (t, 1H, ^3J = 7.6 Hz), 6.72 (d, 1H, ^3J = 7.6 Hz), 4.26 (s, 2H), 3.97 (s, 3H). ^{13}C{^{1}H} NMR ($CDCl_3$, 100.6 MHz): δ 146.3, 140.0, 128.7, 128.4, 128.3, 126.3, 126.0, 122.0 (q, $^1J_{CF}$ = 268.9 Hz), 121.8 (q, $^2J_{CF}$ = 36.8 Hz), 121.2, 117.1 (q, $^3J_{CF}$ = 2.8 Hz), 113.1, 103.9, 55.4, 30.0. ^{19}F NMR ($CDCl_3$, 376.5 MHz): δ -59.1 (s, 3F). HRMS (ESI-TOF): m/z [M-H]$^-$ Calcd for $C_{17}H_{13}F_3NO^-$: 304.0955; found: 304.0960.

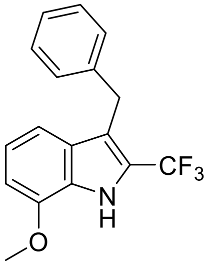

3-Benzyl-5-methoxy-2-(trifluoromethyl)-1H-indole (4q). Obtained using conditions B (0.098 g, 0.279 mmol of **3q**). Pale brown solid, m.p. 102-104 ℃, yield 0.0826 g (97%). ^{1}H NMR ($CDCl_3$, 400.1 MHz): δ 8.29 (br.s, 1H), 7.24-7.33 (m, 5H), 7.19-7.24 (m,1H), 6.99 (dd, 1H, ^3J = 8.9 Hz, ^4J = 2.4 Hz), 6.92 (d, 1H, ^4J = 2.4 Hz), 4.27 (s, 2H), 3.78 (s,3H). ^{13}C{^{1}H} NMR ($CDCl_3$, 100.6 MHz): δ 154.5, 139.9, 130.5, 128.4, 128.3, 127.9, 126.1, 122.6 (q, $^2J_{CF}$ = 36.7 Hz), 121.9 (q, $^1J_{CF}$ = 269.0 Hz), 116.2 (q, $^3J_{CF}$ = 2.4 Hz), 115.6, 112.6, 101.6, 55.7, 29.8. ^{19}F NMR ($CDCl_3$, 376.5 MHz): δ -59.2 (s, 3F). HRMS (ESI-TOF): m/z [M-H]$^-$ Calcd for $C_{17}H_{13}F_3NO^-$: 304.0955; found: 304.0946.

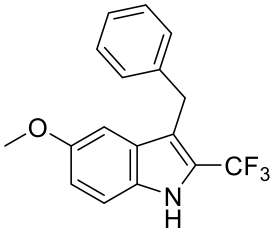

Reduction of ketone 3r. Using conditions A: 12 mL vial with a screw cap was charged with ketone **3r** (0.072 g, 0.220 mmol), NH_4HCO_2 (0.069 g, 1.10 mmol, 5 equiv.), Pd/C (10%, 0.012 g, 0.011 mmol, 5 mol%) and methanol (1.5 mL). Next, the reaction mixture was kept under stirring at 60 °C for 1 h. After that,

6M HCl (0.25 mL, 1.5 mmol) was added in 4-5 portions (evolution of CO_2!). The reaction mixture was filtred through a short celite pad and dispersed between water (10 mL) and CH_2Cl_2 (20 mL). Aqeous layer was separated and extracted with CH_2Cl_2 (3×10 mL). Combined organic phases were dried over Na_2SO_4, volatiles were evaporated in vacuo, the residue was purified by column chromatography on silica gel, using gradient elution by mixture hexane-CH_2Cl_2 (4:1) followed by mixture hexane-CH_2Cl_2 (2:1) to give 3-(thiophen-2-ylmethyl)-2-(trifluoromethyl)-1H-indole **(4r)**, yield 0.0048 g, (8%) and 3-(methoxy(thiophen-2-yl)methyl)-2-(trifluoromethyl)-1H-indole **(6c)**, yield 0.035 g, (51%).

Using conditions D: 12 mL vial with a screw cap was charged with ketone **3r** (0.068 g, 0.208 mmol), NH_4HCO_2 (0.188 g, 2.98 mmol, ~15 equiv.), Pd/C (10%, 0.011 g, 0.0104 mmol, 5 mol%) and methanol (3 mL). Next, the reaction mixture was kept under stirring for 1 day. After that, 6M HCl (0.5 mL, 3 mmol) was added in 4-5 portions (evolution of CO_2!). The reaction mixture was filtred through a short celite pad and dispersed between water (10 mL) and CH_2Cl_2 (20 mL). Aqeous layer was separated and extracted with CH_2Cl_2 (3×10 mL). Combined organic phases were dried over Na_2SO_4, volatiles were evaporated in vacuo, the residue was purified by column chromatography on silica gel, using gradient elution by mixture hexane-CH_2Cl_2 (4:1) followed by mixture hexane-CH_2Cl_2 (2:1) to give 3-(thiophen-2-ylmethyl)-2-(trifluoromethyl)-1H-indole **(4r)**, yield 0.031 g, (53%) and 3-(methoxy(thiophen-2-yl)methyl)-2-(trifluoromethyl)-1H-indole **(6c)**, yield 0.0038 g, (6%).

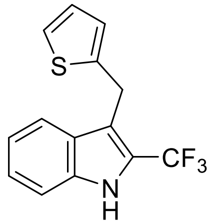

3-(Thiophen-2-ylmethyl)-2-(trifluoromethyl)-1H-indole (4r). White powder, m.p. 88-90 ℃. ^{1}H NMR ($CDCl_3$, 400.1 MHz): δ 8.26 (br.s, 1H), 7.63 (d, 1H, ^3J = 8.1 Hz), 7.37-7.42 (m, 1H), 7.29-7.35 (m, 1H), 7.17 (ddd, 1H, ^3J = 8.0 Hz, ^3J = 7.0 Hz, ^4J = 1.0 Hz), 7.10 (dd, 1H, ^3J = 5.1 Hz, ^4J = 1.2 Hz), 6.89 (dd, 1H, ^3J = 5.1 Hz, ^4J = 3.5 Hz), 6.80-6.86 (m,1H), 4.44 (s, 2H). ^{13}C{^{1}H} NMR ($CDCl_3$, 100.6 MHz): δ 143.0, 135.2, 127.0, 126.7, 125.0, 124.8, 123.6, 121.8 (q, $^1J_{CF}$ = 269.0 Hz), 121.7 (q, $^2J_{CF}$ = 36.8 Hz), 120.8, 120.5, 116.4 (q, $^3J_{CF}$ = 2.7 Hz), 111.7, 24.2. ^{19}F NMR ($CDCl_3$, 376.5 MHz): δ -59.3 (s, 3F). HRMS (ESI-TOF): m/z $[M-H]^+$ Calcd for $C_{14}H_9F_3NOS^+$: 280.0402; found: 280.0404.

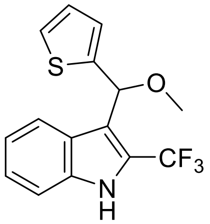

3-(Methoxy(thiophen-2-yl)methyl)-2-(trifluoromethyl)-1H-indole (6c). Grey solid, m.p. 110-112 ℃. ^{1}H NMR ($CDCl_3$, 400.1 MHz): δ 8.43 (br.s, 1H), 7.90 (d, 1H, ^3J = 8.1 Hz), 7.39 (d, 1H, ^3J = 8.3 Hz), 7.32 (t, 1H, ^3J = 7.6 Hz), 7.23 (d, 1H, ^3J = 5.0 Hz), 7.15 (t, 1H, ^3J = 7.5 Hz), 6.86-6.92 (m, 1H), 6.83 (d, 1H, ^4J = 3.4 Hz), 6.03 (s, 1H), 3.41 (s, 3H). ^{13}C{^{1}H} NMR ($CDCl_3$, 100.6 MHz): δ 145.1, 135.3, 126.4, 125.2, 125.1, 125.0, 124.7, 123.0, 122.9 (q, $^2J_{CF}$ = 37.3 Hz), 121.6 (q, $^1J_{CF}$ = 269.4 Hz), 121.1, 117.3 (q, $^3J_{CF}$ = 2.6 Hz), 111.7, 74.3,

56.8. ^{19}F NMR ($CDCl_3$, 376.5 MHz): δ -58.5 (s, 3F). HRMS (ESI-TOF): m/z $[M+Na]^+$ Calcd for $C_{15}H_{12}F_3NOS^+$: 334.0484; found: 334.0475.

Synthesis of 3-(pyridin-4-ylmethyl)-2-(trifluoromethyl)-1H-indole (4s). 12 mL vial was charged with enamine **1a** (0.5 mmol), isonicotinaldehyde **2s** (0.0669 g, 0.625 mmol) and glacial acetic acid (1 mL). Reaction mixture was kept at 80-90 ℃ (hotplate stirrer) under stirring for 10 hours. The reaction mixture was cooled down to room temperature. Next, Pd/C (10%, 0.027 g, 0.025 mmol, 5 mol%) and formic acid (0.115 g, 2.5 mmol) was added and the reaction mixture was heated at 75 ℃ under stirring for 3 hours. The reaction mixture was filtred through a short celite pad and dispersed between water (10 mL) and CH_2Cl_2 (20 mL). Aqeous layer was separated and extracted with CH_2Cl_2 (3×10 mL). Combined organic phases were dried over Na_2SO_4, volatiles were evaporated in vacuo, the residue was purified by column chromatography on silica gel, using gradient elution by mixture hexane-CH_2Cl_2 (1:1) followed by CH_2Cl_2 and CH_2Cl_2-MeOH (100:1) as eluents. Pale yellow-brown powder, m.p. 185-187 ℃, yield 0.029 g (21%). ^1H NMR ($CDCl_3$, 400.1 MHz): δ 9.03 (br.s, 1H), 8.42-8.50 (m, 2H),7.46 (d, 1H, ^3J = 8.1 Hz), 7.43 (d, 2H, ^3J = 8.3 Hz), 7.32 (t, 1H, ^3J = 7.6 Hz), 7.11-7.18 (m, 3H), 4.26 (s, 2H). $^{13}C\{^1H\}$ NMR ($CDCl_3$, 100.6 MHz): δ 150.5, 136.8, 127.8, 125.7, 124.4, 123.2 (q, $^1J_{CF}$ = 268.2 Hz), 123.1 (q, $^2J_{CF}$ = 36.5 Hz), 121.5, 121.0, 115.1 (q, $^3J_{CF}$ = 2.7 Hz), 113.1, 29.5. ^{19}F NMR ($CDCl_3$, 376.5 MHz): δ -59.3 (s, 3F). HRMS (ESI-TOF): m/z $[M+H]^+$ Calcd for $C_{15}H_{12}F_3N_2^+$: 277.0947; found: 277.0950.

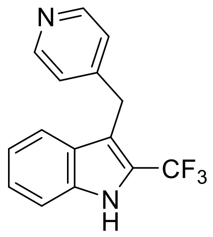

NMR data of the reaction intermediates

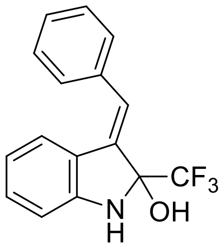

***(E)*-3-Benzylidene-2-(trifluoromethyl)indolin-2-ol (B)**. ^{1}H NMR ($CDCl_3$, 400.1 MHz): δ 7.34-7.48 (m, 5H), 7.22 (d, 1H, ^{3}J = 7.7 Hz), 7.06-7.16 (m, 2H), 6.64-6.70(m, 1H), 6.57-6.64 (m, 1H), 4.95 (s, 1H), 3.15 (br.s, 1H). ^{13}C{^{1}H} NMR ($CDCl_3$, 100.6 MHz): δ 148.7, 137.0, 135.7, 130.6, 128.6, 128.4, 128.1, 127.8 (d, $^{4}J_{CF}$ = 1.3 Hz), 123.8, 123.3 (q, $^{1}J_{CF}$ = 285.7 Hz), 122.7, 119.6, 109.7, 90.4 (q, $^{2}J_{CF}$ = 31.9 Hz). ^{19}F NMR ($CDCl_3$, 376.5 MHz): δ -85.4 (s, 3F).

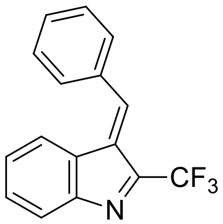

***(E)*-3-Benzylidene-2-(trifluoromethyl)-3*H*-indole (C)**. ^{1}H NMR ($CDCl_3$, 400.1 MHz): δ 7.95 (s, 1H), 7.67-7.78 (m, 3H), 7.50-7.56 (m, 3H), 7.40 (td, 1H, ^{3}J = 7.6 Hz, ^{4}J = 1.1 Hz), 7.15-7.28 (m, 2H). ^{13}C{^{1}H} NMR ($CDCl_3$, 100.6 MHz): δ 158.2 (q, $^{2}J_{CF}$ = 34.9 Hz), 153.3, 142.7 (q, $^{4}J_{CF}$ = 1.7 Hz), 133.9, 133.0, 130.9, 130.0, 129.9, 128.9, 127.7, 122.9, 122.7, 120.7 (q, $^{1}J_{CF}$ = 273.6 Hz). For major isomer: ^{19}F NMR ($CDCl_3$, 376.5 MHz): δ -62.7 (s, 3F). For minor isomer: ^{19}F NMR ($CDCl_3$, 376.5 MHz): δ -61.7 (s, 3F).

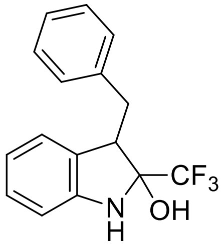

3-Benzyl-2-(trifluoromethyl)indolin-2-ol (D). ^{1}H NMR ($CDCl_3$, 400.1 MHz): δ 7.28-7.39 (m, 5H), 7.05-7.13 (m, 1H), 6.64-6.76 (m, 3H), 4.62 (s, 1H), 3.94 (dd, 1H, ^{3}J = 9.6 Hz, ^{3}J = 5.2 Hz), 3.37 (dd, 1H, ^{3}J = 14.2 Hz, ^{3}J = 5.2 Hz), 2.95 (dd, 1H, ^{3}J = 14.2 Hz, ^{3}J = 9.7 Hz), 2.79 (br.s, 1H). ^{13}C{^{1}H} NMR ($CDCl_3$, 100.6 MHz): δ 145.6,138.8, 129.1, 128.7, 128.6, 128.3, 126.7, 124.8, 124.0 (q, $^{1}J_{CF}$ = 284.1 Hz), 120.0, 92.5 (q, $^{2}J_{CF}$ = 30.9 Hz), 47.1, 34.4. ^{19}F NMR ($CDCl_3$, 376.5 MHz): δ -84.3 (s, 3F).

18 Jun 2020

Acquisition Time (sec)	4.0894	***Comment***	Imported from UXNMR.			***Date***	24 Sep 2019 14:58:12
File Name	C:\DOCS\OUTPUT_301\2019\09.皴青 狃黒BM-1712.H_001001r					***Frequency (MHz)***	400.13
Nucleus	1H	***Number of Transients***	4	***Original Points Count***	32768	***Points Count***	131072
Pulse Sequence	zg30	***Solvent***	CHLOROFORM-D			***Sweep Width (Hz)***	8012.82
Temperature (degree C)	27.000						

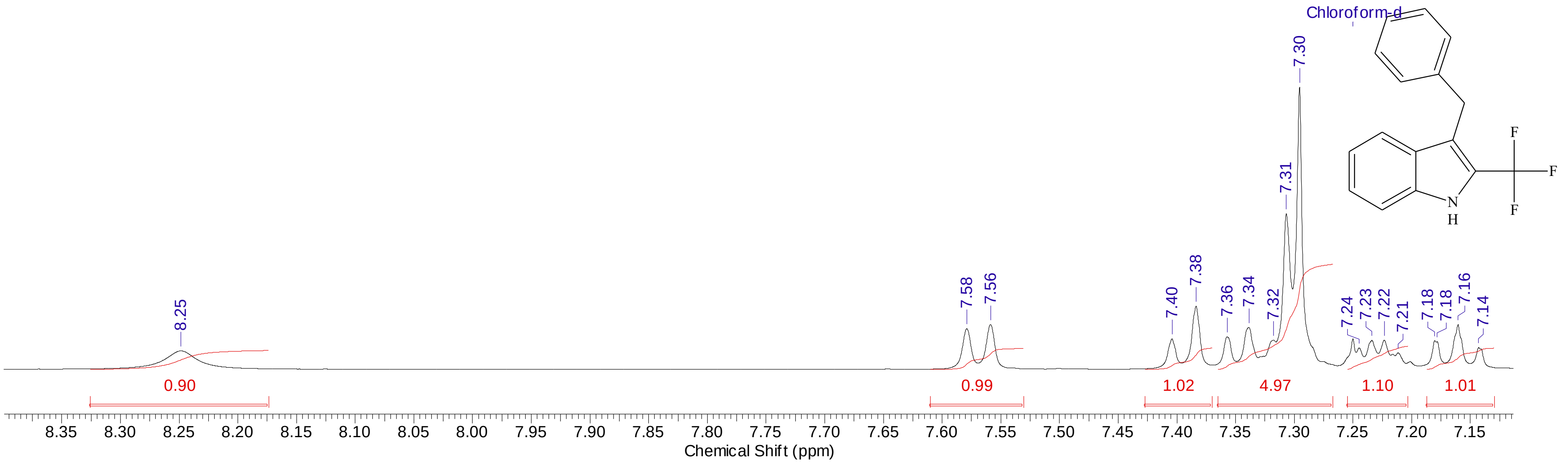

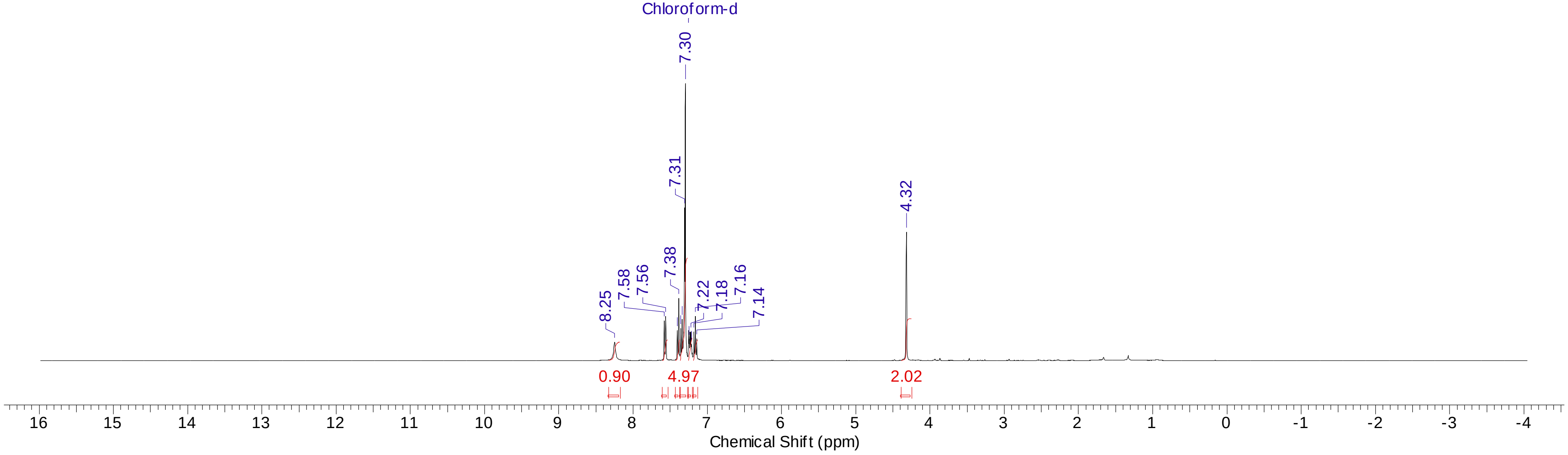

^{1}H NMR spectrum of **4a** (400.1 MHz, $CDCl_3$)

18 Jun 2020

Acquisition Time (sec)	1.5729	Date	Sep 26 2019	File Name	C:\DOCS\OUTPUT_301\F19\2019.09.26\BM-1712-F_20190926_01\FLUORINE_01		
Frequency (MHz)	376.33	Nucleus	19F	Number of Transients	8	Original Points Count	206956
Points Count	262144	Pulse Sequence	s2pul	Solvent	CHLOROFORM-D		
Sweep Width (Hz)	131578.95	Temperature (degree C)	20.000				

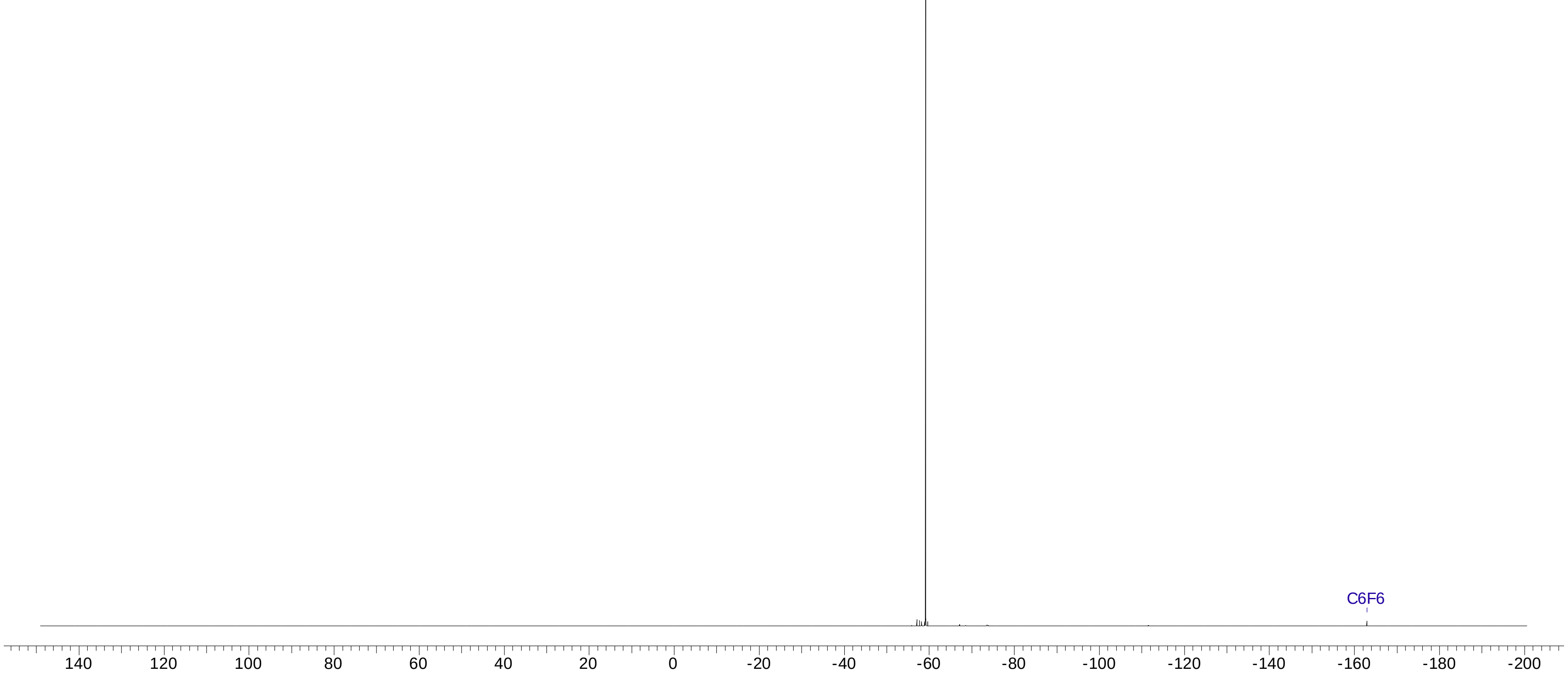

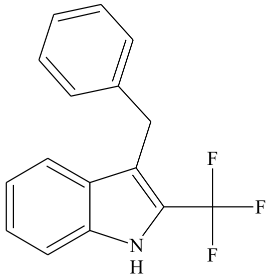

^{19}F NMR spectrum of **4a** (376.5 MHz, $CDCl_3$)

18 Jun 2020

Acquisition Time (sec)	0.6783	***Comment***	Imported from UXNMR.			***Date***	24 Sep 2019 15:09:48
File Name	C:\DOCS\OUTPUT_301\2019\09.皺靑 狃黒BM-1712.C_002001r					***Frequency (MHz)***	100.61
Nucleus	13C	***Number of Transients***	257	***Original Points Count***	16384	***Points Count***	131072
Pulse Sequence	zgpg30	***Solvent***	CHLOROFORM-D			***Sweep Width (Hz)***	24154.59
Temperature (degree C)	27.000						

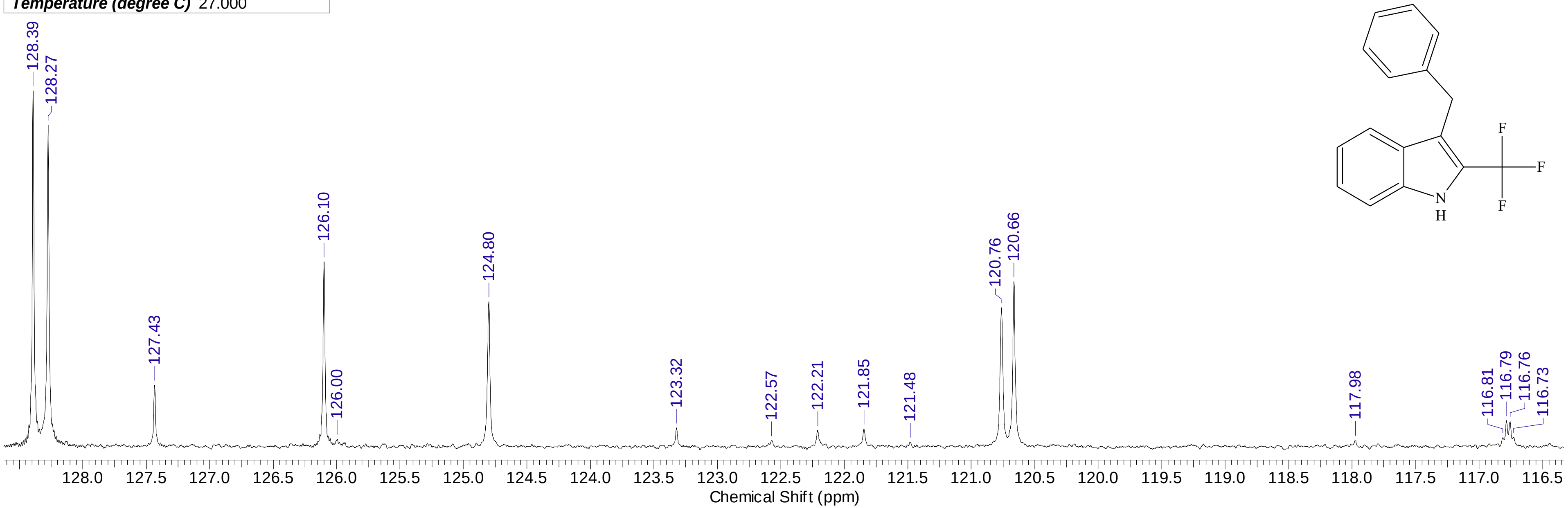

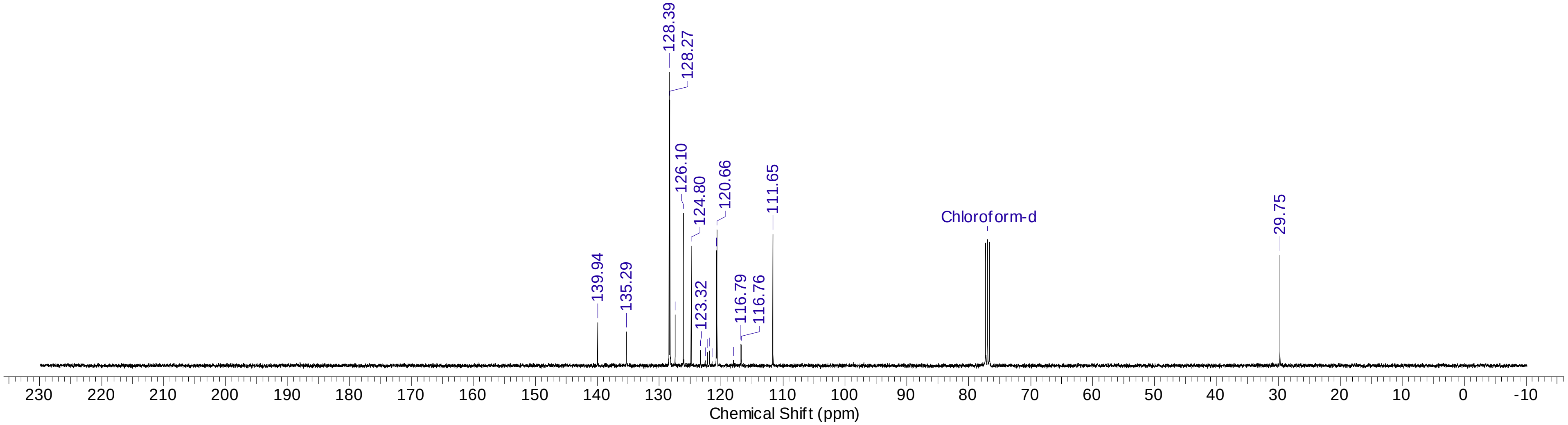

$^{13}C\{^1H\}$ NMR spectrum of **4a** (100.6 MHz, $CDCl_3$)

Acquisition Time (sec)	4.0894	***Comment***	Imported from UXNMR.	***Date***	30 Oct 2019 17:51:48
File Name	C:\DOCS\OUTPUT_301\2019\10.铌? 狃黒BM-1778.H_001001r			***Frequency (MHz)***	400.13
Nucleus	1H	***Number of Transients***	4 ***Original Points Count*** 32768	***Points Count***	131072
Pulse Sequence	zg30	***Solvent***	CHLOROFORM-D	***Sweep Width (Hz)***	8012.82
Temperature (degree C)	27.000				

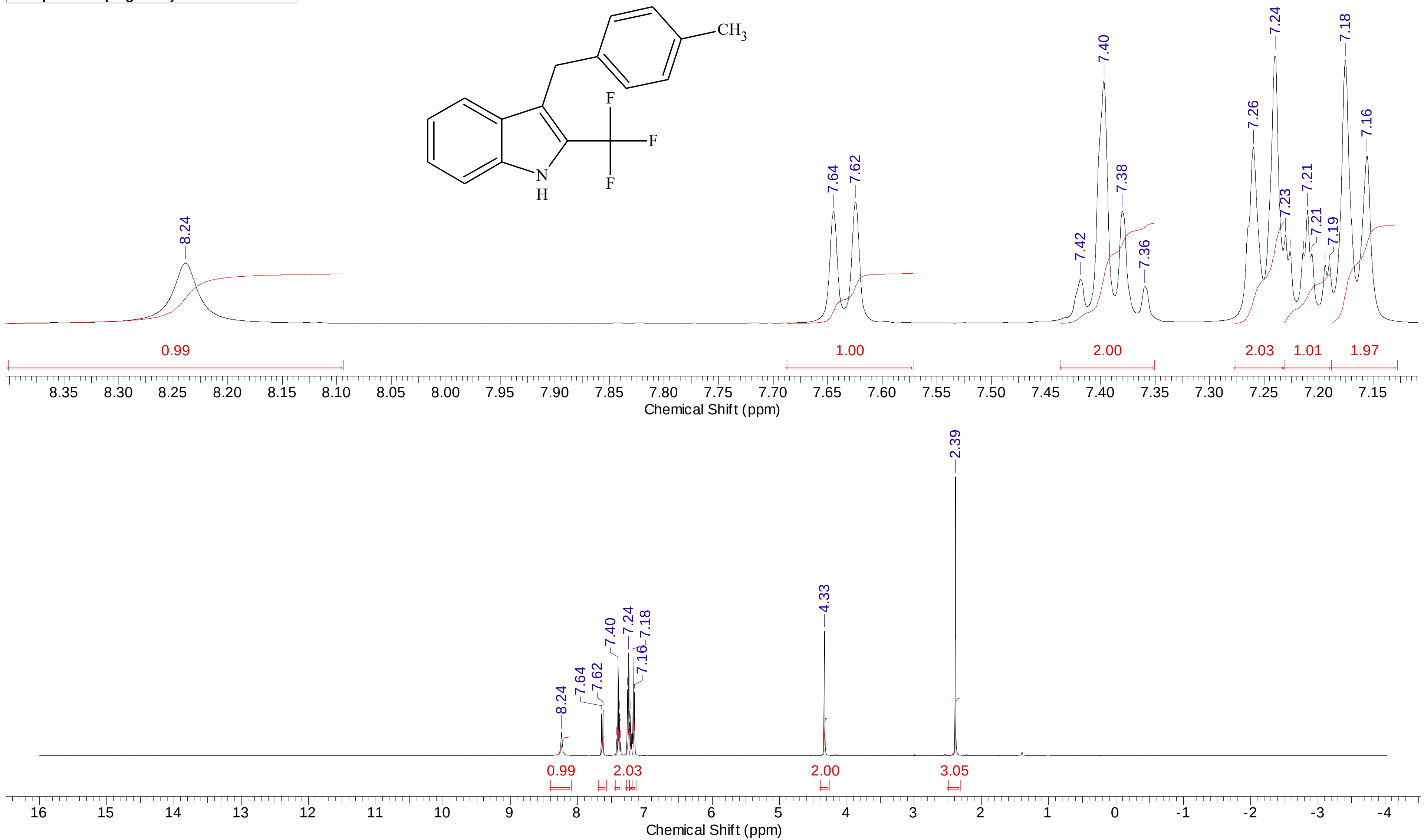

¹H NMR spectrum of **4b** (400.1 MHz, $CDCl_3$)

19 Jun 2020

Acquisition Time (sec)	1.5729	***Date***	Oct 31 2019	***File Name***	C:\DOCS\OUTPUT_301\F19\2019.10.31\BM-1778-F_20191031_01\FLUORINE_01		
Frequency (MHz)	376.33	***Nucleus***	19F	***Number of Transients***	8	***Original Points Count***	196608
Points Count	262144	***Pulse Sequence***	s2pul	***Solvent***	CHLOROFORM-D		
Sweep Width (Hz)	125000.00	***Temperature (degree C)***	30.000				

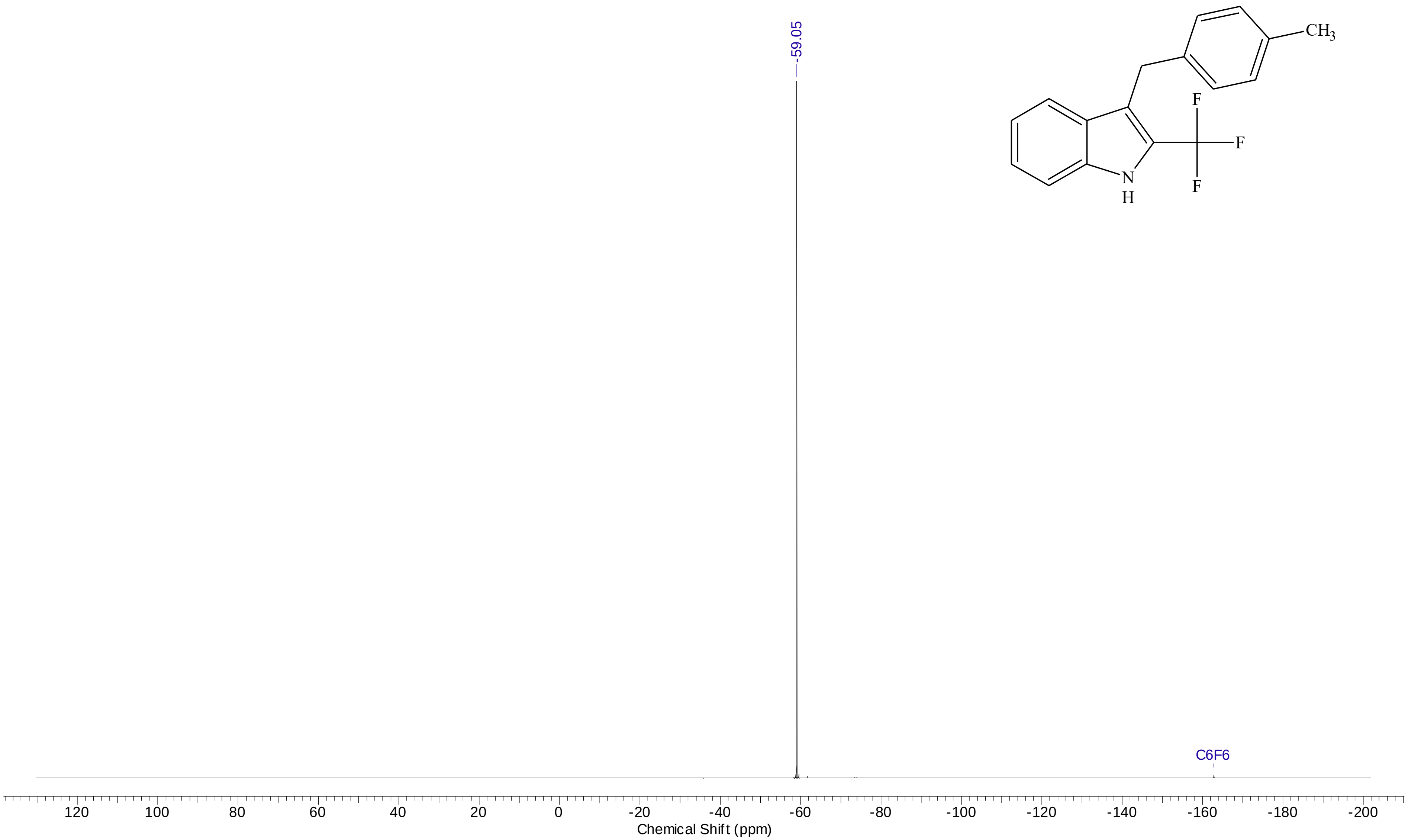

^{19}F NMR spectrum of **4b** (376.5 MHz, $CDCl_3$)

19 Jun 2020

Acquisition Time (sec)	0.6783	Comment	Imported from UXNMR.			Date	30 Oct 2019 17:57:40
File Name	C:\DOCS\OUTPUT_301\2019\10.铌? 狃黒BM-1778.C_002001r					Frequency (MHz)	100.61
Nucleus	13C	Number of Transients	137	Original Points Count	16384	Points Count	131072
Pulse Sequence	zgpg30	Solvent	CHLOROFORM-D			Sweep Width (Hz)	24154.59
Temperature (degree C)	27.000						

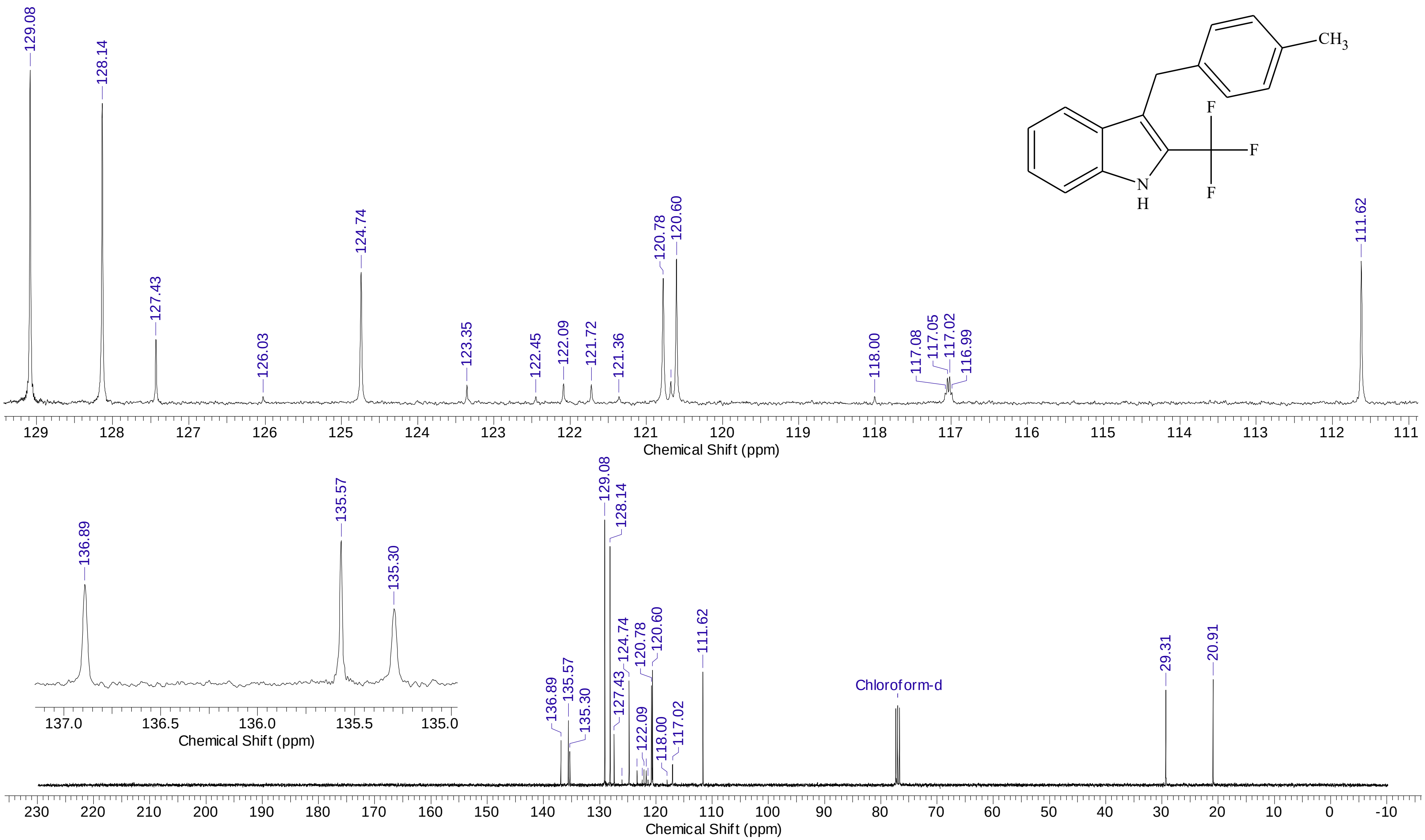

$^{13}C\{^{1}H\}$ NMR spectrum of **4b** (100.6 MHz, $CDCl_3$)

3 Jul 2021

Acquisition Time (sec)	4.0894	***Comment***	Imported from UXNMR.			***Date***	27 Nov 2019 17:46:56
File Name	C:\DOCS\OUTPUT_301\2019\11.肶 狃黒BM-1801.H_001001r					***Frequency (MHz)***	400.13
Nucleus	1H	***Number of Transients***	4	***Original Points Count***	32768	***Points Count***	131072
Pulse Sequence	zg30	***Solvent***	DMSO-D6	***Sweep Width (Hz)***	8012.82	***Temperature (degree C)***	27.000

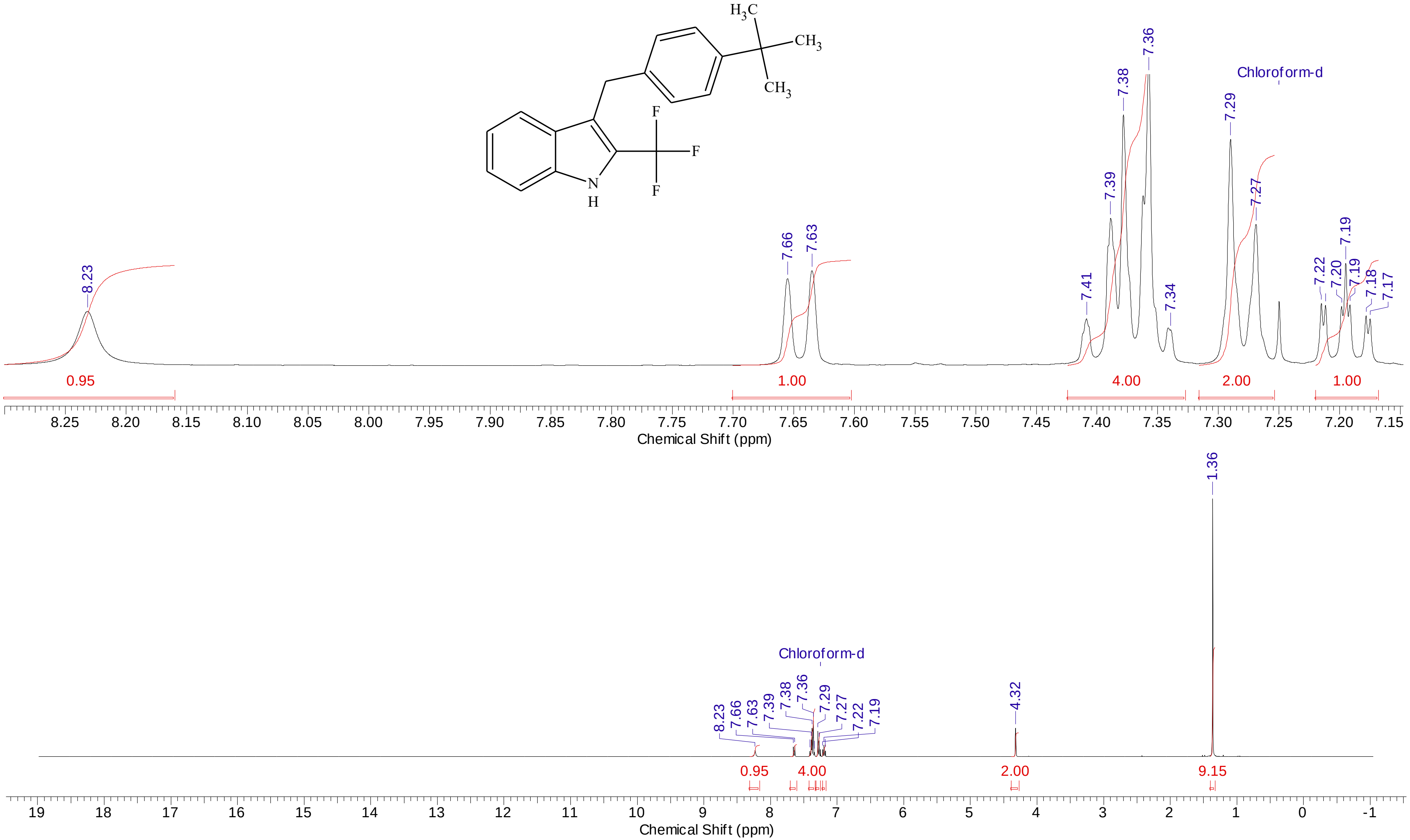

^{1}H NMR spectrum of **4c** (400.1 MHz, $CDCl_3$)

19 Jun 2020

Acquisition Time (sec)	2.3069	***Date***	Nov 28 2019	***File Name***	C:\DOCS\OUTPUT_301\F19\2019.11.28\BM-1801-F_20191128_01\FLUORINE_01		
Frequency (MHz)	376.32	***Nucleus***	19F	***Number of Transients***	8	***Original Points Count***	262144
Points Count	262144	***Pulse Sequence***	s2pul	***Solvent***	CHLOROFORM-D		
Sweep Width (Hz)	113636.37	***Temperature (degree C)***	22.000				

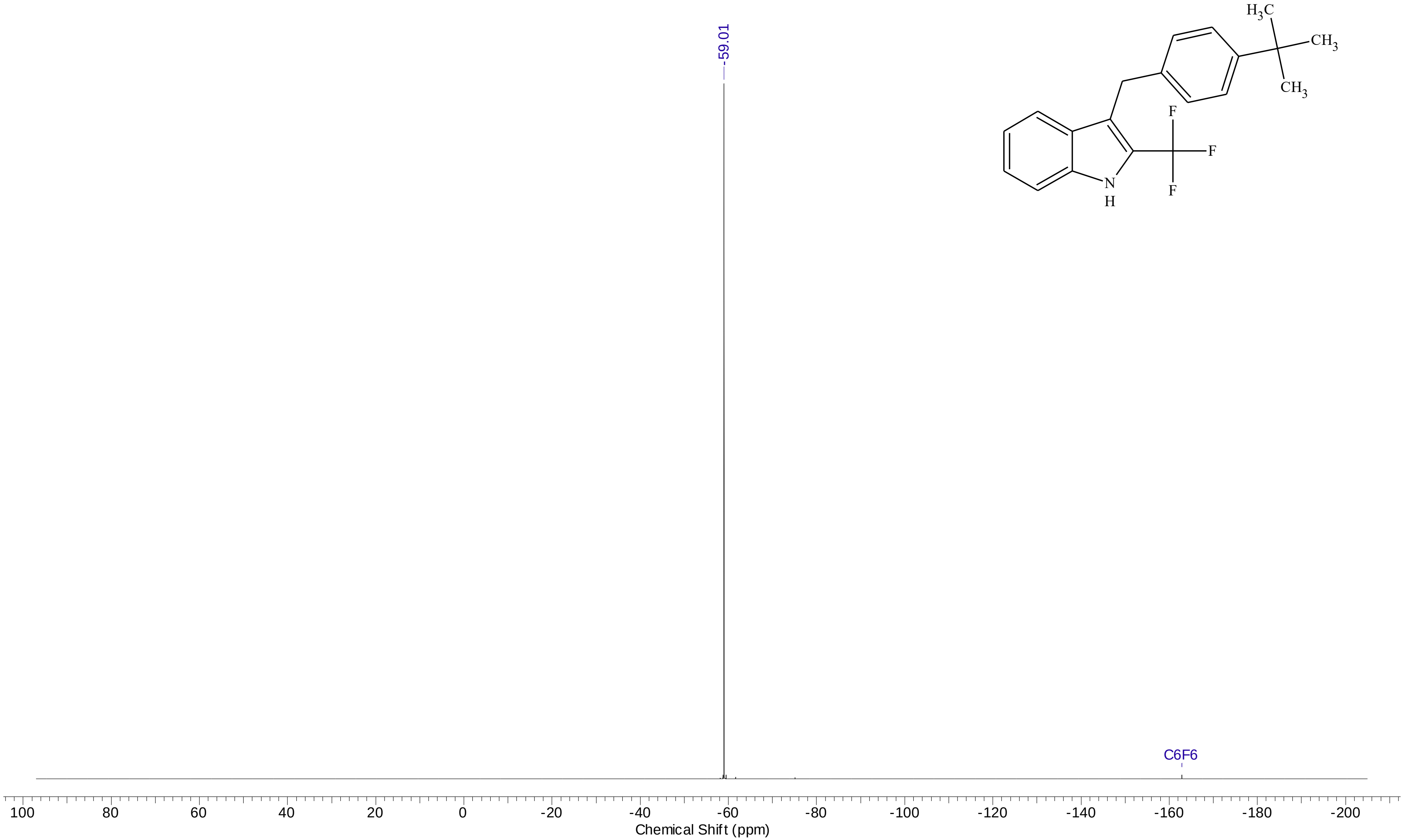

^{19}F NMR spectrum of **4c** (376.5 MHz, $CDCl_3$)

19 Jun 2020

Acquisition Time (sec)	0.6783	Comment	Imported from UXNMR.			Date	27 Nov 2019 17:50:18
File Name	C:\DOCS\OUTPUT_301\2019\11.朖 狃黒BM-1801.C_002001r			Frequency (MHz)	100.61	Nucleus	13C
Number of Transients	73	Original Points Count	16384	Points Count	131072	Pulse Sequence	zgpg30
Solvent	CHLOROFORM-D			Sweep Width (Hz)	24154.59	Temperature (degree C)	27.000

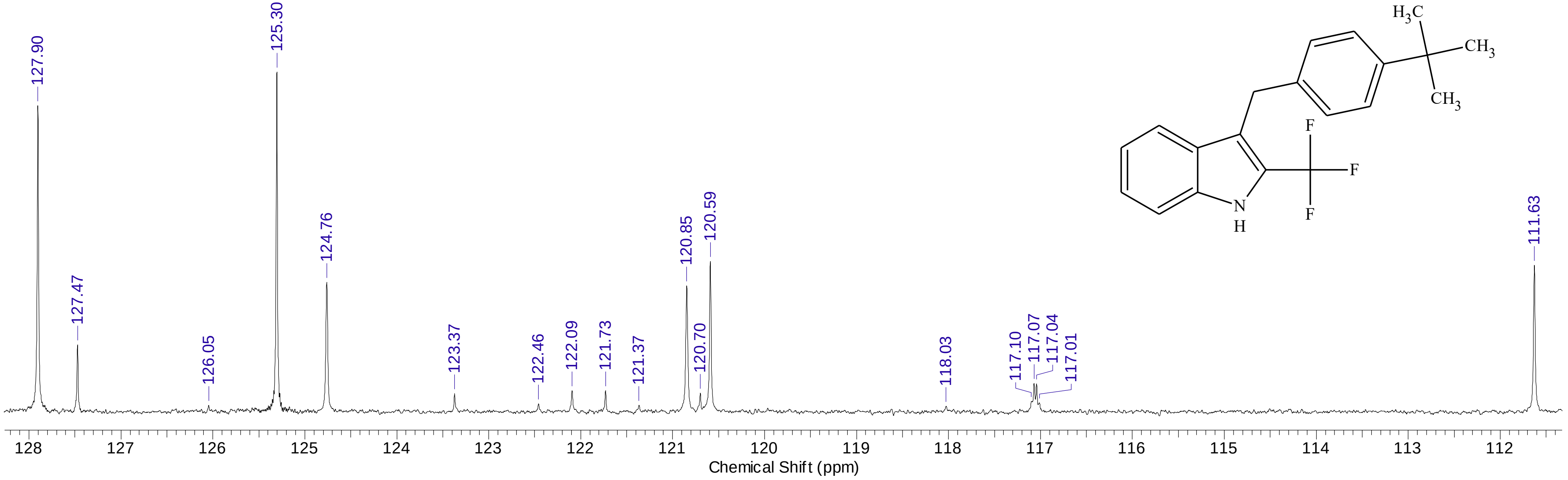

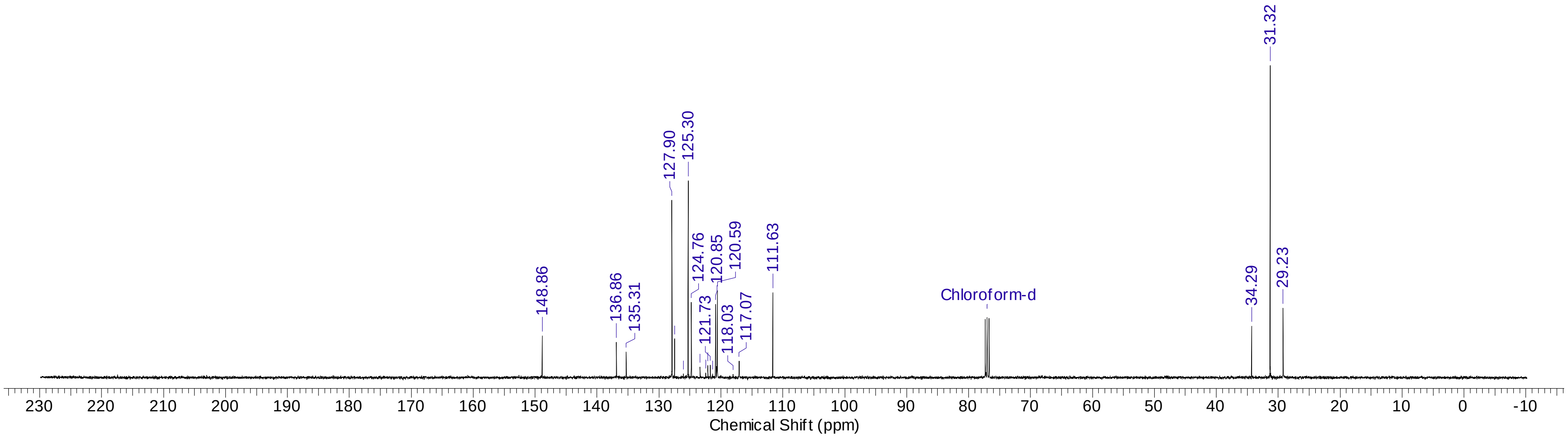

$^{13}C\{^{1}H\}$ NMR spectrum of **4c** (100.6 MHz, $CDCl_3$)

19 Jun 2020

Acquisition Time (sec)	4.0894	***Comment***	Imported from UXNMR.			***Date***	01 Nov 2019 15:37:42
File Name	C:\DOCS\OUTPUT_301\2019\11.眍 狃黒BM-1780.H_001001r					***Frequency (MHz)***	400.13
Nucleus	1H	***Number of Transients***	4	***Original Points Count***	32768	***Points Count***	131072
Pulse Sequence	zg30	***Solvent***	DMSO-D6	***Sweep Width (Hz)***	8012.82	***Temperature (degree C)***	27.000

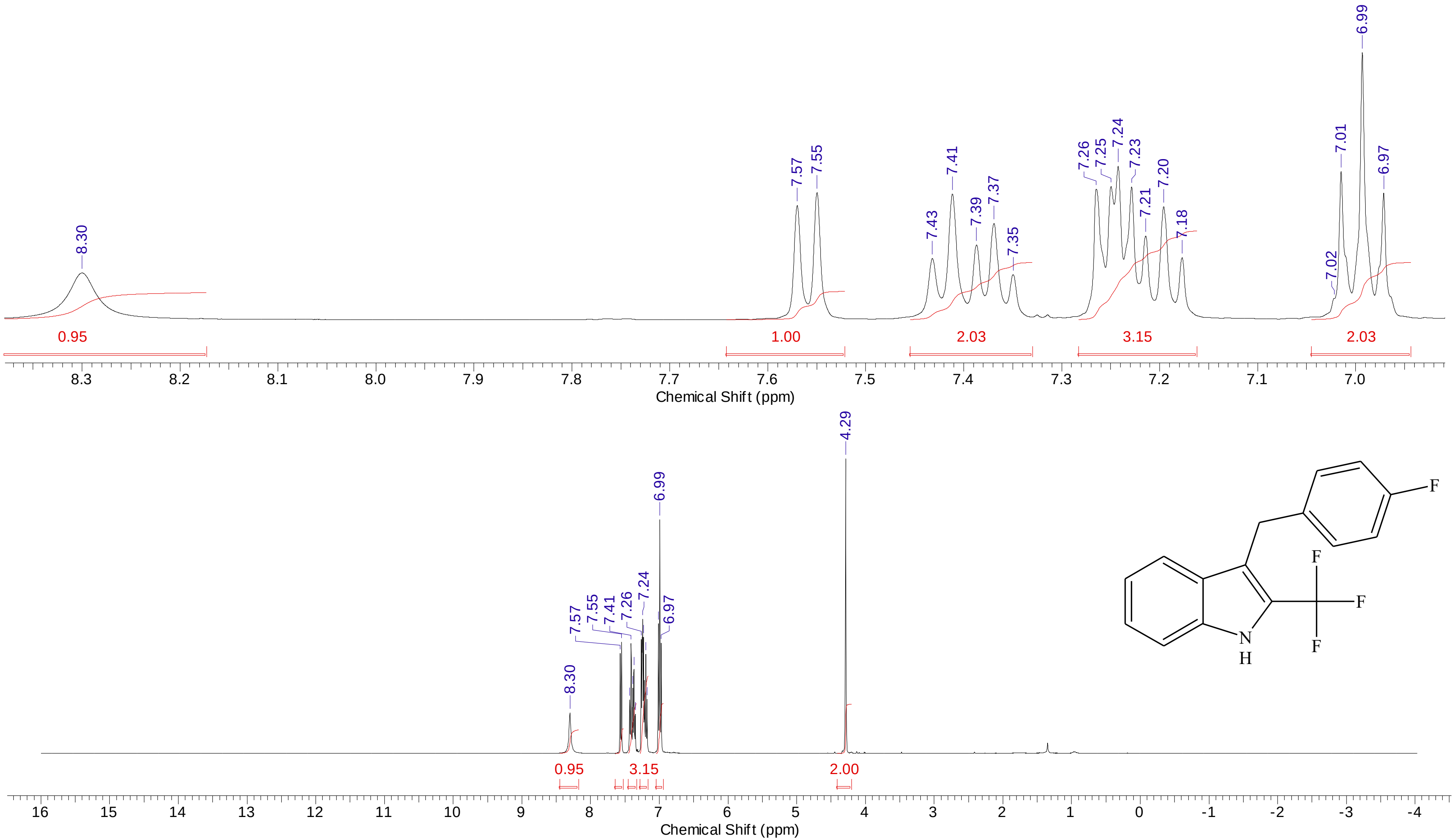

^{1}H NMR spectrum of **4d** (400.1 MHz, $CDCl_3$)

19 Jun 2020

Acquisition Time (sec)	1.5729	Date	Nov 7 2019	File Name	C:\DOCS\OUTPUT_301\F19\2019.11.07\BM-1780-F_20191107_01\FLUORINE_01		
Frequency (MHz)	376.33	Nucleus	19F	Number of Transients	8	Original Points Count	196608
Points Count	262144	Pulse Sequence	s2pul	Solvent	CHLOROFORM-D		
Sweep Width (Hz)	125000.00	Temperature (degree C)	30.000				

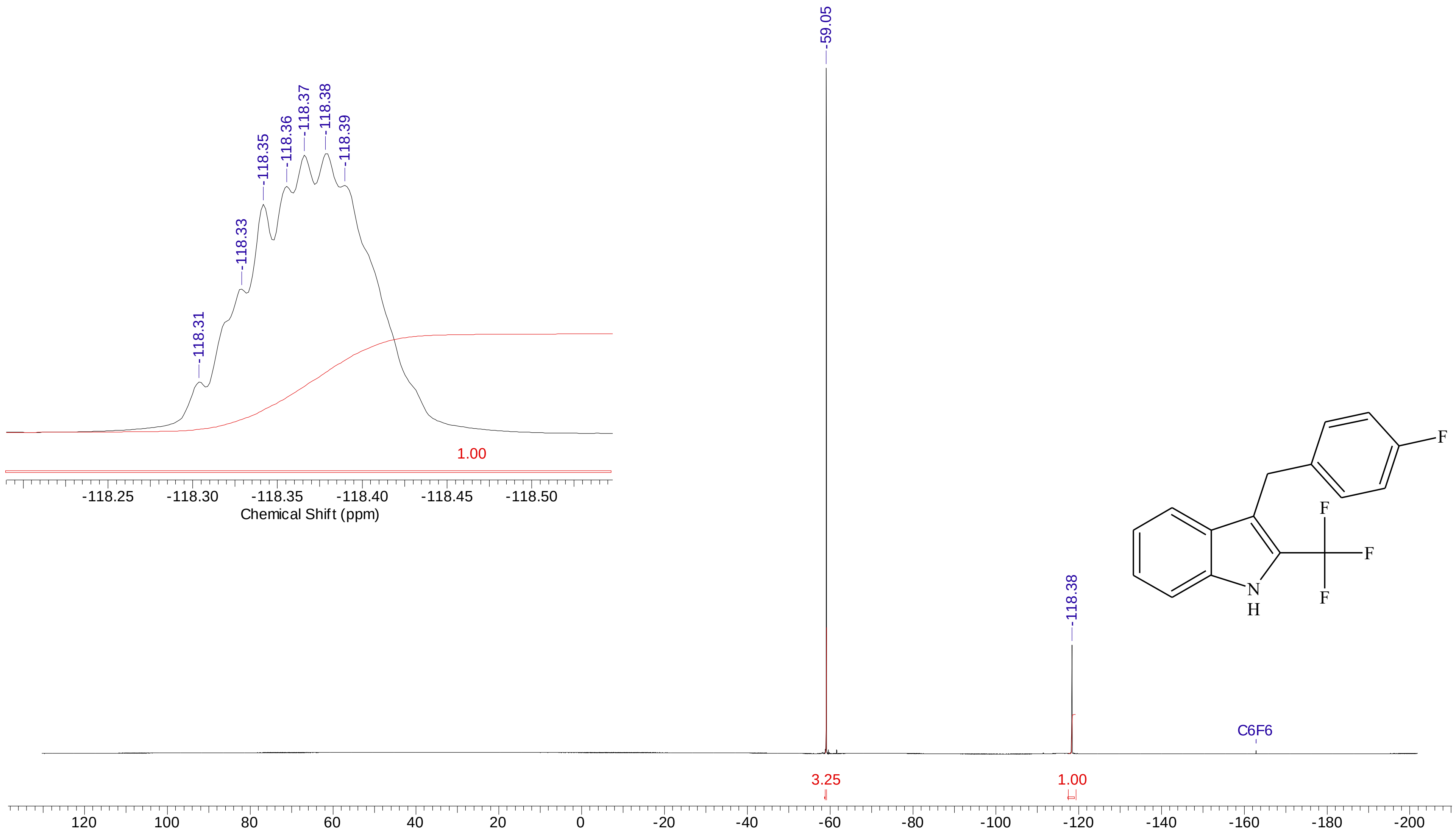

^{19}F NMR spectrum of **4d** (376.5 MHz, $CDCl_3$)

10 Jul 2021

Acquisition Time (sec)	0.6783	Comment	Imported from UXNMR.	Date	01 Nov 2019 15:42:56
File Name	C:\DOCS\OUTPUT_301\2019\11.朊 狃黒BM-1780.C_002001r	Frequency (MHz)	100.61	Nucleus	13C
Number of Transients	122	Original Points Count 16384	Points Count 131072	Pulse Sequence	zgpg30
Solvent	CHLOROFORM-D	Sweep Width (Hz)	24154.59	Temperature (degree C)	27.000

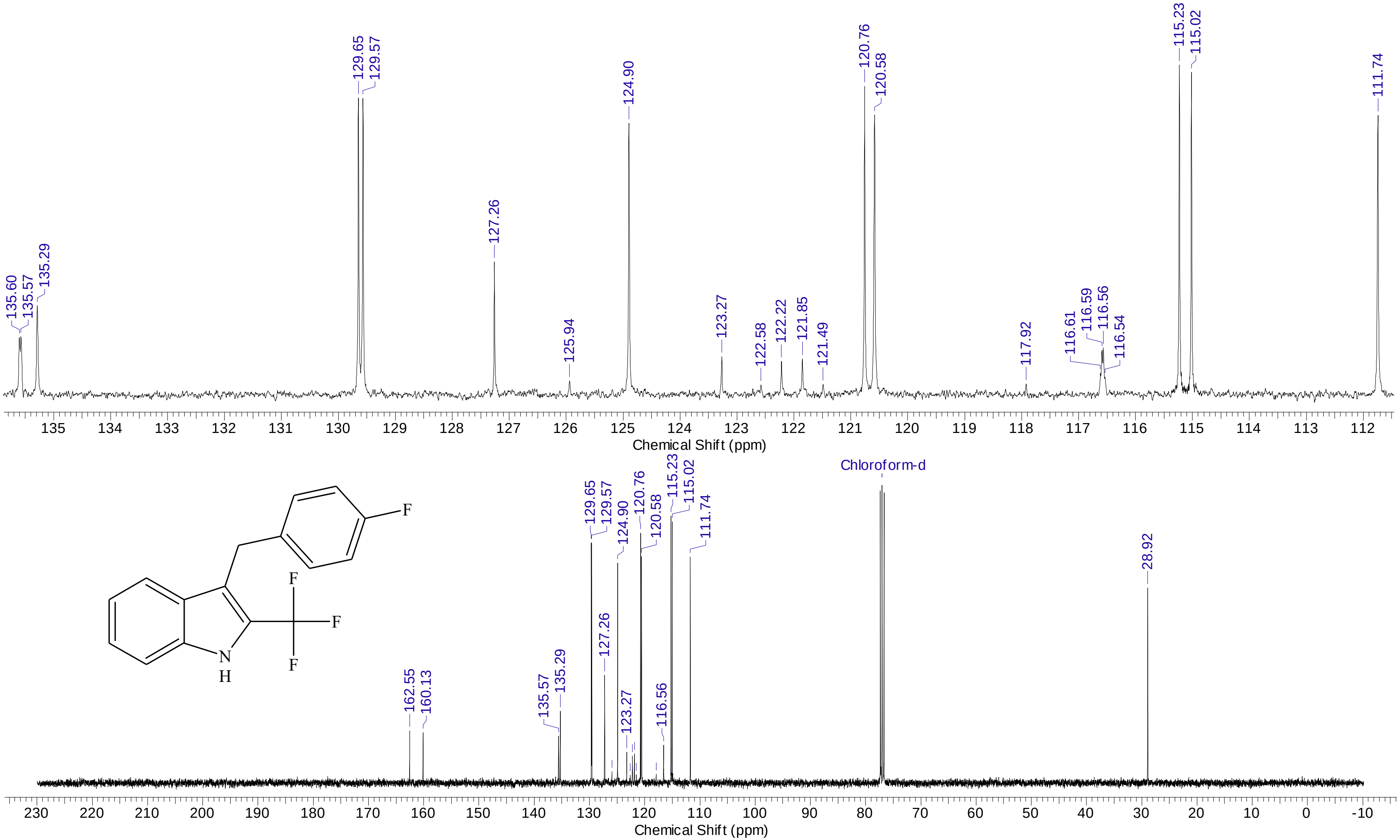

$^{13}C\{^1H\}$ NMR spectrum of **4d** (100.6 MHz, $CDCl_3$).

19 Jun 2020

Acquisition Time (sec)	4.0894	***Comment***	Imported from UXNMR.			***Date***	27 Nov 2019 17:28:52
File Name	C:\DOCS\OUTPUT_301\2019\11.朊 狃黒BM-1803.H_001001r					***Frequency (MHz)***	400.13
Nucleus	1H	***Number of Transients***	4	***Original Points Count***	32768	***Points Count***	131072
Pulse Sequence	zg30	***Solvent***	DMSO-D6	***Sweep Width (Hz)***	8012.82	***Temperature (degree C)***	27.000

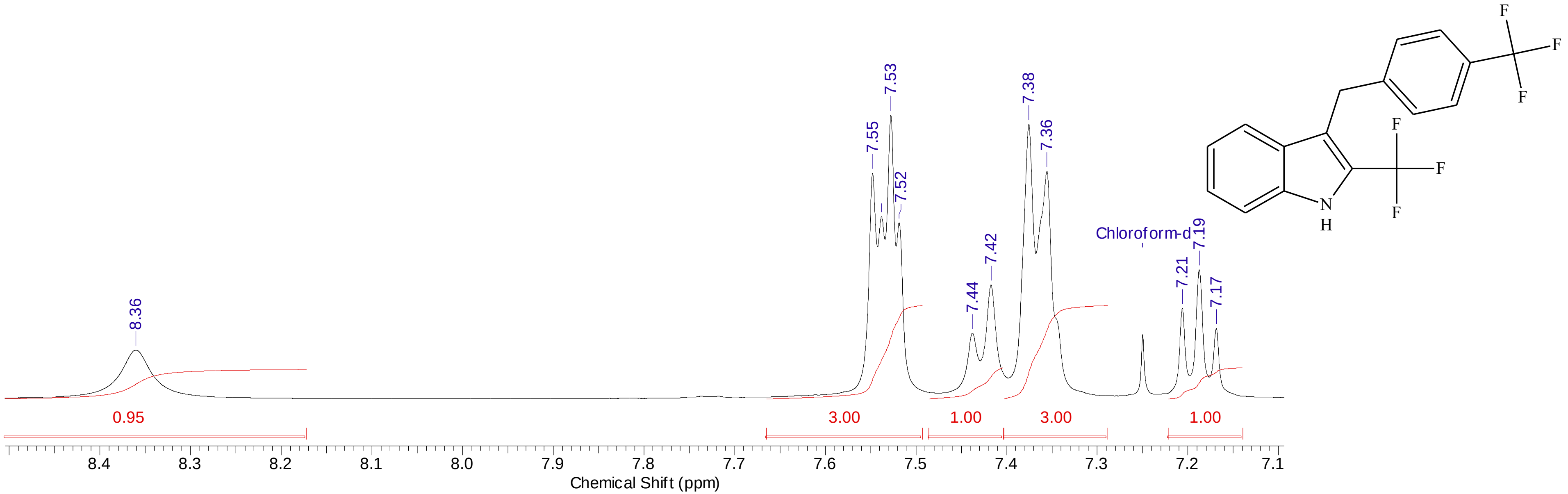

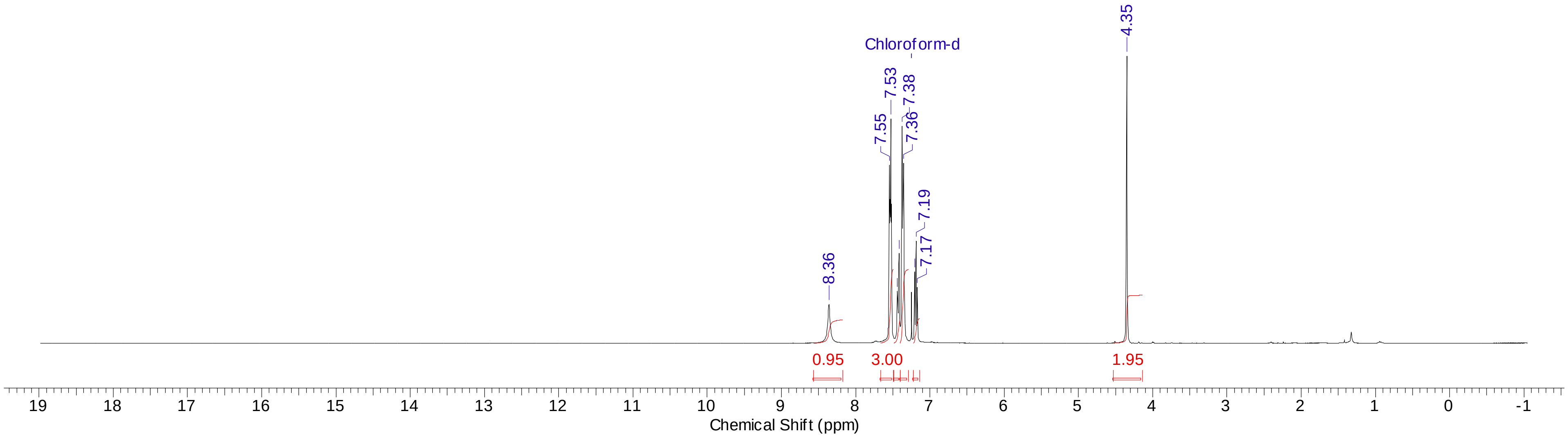

^1H NMR spectrum of **4e** (400.1 MHz, $CDCl_3$)

19 Jun 2020

Acquisition Time (sec)	1.0000	***Date***	Nov 25 2019	***File Name***	C:\DOCS\OUTPUT_301\F19\2019.11.25\BM-1803_20191125_01\FLUORINE_01		
Frequency (MHz)	376.31	***Nucleus***	19F	***Number of Transients***	16	***Original Points Count***	89286
Points Count	131072	***Pulse Sequence***	s2pul	***Solvent***	CHLOROFORM-D		
Sweep Width (Hz)	89285.71	***Temperature (degree C)***	20.000				

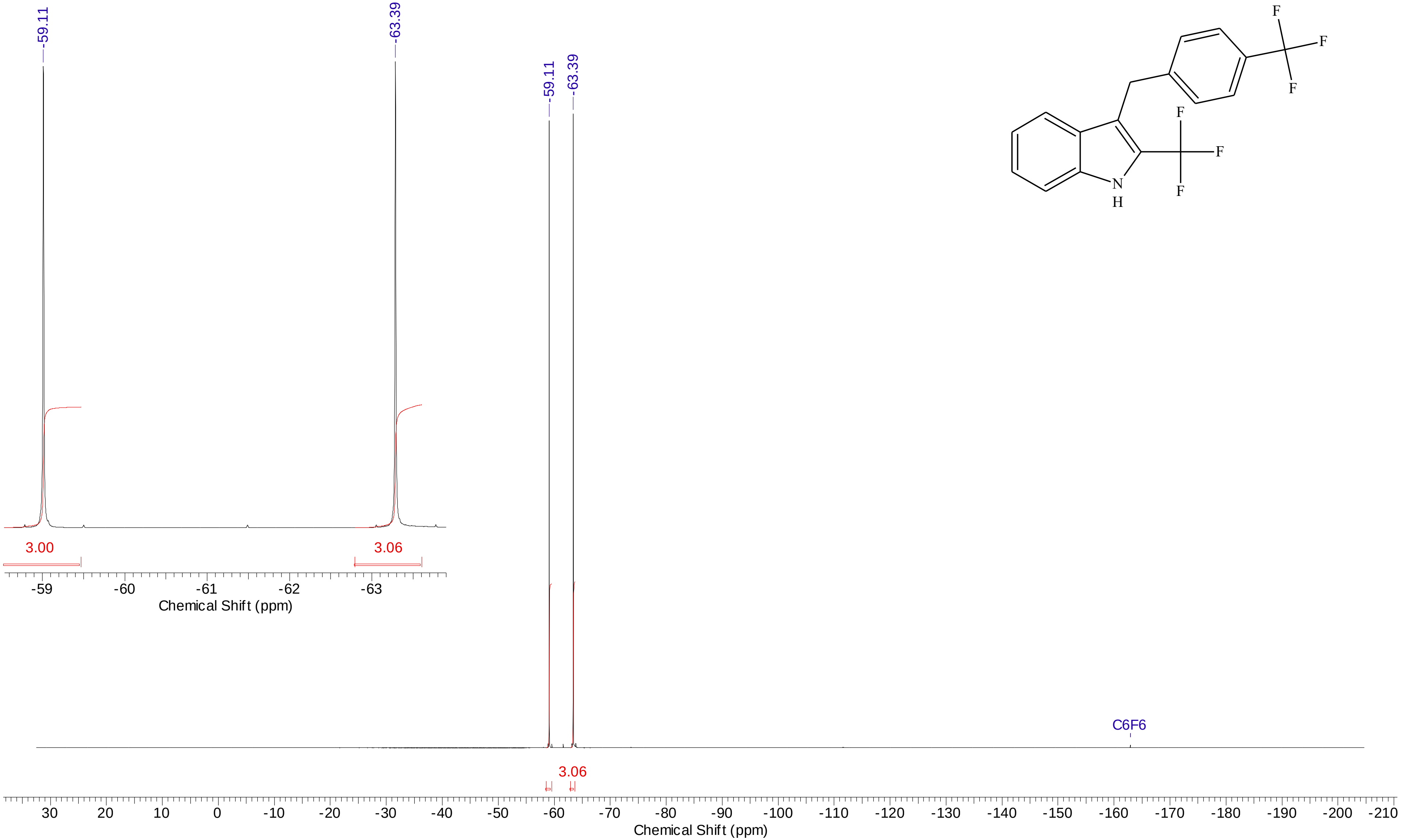

^{19}F NMR spectrum of **4e** (376.5 MHz, $CDCl_3$)

19 Jun 2020

Acquisition Time (sec)	0.6783	***Comment***	Imported from UXNMR.			***Date***	26 Nov 2019 13:12:08
File Name	C:\DOCS\OUTPUT_301\2019\11.眍 狃黒BM-1803.C_002001r			***Frequency (MHz)***	100.61	***Nucleus***	13C
Number of Transients	210	***Original Points Count***	16384	***Points Count***	131072	***Pulse Sequence***	zgpg30
Solvent	CHLOROFORM-D			***Sweep Width (Hz)***	24154.59	***Temperature (degree C)***	27.000

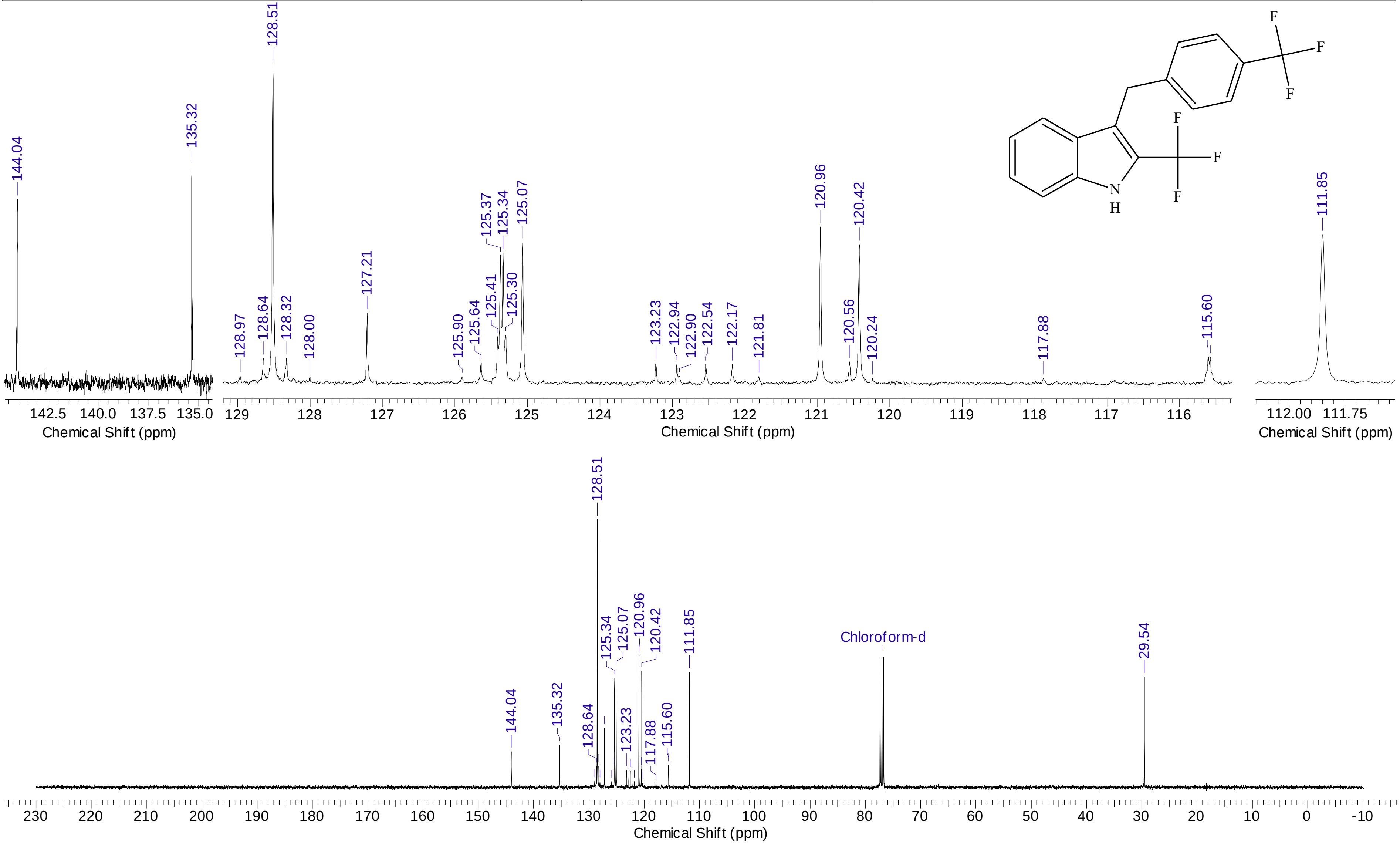

$^{13}C\{^{1}H\}$ NMR spectrum of **4e** (100.6 MHz, $CDCl_3$)

19 Jun 2020

Acquisition Time (sec)	4.0894	***Comment***	Imported from UXNMR.		***Date***	26 Nov 2019 20:56:52	
File Name	C:\DOCS\BM\bm191126\BM-1805_001001r		***Frequency (MHz)***	400.13	***Nucleus***	1H	***Number of Transients*** 8
Original Points Count	32768	***Points Count*** 131072	***Pulse Sequence***	zg30	***Solvent***	CHLOROFORM-D	
Sweep Width (Hz)	8012.82	***Temperature (degree C)*** 27.000					

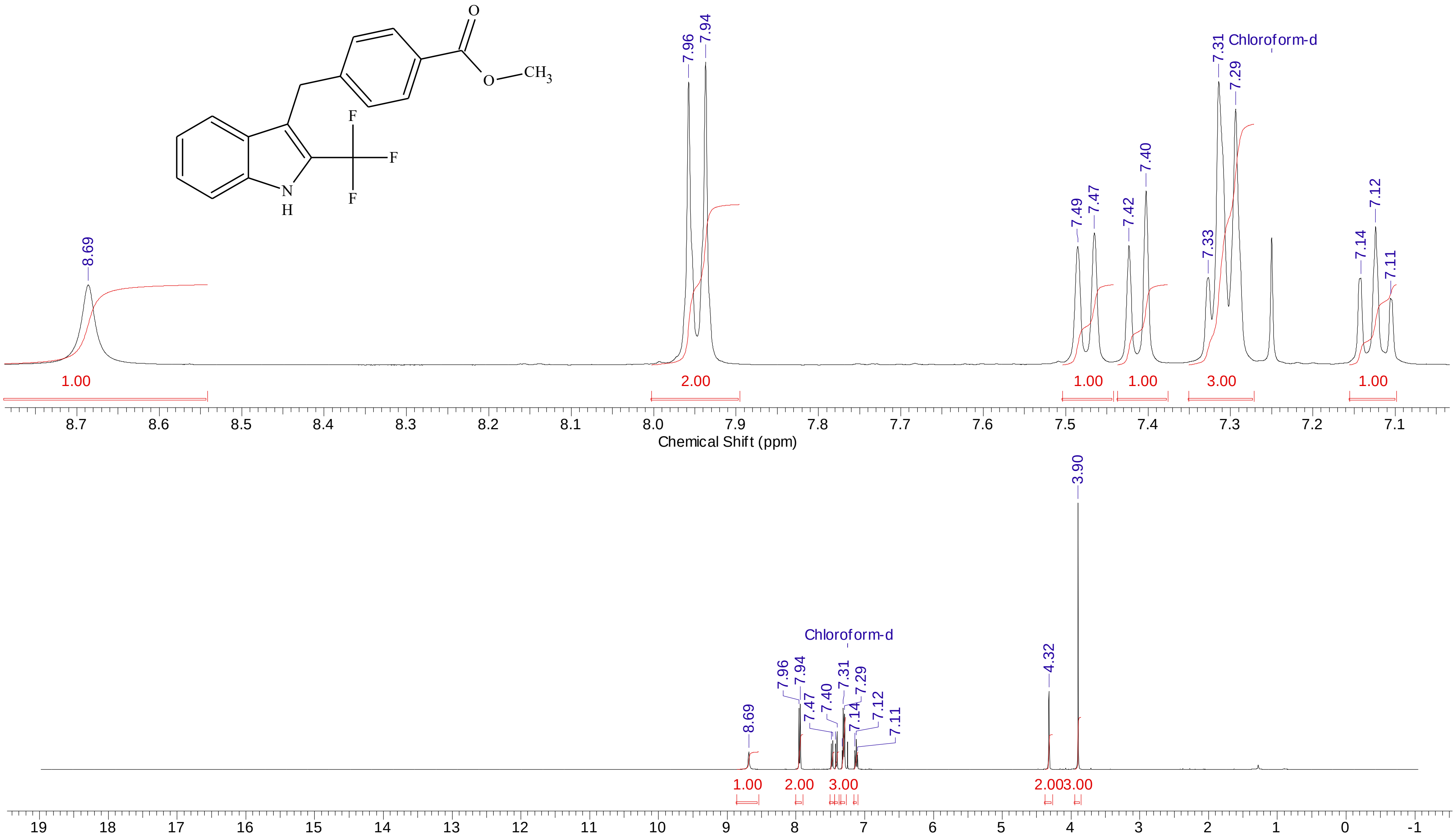

^{1}H NMR spectrum of **4f** (400.1 MHz, $CDCl_3$)

19 Jun 2020

Acquisition Time (sec)	0.7340	***Date***	Nov 26 2019	***File Name***	C:\DOCS\OUTPUT_301\F19\2019.11.26\bm1805-f_20191126_01\FLUORINE_01		
Frequency (MHz)	376.31	***Nucleus***	19F	***Number of Transients***	100	***Original Points Count***	65536
Points Count	65536	***Pulse Sequence***	s2pul	***Solvent***	CHLOROFORM-D		
Sweep Width (Hz)	89285.71	***Temperature (degree C)***	20.000				

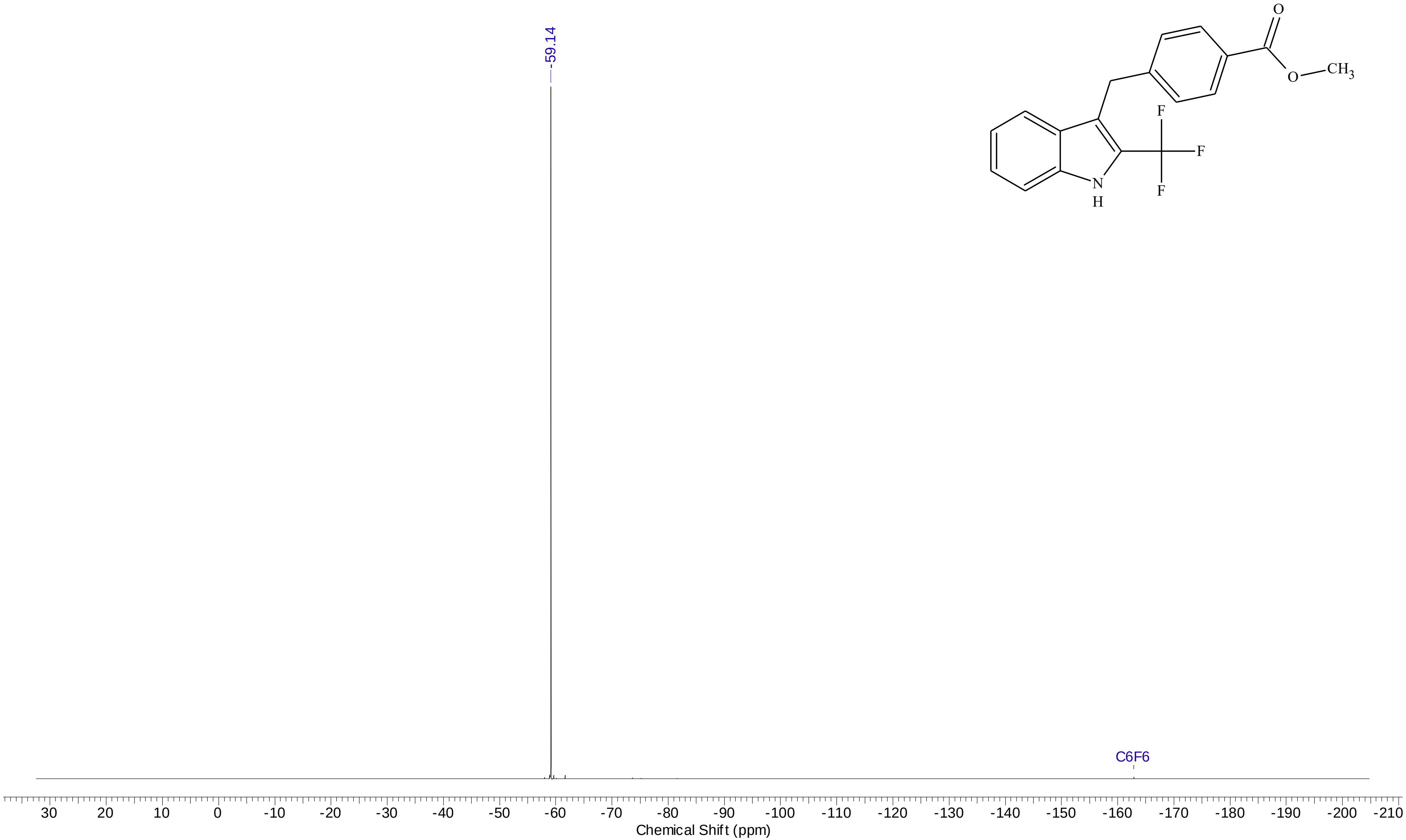

^{19}F NMR spectrum of **4f** (376.5 MHz, $CDCl_3$)

19 Jun 2020

Acquisition Time (sec)	0.6783	Comment	Imported from UXNMR.			Date	26 Nov 2019 21:00:54		
File Name	C:\DOCS\BM\bm191126\BM-1805_002001r			Frequency (MHz)	100.61	Nucleus	13C	Number of Transients	72
Original Points Count	16384	Points Count	131072	Pulse Sequence	zgpg30	Solvent	CHLOROFORM-D		
Sweep Width (Hz)	24154.59	Temperature (degree C)	27.000						

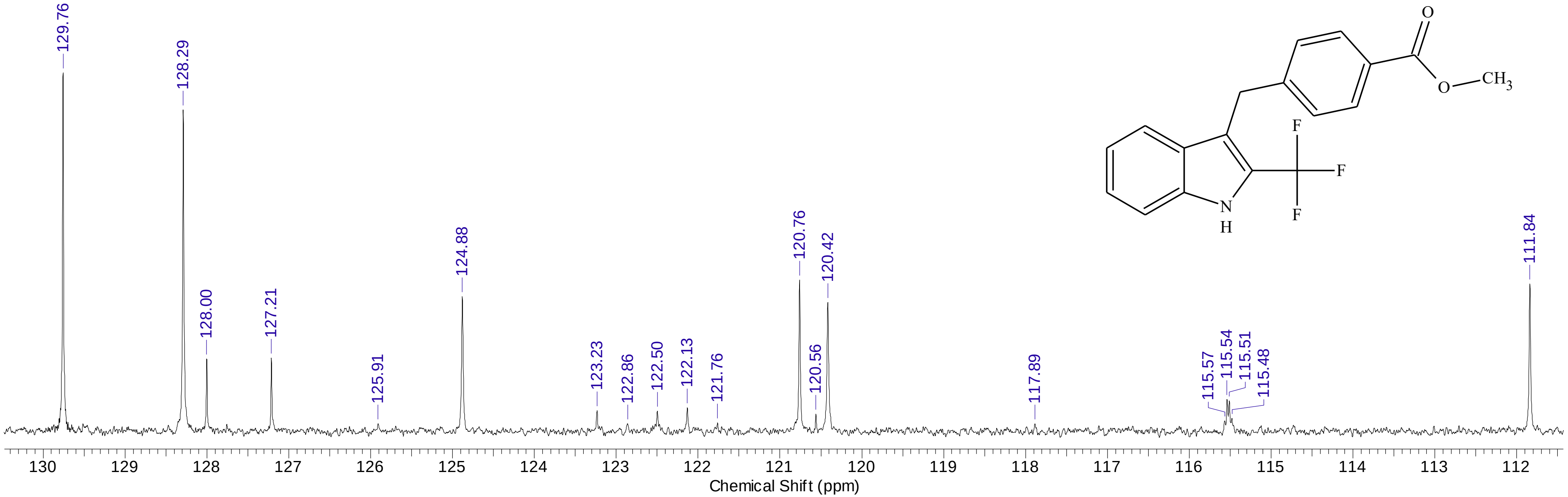

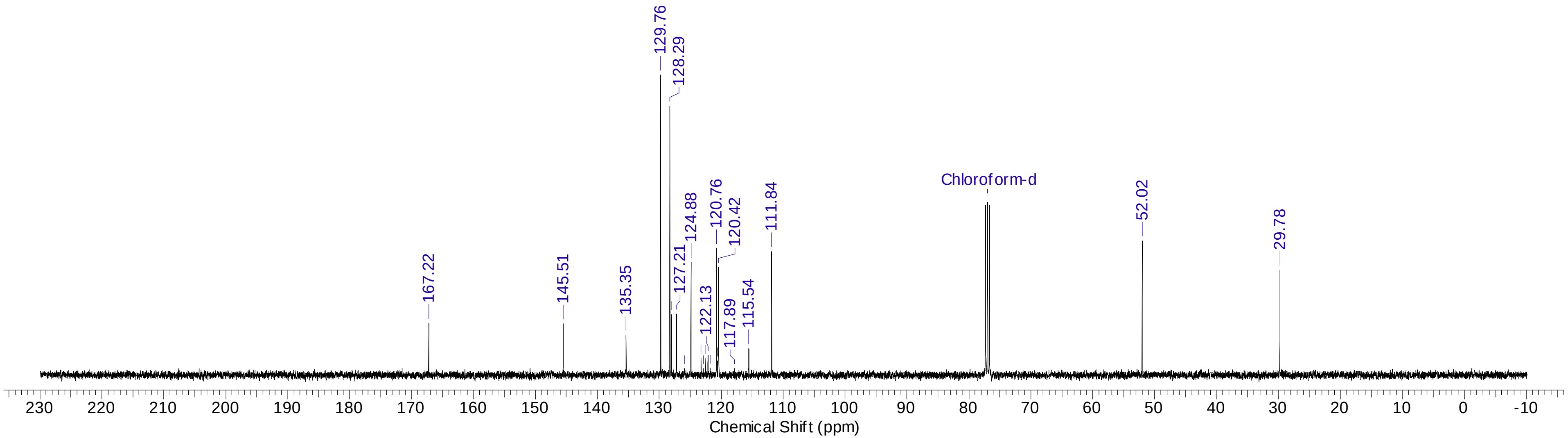

$^{13}C\{^{1}H\}$ NMR spectrum of **4f** (100.6 MHz, $CDCl_3$)

18 Jun 2020

Acquisition Time (sec)	4.0894	***Comment***	Imported from UXNMR.			***Date***	11 Oct 2019 15:35:34
File Name	C:\DOCS\OUTPUT_301\2019\10.铌? 狃黒BM-1732-1.H_001001r					***Frequency (MHz)***	400.13
Nucleus	1H	***Number of Transients***	4	***Original Points Count***	32768	***Points Count***	131072
Pulse Sequence	zg30	***Solvent***	CHLOROFORM-D			***Sweep Width (Hz)***	8012.82
Temperature (degree C)	27.000						

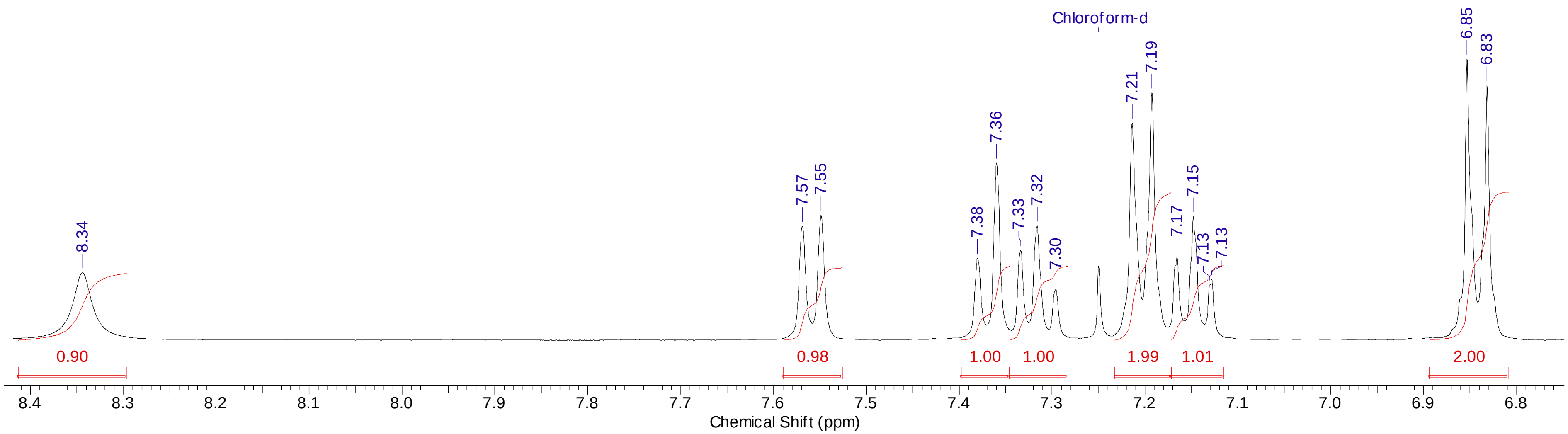

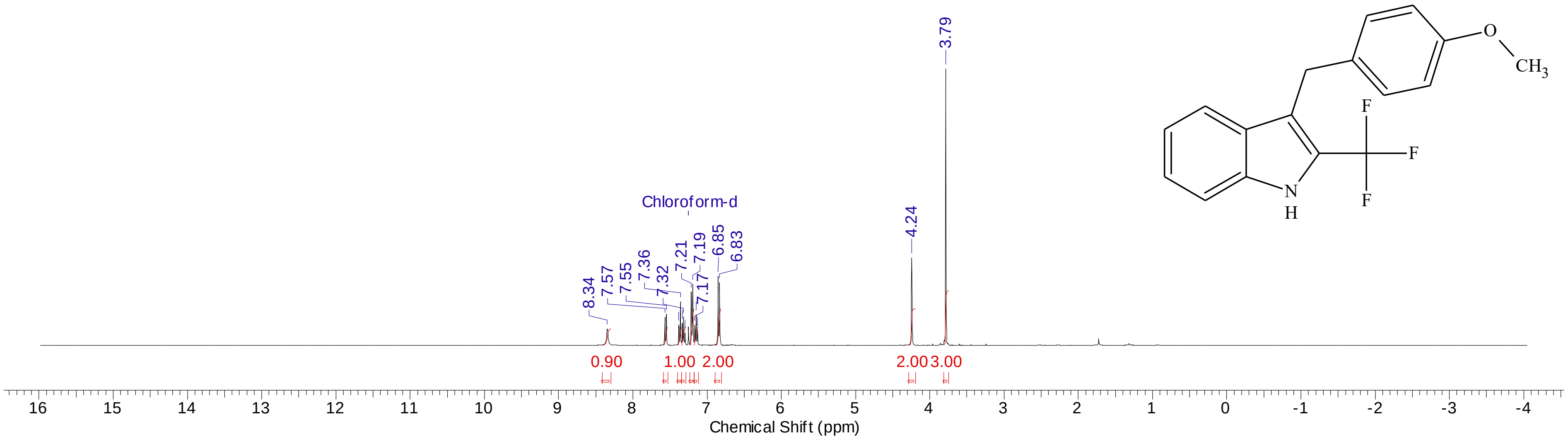

^{1}H NMR spectrum of **4g** (400.1 MHz, $CDCl_3$)

18 Jun 2020

Acquisition Time (sec)	2.0000	***Date***	Oct 14 2019	***File Name***	C:\DOCS\OUTPUT_301\F19\2019.10.14\BM-1732-1_20191014_01\FLUORINE_01		
Frequency (MHz)	376.31	***Nucleus***	19F	***Number of Transients***	16	***Original Points Count***	178571
Points Count	262144	***Pulse Sequence***	s2pul	***Solvent***	CHLOROFORM-D		
Sweep Width (Hz)	89285.71	***Temperature (degree C)***	30.000				

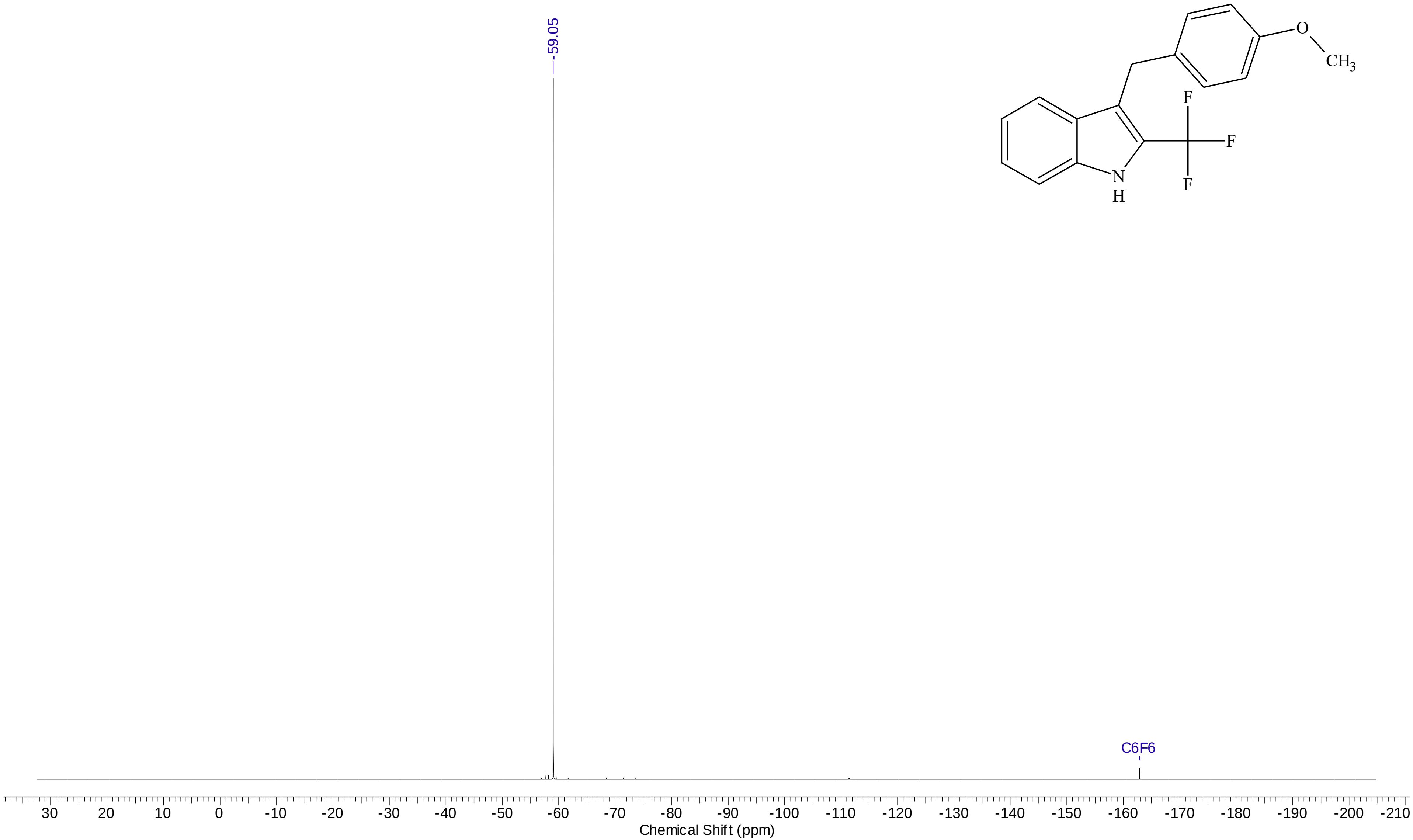

^{19}F NMR spectrum of **4g** (376.5 MHz, $CDCl_3$)

18 Jun 2020

Acquisition Time (sec)	0.6783	***Comment***	Imported from UXNMR.	***Date***	11 Oct 2019 15:41:16
File Name	C:\DOCS\OUTPUT_301\2019\10.铌? 狃黒BM-1732-1.C_002001r			***Frequency (MHz)***	100.61
Nucleus	13C	***Number of Transients***	137 ***Original Points Count*** 16384	***Points Count***	131072
Pulse Sequence	zgpg30	***Solvent***	CHLOROFORM-D	***Sweep Width (Hz)***	24154.59
Temperature (degree C)	27.000				

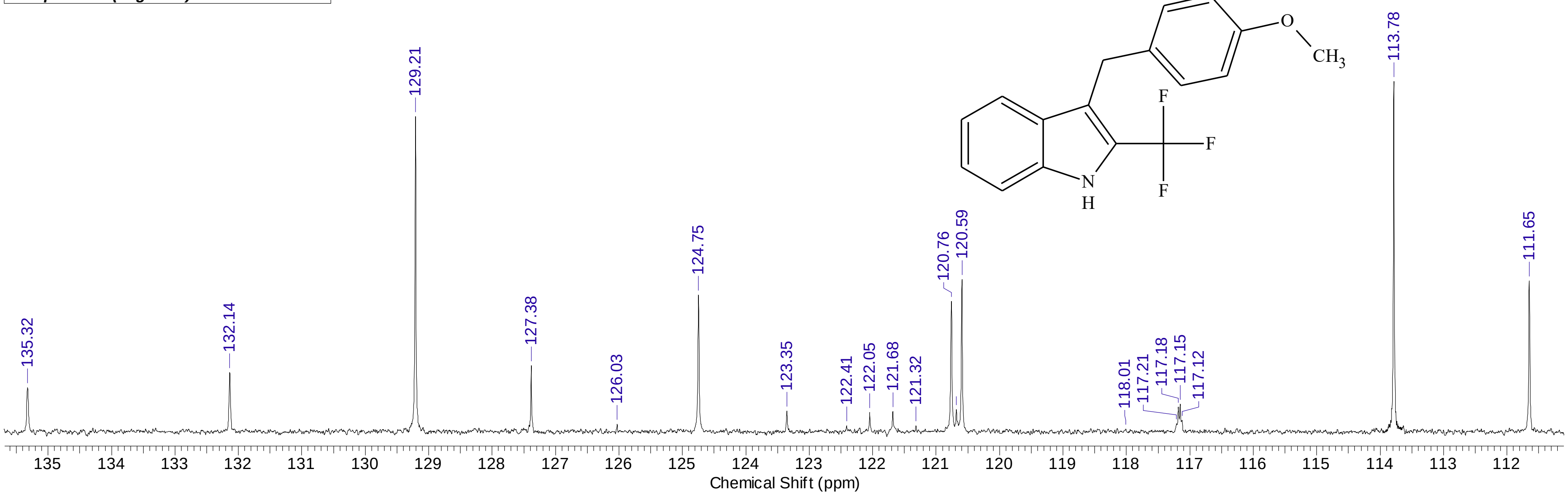

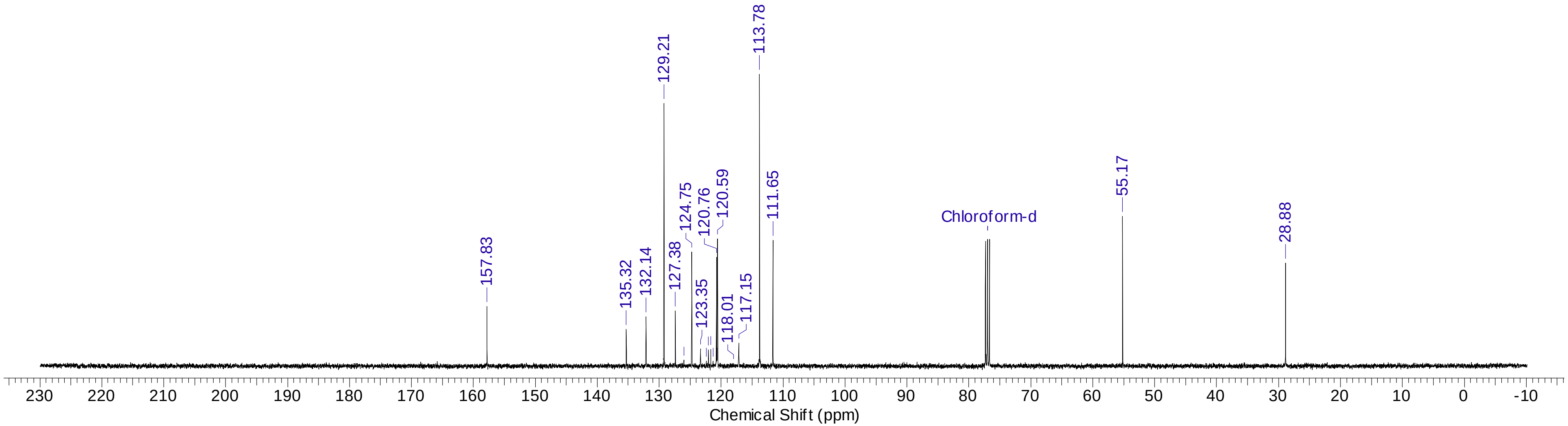

$^{13}C\{^{1}H\}$ NMR spectrum of **4g** (100.6 MHz, $CDCl_3$)

19 Jun 2020

Acquisition Time (sec)	4.0894	Comment	Imported from UXNMR.			Date	26 Nov 2019 21:03:36		
File Name	C:\DOCS\BM\bm191126\BM-1798_001001r			Frequency (MHz)	400.13	Nucleus	1H	Number of Transients	8
Original Points Count	32768	Points Count	131072	Pulse Sequence	zg30	Solvent	CHLOROFORM-D		
Sweep Width (Hz)	8012.82	Temperature (degree C)	27.000						

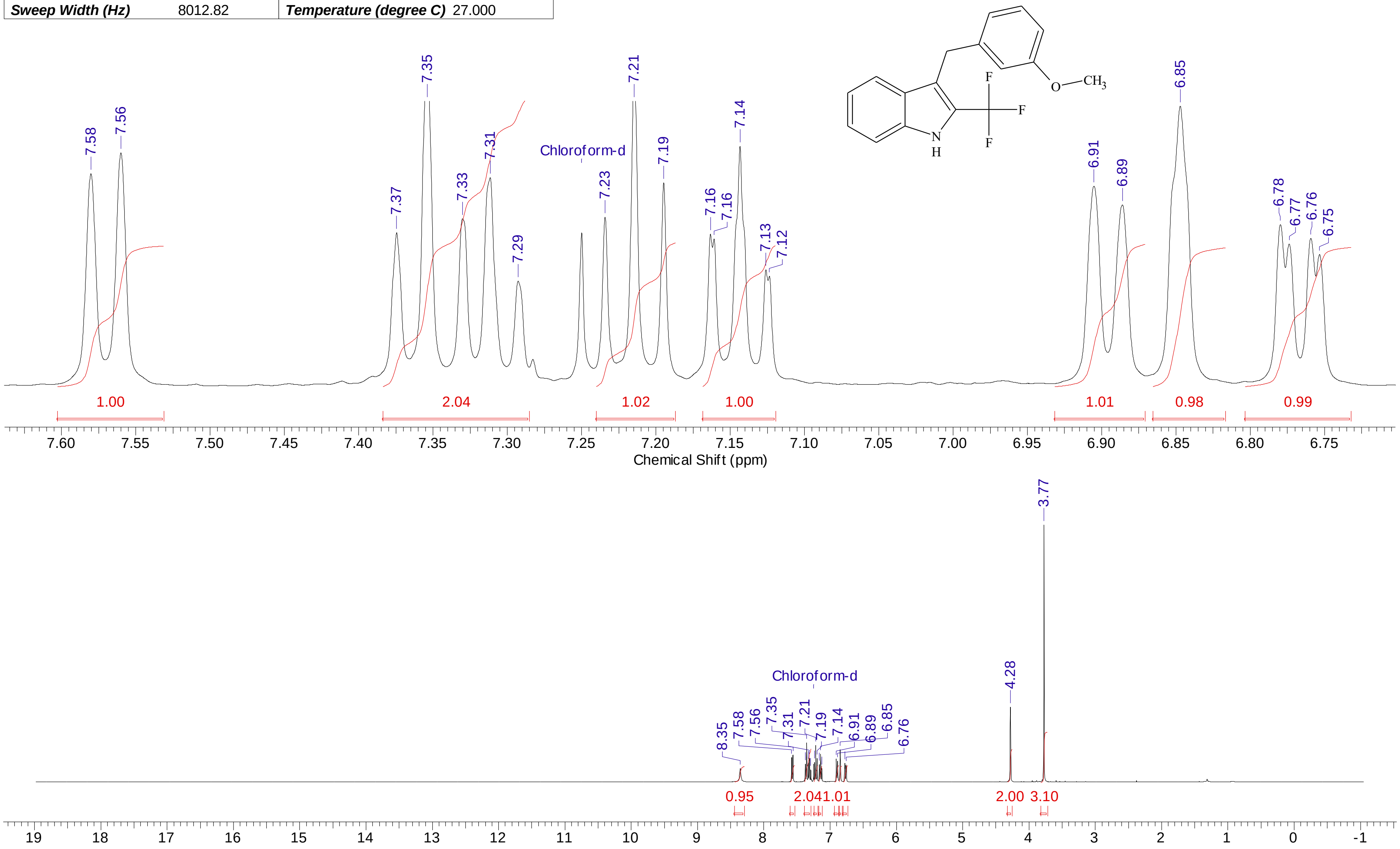

^{1}H NMR spectrum of **4h** (400.1 MHz, $CDCl_3$)

19 Jun 2020

Acquisition Time (sec)	1.0000	Comment	STANDARD FLUORINE PARAMETERS			Date	Nov 25 2019
File Name	C:\DOCS\OUTPUT_301\F19\2019.11.25\BM-1798_20191125_01\FLUORINE_01					Frequency (MHz)	376.31
Nucleus	19F	Number of Transients	16	Original Points Count	89286	Points Count	131072
Pulse Sequence	s2pul	Solvent	CHLOROFORM-D	Sweep Width (Hz)	89285.71	Temperature (degree C)	20.000

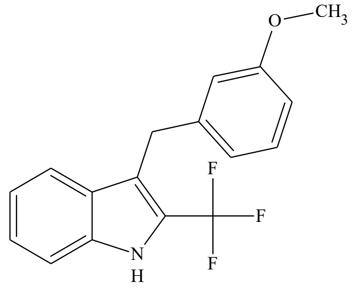

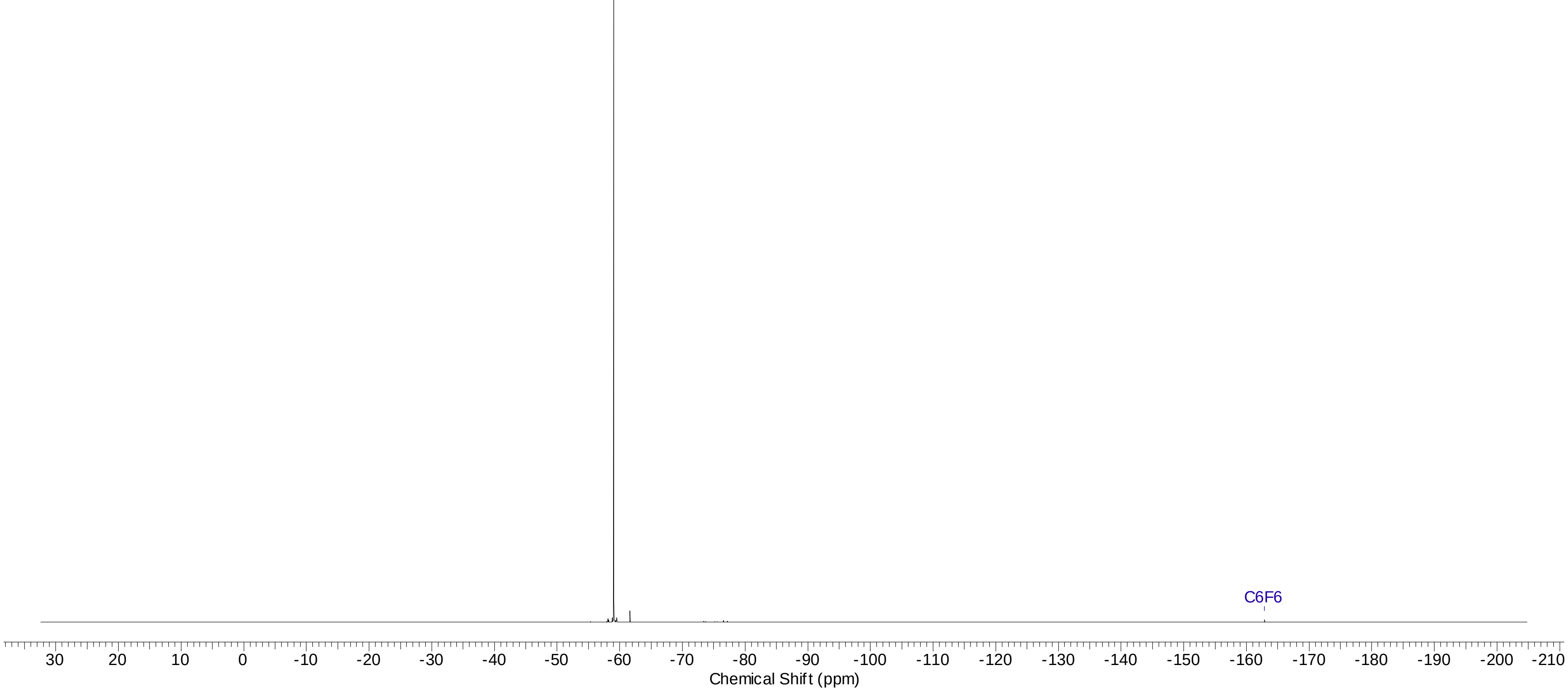

^{19}F NMR spectrum of **4h** (376.5 MHz, $CDCl_3$)

19 Jun 2020

Acquisition Time (sec)	0.6783	***Comment***	Imported from UXNMR.			***Date***	26 Nov 2019 21:09:40
File Name	C:\DOCS\BM\bm191126\BM-1798_002001r			***Frequency (MHz)***	100.61	***Nucleus***	13C
Number of Transients	136	***Original Points Count***	16384	***Points Count***	131072	***Pulse Sequence***	zgpg30
Solvent	CHLOROFORM-D			***Sweep Width (Hz)***	24154.59	***Temperature (degree C)***	27.000

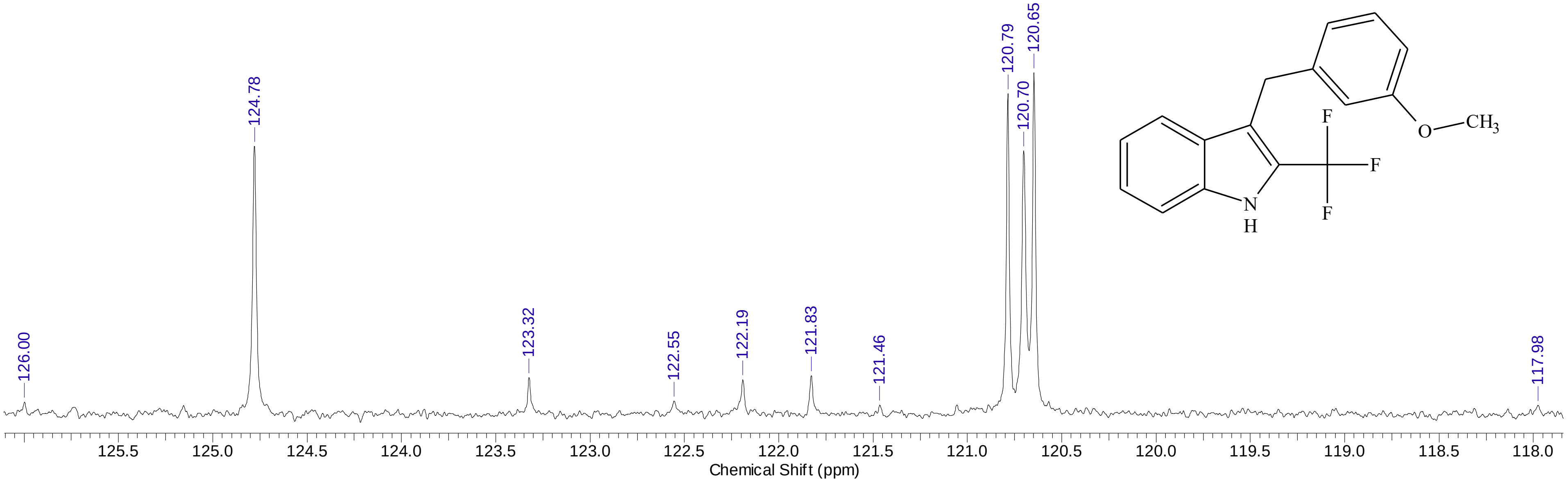

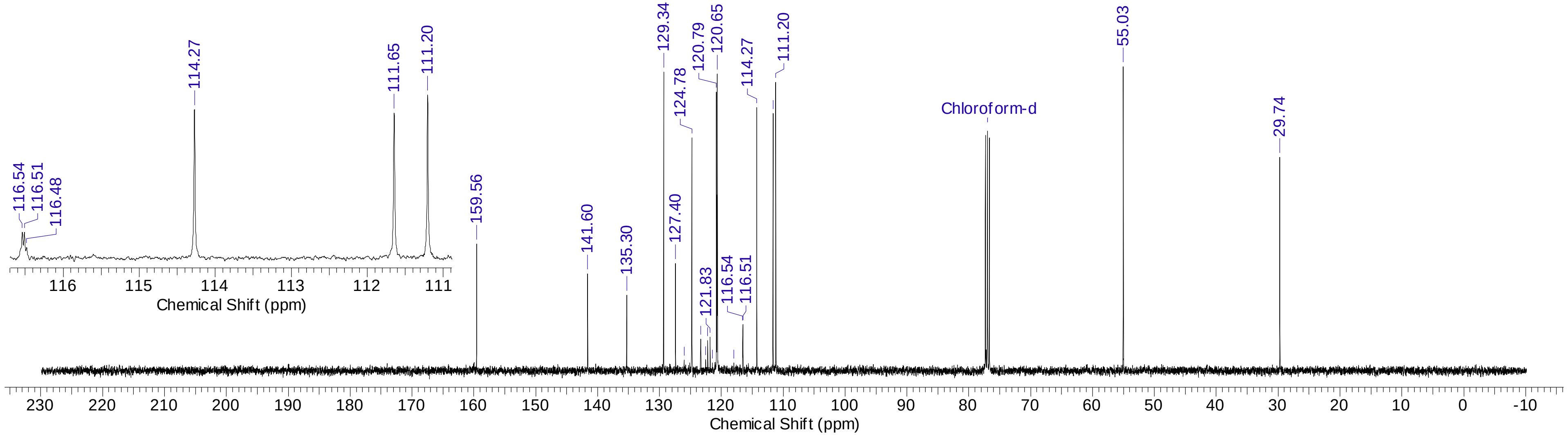

$^{13}C\{^1H\}$ NMR spectrum of **4h** (100.6 MHz, $CDCl_3$)

19 Jun 2020

Acquisition Time (sec)	4.0894	***Comment***	Imported from UXNMR.			***Date***	27 Nov 2019 17:53:40
File Name	C:\DOCS\OUTPUT_301\2019\11.胚 犴黒BM-1799.H_001001r					***Frequency (MHz)***	400.13
Nucleus	1H	***Number of Transients***	4	***Original Points Count***	32768	***Points Count***	131072
Pulse Sequence	zg30	***Solvent***	DMSO-D6	***Sweep Width (Hz)***	8012.82	***Temperature (degree C)***	27.000

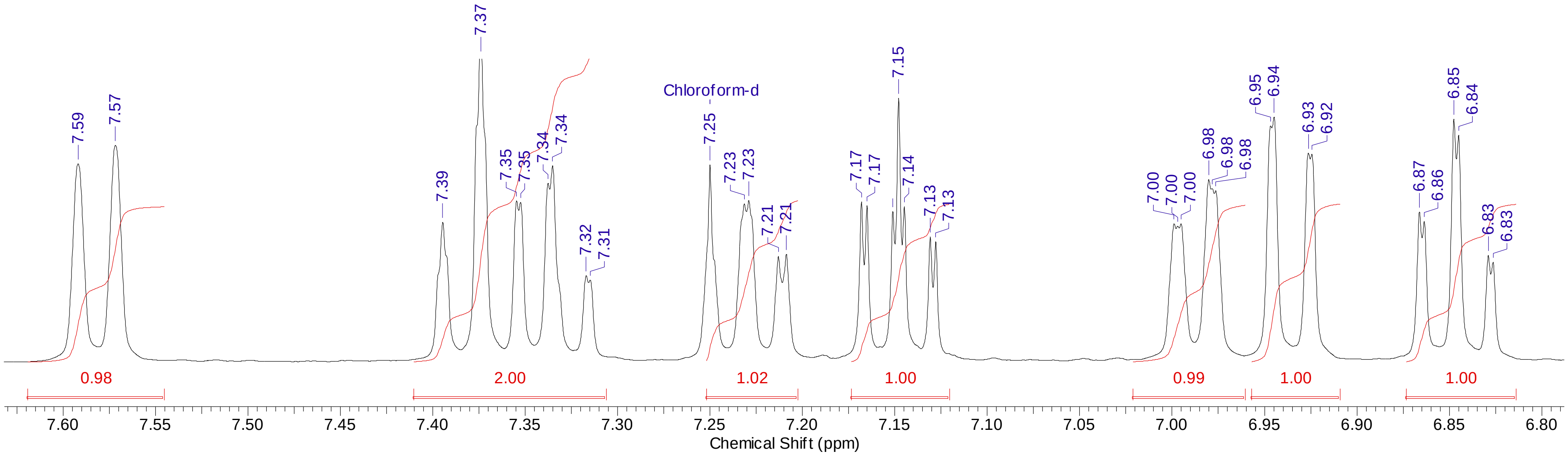

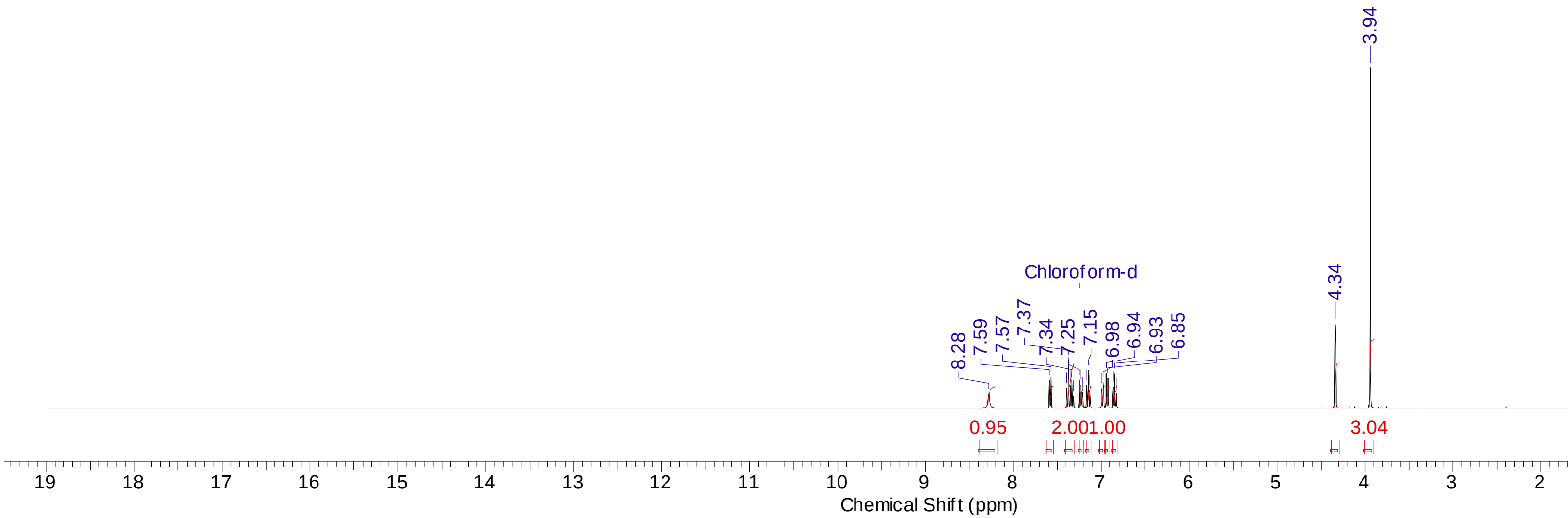

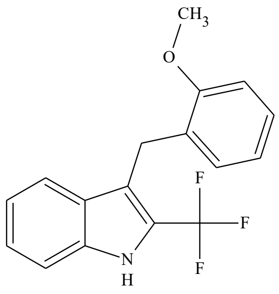

^1H NMR spectrum of **4i** (400.1 MHz, $CDCl_3$)

19 Jun 2020

Acquisition Time (sec)	1.0000	***Date***	Nov 25 2019	***File Name***	C:\DOCS\OUTPUT_301\F19\2019.11.25\BM-1799_20191125_01\FLUORINE_01		
Frequency (MHz)	376.31	***Nucleus***	19F	***Number of Transients***	16	***Original Points Count***	89286
Points Count	131072	***Pulse Sequence***	s2pul	***Solvent***	CHLOROFORM-D		
Sweep Width (Hz)	89285.71	***Temperature (degree C)***	20.000				

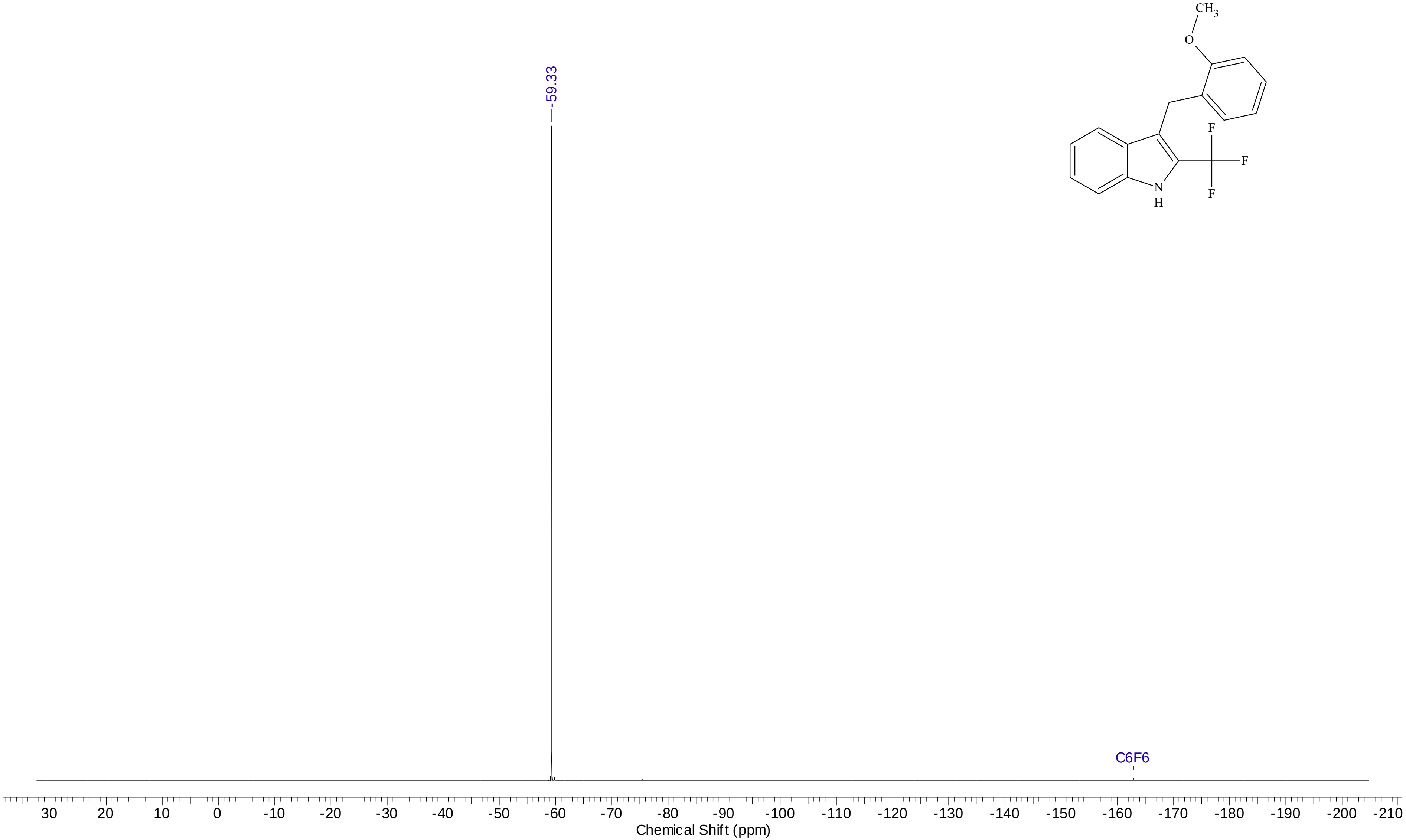

^{19}F NMR spectrum of **4i** (376.5 MHz, $CDCl_3$)

19 Jun 2020

**_Acquisition Time (sec)_**	0.6783	**_Comment_**	Imported from UXNMR.			**_Date_**	27 Nov 2019 17:57:52
**_File Name_**	C:\DOCS\OUTPUT_301\2019\11.䏊 狃黒BM-1799.C_002001r			**_Frequency (MHz)_**	100.61	**_Nucleus_**	13C
**_Number of Transients_**	98	**_Original Points Count_**	16384	**_Points Count_**	131072	**_Pulse Sequence_**	zgpg30
**_Solvent_**	CHLOROFORM-D			**_Sweep Width (Hz)_**	24154.59	**_Temperature (degree C)_**	27.000

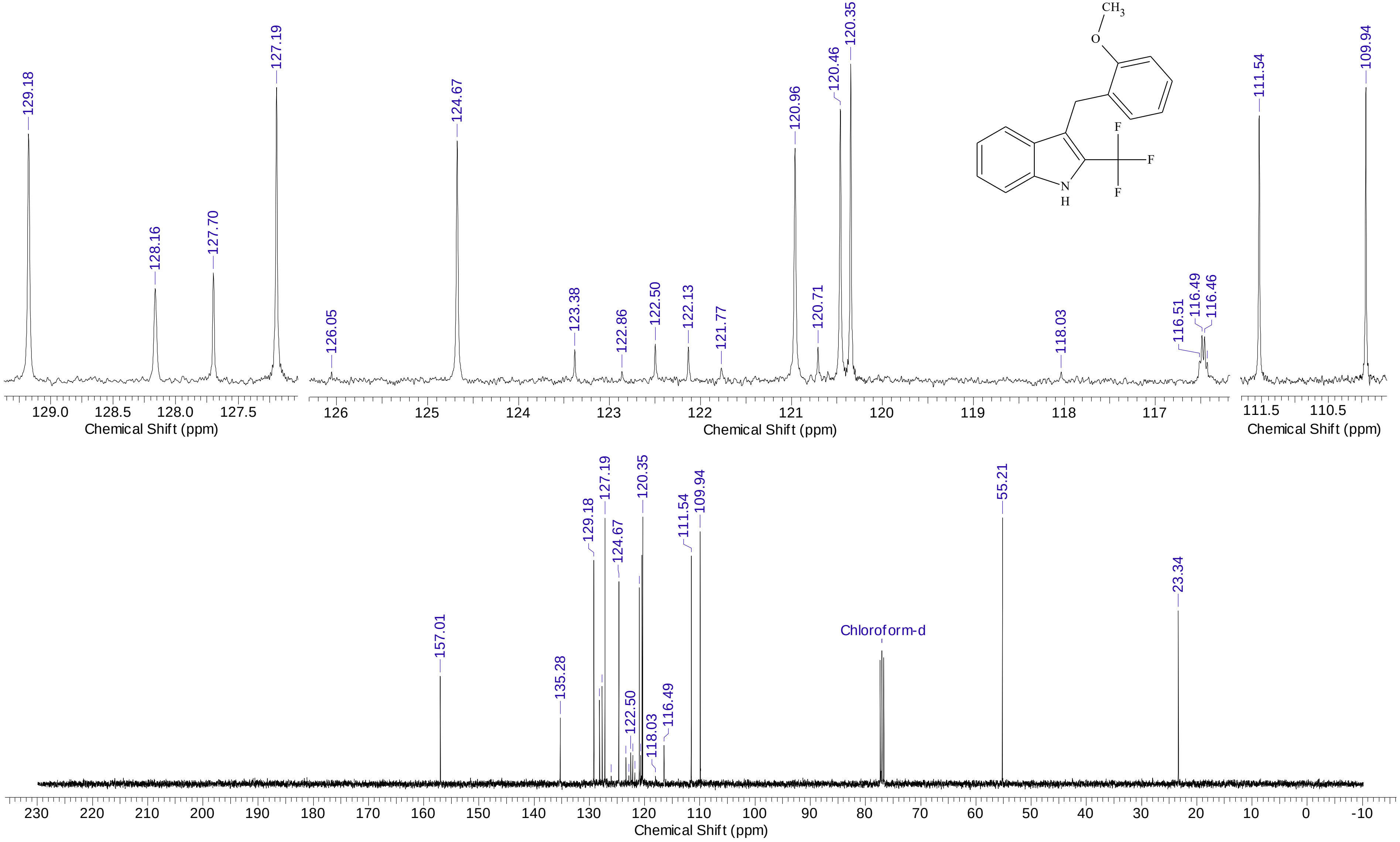

$^{13}C\{^1H\}$ NMR spectrum of **4i** (100.6 MHz, $CDCl_3$)

19 Jun 2020

Acquisition Time (sec)	4.0894	Comment	Imported from UXNMR.	Date	14 Dec 2019 13:50:26		
File Name	C:\DOCS\BM\BM-1825-p\BM-1825-p_001001r	Frequency (MHz)	400.13	Nucleus	1H	Number of Transients	8
Original Points Count	32768	Points Count	131072	Pulse Sequence	zg30	Solvent	ACETONITRILE-D3
Sweep Width (Hz)	8012.82	Temperature (degree C)	27.000				

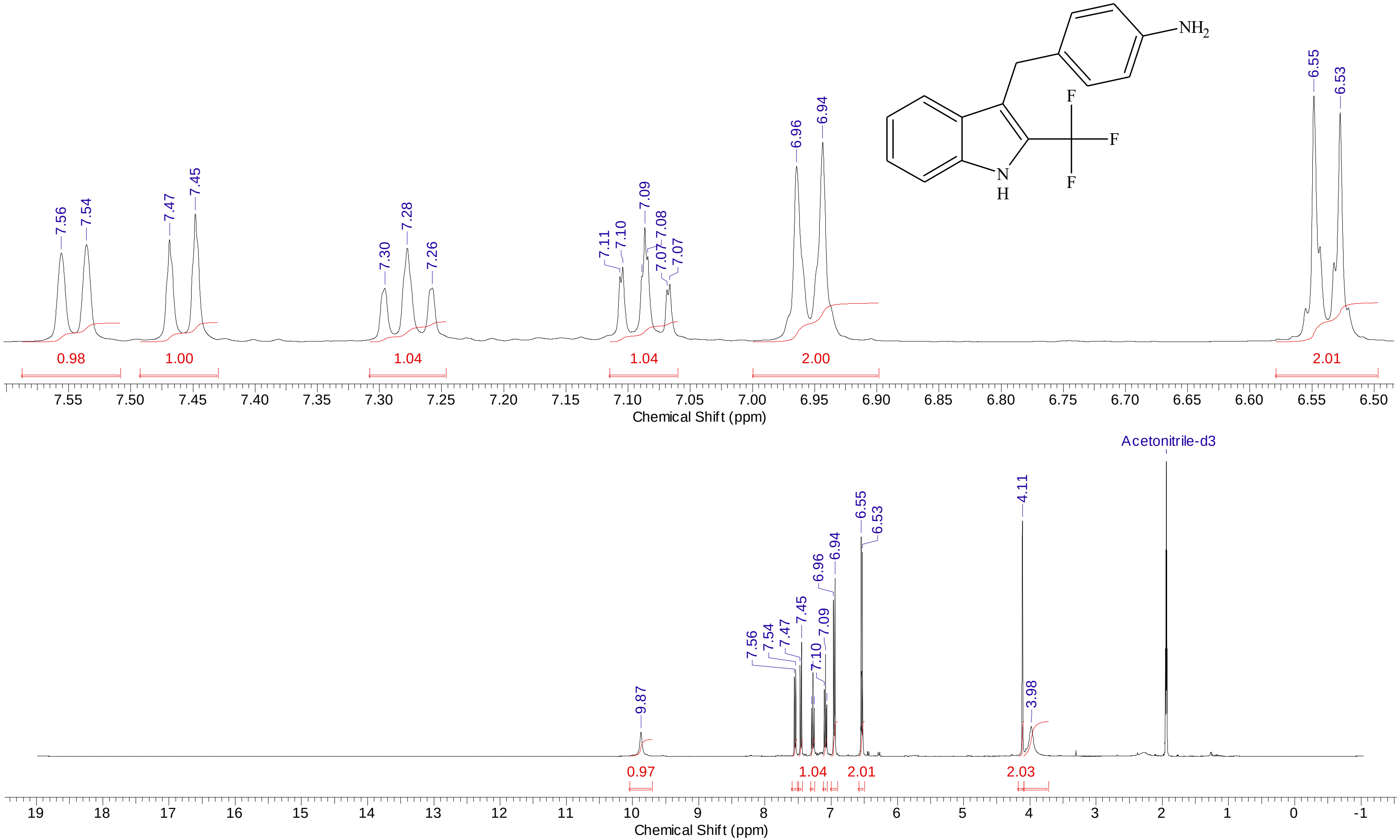

^{1}H NMR spectrum of **4j** (400.1 MHz, $CDCl_3$)

19 Jun 2020

Acquisition Time (sec)	2.0000	***Date***	Dec 16 2019	***File Name***	C:\DOCS\OUTPUT_301\F19\2019.12.16\BM-1825-p_20191216_01\FLUORINE_01		
Frequency (MHz)	376.31	***Nucleus***	19F	***Number of Transients***	16	***Original Points Count***	178571
Points Count	262144	***Pulse Sequence***	s2pul	***Solvent***	ACETONITRILE-D3		
Sweep Width (Hz)	89285.71	***Temperature (degree C)***	30.000				

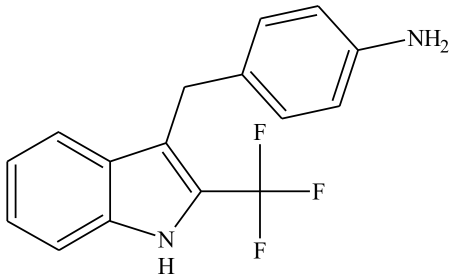

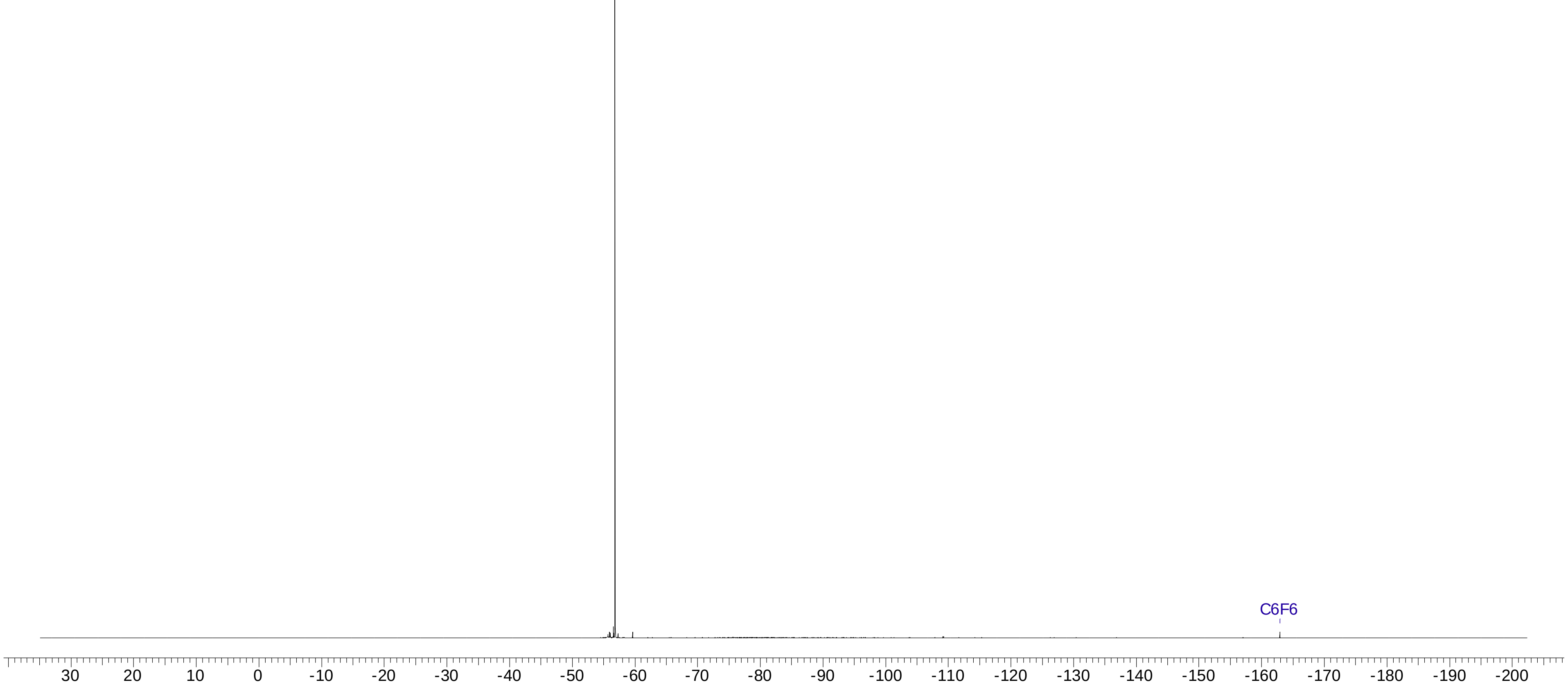

^{19}F NMR spectrum of **4j** (376.5 MHz $CDCl_3$)

19 Jun 2020

Acquisition Time (sec)	0.6783	***Comment***	Imported from UXNMR.			***Date***	12 Dec 2019 13:00:36
File Name	C:\DOCS\OUTPUT_301\2019\12.溴赅狃黒BM-1825.C_002001r					***Frequency (MHz)***	100.61
Nucleus	13C	***Number of Transients***	233	***Original Points Count***	16384	***Points Count***	131072
Pulse Sequence	zgpg30	***Solvent***	CHLOROFORM-D			***Sweep Width (Hz)***	24154.59
Temperature (degree C)	27.000						

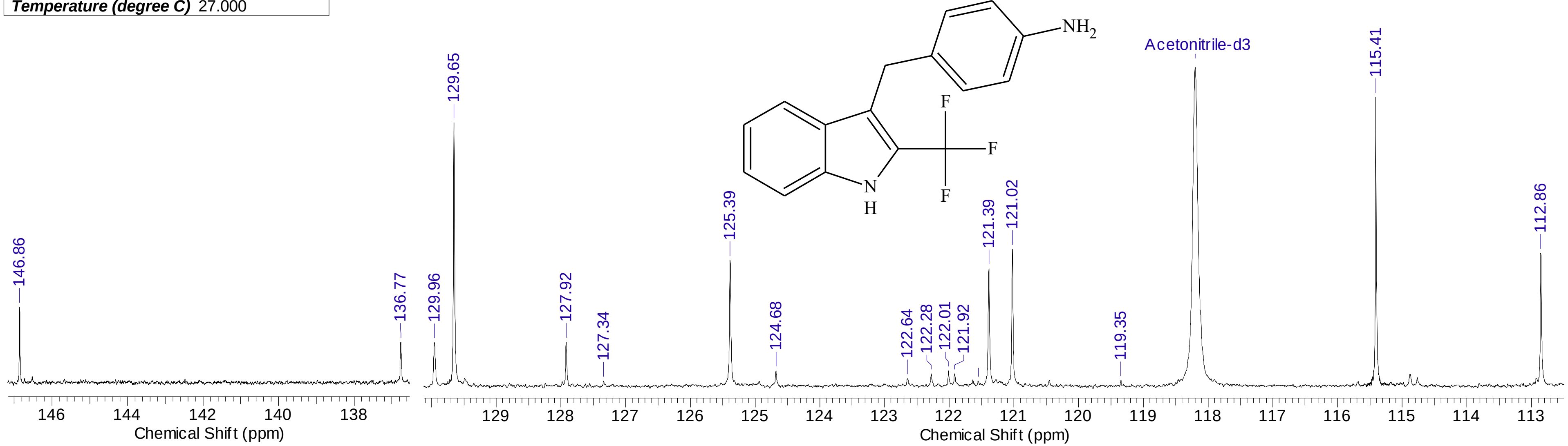

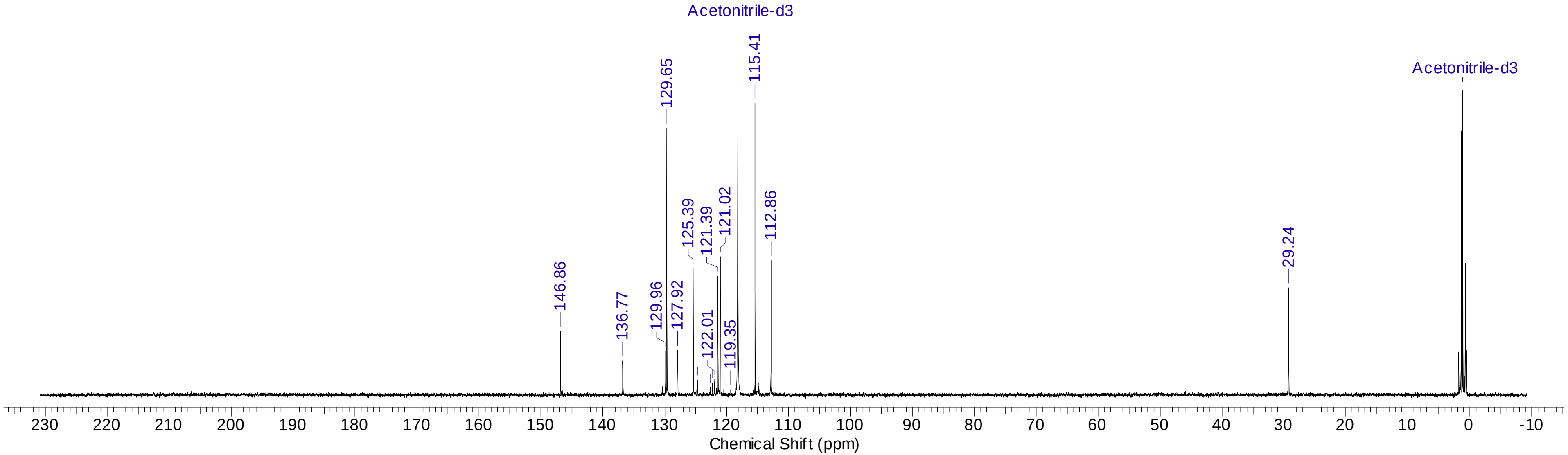

$^{13}C\{^{1}H\}$ NMR spectrum of **4j** (100.6 MHz, CD_3CN)

19 Jun 2020

Acquisition Time (sec)	4.0894	***Comment***	Imported from UXNMR.	***Date***	29 Nov 2019 15:39:34
File Name	C:\DOCS\OUTPUT_301\2019\11.眍 狃黒BM-1804.H_001001r			***Frequency (MHz)***	400.13
Nucleus	1H	***Number of Transients***	4 ***Original Points Count*** 32768	***Points Count***	131072
Pulse Sequence	zg30	***Solvent***	CHLOROFORM-D	***Sweep Width (Hz)***	8012.82
Temperature (degree C)	27.000				

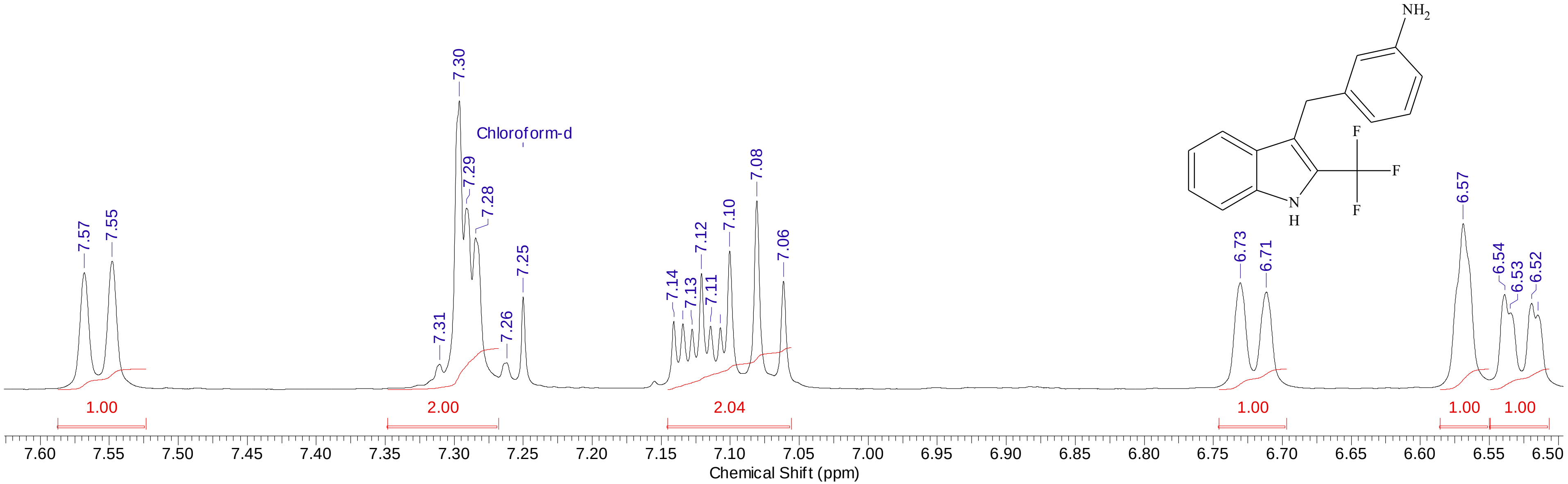

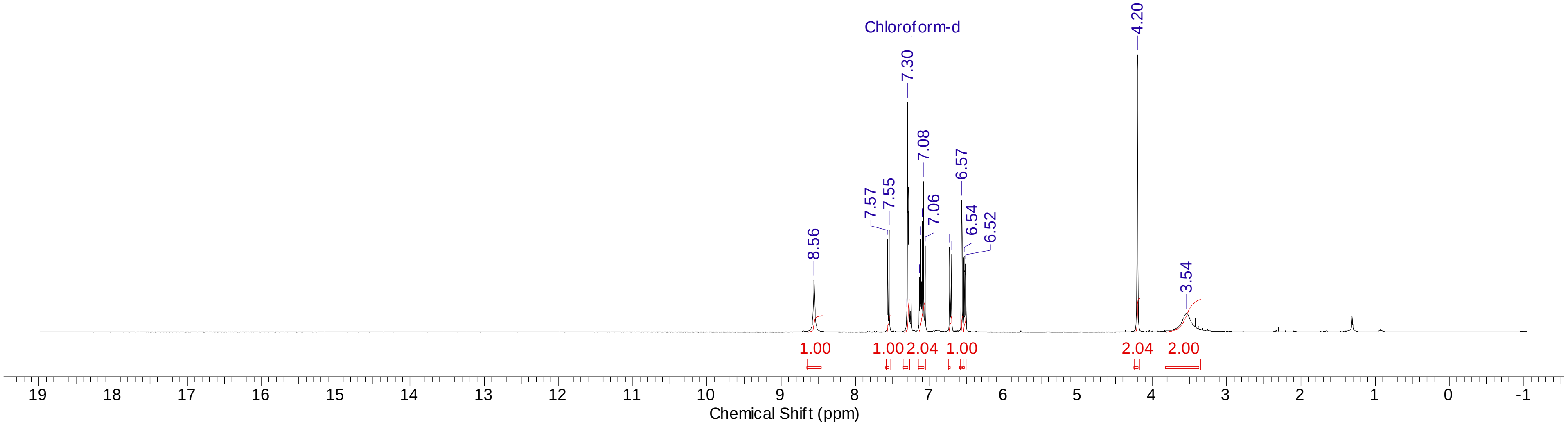

^1H NMR spectrum of **4k** (400.1 MHz, $CDCl_3$)

19 Jun 2020

Acquisition Time (sec)	1.0000	***Date***	Nov 29 2019	***File Name***	C:\DOCS\OUTPUT_301\F19\2019.11.29\BM-1804_20191129_01\FLUORINE_01		
Frequency (MHz)	376.31	***Nucleus***	19F	***Number of Transients***	16	***Original Points Count***	89286
Points Count	131072	***Pulse Sequence***	s2pul	***Solvent***	CHLOROFORM-D		
Sweep Width (Hz)	89285.71	***Temperature (degree C)***	30.000				

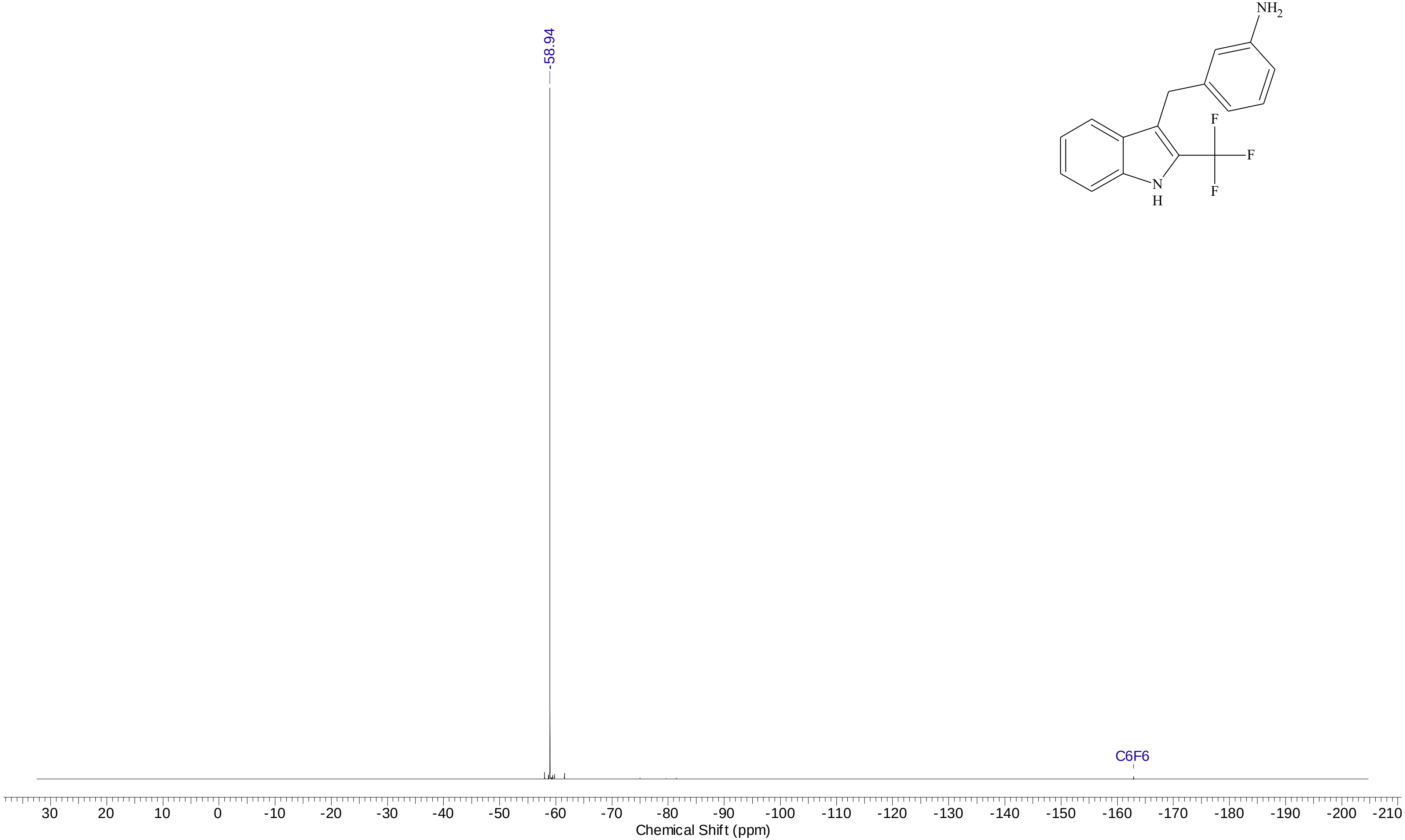

^{19}F NMR spectrum of **4k** (376.5 MHz, $CDCl_3$)

Acquisition Time (sec)	0.6783	***Comment***	Imported from UXNMR.	***Date***	29 Nov 2019 15:46:36
File Name	C:\DOCS\OUTPUT_301\2019\11.朊 狃黒BM-1804.C_002001r	***Frequency (MHz)***	100.61	***Nucleus***	13C
Number of Transients	105	***Original Points Count*** 16384	***Points Count*** 131072	***Pulse Sequence***	zgpg30
Solvent	CHLOROFORM-D		***Sweep Width (Hz)*** 24154.59	***Temperature (degree C)***	27.000

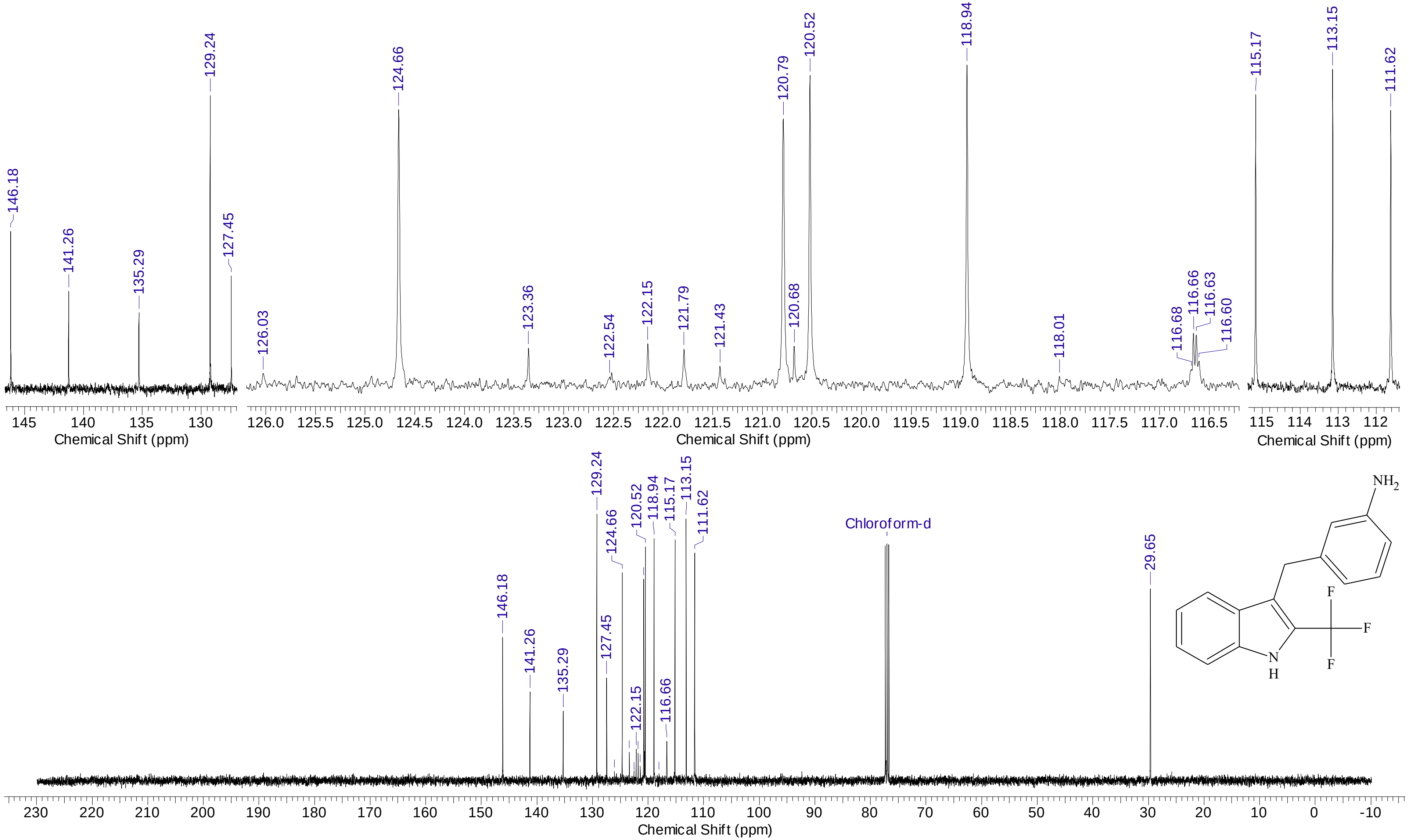

$^{13}C\{^1H\}$ NMR spectrum of **4k** (100.6 MHz, $CDCl_3$)

3 Jul 2021

Acquisition Time (sec)	4.0894	Comment	Imported from UXNMR.			Date	14 Jul 2020 14:41:46
File Name	C:\DOCS\OUTPUT_301\2020\07.棹 朦\BM-1893-2.H_001001r					Frequency (MHz)	400.13
Nucleus	1H	Number of Transients	4	Original Points Count	32768	Points Count	131072
Pulse Sequence	zg30	Solvent	CHLOROFORM-D			Sweep Width (Hz)	8012.82
Temperature (degree C)	27.000						

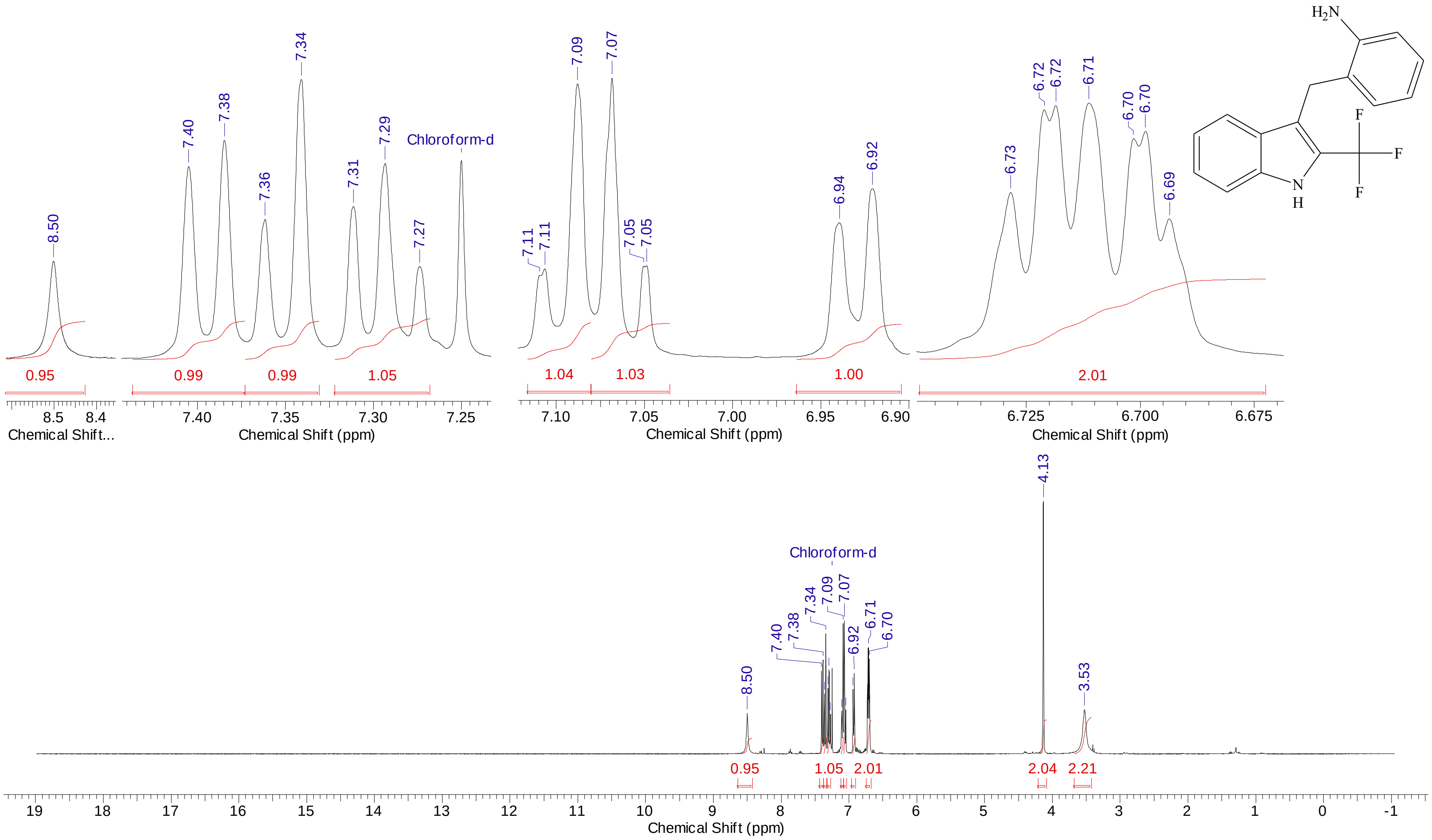

^{1}H NMR spectrum of **4l** (400.1 MHz, $CDCl_3$)

3 Jul 2021

Acquisition Time (sec)	1.7433	***Comment***	Imported from UXNMR.			***Date***	14 Jul 2020 20:54:04
File Name	C:\DOCS\BM\2020.07.14\BM-1893-2_005001r			***Frequency (MHz)***	376.50	***Nucleus***	19F
Number of Transients	16	***Original Points Count***	131072	***Points Count***	262144	***Pulse Sequence***	zgflqn
Solvent	CHLOROFORM-D			***Sweep Width (Hz)***	75187.97	***Temperature (degree C)***	27.000

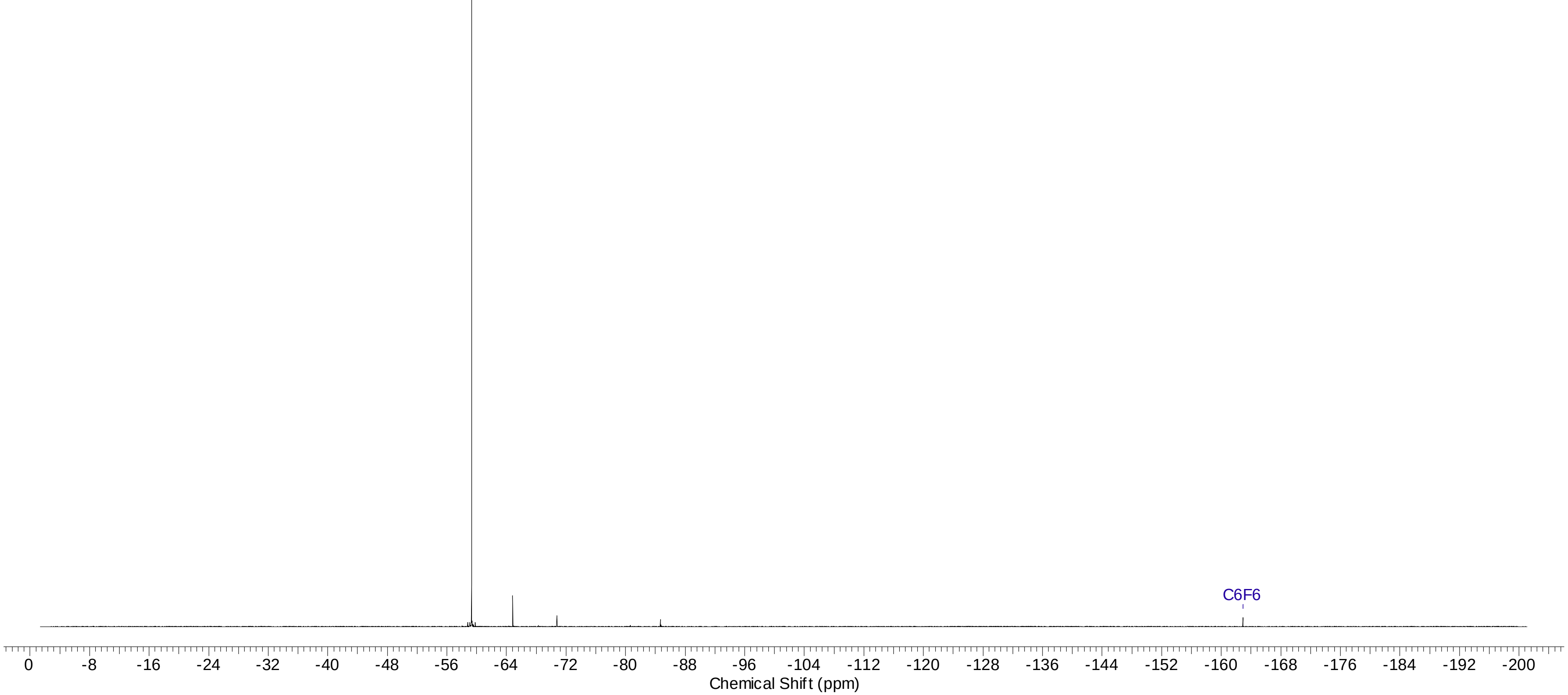

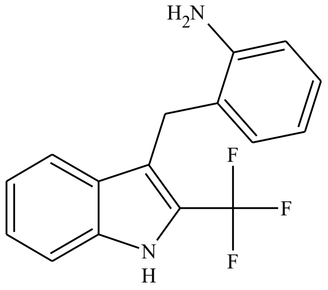

^{19}F NMR spectrum of **4l** (376.5 MHz, $CDCl_3$)

3 Jul 2021

Acquisition Time (sec)	0.6783	***Comment***	Imported from UXNMR.	***Date***	14 Jul 2020 14:57:32
File Name	C:\DOCS\OUTPUT_301\2020\07.棹 朦\BM-1893-2.C_002001r	***Frequency (MHz)***	100.61	***Nucleus***	13C
Number of Transients	353	***Original Points Count*** 16384 / ***Points Count***	131072	***Pulse Sequence***	zgpg30
Solvent	CHLOROFORM-D	***Sweep Width (Hz)***	24154.59	***Temperature (degree C)***	27.000

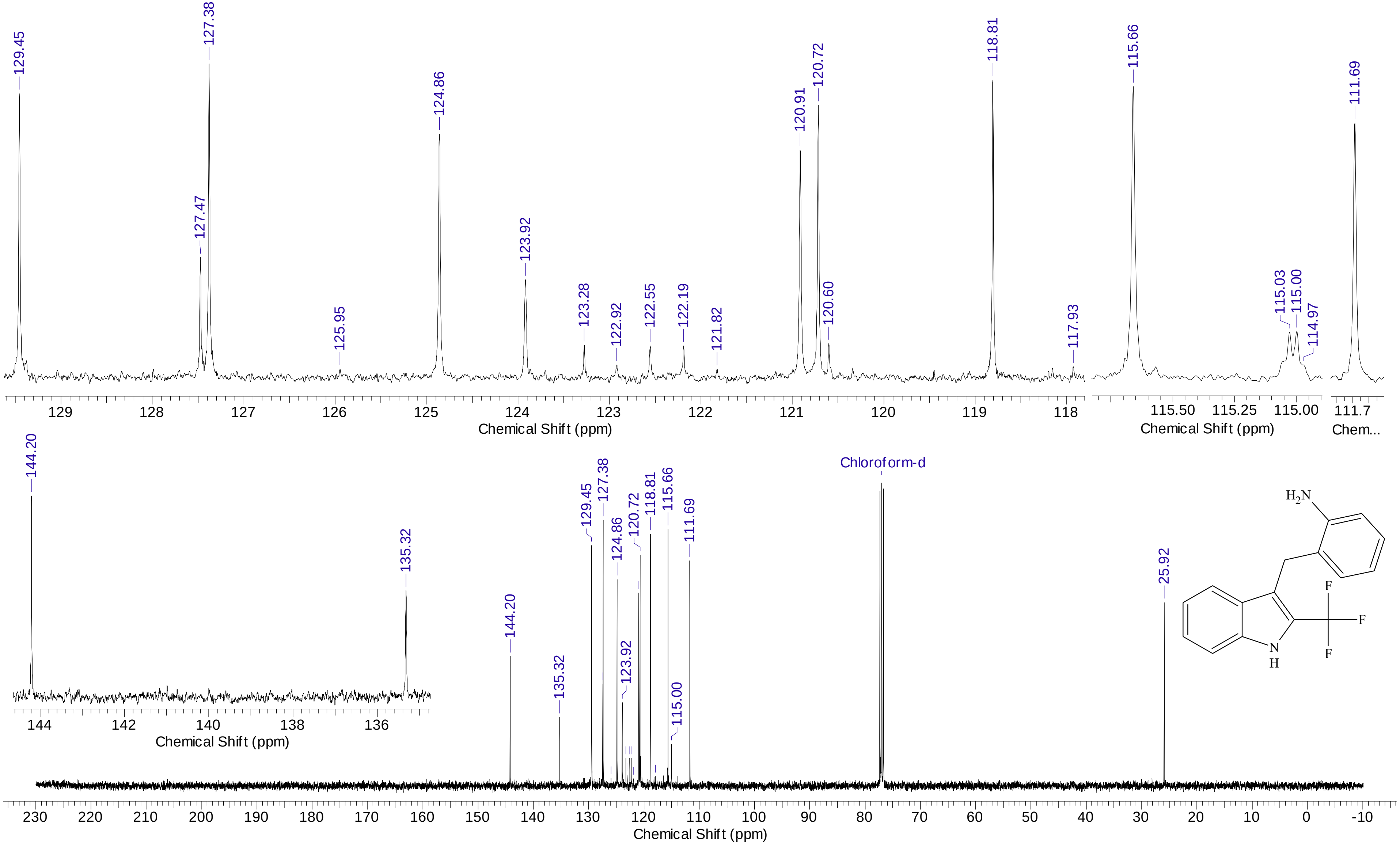

$^{13}C\{^1H\}$ NMR spectrum of **4l** (100.6 MHz, $CDCl_3$)

19 Jun 2020

Acquisition Time (sec)	4.0894	***Comment***	Imported from UXNMR.			***Date***	26 Nov 2019 12:36:00
File Name	C:\DOCS\OUTPUT_301\2019\11.胒 狃黒BM-1800.H_001001r					***Frequency (MHz)***	400.13
Nucleus	1H	***Number of Transients***	4	***Original Points Count***	32768	***Points Count***	131072
Pulse Sequence	zg30	***Solvent***	DMSO-D6	***Sweep Width (Hz)***	8012.82	***Temperature (degree C)***	27.000

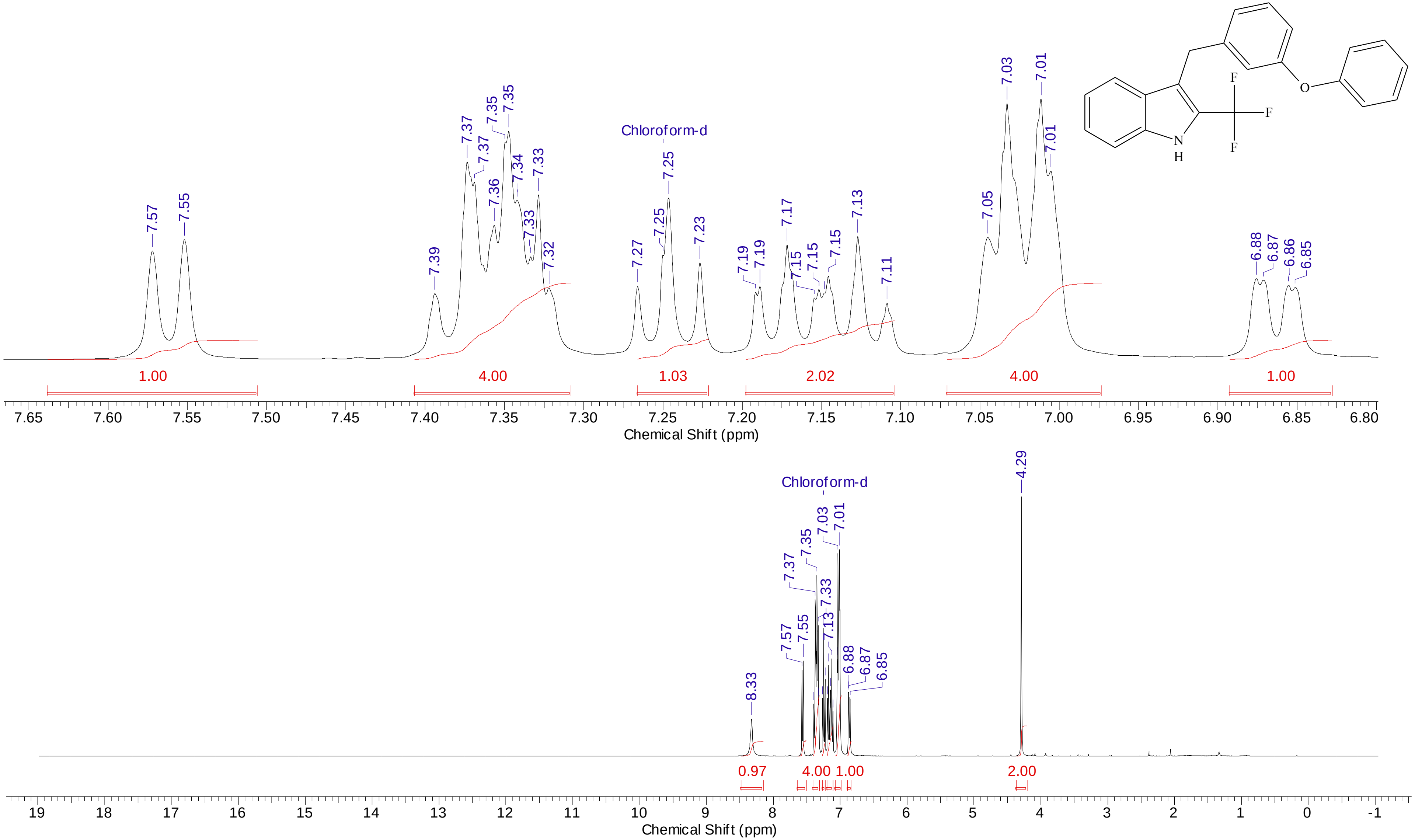

^{1}H NMR spectrum of **4m** (400.1 MHz, $CDCl_3$)

Acquisition Time (sec)	1.0000	Date	Nov 25 2019	File Name	C:\DOCS\OUTPUT_301\F19\2019.11.25\BM-1800_20191125_01\FLUORINE_01		
Frequency (MHz)	376.31	Nucleus	19F	Number of Transients	16	Original Points Count	89286
Points Count	131072	Pulse Sequence	s2pul	Solvent	CHLOROFORM-D		
Sweep Width (Hz)	89285.71	Temperature (degree C)	20.000				

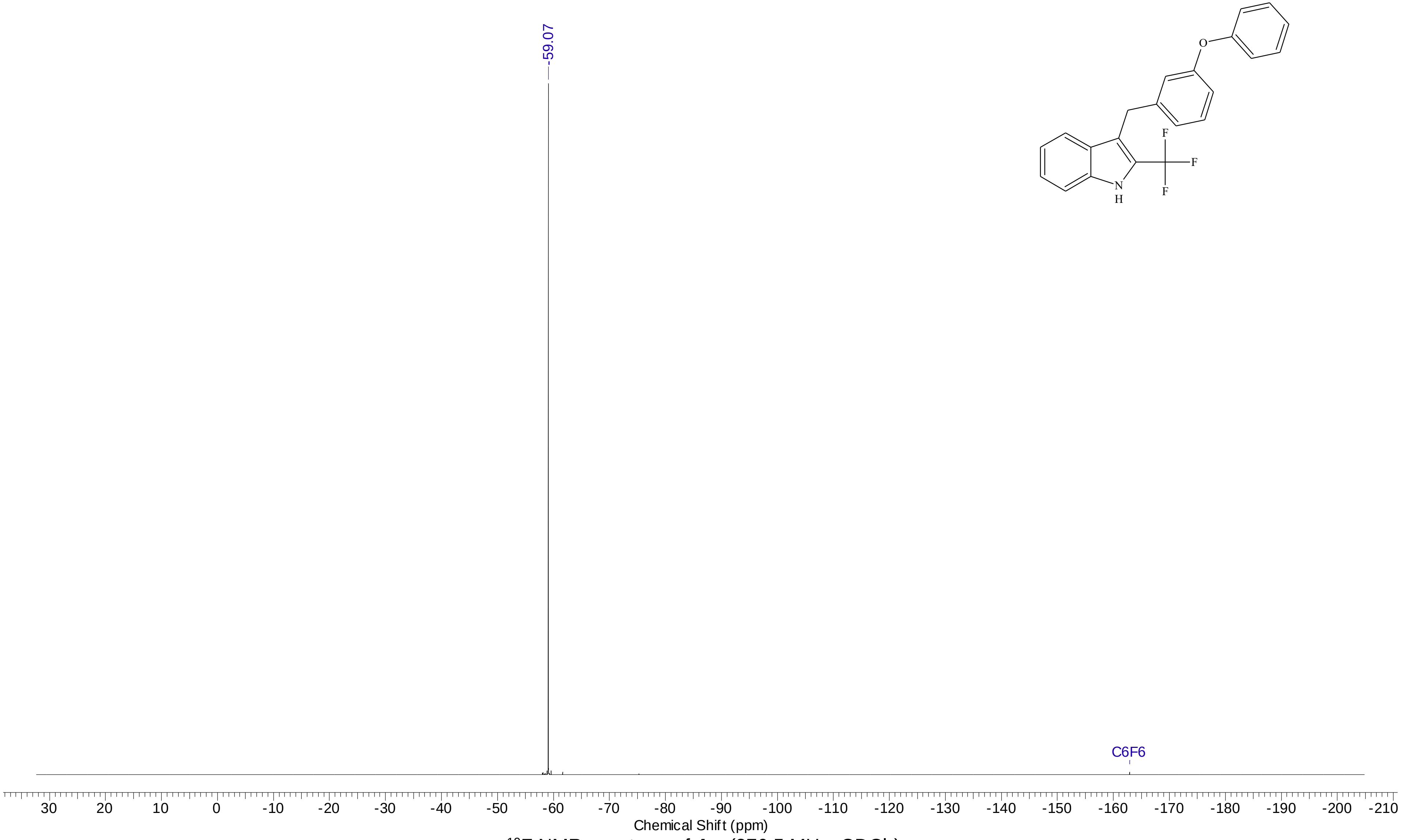

^{19}F NMR spectrum of **4m** (376.5 MHz, $CDCl_3$)

19 Jun 2020

Acquisition Time (sec)	0.6783	Comment	Imported from UXNMR.			Date	26 Nov 2019 09:38:08
File Name	C:\DOCS\OUTPUT_301\2019\11.肶 狃黒BM-1800.C\BM-1800.C_002000fid					Frequency (MHz)	100.62
Nucleus	13C	Number of Transients	129	Original Points Count	16384	Points Count	131072
Pulse Sequence	zgpg30	Solvent	CHLOROFORM-D			Sweep Width (Hz)	24154.59
Temperature (degree C)	27.000						

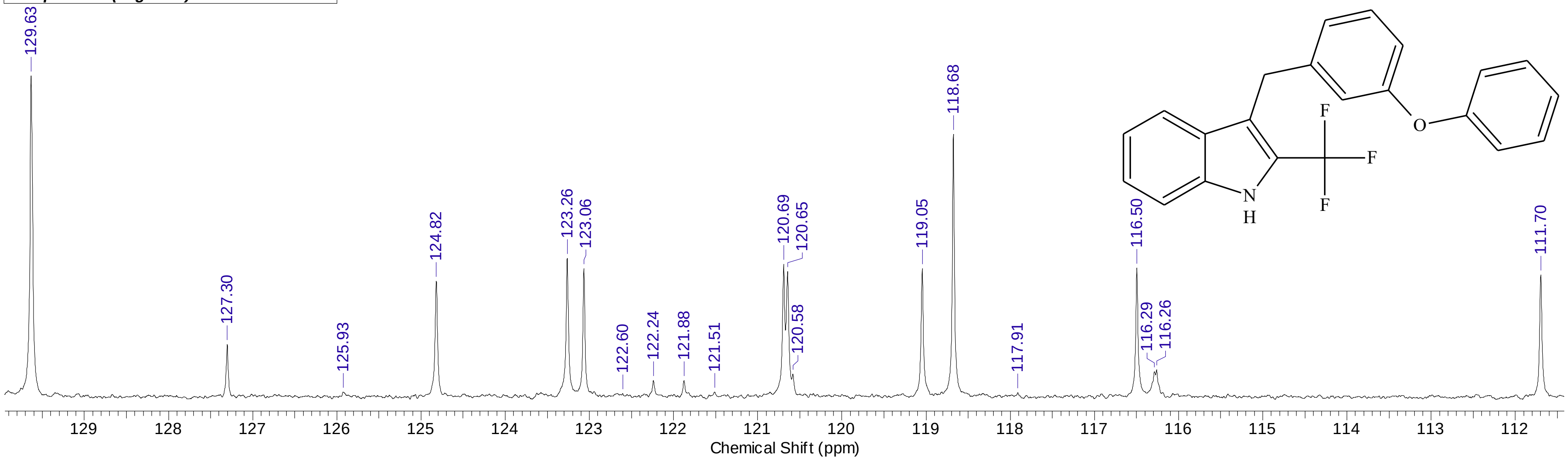

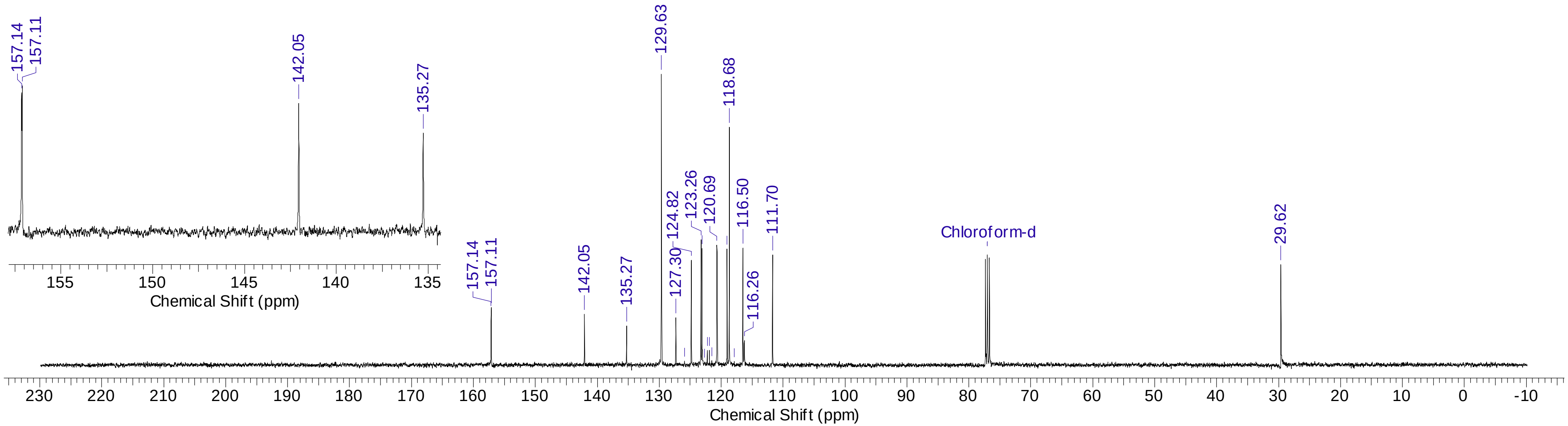

$^{13}C\{^{1}H\}$ NMR spectrum of **4m** (100.6 MHz, $CDCl_3$)

19 Jun 2020

Acquisition Time (sec)	1.3664	***Comment***	Imported from UXNMR.	***Date***	30 Nov 2019 23:12:52		
File Name	C:\DOCS\BM\BM-1800\BM-1800_004001r	***Frequency (MHz)***	100.61	***Nucleus***	13C	***Number of Transients***	64
Original Points Count	32768	***Points Count***	131072	***Pulse Sequence***	jmod	***Solvent***	CHLOROFORM-D
Sweep Width (Hz)	23980.81	***Temperature (degree C)***	27.000				

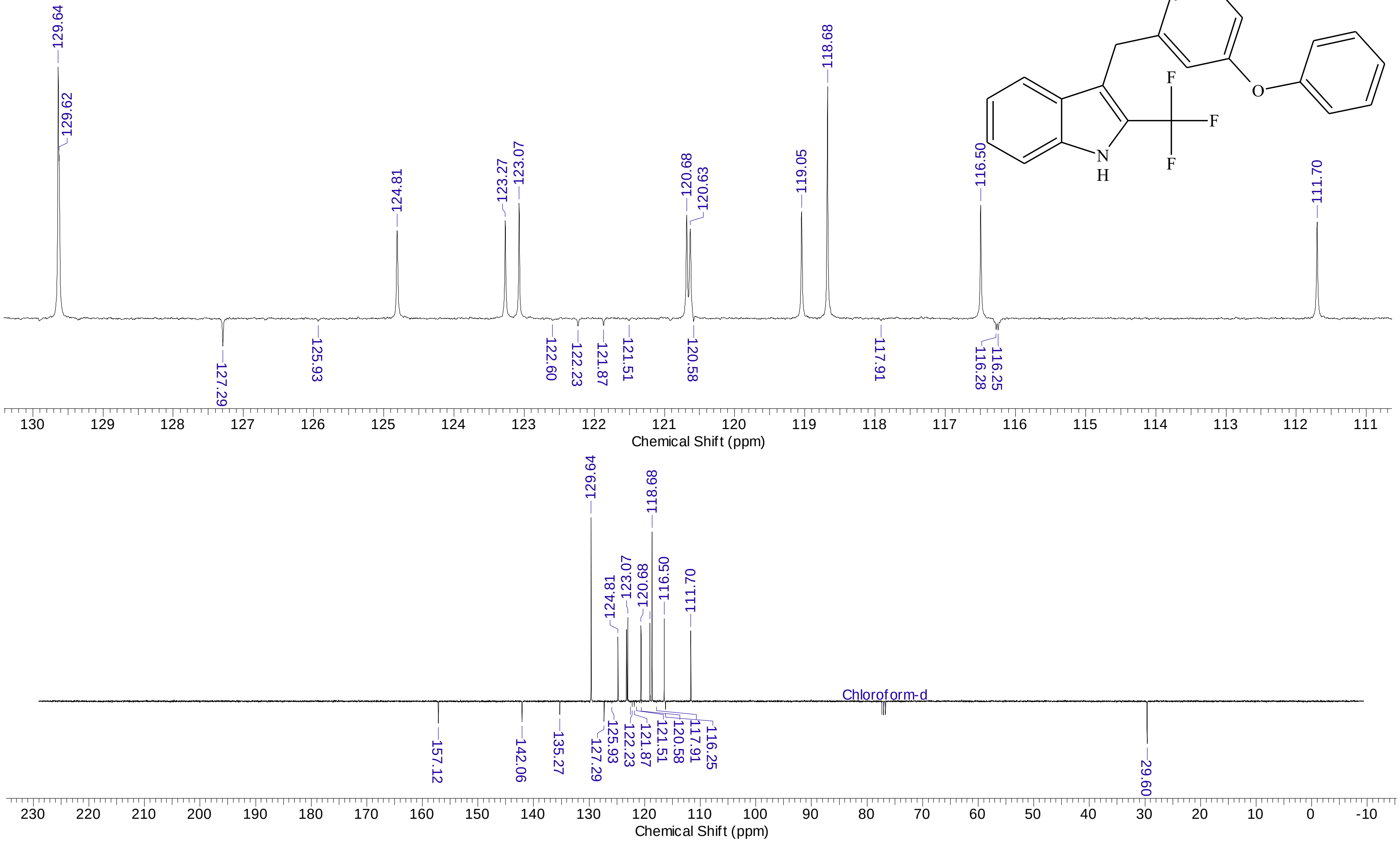

$^{13}C\{^{1}H\}$ APT NMR spectrum of **4m** (100.6 MHz, $CDCl_3$)

19 Jun 2020

Acquisition Time (sec)	4.0894	***Comment***	Imported from UXNMR.			***Date***	12 Dec 2019 12:19:10
File Name	C:\DOCS\OUTPUT_301\2019\12.溴贬狃黒BM-1824-1.H_001001r					***Frequency (MHz)***	400.13
Nucleus	1H	***Number of Transients***	4	***Original Points Count***	32768	***Points Count***	131072
Pulse Sequence	zg30	***Solvent***	CHLOROFORM-D			***Sweep Width (Hz)***	8012.82
Temperature (degree C)	27.000						

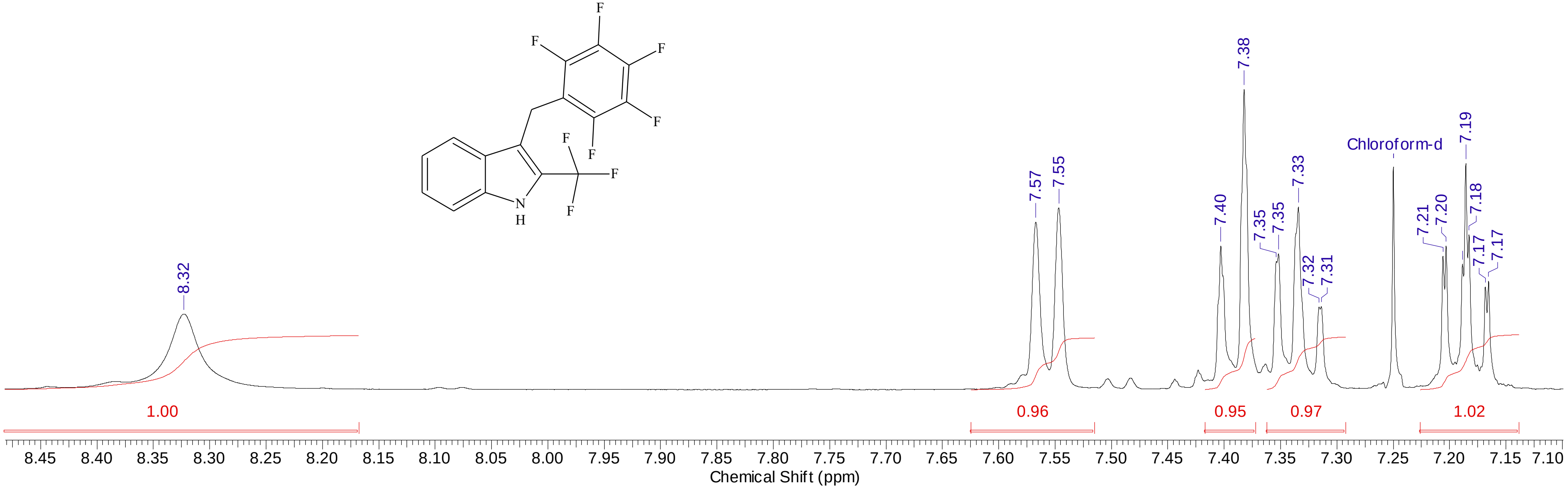

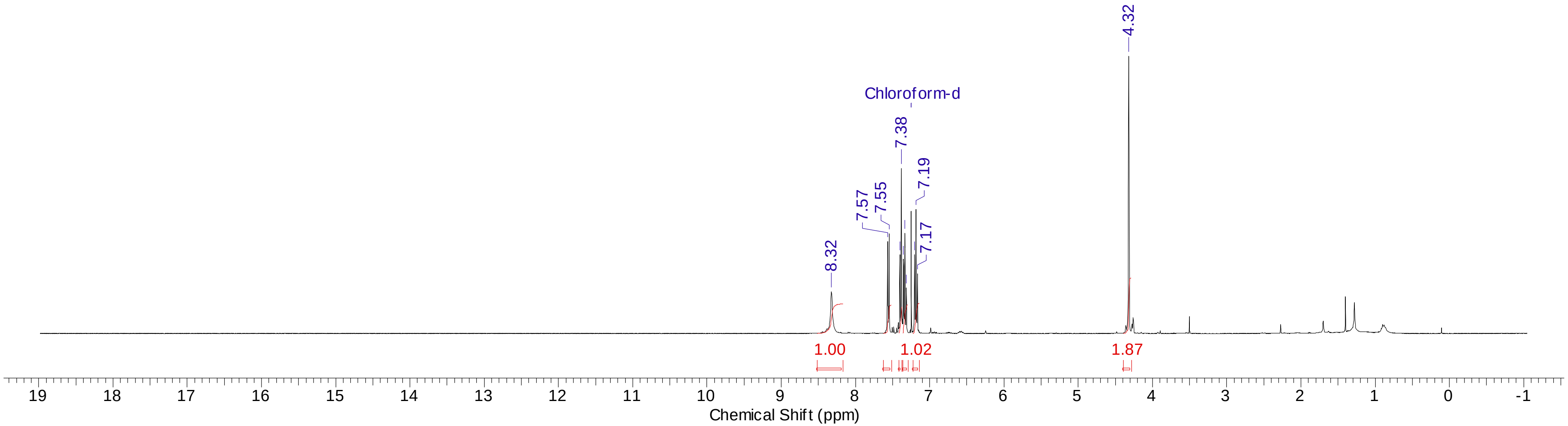

^{1}H NMR spectrum of **4n** (400.1 MHz, $CDCl_3$).

19 Jun 2020

Acquisition Time (sec)	0.7340	Date	Dec 13 2019	File Name	C:\DOCS\OUTPUT_301\F19\2019.12.13\BM-1824-1_20191213_01\FLUORINE_01		
Frequency (MHz)	376.31	Nucleus	19F	Number of Transients	1	Original Points Count	65536
Points Count	65536	Pulse Sequence	s2pul	Solvent	CHLOROFORM-D		
Sweep Width (Hz)	89285.71	Temperature (degree C)	30.000				

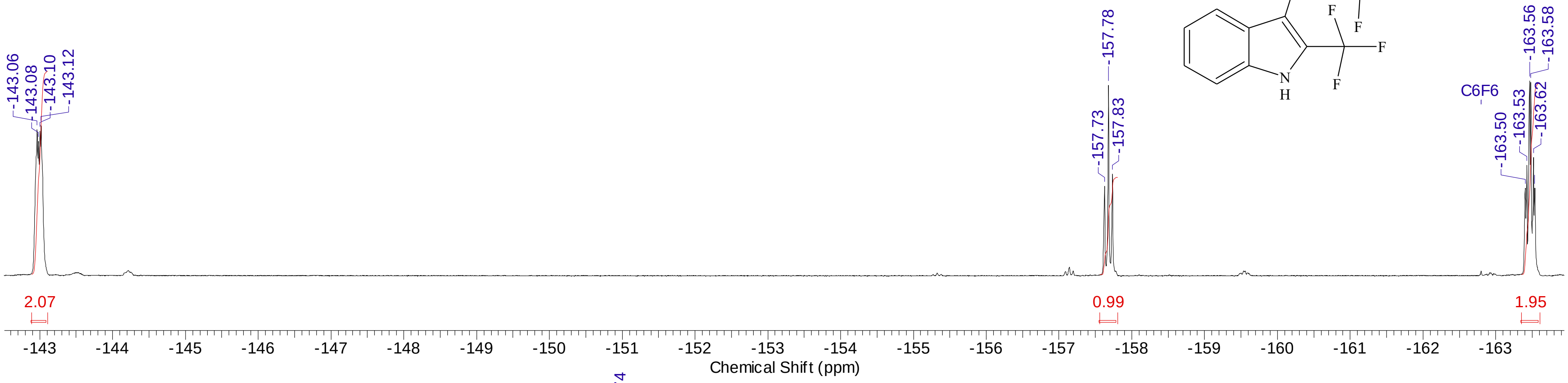

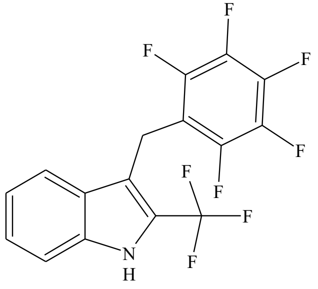

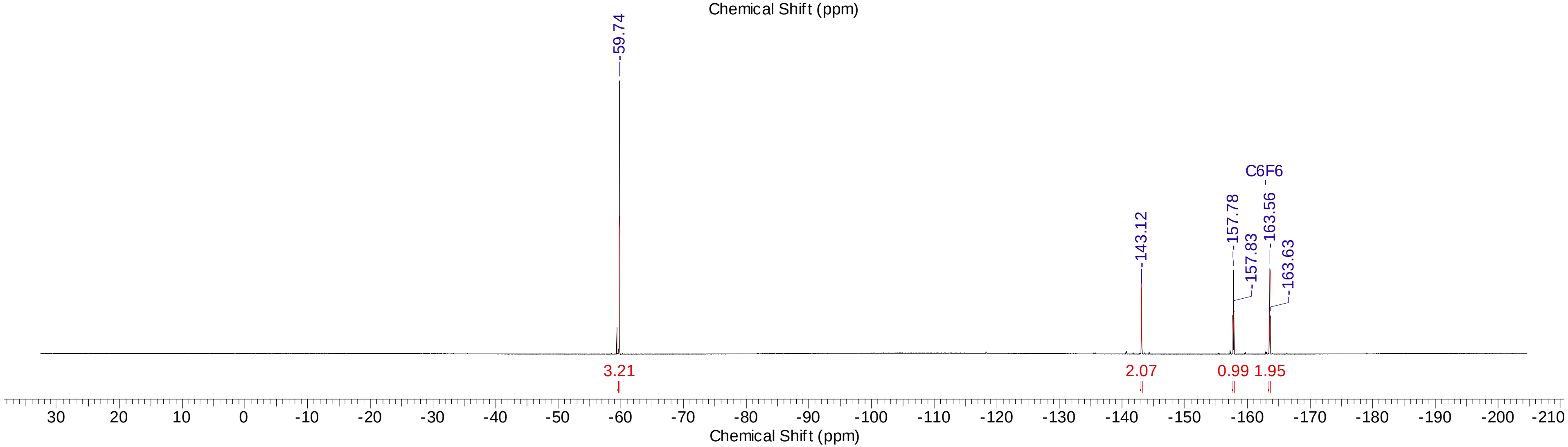

^{19}F NMR spectrum of **4n** (376.5 MHz, $CDCl_3$)

19 Jun 2020

Acquisition Time (sec)	0.6783	***Comment***	Imported from UXNMR.			***Date***	12 Dec 2019 12:43:54
File Name	C:\DOCS\OUTPUT_301\2019\12.溴赅狃黒BM-1824-1.C_002001r					***Frequency (MHz)***	100.61
Nucleus	13C	***Number of Transients***	626	***Original Points Count***	16384	***Points Count***	131072
Pulse Sequence	zgpg30	***Solvent***	CHLOROFORM-D			***Sweep Width (Hz)***	24154.59
Temperature (degree C)	27.000						

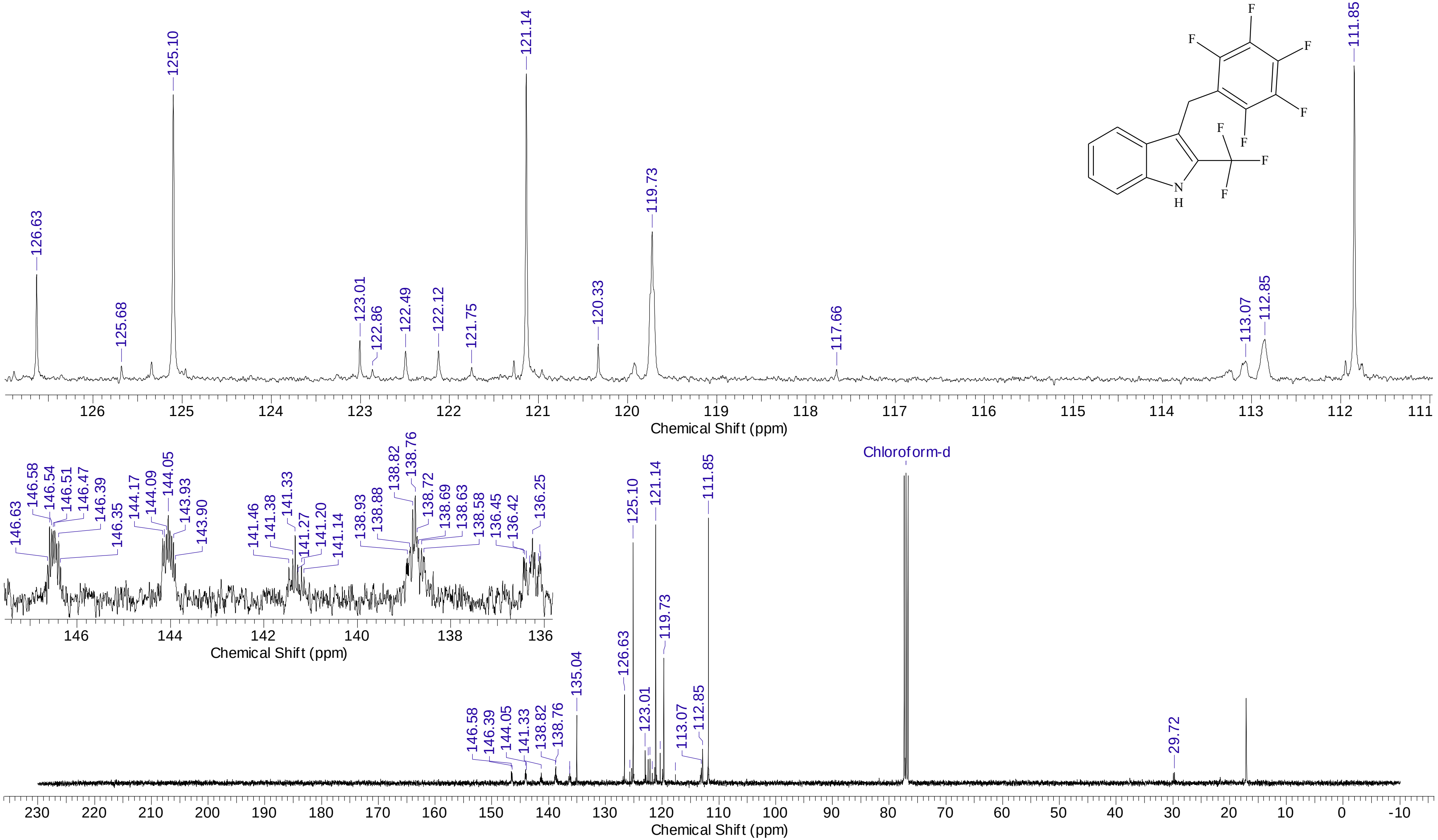

$^{13}C\{^{1}H\}$ NMR spectrum of **4n** (100.6 MHz, $CDCl_3$)

19 Jun 2020

Acquisition Time (sec)	4.0894	***Comment***	Imported from UXNMR.			***Date***	02 Dec 2019 15:51:50
File Name	C:\DOCS\OUTPUT_301\2019\12.溴赅狃黒BM-1802-1.H_001001r					***Frequency (MHz)***	400.13
Nucleus	1H	***Number of Transients***	7	***Original Points Count***	32768	***Points Count***	131072
Pulse Sequence	zg30	***Solvent***	CHLOROFORM-D			***Sweep Width (Hz)***	8012.82
Temperature (degree C)	27.000						

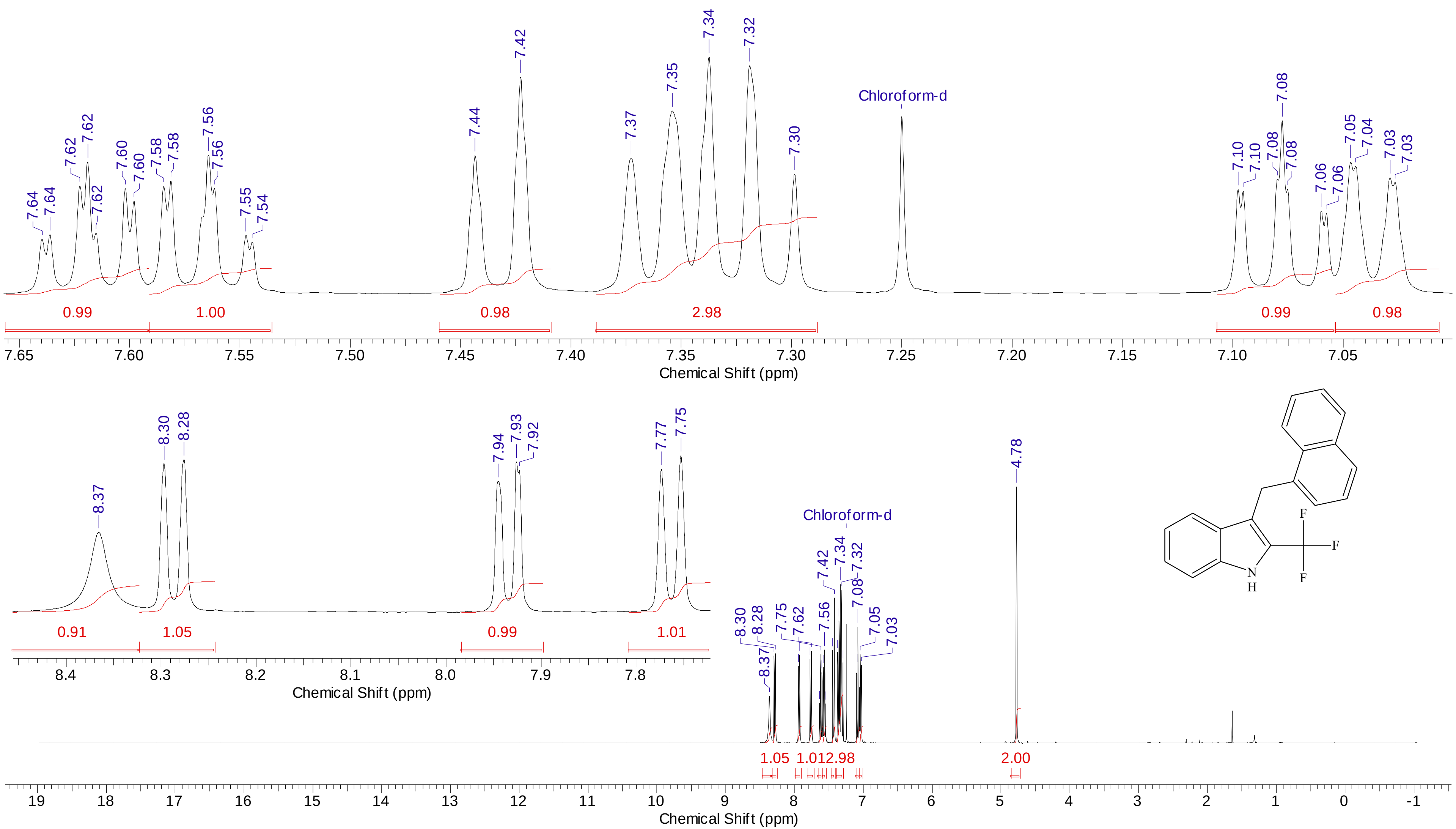

^{1}H NMR spectrum of **4o** (400.1 MHz, $CDCl_3$)

19 Jun 2020

Acquisition Time (sec)	2.0000	***Date***	Dec 2 2019	***File Name***	C:\DOCS\OUTPUT_301\F19\2019.12.02\BM-1802-1_20191202_01\FLUORINE_01		
Frequency (MHz)	376.31	***Nucleus***	19F	***Number of Transients***	16	***Original Points Count***	178571
Points Count	262144	***Pulse Sequence***	s2pul	***Solvent***	CHLOROFORM-D		
Sweep Width (Hz)	89285.71	***Temperature (degree C)***	22.000				

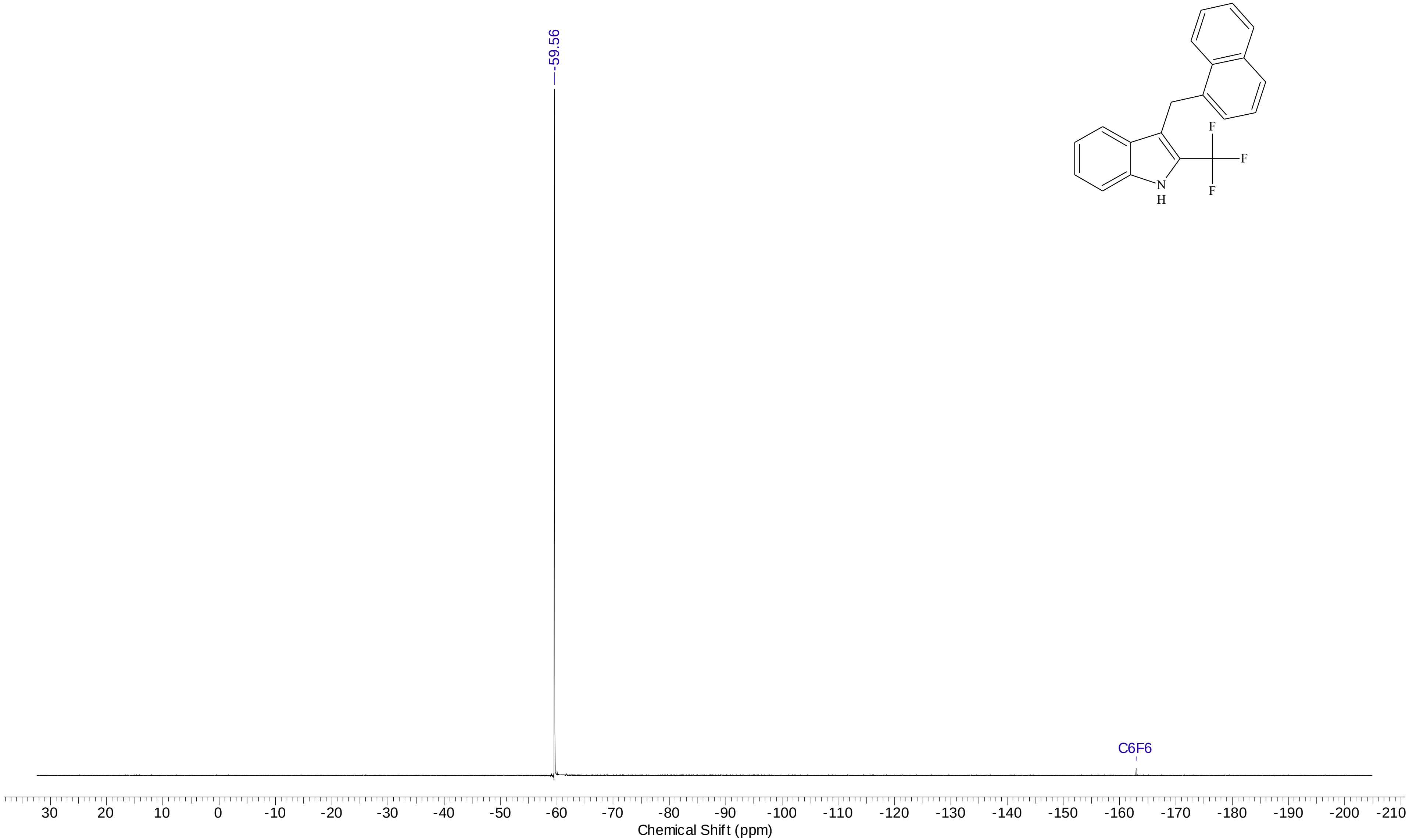

^{19}F NMR spectrum of **4o** (376.5 MHz, $CDCl_3$)

19 Jun 2020

Acquisition Time (sec)	0.6783	Comment	Imported from UXNMR.			Date	02 Dec 2019 15:57:02
File Name	C:\DOCS\OUTPUT_301\2019\12.溴贱狃黒BM-1802-1.C_002001r					Frequency (MHz)	100.61
Nucleus	13C	Number of Transients	122	Original Points Count	16384	Points Count	131072
Pulse Sequence	zgpg30	Solvent	CHLOROFORM-D			Sweep Width (Hz)	24154.59
Temperature (degree C)	27.000						

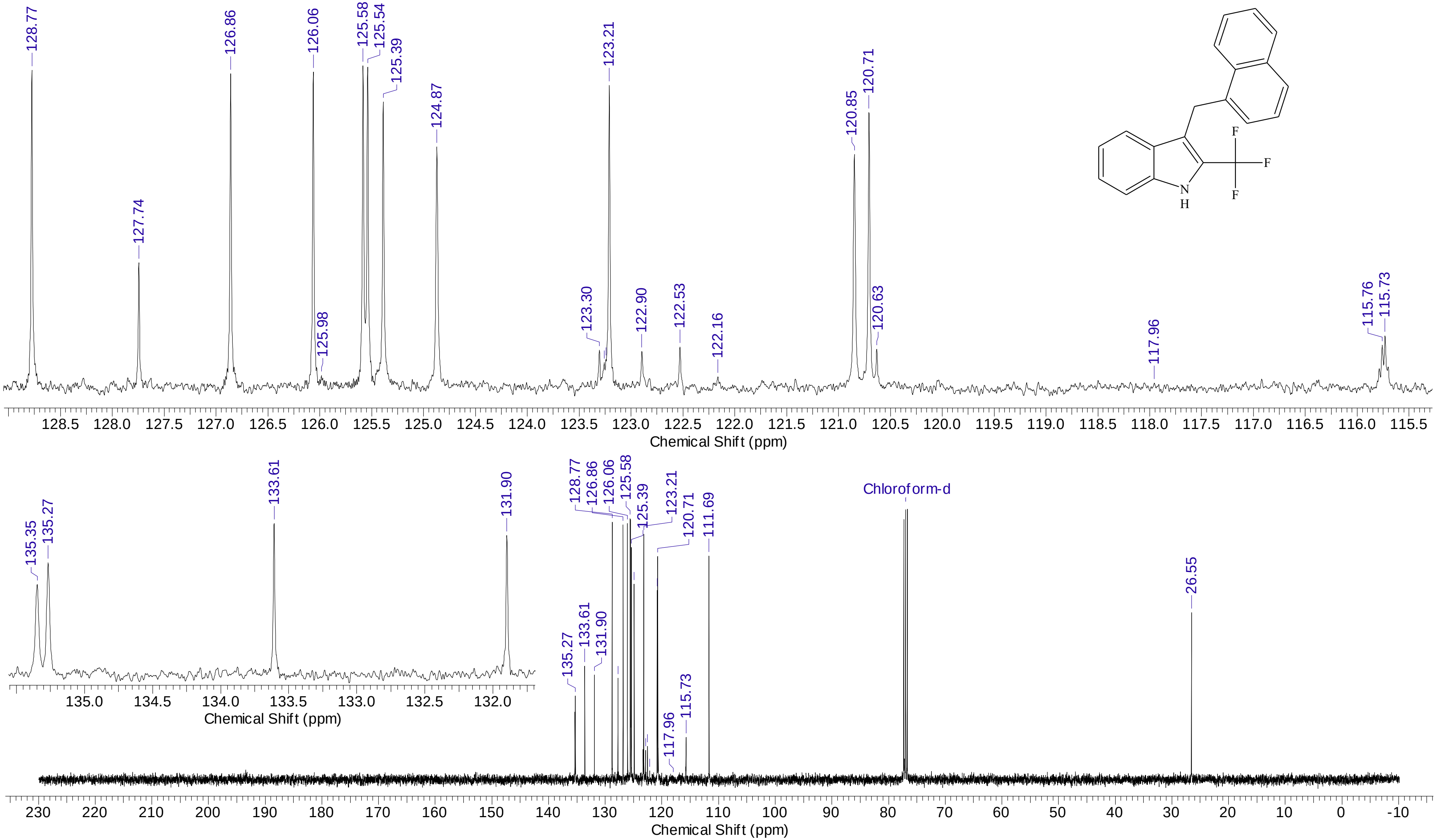

$^{13}C\{^1H\}$ NMR spectrum of **4o** (100.6 MHz, $CDCl_3$)

3 Jul 2021

Acquisition Time (sec)	4.0894	***Comment***	Imported from UXNMR.		***Date***	11 Jul 2020 21:52:54	
File Name	C:\BM_DATA\DOCS\200711\BM-1896_001001r		***Frequency (MHz)***	400.13	***Nucleus***	1H	***Number of Transients*** 8
Original Points Count	32768	***Points Count*** 131072	***Pulse Sequence***	zg30	***Solvent***	CHLOROFORM-D	
Sweep Width (Hz)	8012.82	***Temperature (degree C)*** 27.000					

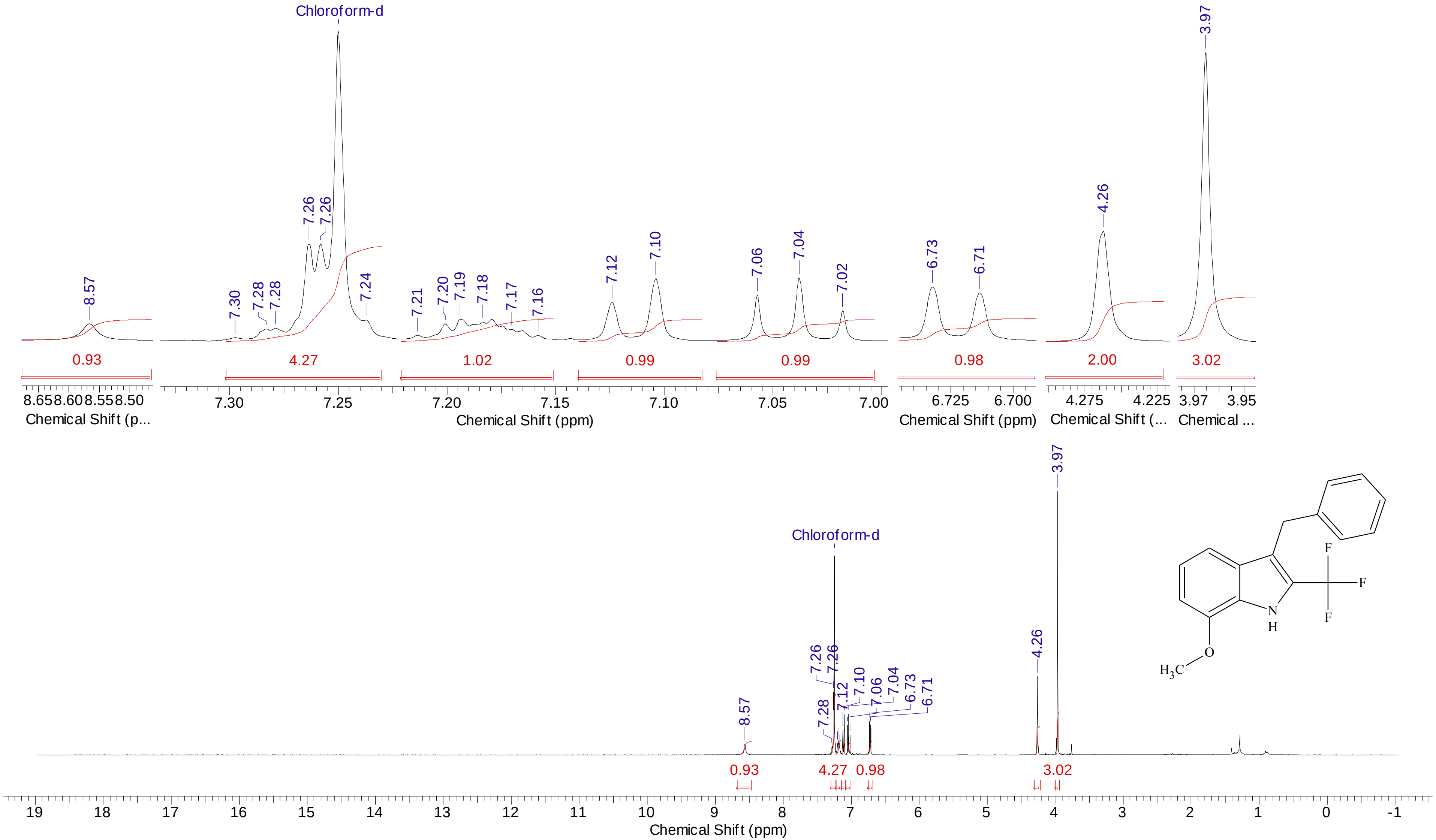

^{1}H NMR spectrum of **4p** (400.1 MHz, $CDCl_3$)

Acquisition Time (sec)	1.7433	***Comment***	Imported from UXNMR.			***Date***	11 Jul 2020 21:54:32
File Name	C:\BM_DATA\DOCS\200711\BM-1896_005001r			***Frequency (MHz)***	376.50	***Nucleus***	19F
Number of Transients	16	***Original Points Count***	131072	***Points Count***	262144	***Pulse Sequence***	zgflqn
Solvent	CHLOROFORM-D			***Sweep Width (Hz)***	75187.97	***Temperature (degree C)***	27.000

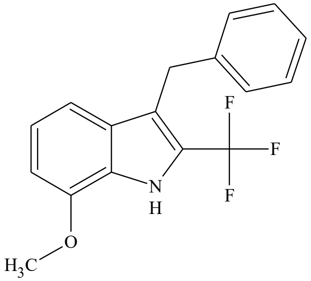

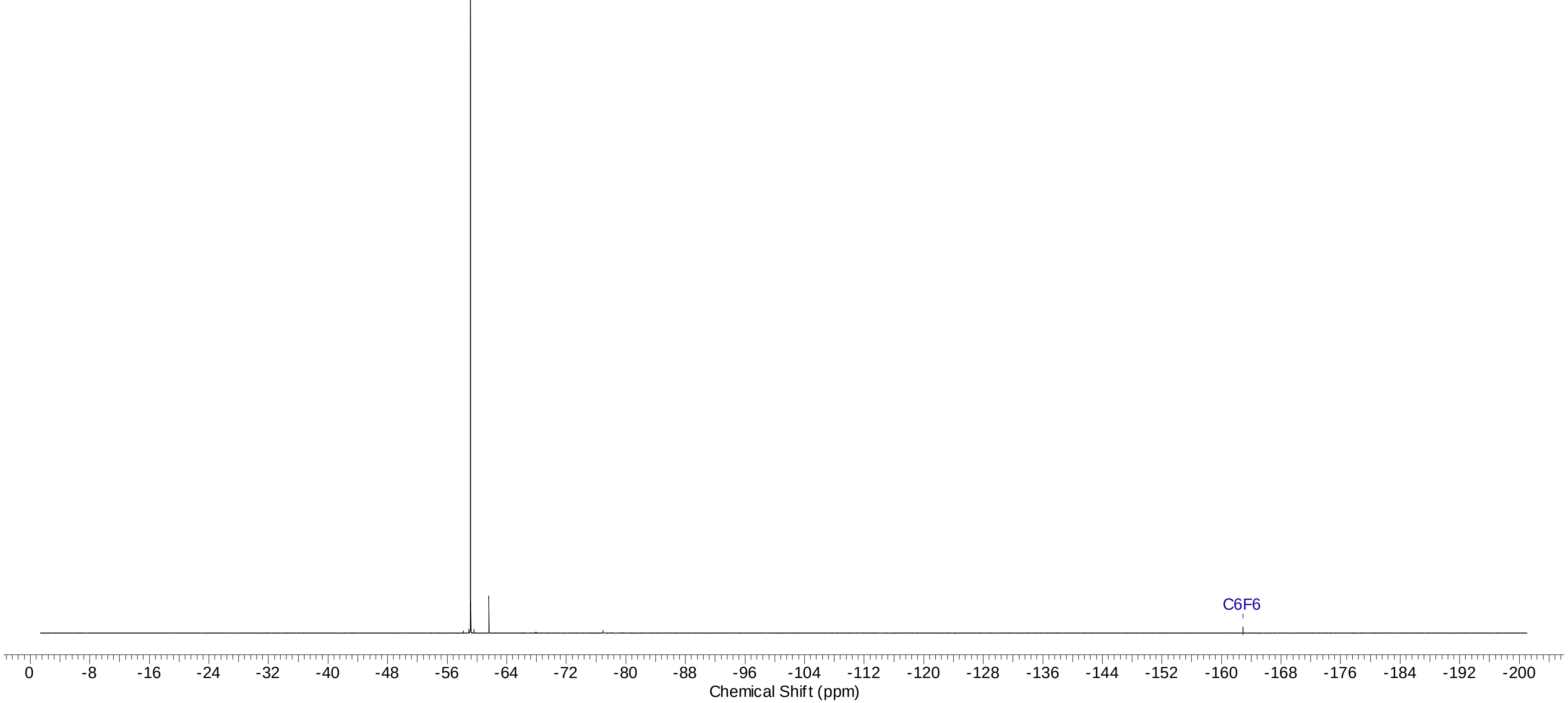

^{19}F NMR spectrum of **4p** (376.5 MHz, $CDCl_3$)

Acquisition Time (sec)	0.6783	***Comment***	Imported from UXNMR.			***Date***	10 Jul 2020 15:50:00
File Name	C:\DOCS\OUTPUT_301\2020\07.棹 朦\BM-1896.C_002001r			***Frequency (MHz)***	100.61	***Nucleus***	13C
Number of Transients	266	***Original Points Count***	16384	***Points Count***	131072	***Pulse Sequence***	zgpg30
Solvent	CHLOROFORM-D			***Sweep Width (Hz)***	24154.59	***Temperature (degree C)***	27.000

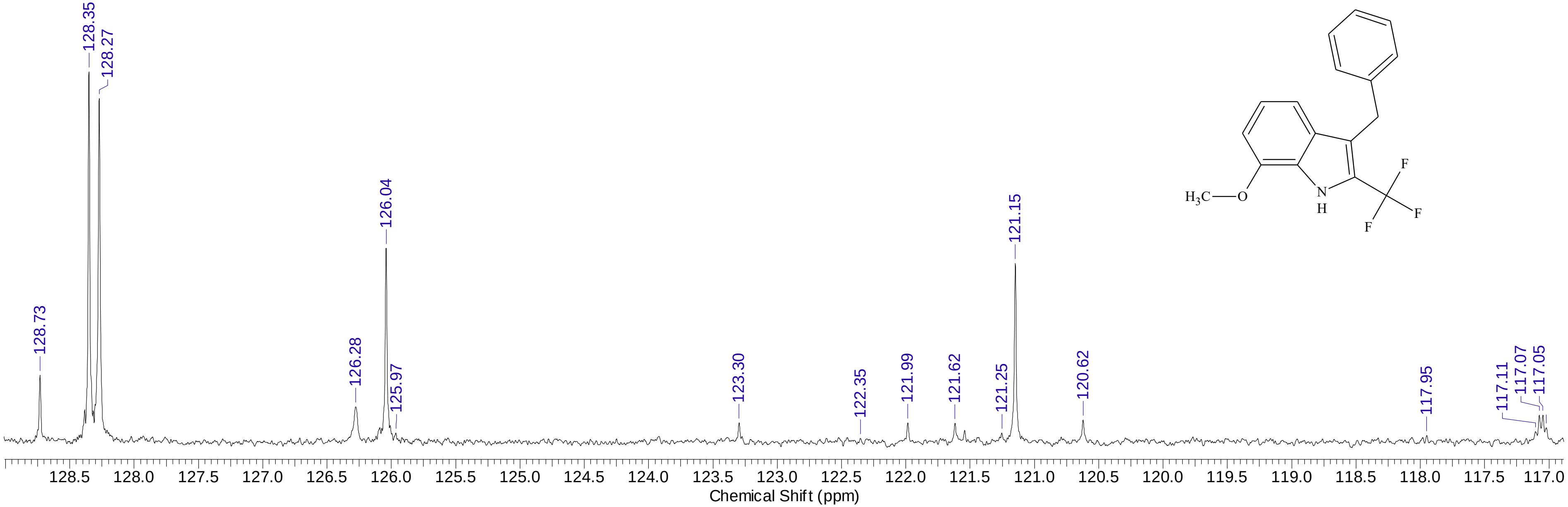

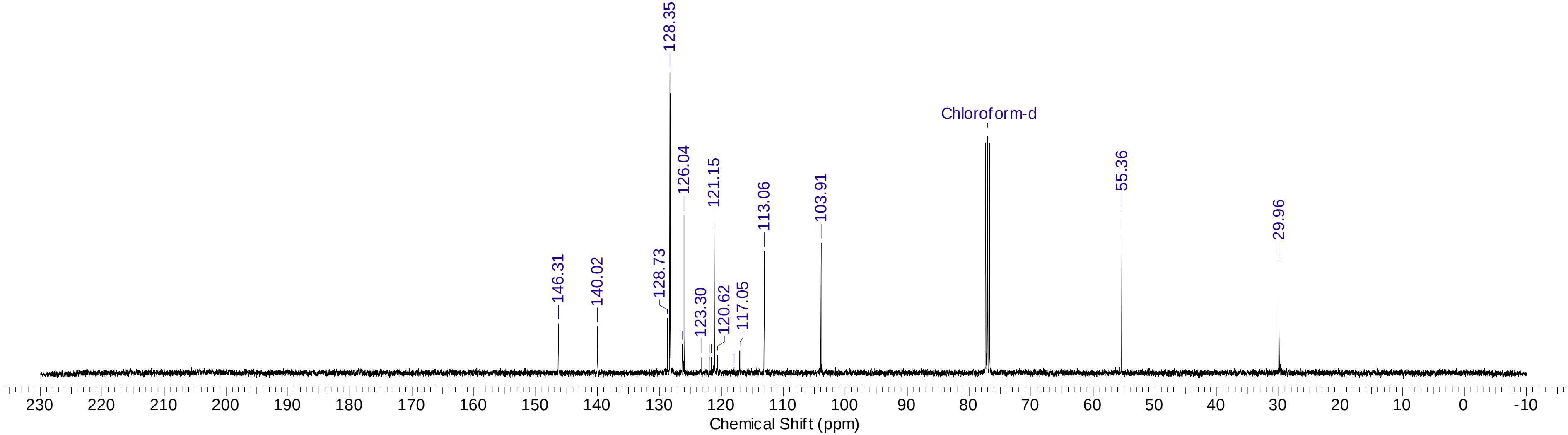

$^{13}C\{^1H\}$ NMR spectrum of **4p** (100.6 MHz, $CDCl_3$)

3 Jul 2021

Acquisition Time (sec)	4.0894	Comment	Imported from UXNMR.			Date	10 Apr 2021 13:30:22
File Name	C:\DOCS\OUTPUT_301\2021\04.图疱滕\BM-2130.H_001001r					Frequency (MHz)	400.13
Nucleus	1H	Number of Transients	4	Original Points Count	32768	Points Count	131072
Pulse Sequence	zg30	Solvent	CHLOROFORM-D			Sweep Width (Hz)	8012.82
Temperature (degree C)	27.000						

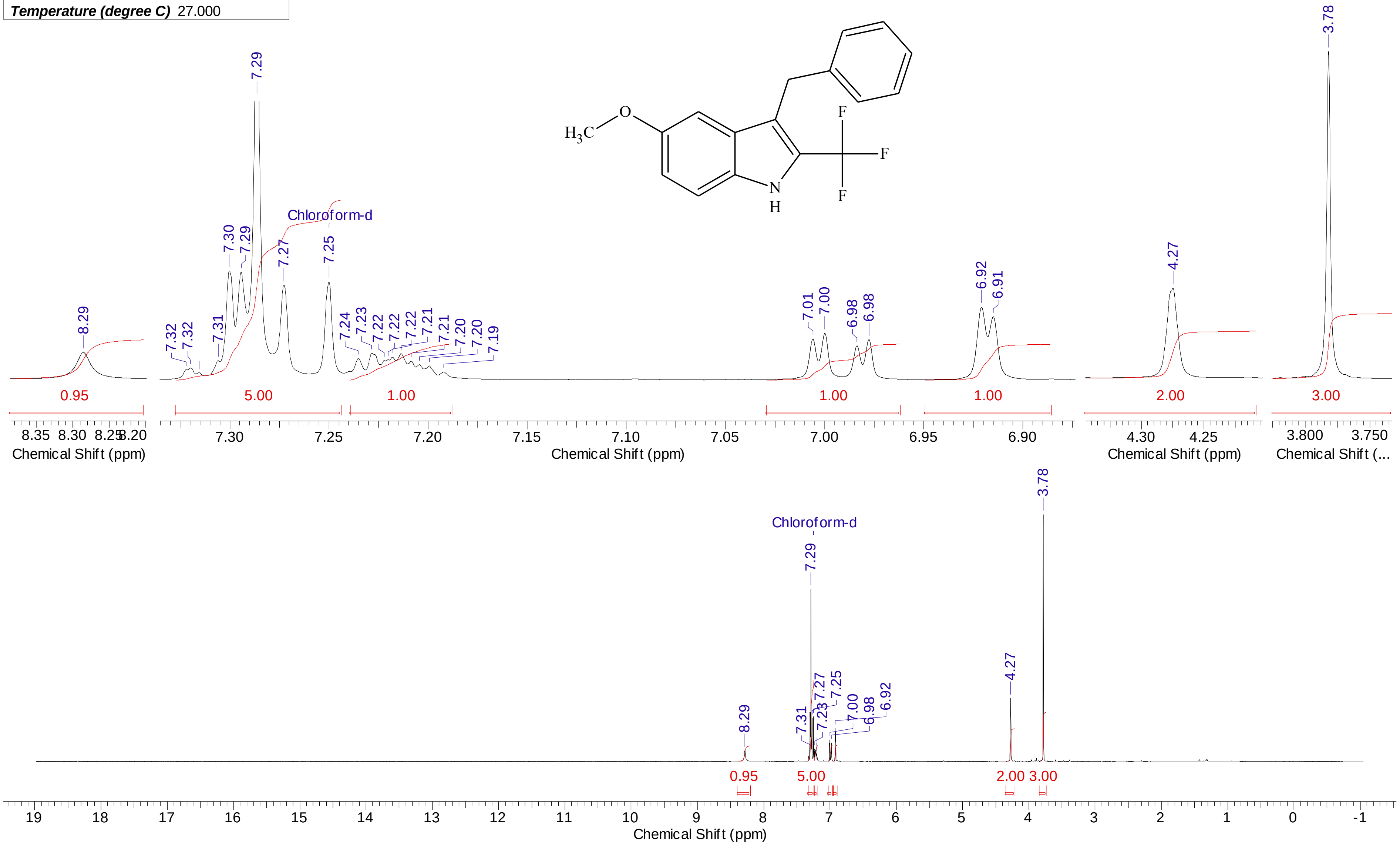

^{1}H NMR spectrum of **4q** (400.1 MHz, $CDCl_3$)

3 Jul 2021

Acquisition Time (sec)	1.7433	***Comment***	Imported from UXNMR.			***Date***	10 Apr 2021 13:44:20
File Name	C:\DOCS\OUTPUT_301\2021\04.图疱滕\BM-2130.F_005001r					***Frequency (MHz)***	376.50
Nucleus	19F	***Number of Transients***	16	***Original Points Count***	131072	***Points Count***	262144
Pulse Sequence	zgflqn	***Solvent***	DMSO-D6	***Sweep Width (Hz)***	75187.97	***Temperature (degree C)***	27.000

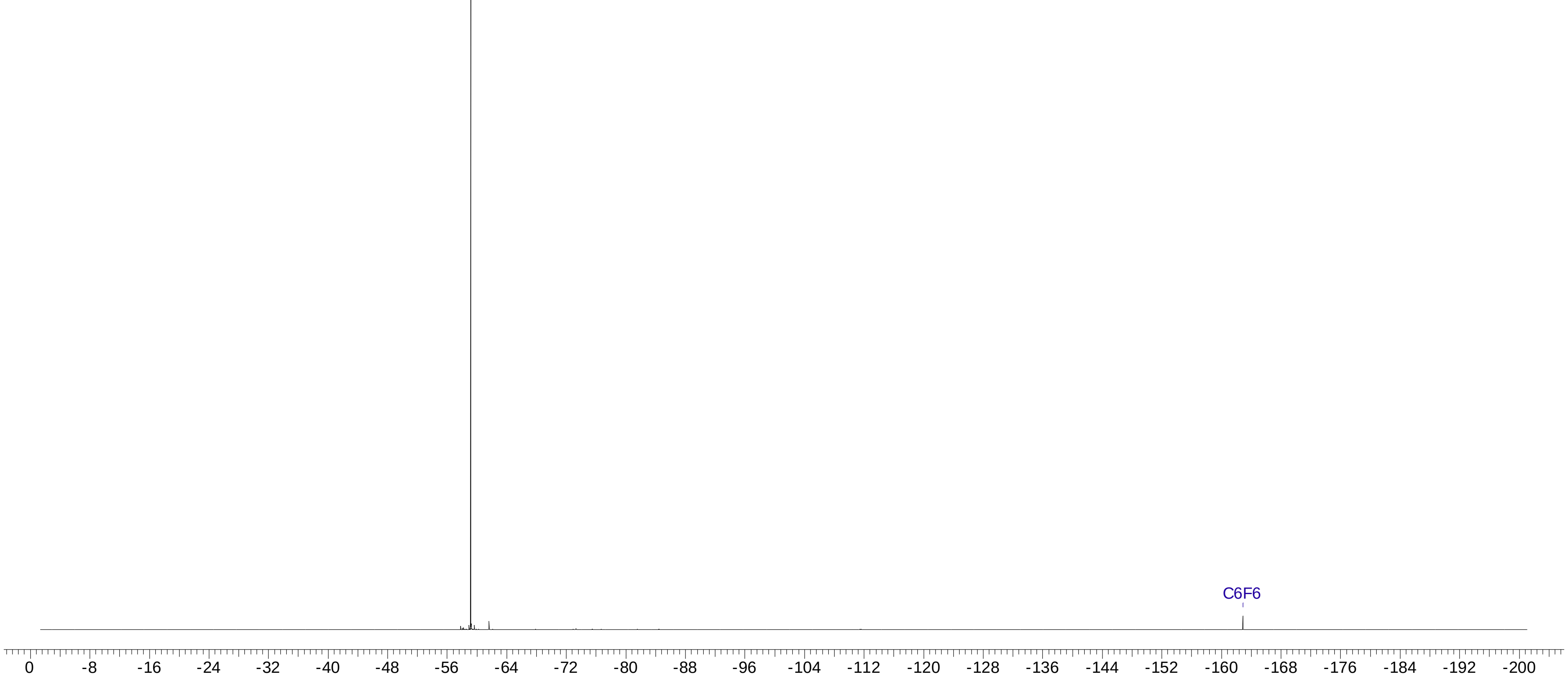

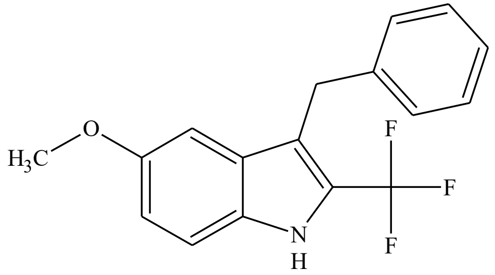

^{19}F NMR spectrum of **4q** (376.5 MHz, $CDCl_3$)

3 Jul 2021

Acquisition Time (sec)	0.6783	***Comment***	Imported from UXNMR.	***Date***	10 Apr 2021 13:40:10
File Name	C:\DOCS\OUTPUT_301\2021\04.図疱朦\BM-2130.C_002001r	***Frequency (MHz)***	100.61	***Nucleus***	13C
Number of Transients	201	***Original Points Count*** 16384	***Points Count*** 131072	***Pulse Sequence***	zgpg30
Solvent	CHLOROFORM-D	***Sweep Width (Hz)***	24154.59	***Temperature (degree C)***	27.000

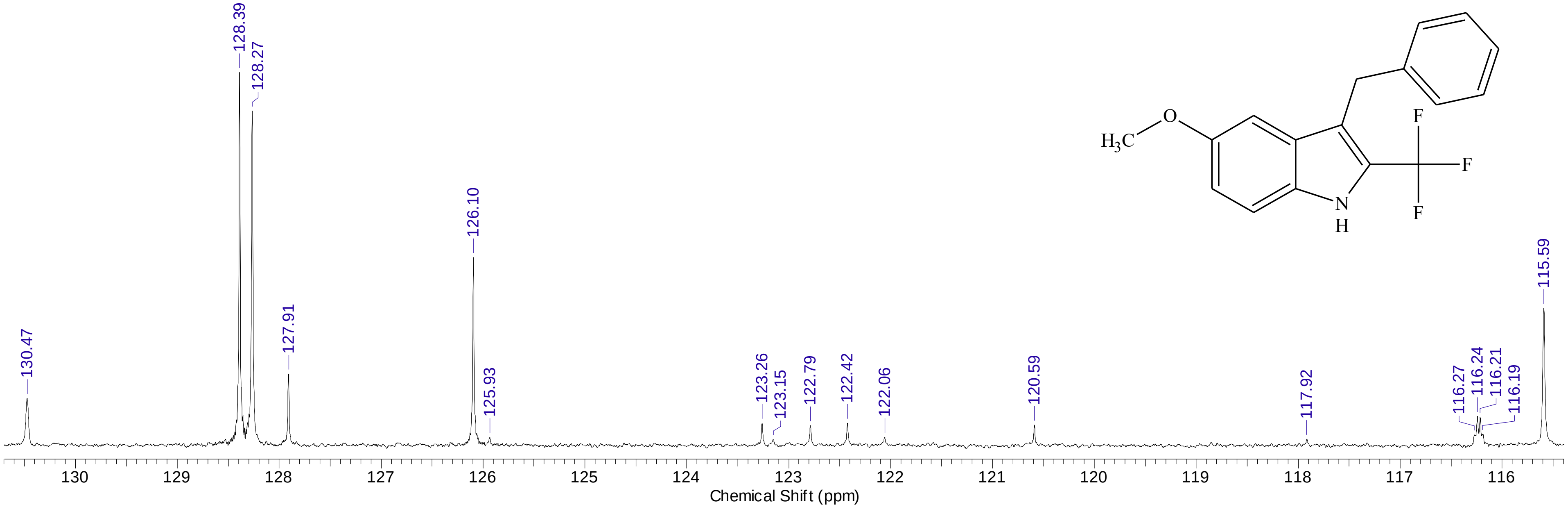

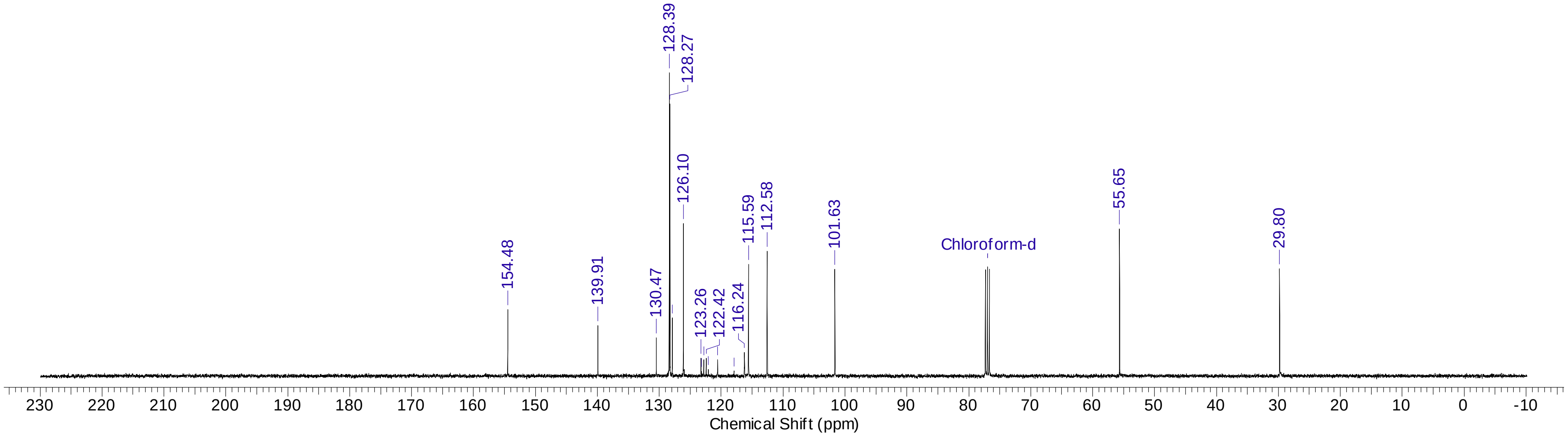

$^{13}C\{^1H\}$ NMR spectrum of **4q** (100.6 MHz, $CDCl_3$)

3 Jul 2021

Acquisition Time (sec)	4.0894	***Comment***	Imported from UXNMR.			***Date***	30 Mar 2021 14:49:28
File Name	C:\DOCS\OUTPUT_301\2021\03.爨 疖\BM-2116-1.H_001001r					***Frequency (MHz)***	400.13
Nucleus	1H	***Number of Transients***	4	***Original Points Count***	32768	***Points Count***	131072
Pulse Sequence	zg30	***Solvent***	DMSO-D6	***Sweep Width (Hz)***	8012.82	***Temperature (degree C)***	27.000

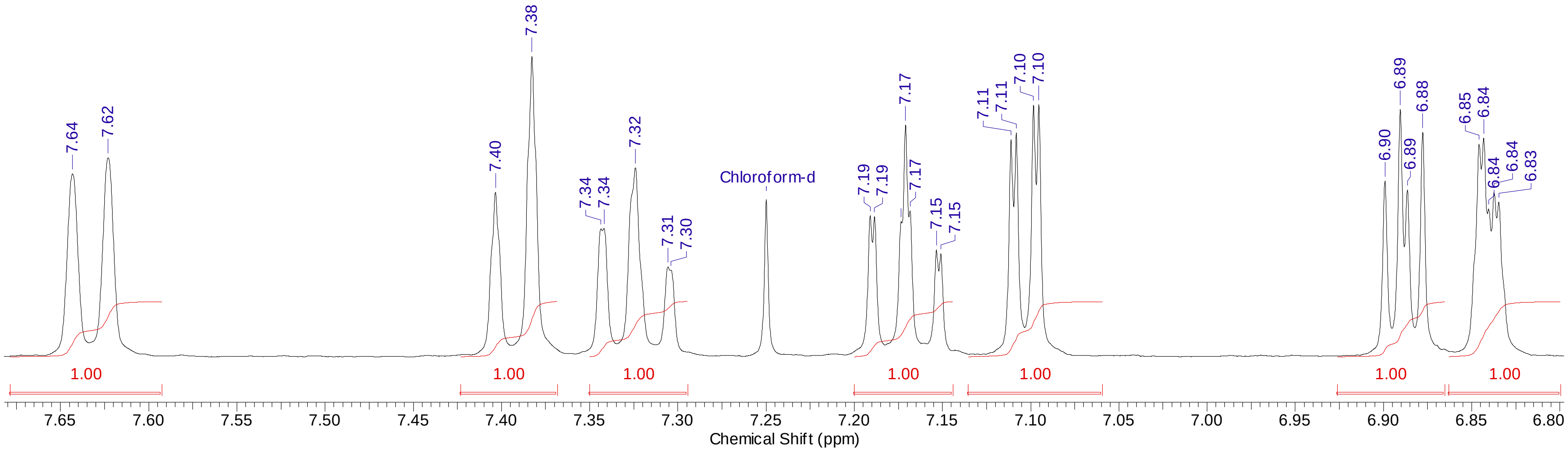

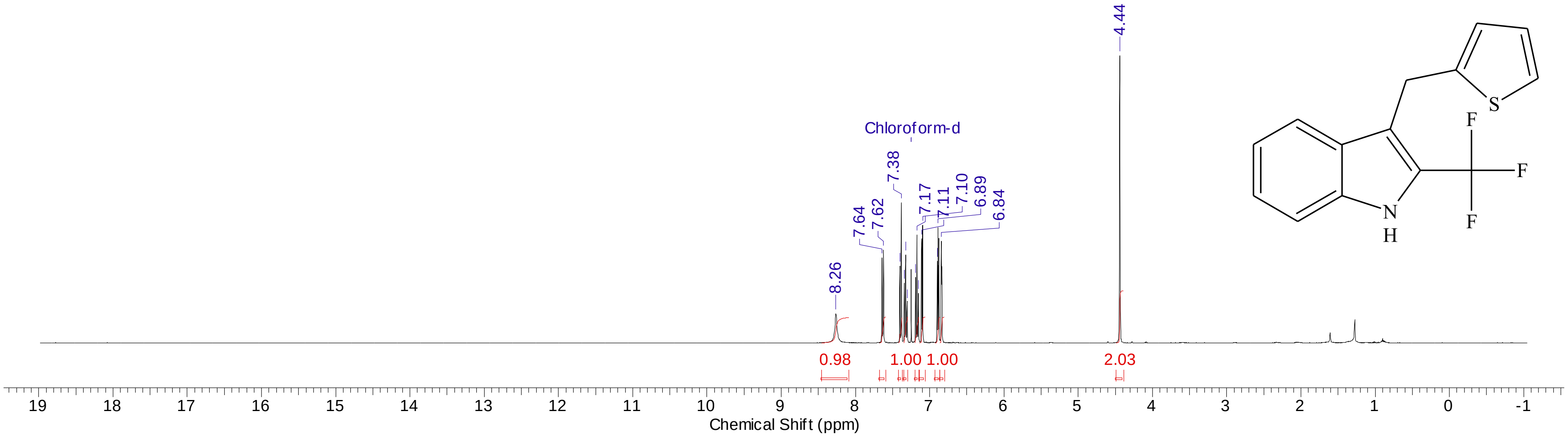

^{1}H NMR spectrum of **4r** (400.1 MHz, $CDCl_3$)

3 Jul 2021

Acquisition Time (sec)	1.7433	***Comment***	Imported from UXNMR.			***Date***	30 Mar 2021 15:52:02
File Name	C:\DOCS\OUTPUT_301\2021\03.爨 痱\BM-2116-1.F_005001r					***Frequency (MHz)***	376.50
Nucleus	19F	***Number of Transients***	11	***Original Points Count***	131072	***Points Count***	262144
Pulse Sequence	zgflqn	***Solvent***	DMSO-D6	***Sweep Width (Hz)***	75187.97	***Temperature (degree C)***	27.000

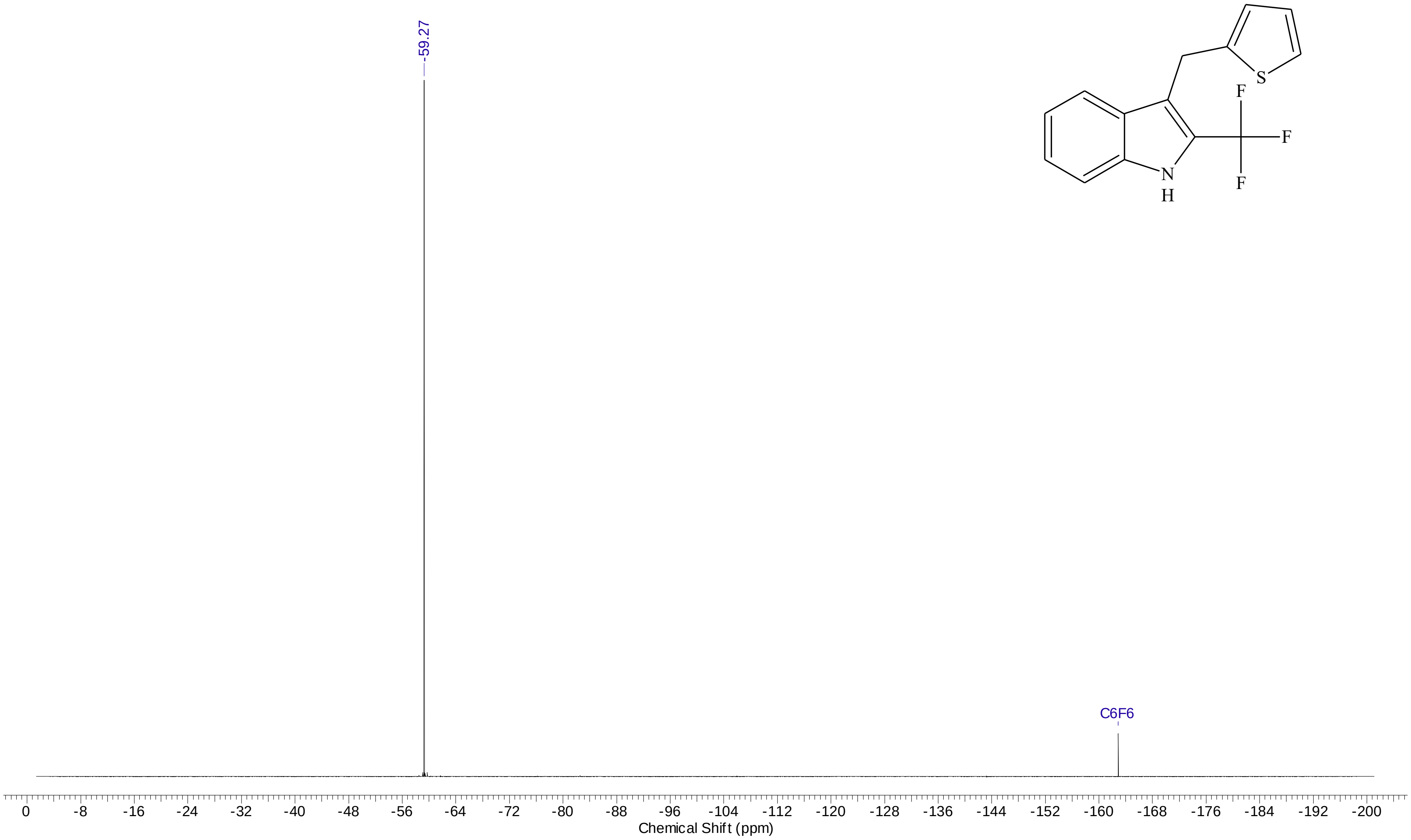

^{19}F NMR spectrum of **4r** (376.5 MHz, $CDCl_3$)

Acquisition Time (sec)	0.6783	***Comment***	Imported from UXNMR.	***Date***	30 Mar 2021 15:06:26
File Name	C:\DOCS\OUTPUT_301\2021\03.爨 痱\BM-2116-1.C_002001r	***Frequency (MHz)***	100.61	***Nucleus***	13C
Number of Transients	433	***Original Points Count*** 16384	***Points Count*** 131072	***Pulse Sequence***	zgpg30
Solvent	CHLOROFORM-D	***Sweep Width (Hz)***	24154.59	***Temperature (degree C)***	27.000

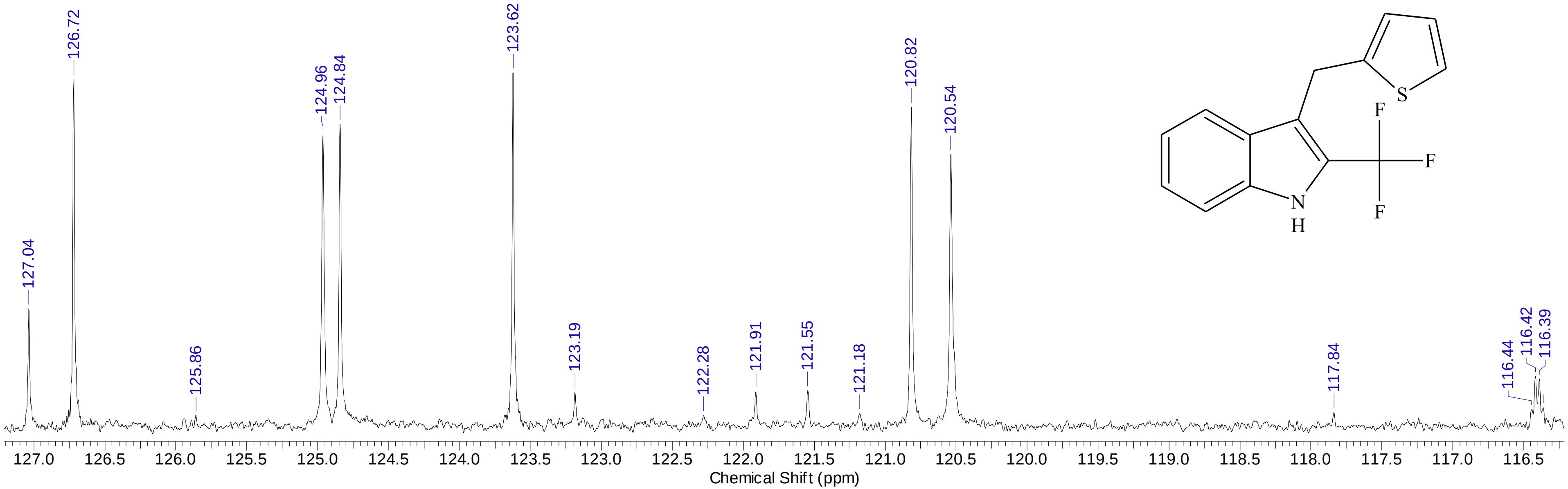

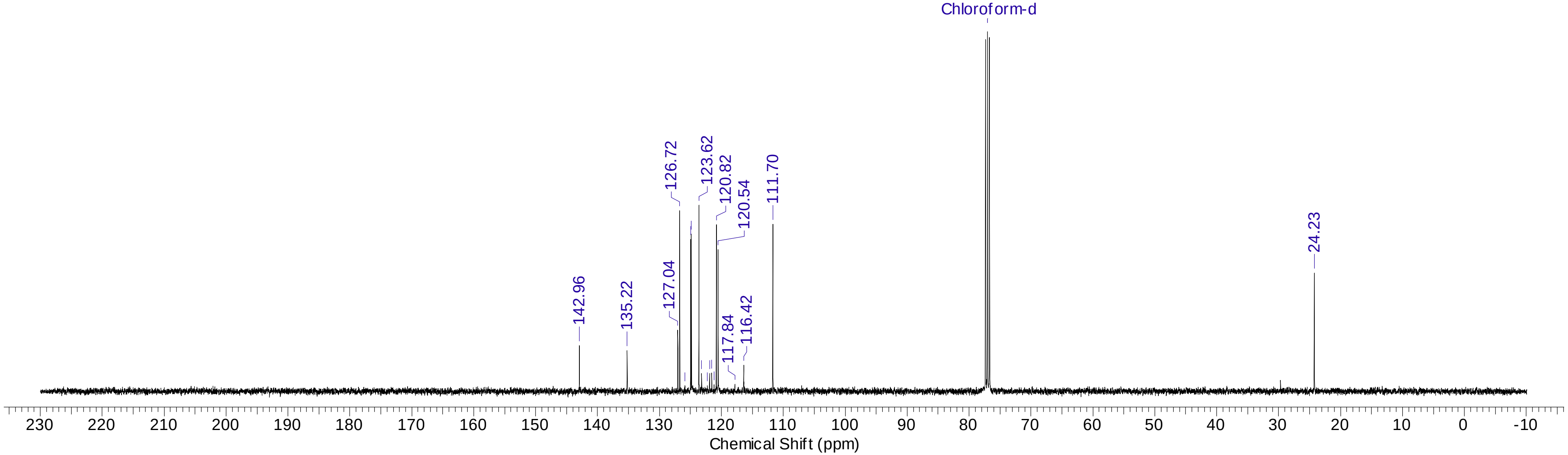

$^{13}C\{^1H\}$ NMR spectrum of **4r** (100.6 MHz, $CDCl_3$)

3 Jul 2021

Acquisition Time (sec)	4.0894	***Comment***	Imported from UXNMR.			***Date***	18 Mar 2021 12:10:04
File Name	C:\DOCS\OUTPUT_301\2021\03.爨 痱\BM-2104-C.H_001001r					***Frequency (MHz)***	400.13
Nucleus	1H	***Number of Transients***	4	***Original Points Count***	32768	***Points Count***	131072
Pulse Sequence	zg30	***Solvent***	CHLOROFORM-D			***Sweep Width (Hz)***	8012.82
Temperature (degree C)	27.000						

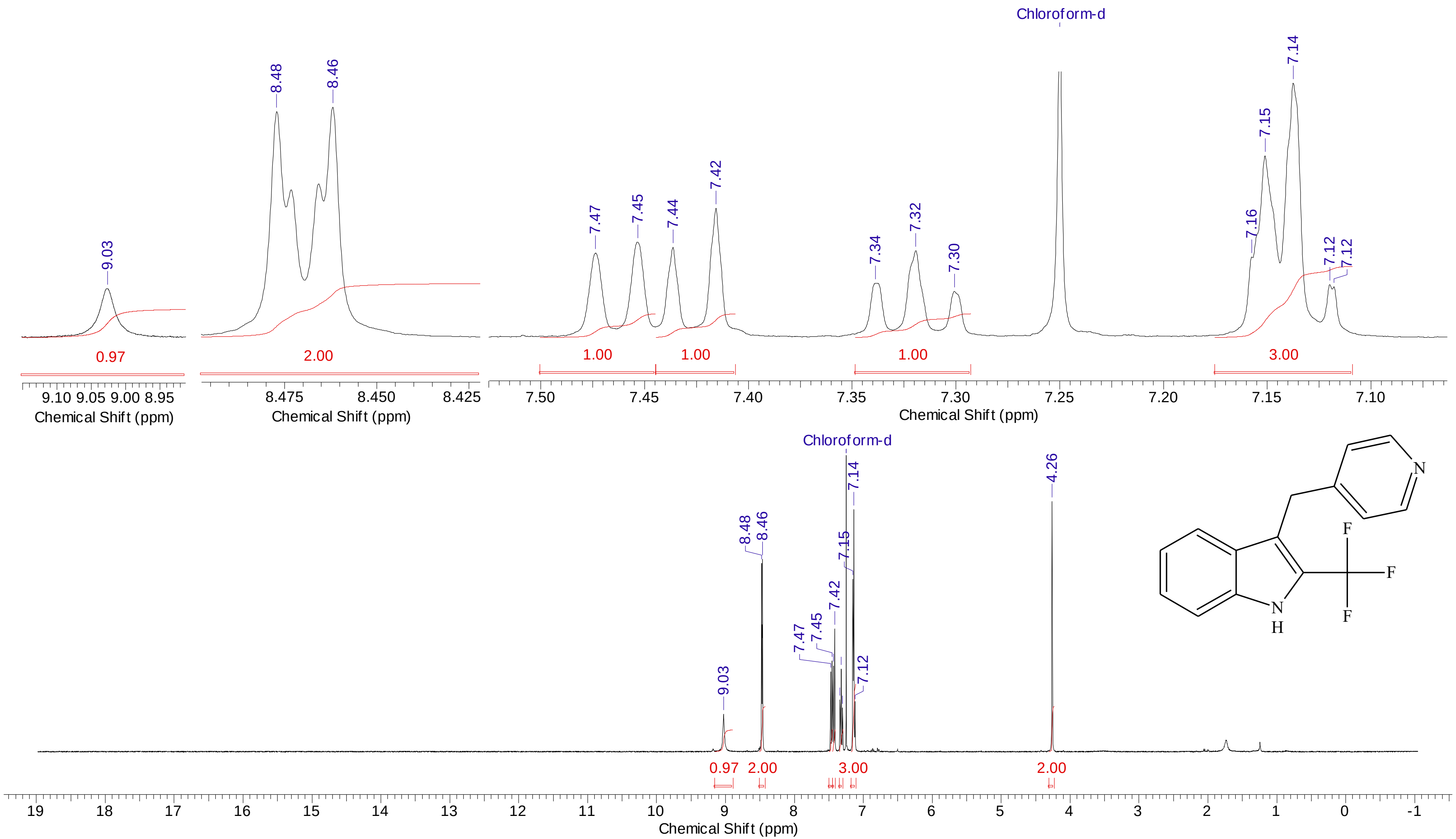

^{1}H NMR spectrum of **4s** (400.1 MHz, CD_3CN)

3 Jul 2021

Acquisition Time (sec)	1.7433	***Comment***	Imported from UXNMR.	***Date***	18 Mar 2021 13:06:30
File Name	C:\DOCS\OUTPUT_301\2021\03.爨 痱\BM-2104-C.F_005001r	***Frequency (MHz)***	376.50	***Nucleus***	19F
Number of Transients	16	***Original Points Count*** 131072	***Points Count*** 262144	***Pulse Sequence***	zgflqn
Solvent	CHLOROFORM-D		***Sweep Width (Hz)*** 75187.97	***Temperature (degree C)***	27.000

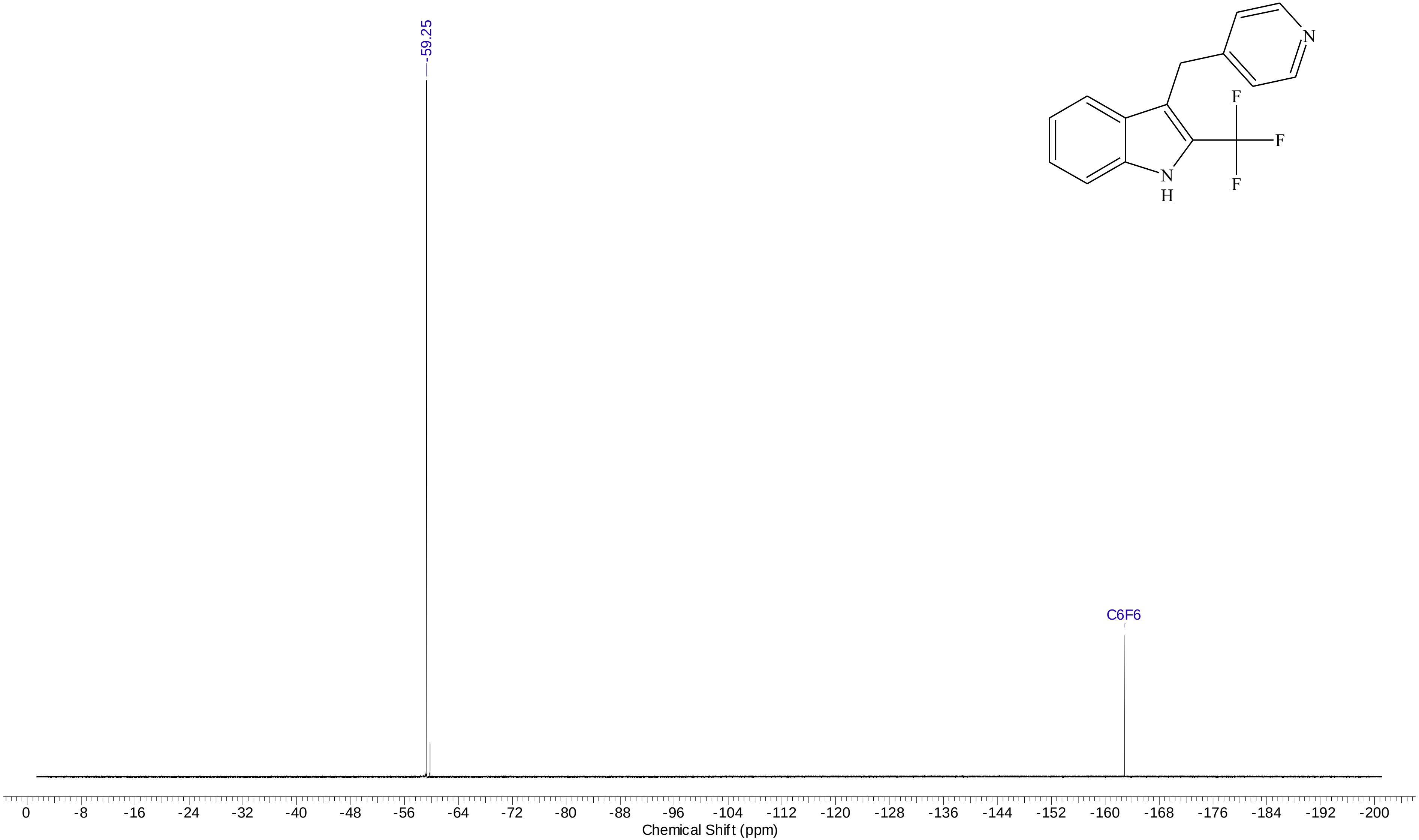

^{19}F NMR spectrum of **4s** (376.5 MHz, CD_3CN)

3 Jul 2021

Acquisition Time (sec)	0.6783	Comment	Imported from UXNMR.			Date	16 Mar 2021 14:07:26
File Name	C:\DOCS\OUTPUT_301\2021\03.爨 痱\BM-2104-2.C_002001r					Frequency (MHz)	100.61
Nucleus	13C	Number of Transients	801	Original Points Count	16384	Points Count	131072
Pulse Sequence	zgpg30	Solvent	DMSO-D6	Sweep Width (Hz)	24154.59	Temperature (degree C)	27.000

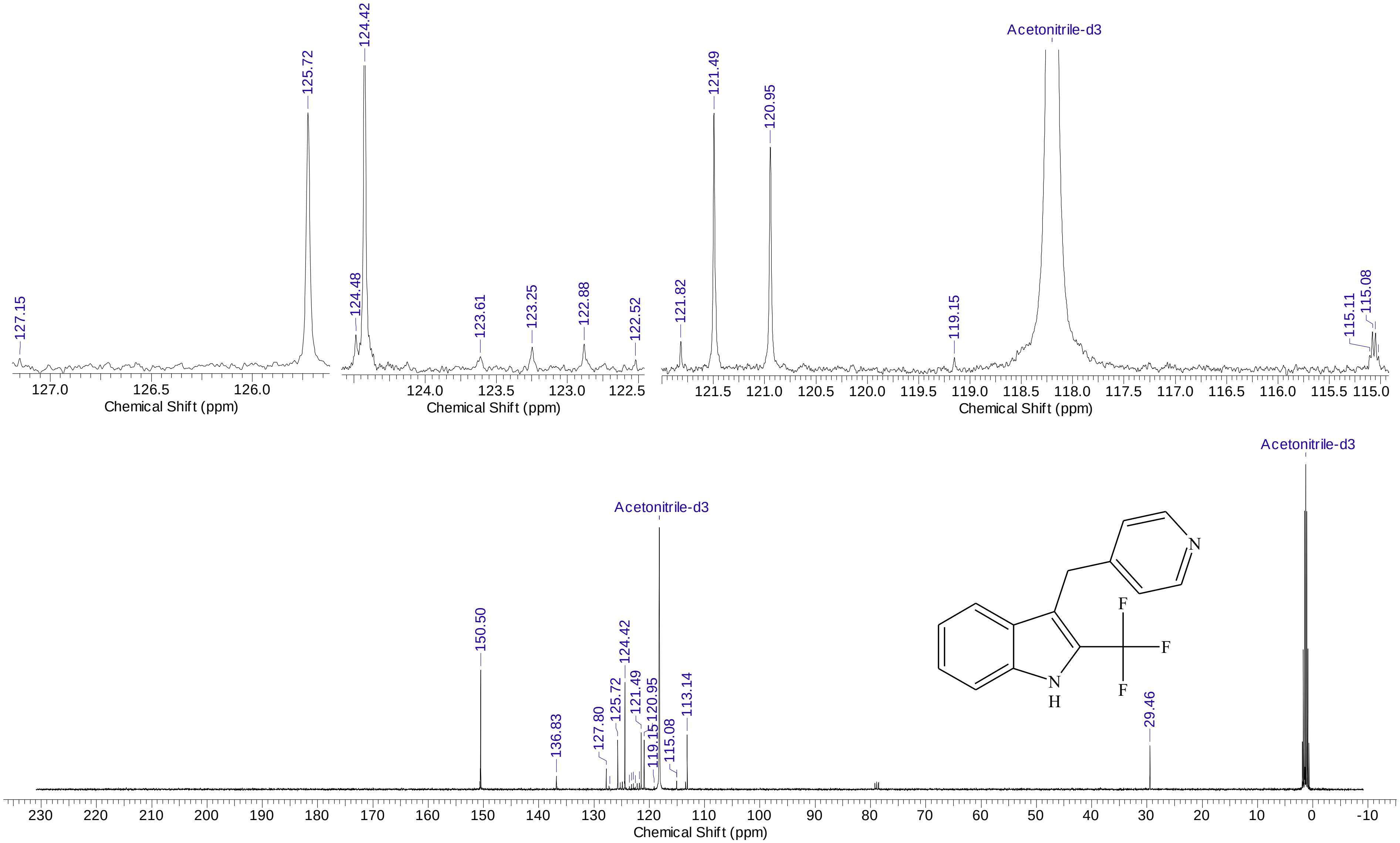

$^{13}C\{^1H\}$ NMR spectrum of **4s** (100.6 MHz, CD_3CN)

9 Jun 2021

Acquisition Time (sec)	4.0894	Comment	Imported from UXNMR.		Date	23 Jan 2021 12:24:10
File Name	C:\DOCS\OUTPUT_301\2021\01. 磺囧黒BM-2038-3p.H_001001r				Frequency (MHz)	400.13
Nucleus	1H	Number of Transients	4	Original Points Count 32768	Points Count	131072
Pulse Sequence	zg30	Solvent	CHLOROFORM-D		Sweep Width (Hz)	8012.82
Temperature (degree C)	27.000					

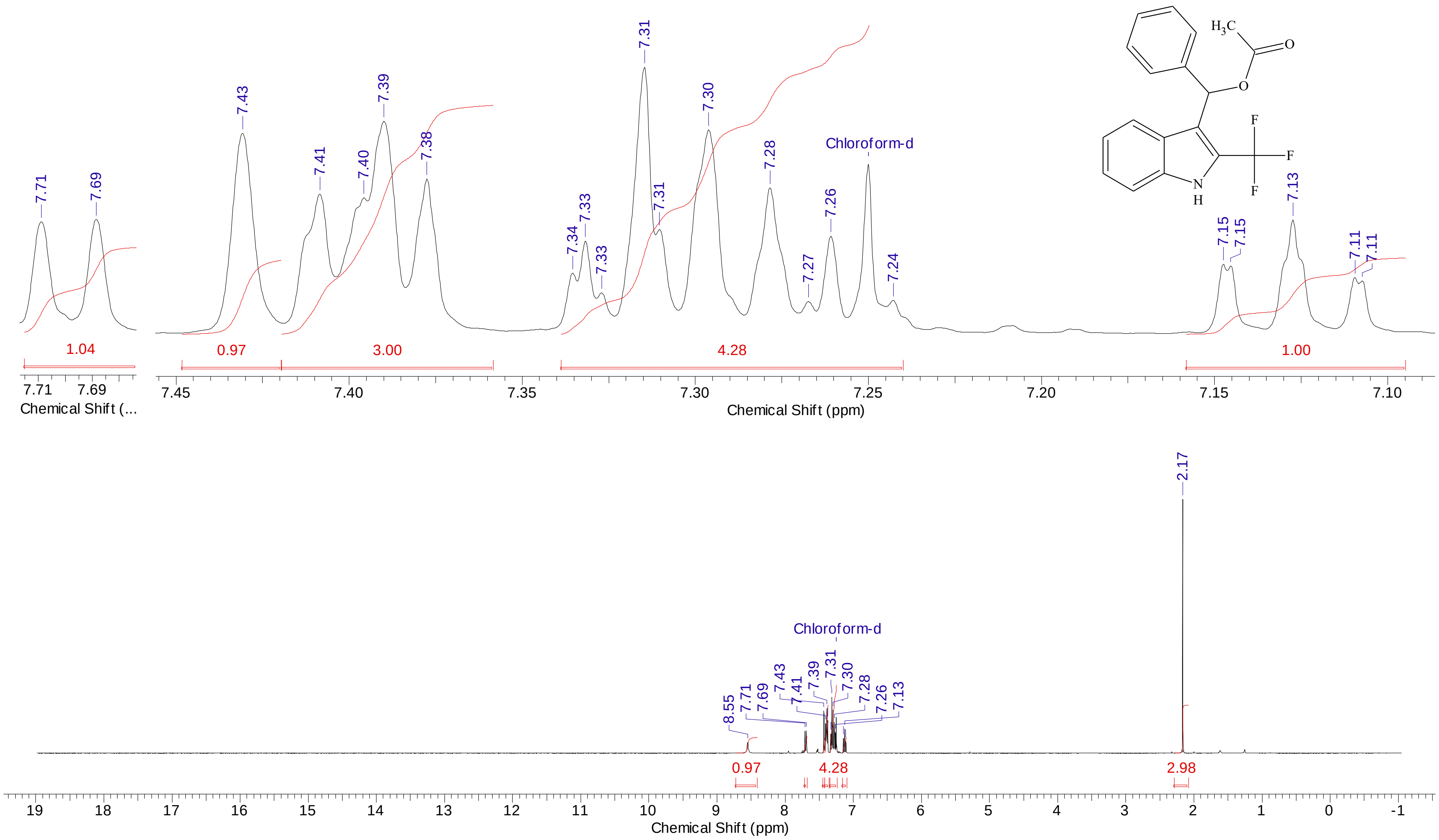

^{1}H NMR spectrum of **5a** (400.1 MHz, $CDCl_3$)

9 Jun 2021

Acquisition Time (sec)	1.7433	***Comment***	Imported from UXNMR.			***Date***	23 Jan 2021 23:10:56
File Name	C:\DOCS\BM\bm210123\BM-2038-3p_005001r			***Frequency (MHz)***	376.50	***Nucleus***	19F
Number of Transients	16	***Original Points Count***	131072	***Points Count***	262144	***Pulse Sequence***	zgflqn
Solvent	CHLOROFORM-D			***Sweep Width (Hz)***	75187.97	***Temperature (degree C)***	27.000

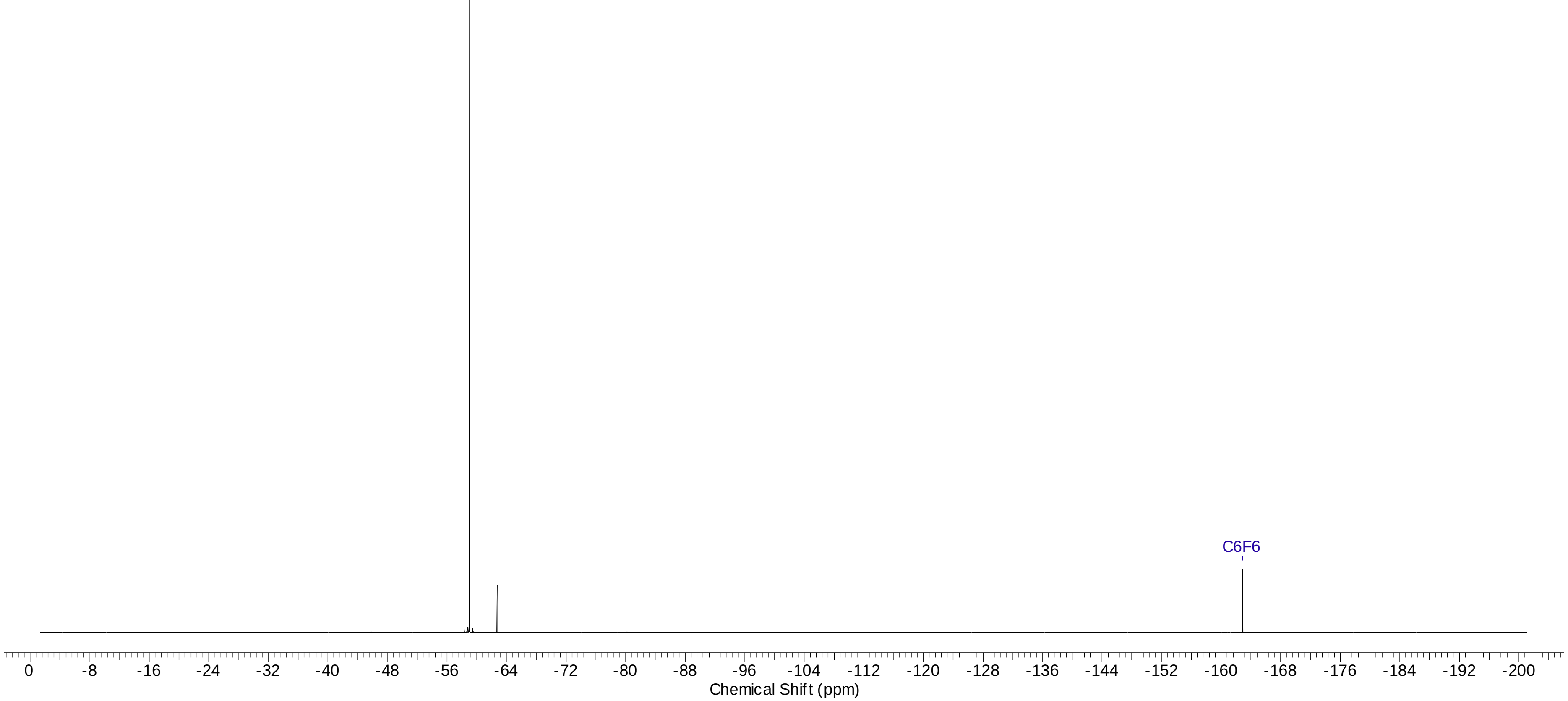

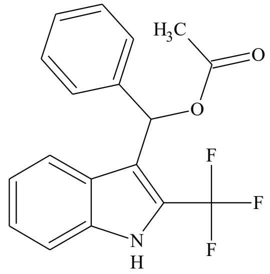

^{19}F NMR spectrum of **5a** (376.5 MHz $CDCl_3$)

9 Jun 2021

Acquisition Time (sec)	0.6783	***Comment***	Imported from UXNMR.			***Date***	26 Jan 2021 16:10:22
File Name	C:\DOCS\OUTPUT_301\2021\01. 磡啚黒BM-2038-3p.C_002001r					***Frequency (MHz)***	100.61
Nucleus	13C	***Number of Transients***	929	***Original Points Count***	16384	***Points Count***	131072
Pulse Sequence	zgpg30	***Solvent***	DMSO-D6	***Sweep Width (Hz)***	24154.59	***Temperature (degree C)***	27.000

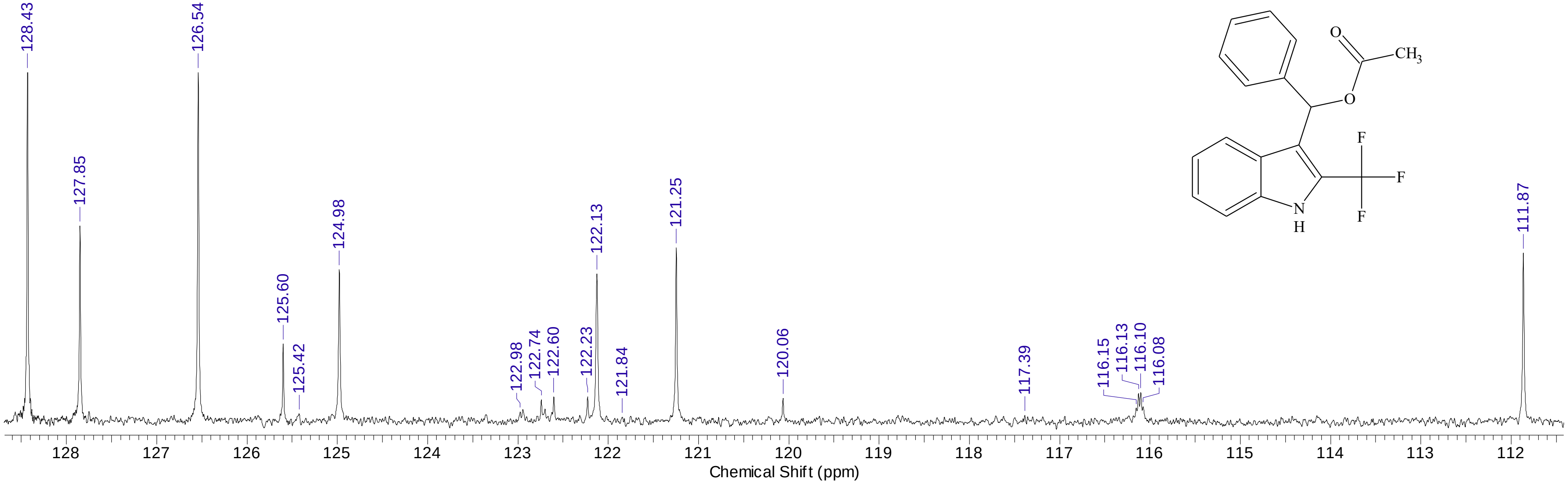

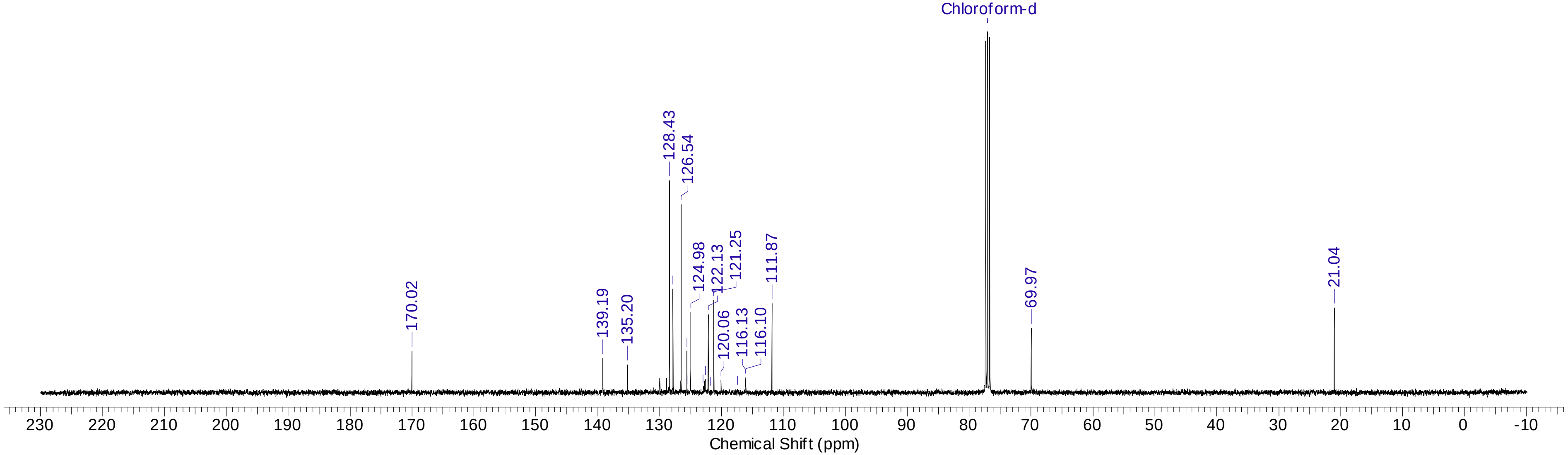

$^{13}C\{^{1}H\}$ NMR spectrum of **5a** (100.6 MHz, $CDCl_3$)

9 Jun 2021

Acquisition Time (sec)	4.0894	***Comment***	Imported from UXNMR.	***Date***	06 Nov 2020 15:46:12
File Name	C:\DOCS\OUTPUT_301\2020\11.眍 狃黒BM-1950.H_001001r			***Frequency (MHz)***	400.13
Nucleus	1H	***Number of Transients*** 4	***Original Points Count*** 32768	***Points Count***	131072
Pulse Sequence	zg30	***Solvent***	CHLOROFORM-D	***Sweep Width (Hz)***	8012.82
Temperature (degree C)	27.000				

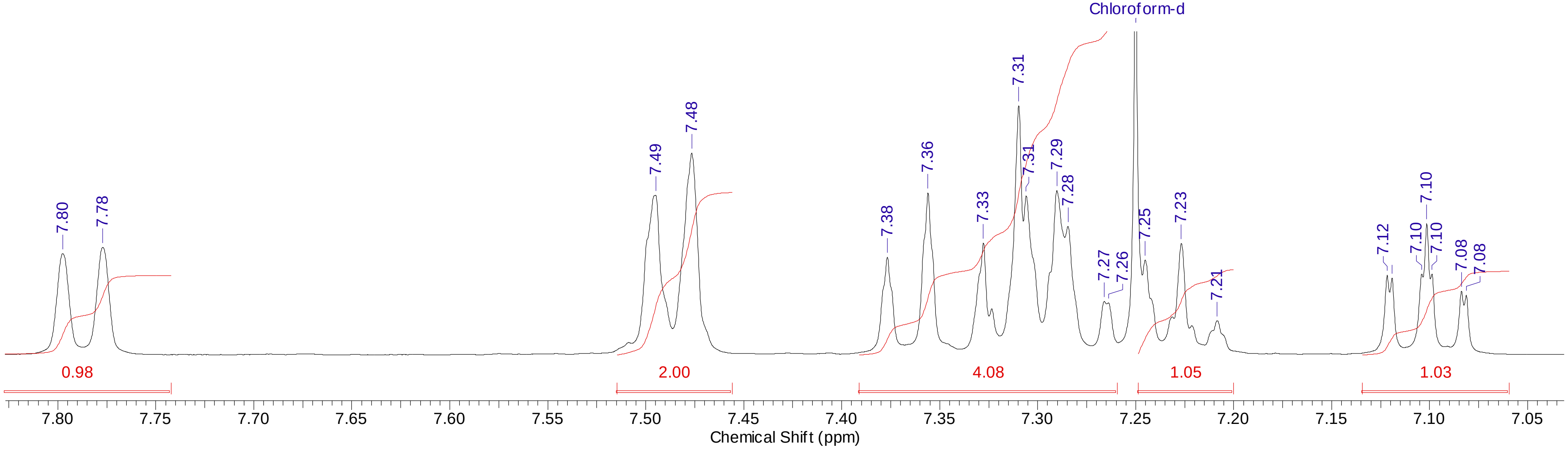

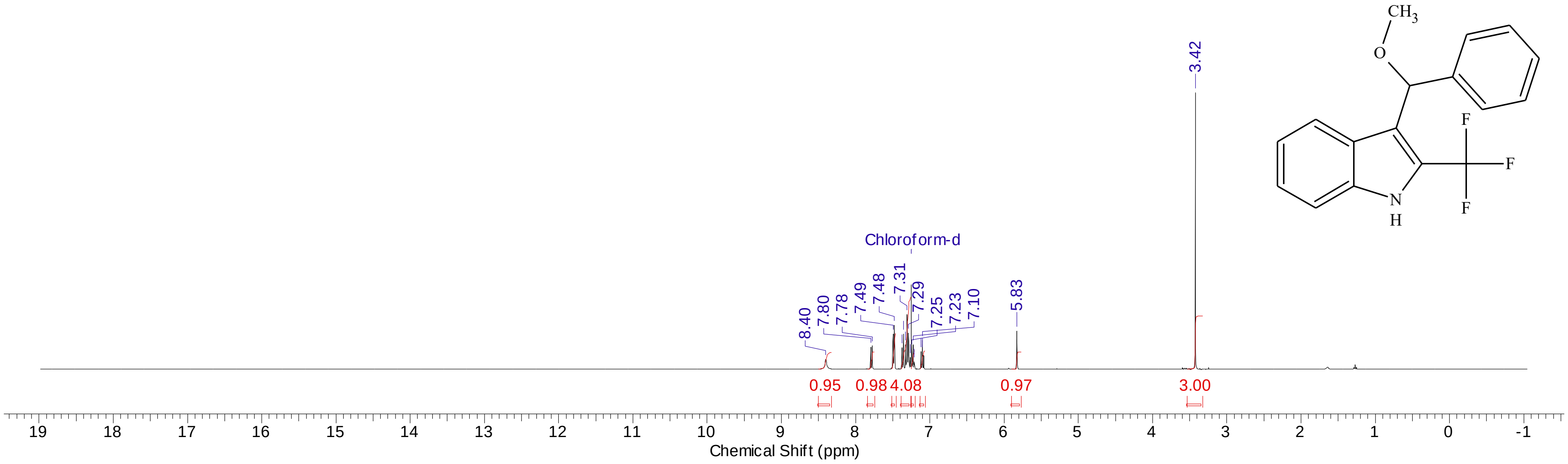

^{1}H NMR spectrum of **6a** (400.1 MHz, $CDCl_3$)

9 Jun 2021

Acquisition Time (sec)	1.0000	***Date***	Nov 9 2020	***File Name***	C:\DOCS\OUTPUT_301\F19\2020.11.09\BM-1950_20201109_01\FLUORINE_01		
Frequency (MHz)	376.31	***Nucleus***	19F	***Number of Transients***	16	***Original Points Count***	89286
Points Count	131072	***Pulse Sequence***	s2pul	***Solvent***	CHLOROFORM-D		
Sweep Width (Hz)	89285.71	***Temperature (degree C)***	24.000				

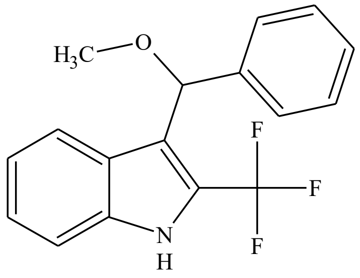

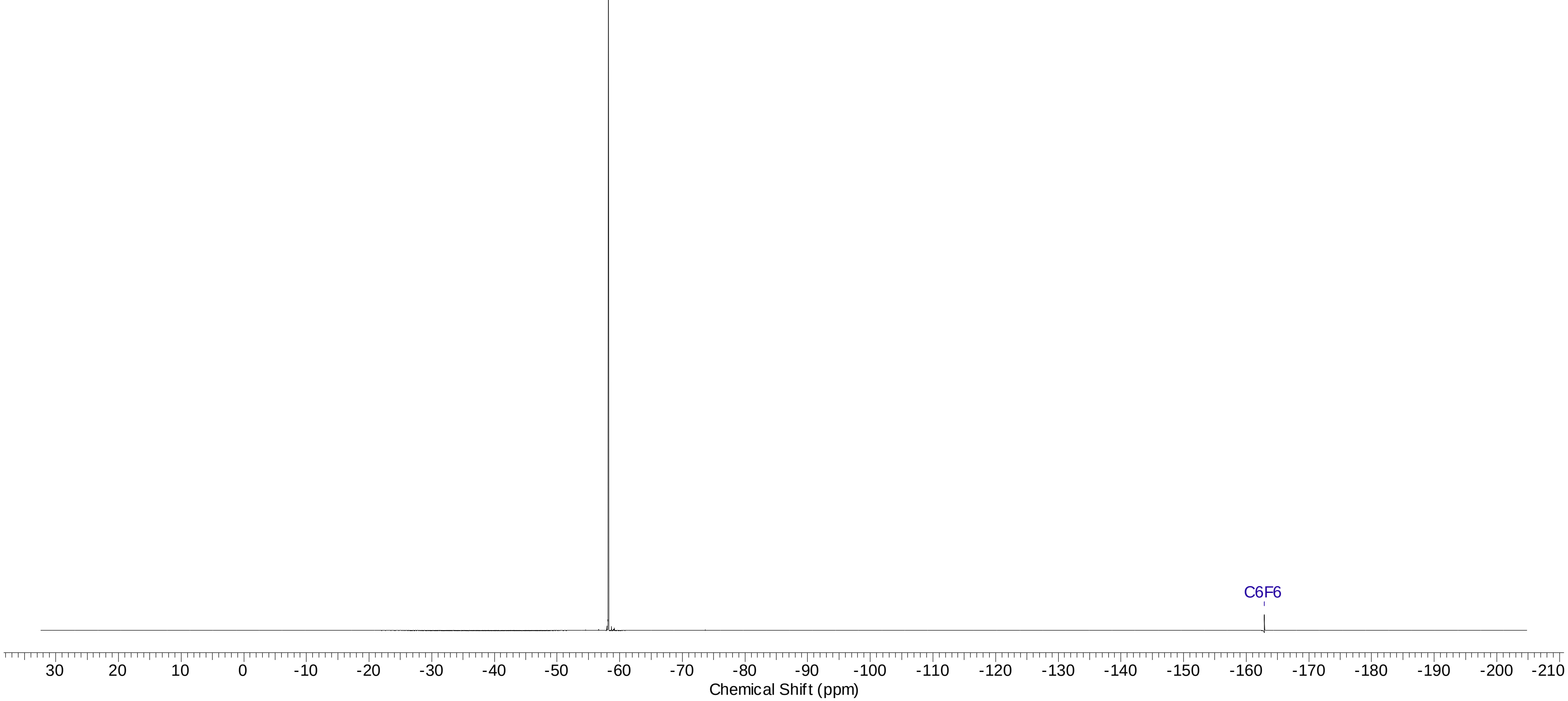

^{19}F NMR spectrum of **6a** (376.5 MHz, $CDCl_3$)

**Acquisition Time (sec)**	0.6783	**Comment**	Imported from UXNMR.			**Date**	07 Nov 2020 13:25:40
**File Name**	C:\DOCS\BM\BM_spectra\BM-1950_002001r			**Frequency (MHz)**	100.61	**Nucleus**	13C
**Number of Transients**	1272	**Original Points Count**	16384	**Points Count**	131072	**Pulse Sequence**	zgpg30
**Solvent**	CHLOROFORM-D			**Sweep Width (Hz)**	24154.59	**Temperature (degree C)**	27.000

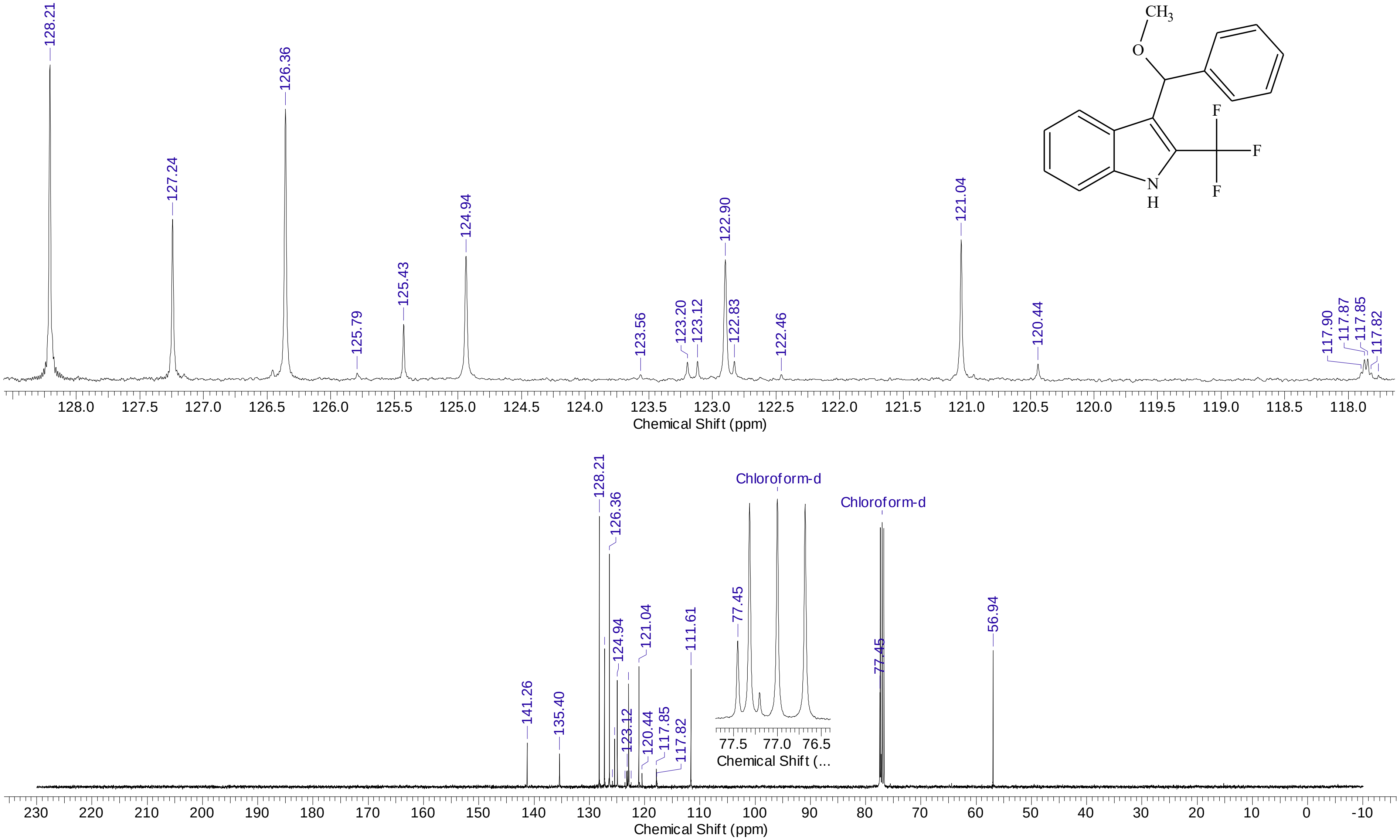

$^{13}C\{^1H\}$ NMR spectrum of **6a** (100.6 MHz, $CDCl_3$)

19 Jun 2020

Acquisition Time (sec)	4.0894	Comment	Imported from UXNMR.			Date	03 Dec 2019 14:09:50
File Name	C:\DOCS\OUTPUT_301\2019\12.溴贬狃黒BM-1802-2.H_001001r					Frequency (MHz)	400.13
Nucleus	1H	Number of Transients	4	Original Points Count	32768	Points Count	131072
Pulse Sequence	zg30	Solvent	CHLOROFORM-D			Sweep Width (Hz)	8012.82
Temperature (degree C)	27.000						

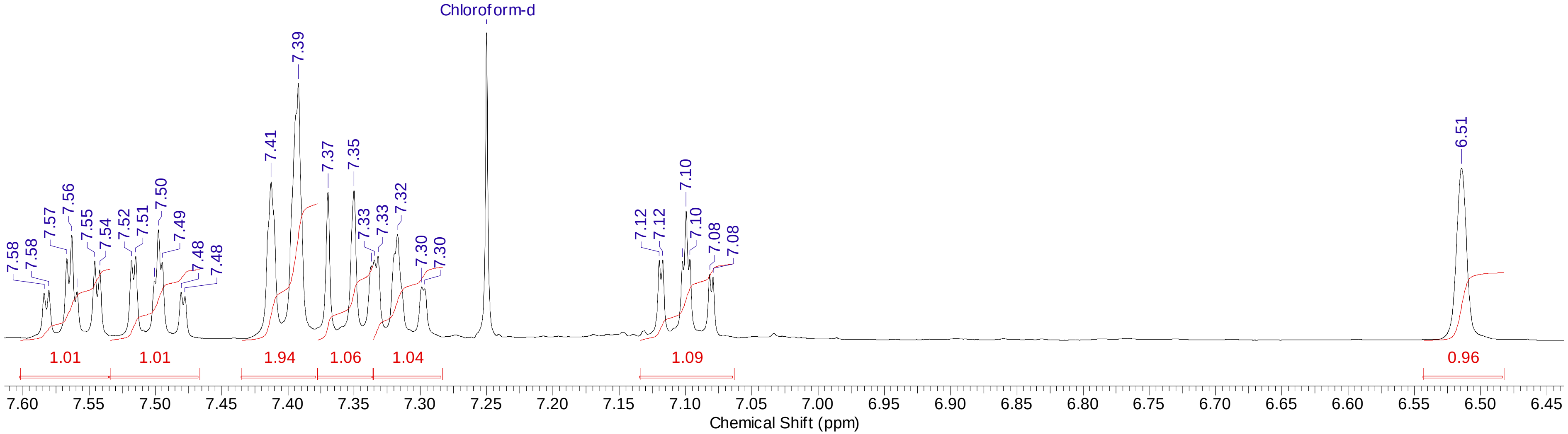

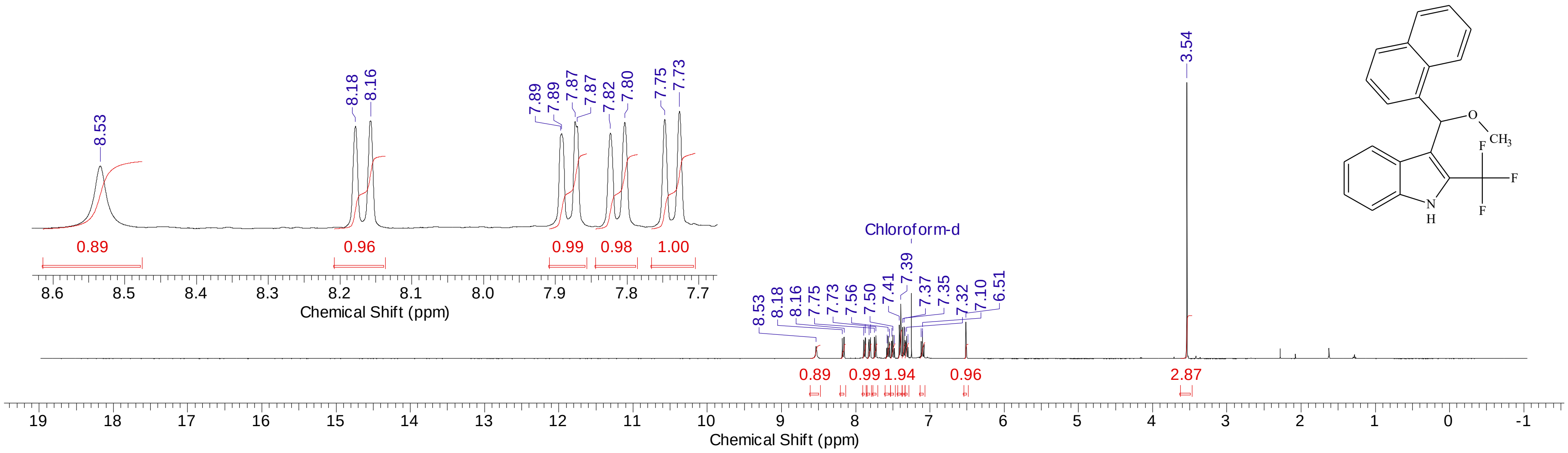

^{1}H NMR spectrum of **6b** (400.1 MHz, $CDCl_3$)

19 Jun 2020

Acquisition Time (sec)	2.0000	***Date***	Dec 2 2019	***File Name***	C:\DOCS\OUTPUT_301\F19\2019.12.02\BM-1802-2_20191202_01\FLUORINE_01		
Frequency (MHz)	376.31	***Nucleus***	19F	***Number of Transients***	16	***Original Points Count***	178571
Points Count	262144	***Pulse Sequence***	s2pul	***Solvent***	CHLOROFORM-D		
Sweep Width (Hz)	89285.71	***Temperature (degree C)***	22.000				

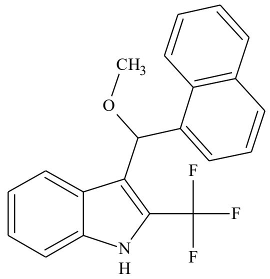

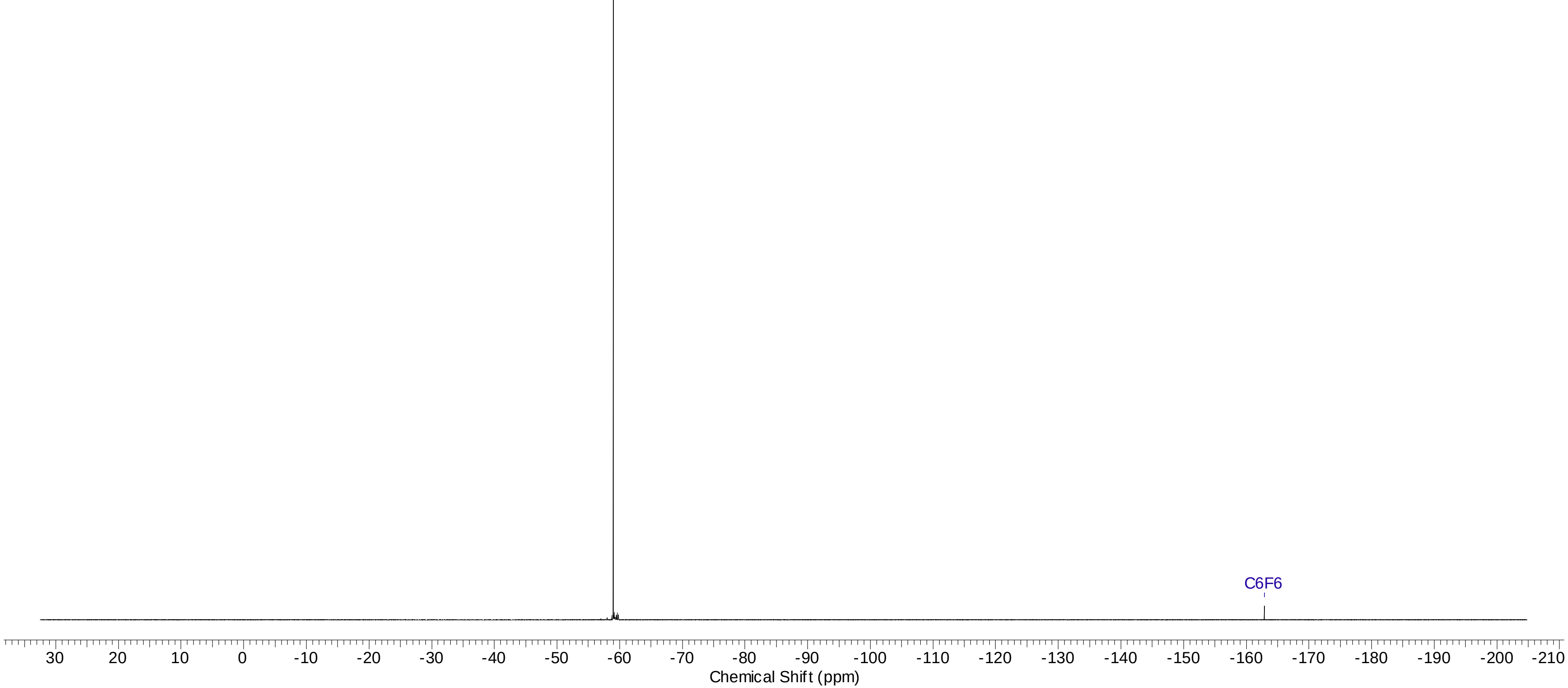

^{19}F NMR spectrum of **6b** (376.5 MHz, $CDCl_3$)

19 Jun 2020

Acquisition Time (sec)	0.6783	***Comment***	Imported from UXNMR.		***Date***	03 Dec 2019 14:08:24
File Name	C:\DOCS\OUTPUT_301\2019\12.溴贬狃黒BM-1802-2.C_002001r				***Frequency (MHz)***	100.61
Nucleus	13C	***Number of Transients***	572	***Original Points Count*** 16384	***Points Count***	131072
Pulse Sequence	zgpg30	***Solvent***	CHLOROFORM-D		***Sweep Width (Hz)***	24154.59
Temperature (degree C)	27.000					

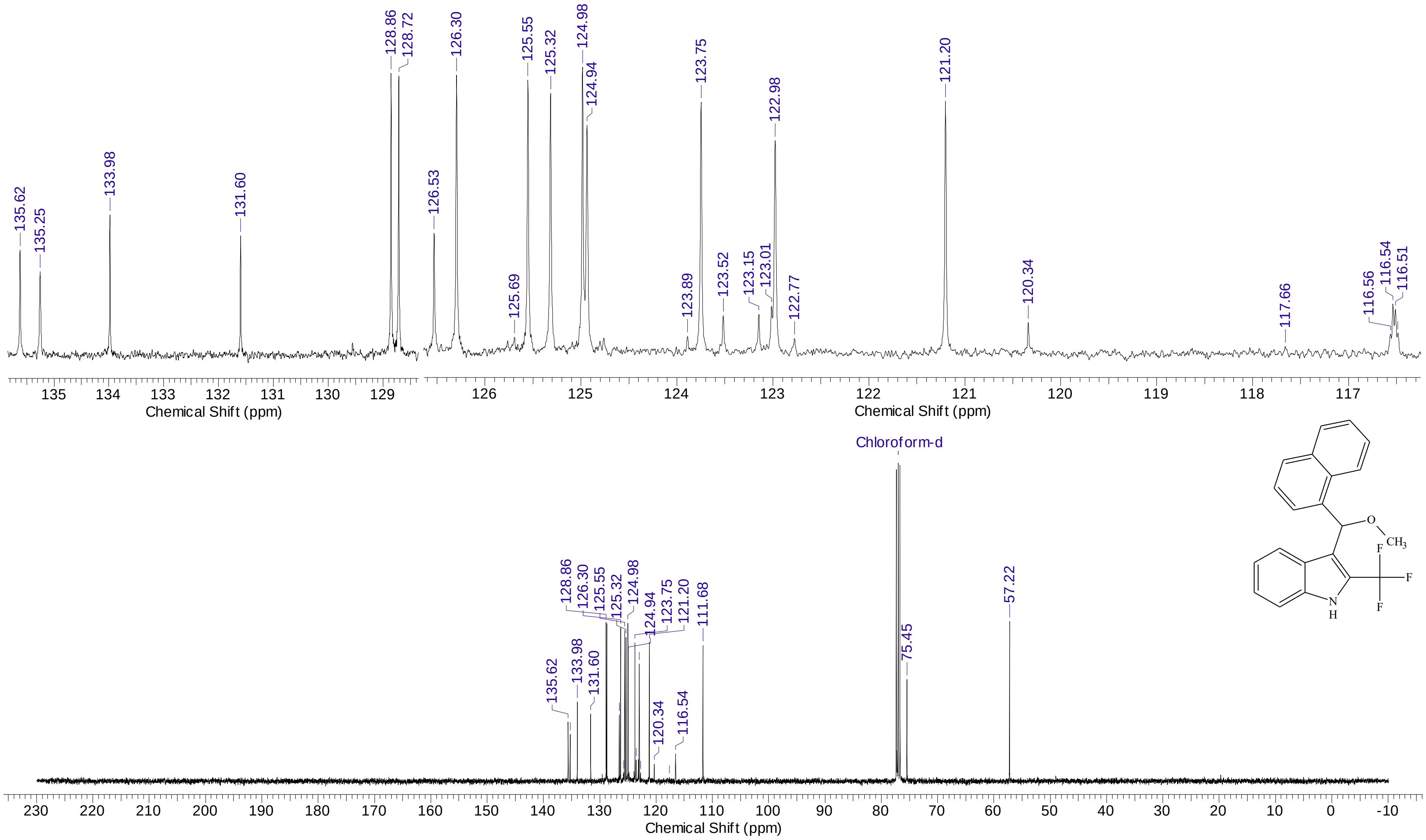

$^{13}C\{^{1}H\}$ NMR spectrum of **6b** (100.6 MHz, $CDCl_3$)

3 Jul 2021

Acquisition Time (sec)	4.0894	***Comment***	Imported from UXNMR.			***Date***	17 Mar 2021 17:45:46
File Name	C:\DOCS\OUTPUT_301\2021\03.爨 痱\BM-2108-2.H_001001r					***Frequency (MHz)***	400.13
Nucleus	1H	***Number of Transients***	4	***Original Points Count***	32768	***Points Count***	131072
Pulse Sequence	zg30	***Solvent***	CHLOROFORM-D			***Sweep Width (Hz)***	8012.82
Temperature (degree C)	27.000						

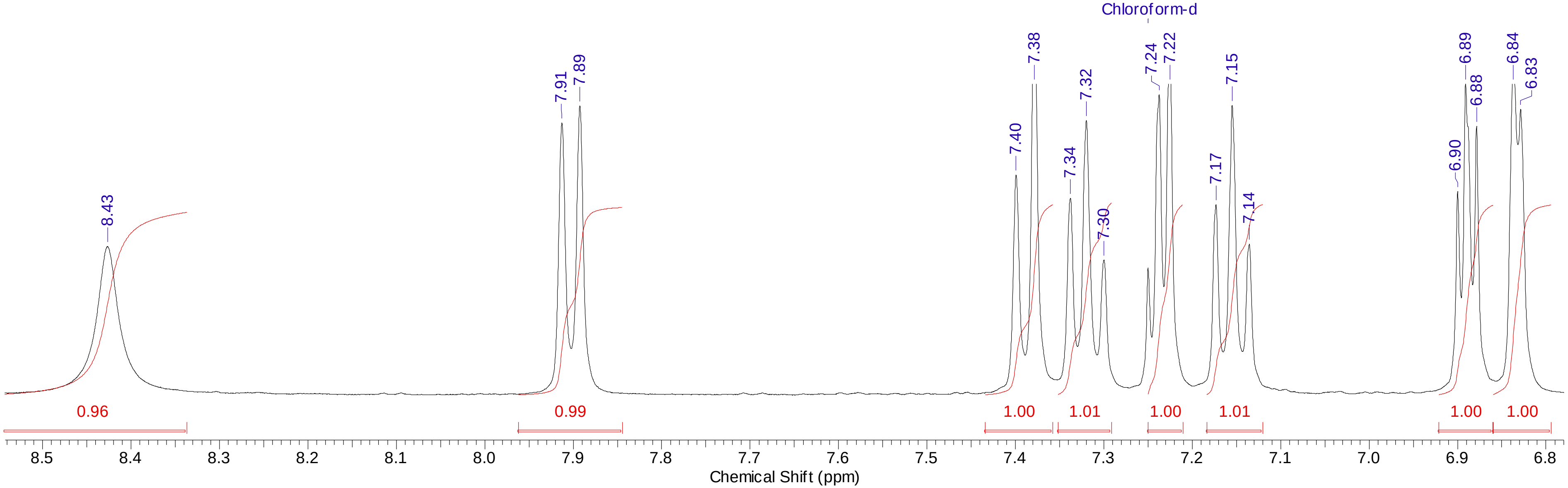

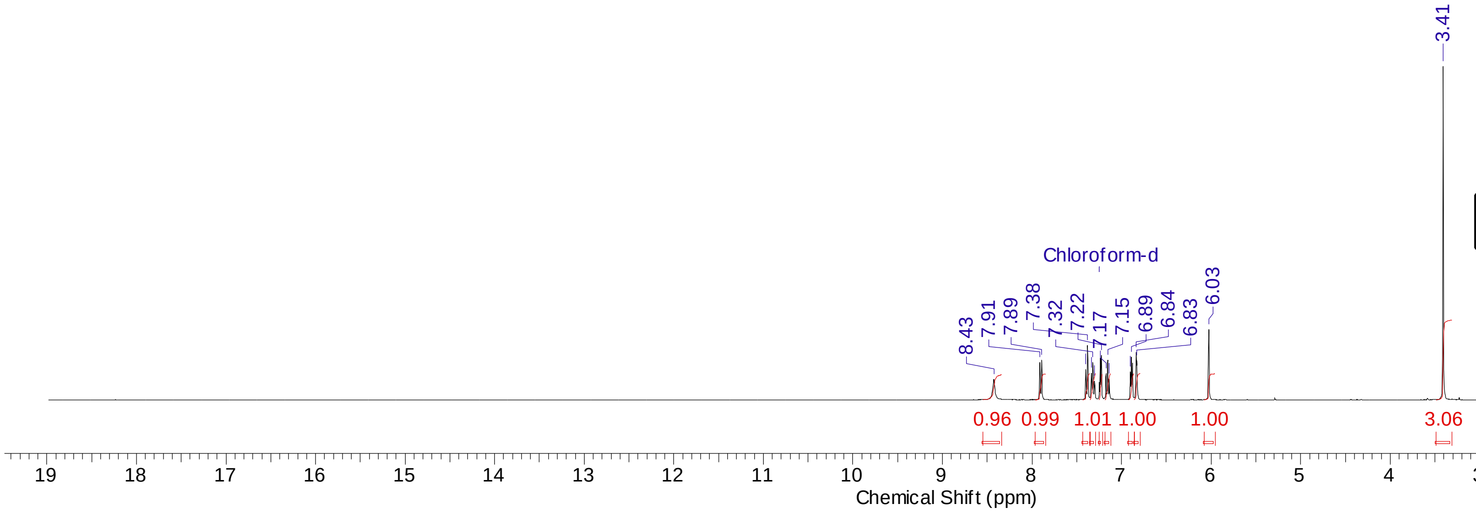

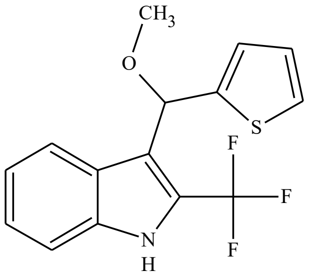

^{1}H NMR spectrum of **6c** (400.1 MHz, $CDCl_3$)

3 Jul 2021

Acquisition Time (sec)	1.7433	***Comment***	Imported from UXNMR.			***Date***	18 Mar 2021 12:59:50
File Name	C:\DOCS\OUTPUT_301\2021\03.爨 痱\BM-2108-2.F_005001r			***Frequency (MHz)***	376.50	***Nucleus***	19F
Number of Transients	11	***Original Points Count***	131072	***Points Count***	262144	***Pulse Sequence***	zgflqn
Solvent	CHLOROFORM-D			***Sweep Width (Hz)***	75187.97	***Temperature (degree C)***	27.000

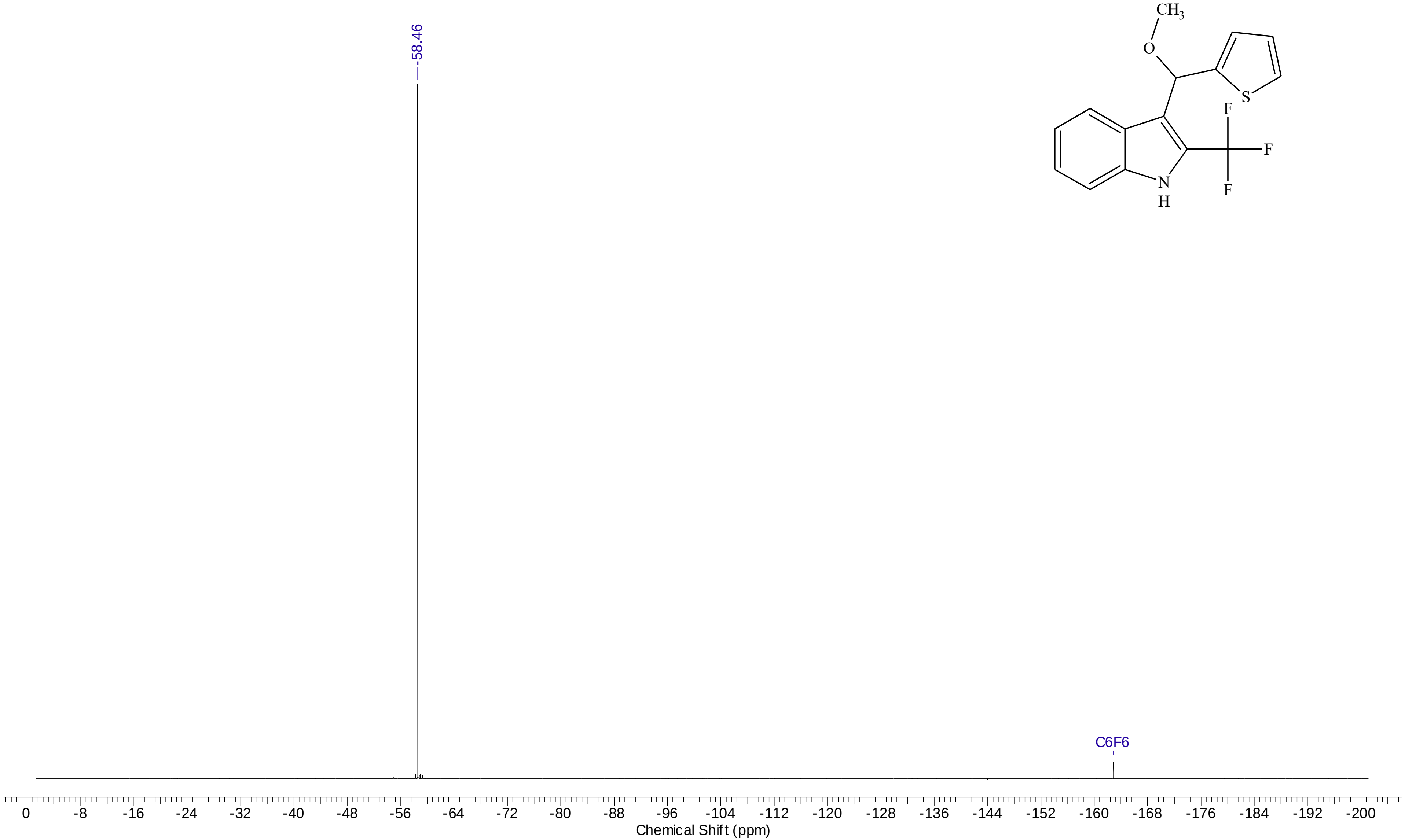

^{19}F NMR spectrum of **6c** (376.5 MHz, $CDCl_3$)

3 Jul 2021

Acquisition Time (sec)	0.6783	***Comment***	Imported from UXNMR.			***Date***	18 Mar 2021 12:43:52
File Name	C:\DOCS\OUTPUT_301\2021\03.釁 痱\BM-2108-2.C_002001r					***Frequency (MHz)***	100.61
Nucleus	13C	***Number of Transients***	265	***Original Points Count***	16384	***Points Count***	131072
Pulse Sequence	zgpg30	***Solvent***	DMSO-D6	***Sweep Width (Hz)***	24154.59	***Temperature (degree C)***	27.000

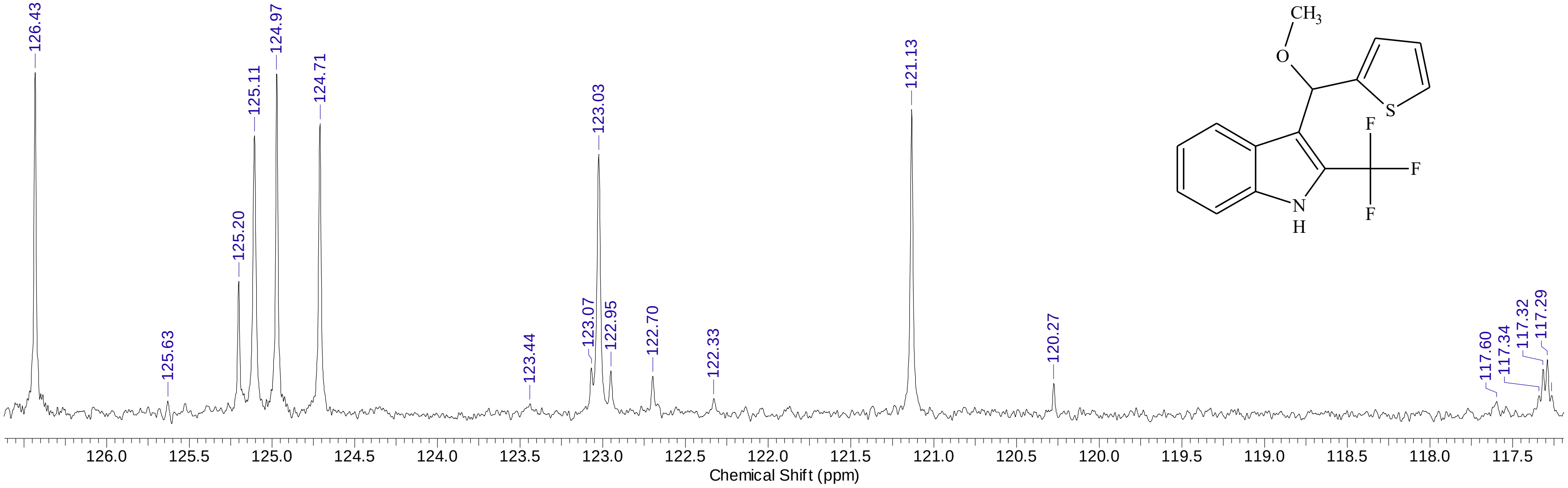

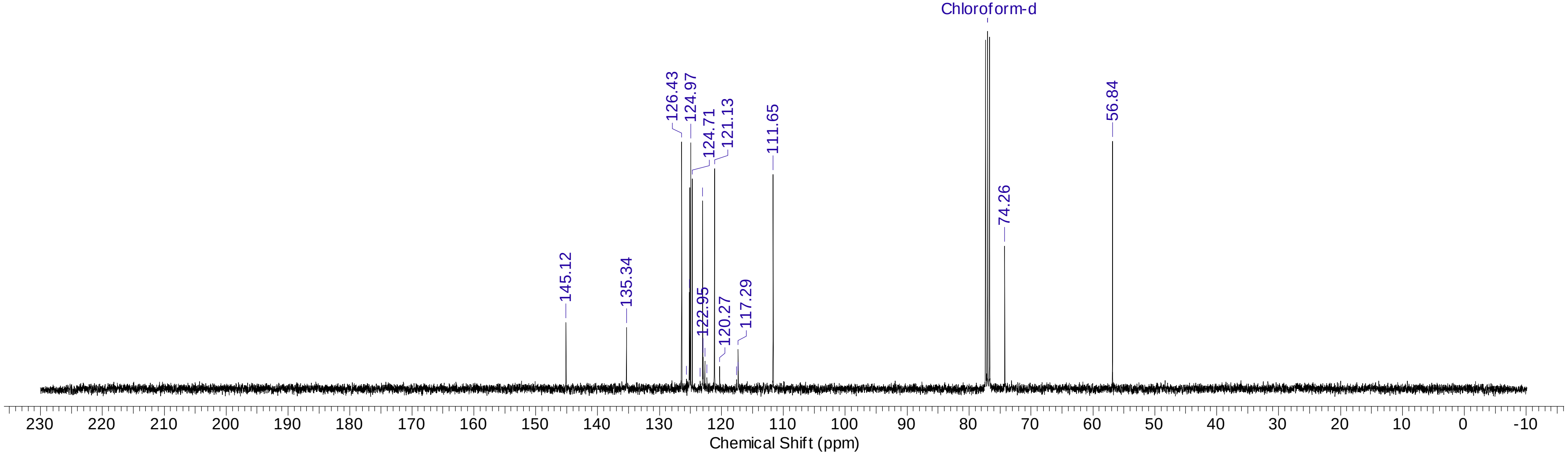

$^{13}C\{^{1}H\}$ NMR spectrum of **6c** (100.6 MHz, $CDCl_3$)

9 Jun 2021

Acquisition Time (sec)	4.0894	***Comment***	Imported from UXNMR.			***Date***	22 Dec 2020 15:18:22
File Name	C:\DOCS\OUTPUT_301\2020\12.溴赅狃黑BM-2023-R.H_001001r					***Frequency (MHz)***	400.13
Nucleus	1H	***Number of Transients***	4	***Original Points Count***	32768	***Points Count***	131072
Pulse Sequence	zg30	***Solvent***	CHLOROFORM-D			***Sweep Width (Hz)***	8012.82
Temperature (degree C)	27.000						

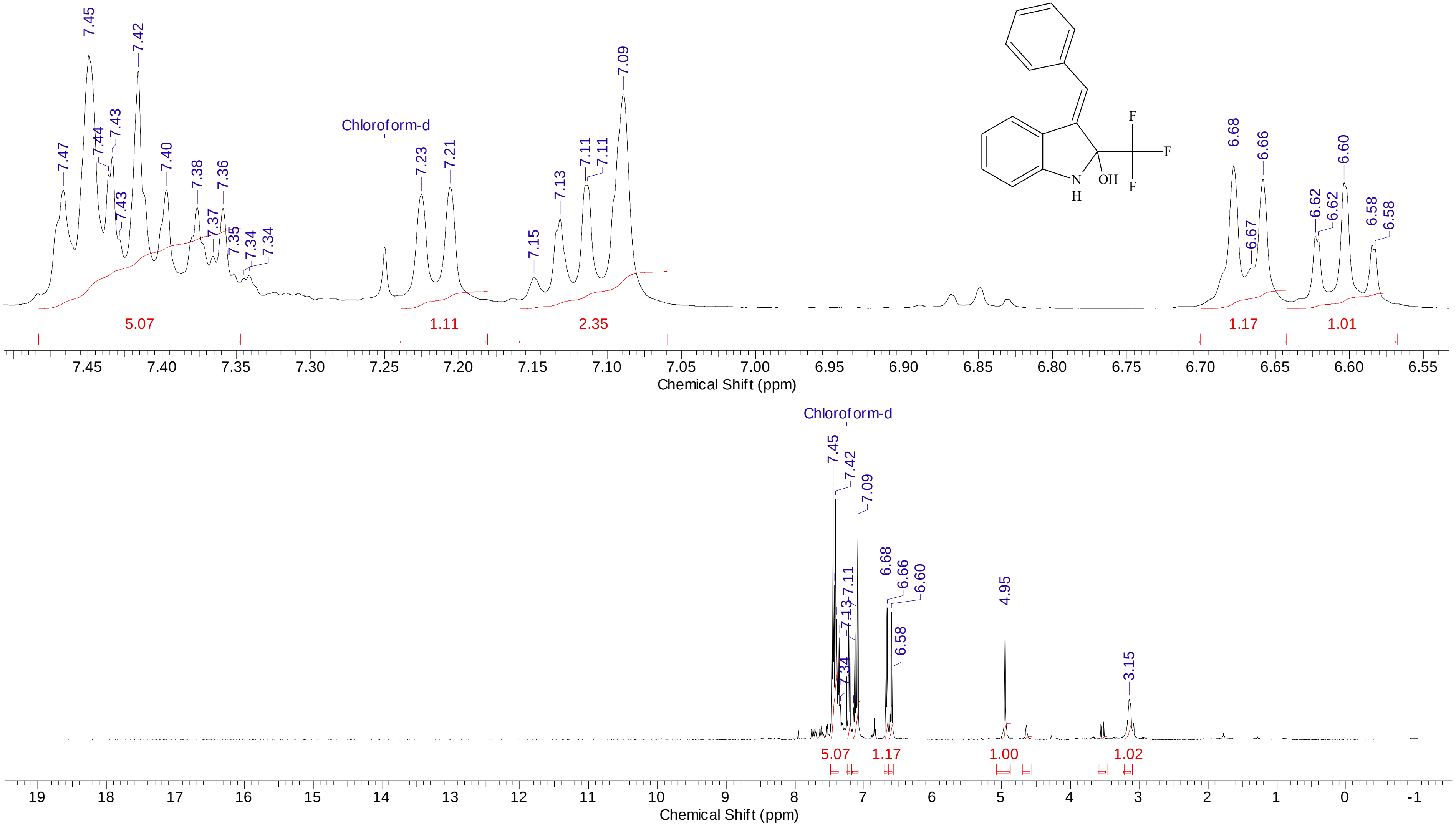

^{1}H NMR spectrum of **B** (400.1 MHz, $CDCl_3$)

9 Jun 2021

Acquisition Time (sec)	1.0000	***Comment***	STANDARD FLUORINE PARAMETERS			***Date***	Dec 18 2020
File Name	C:\DOCS\OUTPUT_301\F19\2020.12.18\BM-2023-R_20201218_01\FLUORINE_01					***Frequency (MHz)***	376.31
Nucleus	19F	***Number of Transients***	16	***Original Points Count***	89286	***Points Count***	131072
Pulse Sequence	s2pul	***Solvent***	CHLOROFORM-D	***Sweep Width (Hz)***	89285.71	***Temperature (degree C)***	24.000

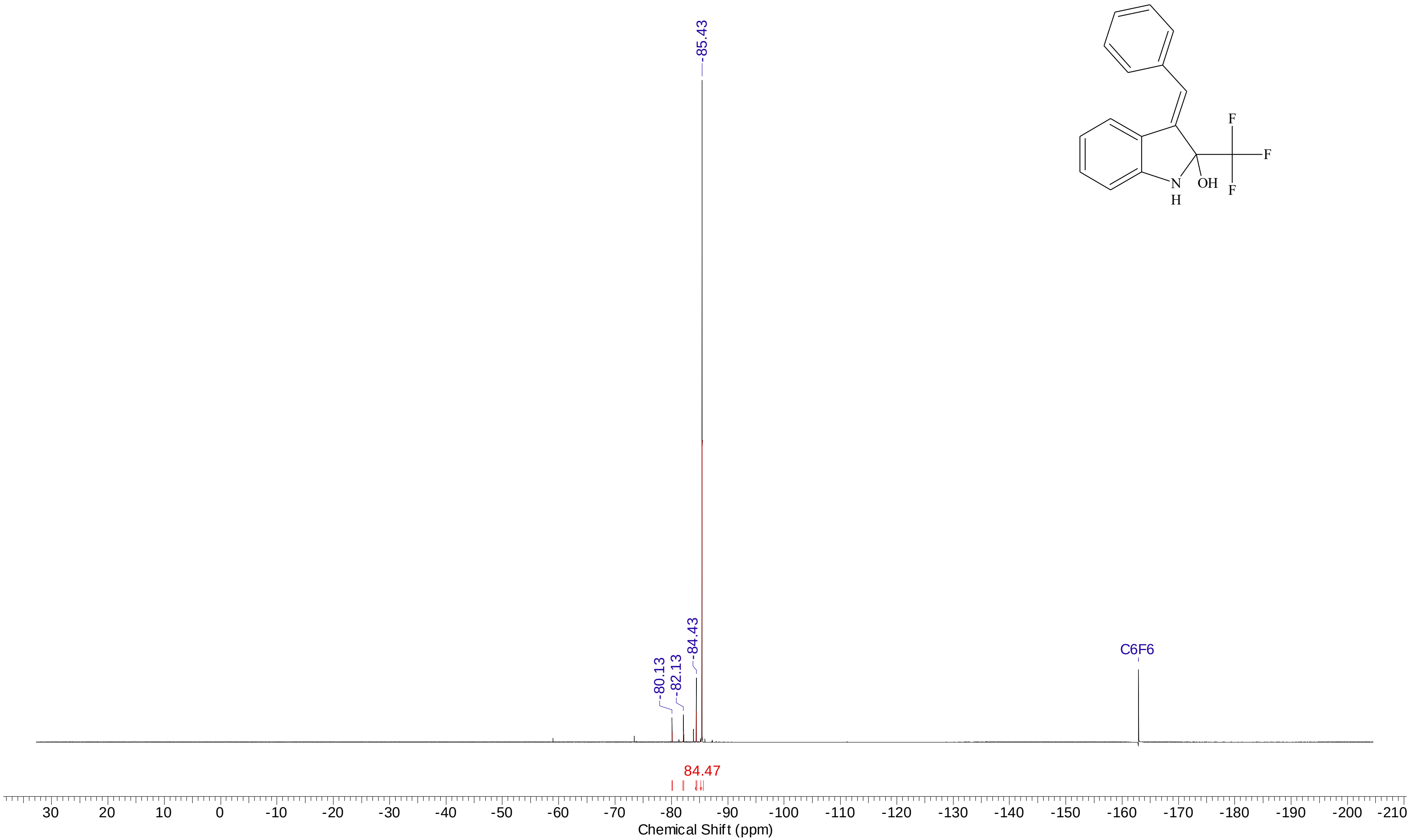

^{19}F NMR spectrum of **B** (376.5 MHz, $CDCl_3$)

Acquisition Time (sec)	0.6783	***Comment***	Imported from UXNMR.			***Date***	22 Dec 2020 15:27:46
File Name	C:\DOCS\OUTPUT_301\2020\12.溴赅狃黒BM-2023-R.C_002001r					***Frequency (MHz)***	100.61
Nucleus	13C	***Number of Transients***	233	***Original Points Count***	16384	***Points Count***	131072
Pulse Sequence	zgpg30	***Solvent***	DMSO-D6	***Sweep Width (Hz)***	24154.59	***Temperature (degree C)***	27.000

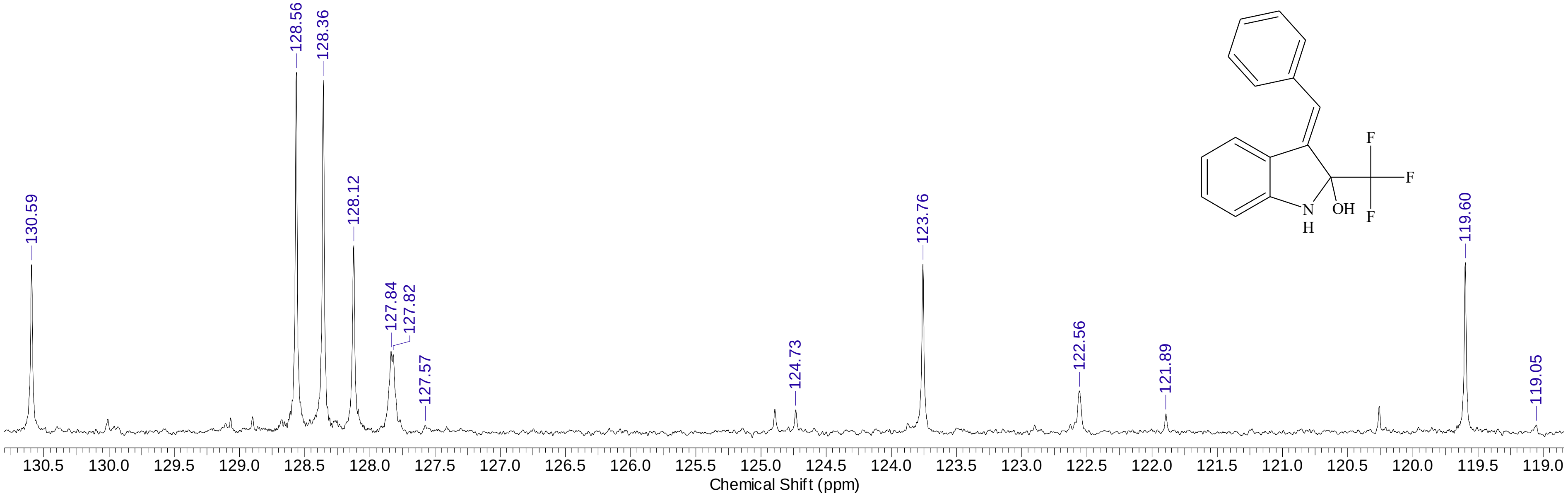

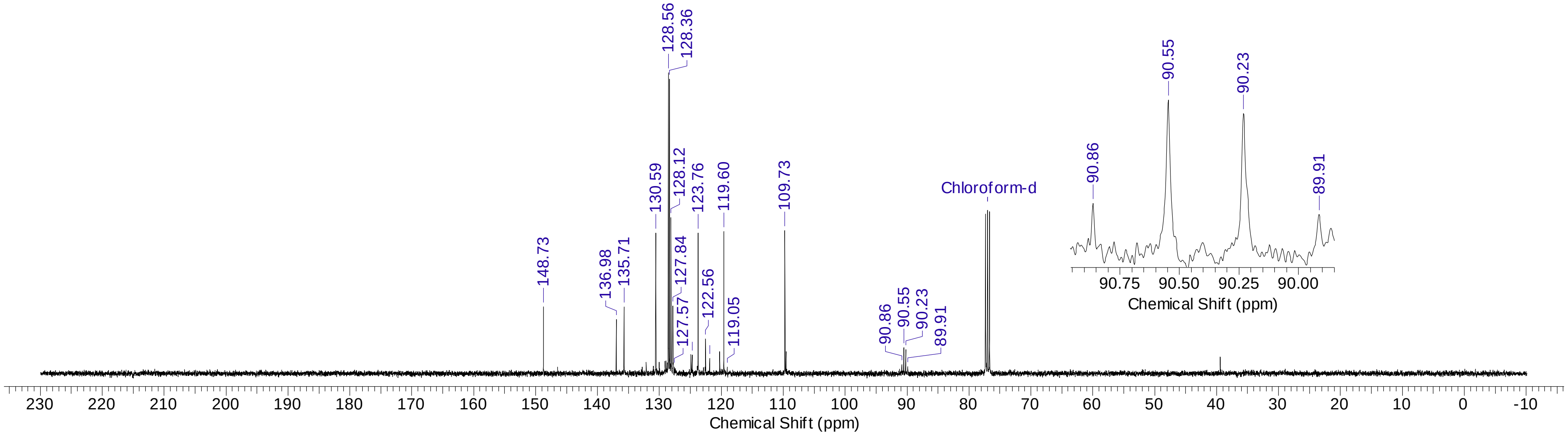

$^{13}C\{^{1}H\}$ NMR spectrum of **B** (100.6 MHz, $CDCl_3$)

9 Jun 2021

Acquisition Time (sec)	4.0894	***Comment***	Imported from UXNMR.			***Date***	27 Jan 2021 17:23:32
File Name	C:\DOCS\OUTPUT_301\2021\01. 磤囵黒BM-2023-R-21.H_001001r					***Frequency (MHz)***	400.13
Nucleus	1H	***Number of Transients***	4	***Original Points Count***	32768	***Points Count***	131072
Pulse Sequence	zg30	***Solvent***	CHLOROFORM-D			***Sweep Width (Hz)***	8012.82
Temperature (degree C)	27.000						

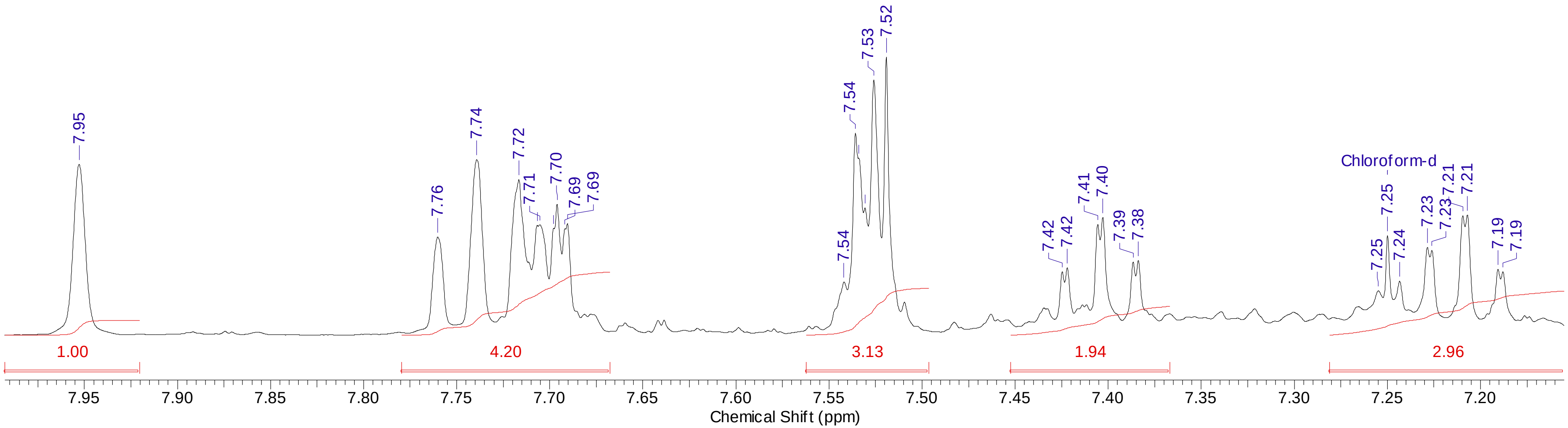

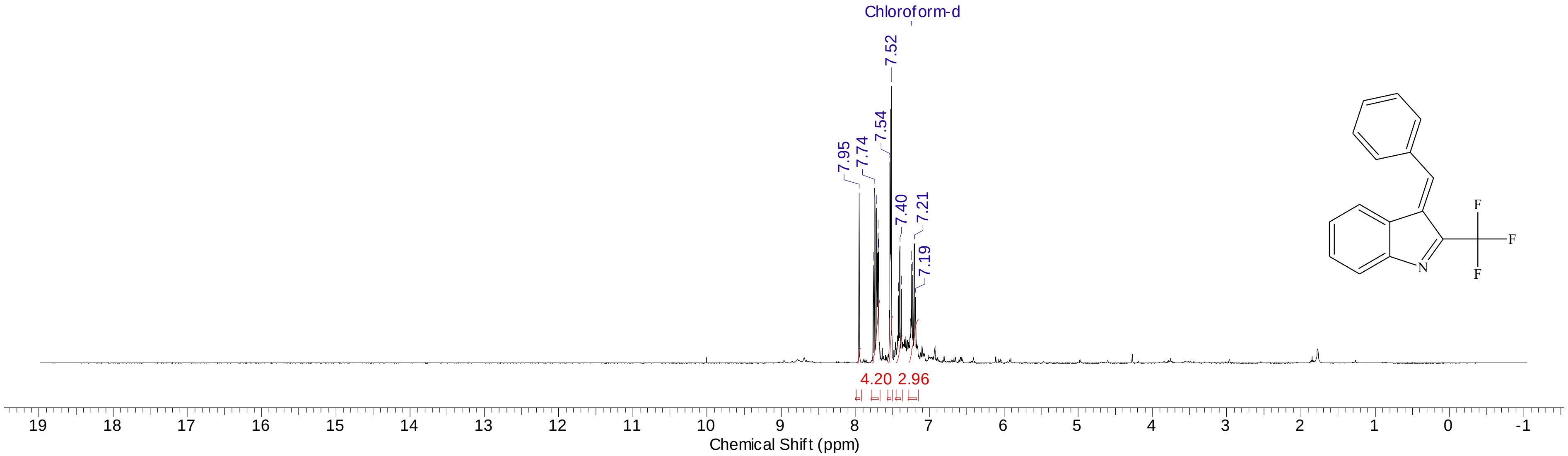

^{1}H NMR spectrum of **C** (400.1 MHz, $CDCl_3$)

9 Jun 2021

Acquisition Time (sec)	1.7433	***Comment***	Imported from UXNMR.	***Date***	26 Jan 2021 15:17:26
File Name	C:\DOCS\OUTPUT_301\2021\01. 磁圀黒BM-2023-R-21.F_005001r			***Frequency (MHz)***	376.50
Nucleus	19F	***Number of Transients***	16	***Original Points Count*** 131072	***Points Count*** 262144
Pulse Sequence	zgflqn	***Solvent***	CHLOROFORM-D	***Sweep Width (Hz)***	75187.97
Temperature (degree C)	27.000				

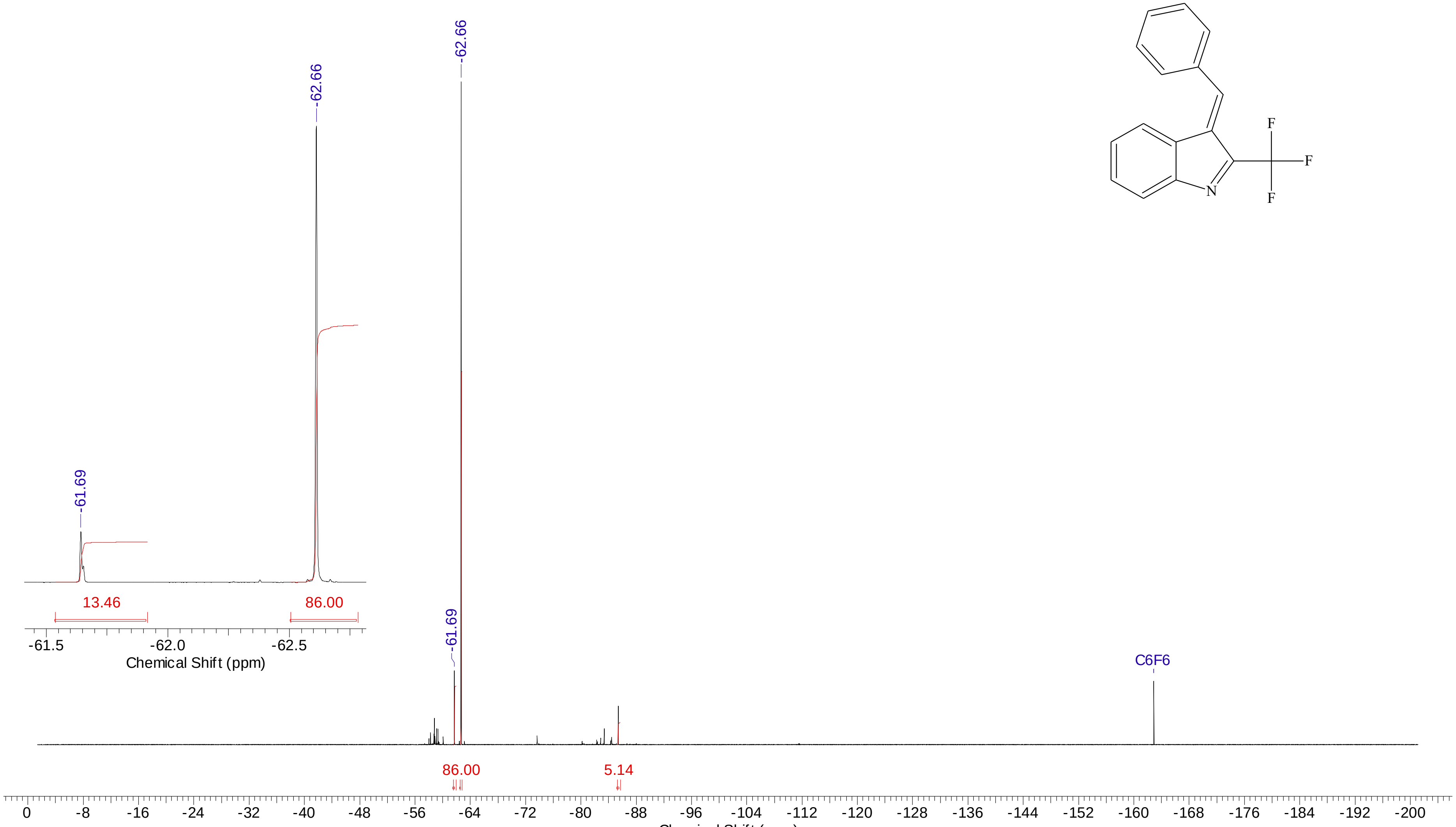

^{19}F NMR spectrum of **C** (376.5 MHz, $CDCl_3$)

9 Jun 2021

Acquisition Time (sec)	0.6783	Comment	Imported from UXNMR.			Date	27 Jan 2021 17:35:28
File Name	C:\DOCS\OUTPUT_301\2021\01. 礅囵黒BM-2023-R-21.C_002001r					Frequency (MHz)	100.61
Nucleus	13C	Number of Transients	241	Original Points Count	16384	Points Count	131072
Pulse Sequence	zgpg30	Solvent	DMSO-D6	Sweep Width (Hz)	24154.59	Temperature (degree C)	27.000

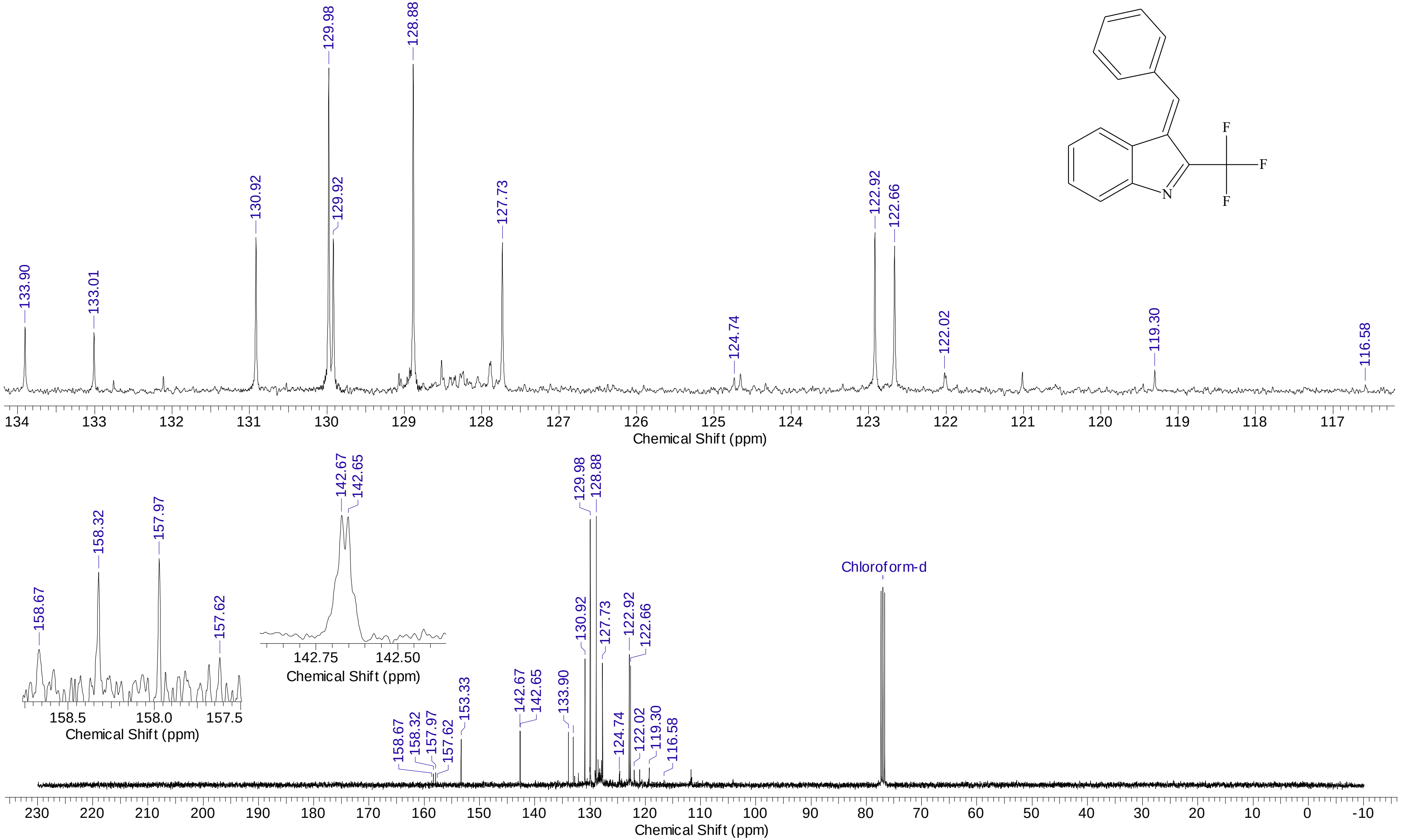

$^{13}C\{^{1}H\}$ NMR spectrum of **C** (100.6 MHz, $CDCl_3$)

9 Jun 2021

**Acquisition Time (sec)**	1.3664	**Comment**	Imported from UXNMR.	**Date**	28 Jan 2021 12:18:36
**File Name**	C:\DOCS\OUTPUT_301\2021\01. 礅囧黒BM-2023-R-21.APT_004001r			**Frequency (MHz)**	100.61
**Nucleus**	13C	**Number of Transients**	109 **Original Points Count** 32768	**Points Count**	131072
**Pulse Sequence**	jmod	**Solvent**	CHLOROFORM-D	**Sweep Width (Hz)**	23980.81
**Temperature (degree C)**	27.000				

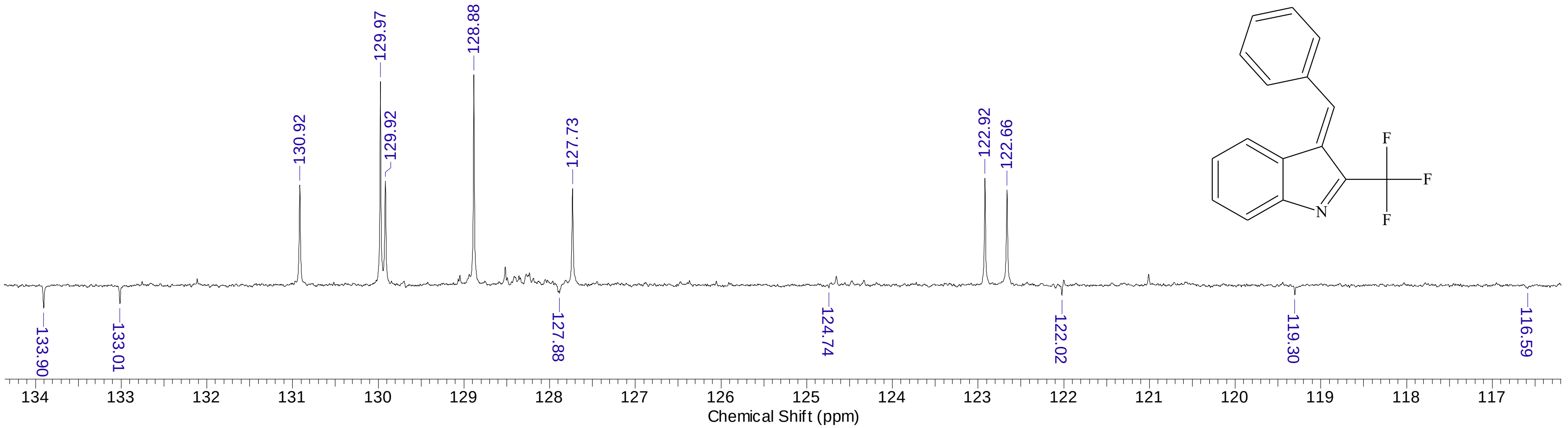

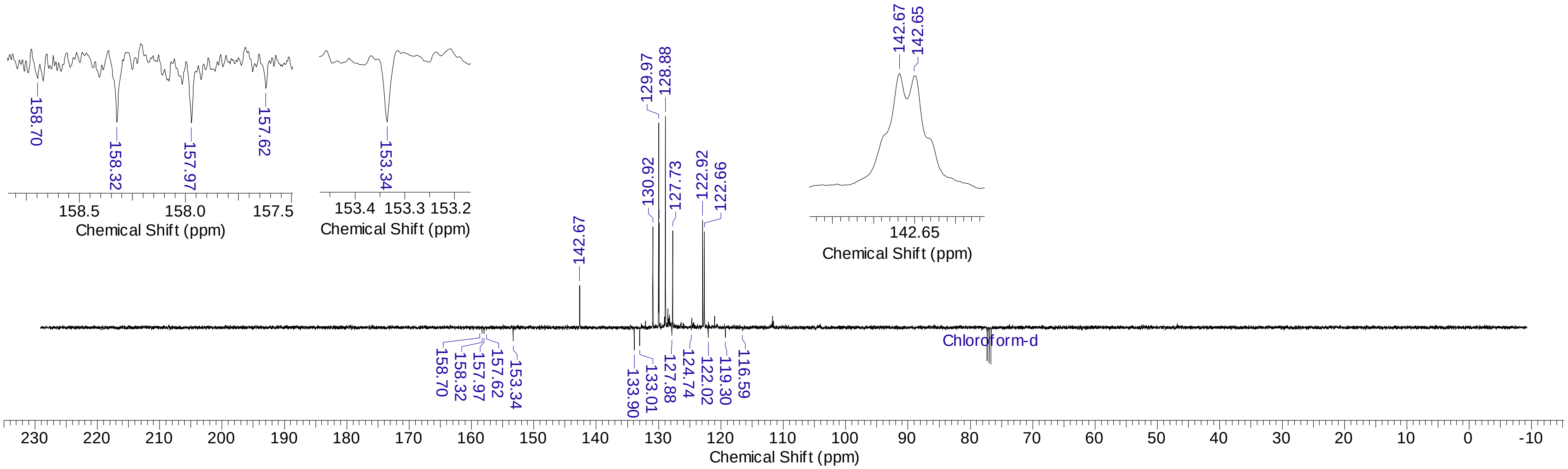

$^{13}C\{^{1}H\}$ APT NMR spectrum of **C** (100.6 MHz, $CDCl_3$)

19 Jun 2020

FW	568.5521	***Formula***	$C_{32}H_{26}F_6N_2O$

Acquisition Time (sec)	4.0894	***Comment***	Imported from UXNMR.			***Date***	02 Dec 2019 15:59:26
File Name	C:\DOCS\OUTPUT_301\2019\12.溴赅狃黑BM-1816-R.H_001001r					***Frequency (MHz)***	400.13
Nucleus	1H	***Number of Transients***	6	***Original Points Count***	32768	***Points Count***	131072
Pulse Sequence	zg30	***Solvent***	CHLOROFORM-D			***Sweep Width (Hz)***	8012.82
Temperature (degree C)	27.000						

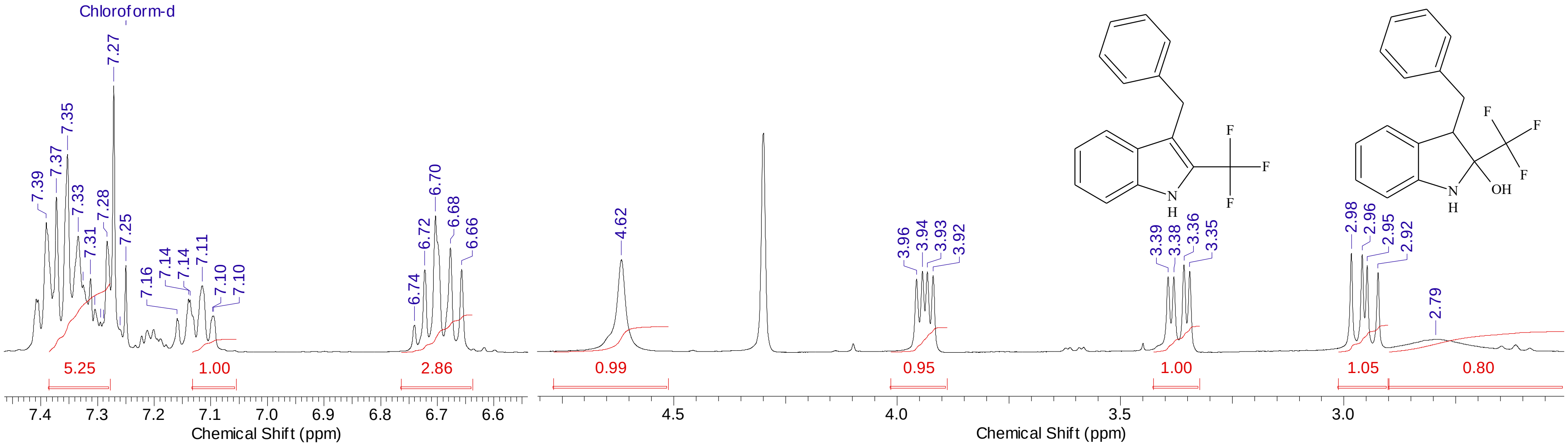

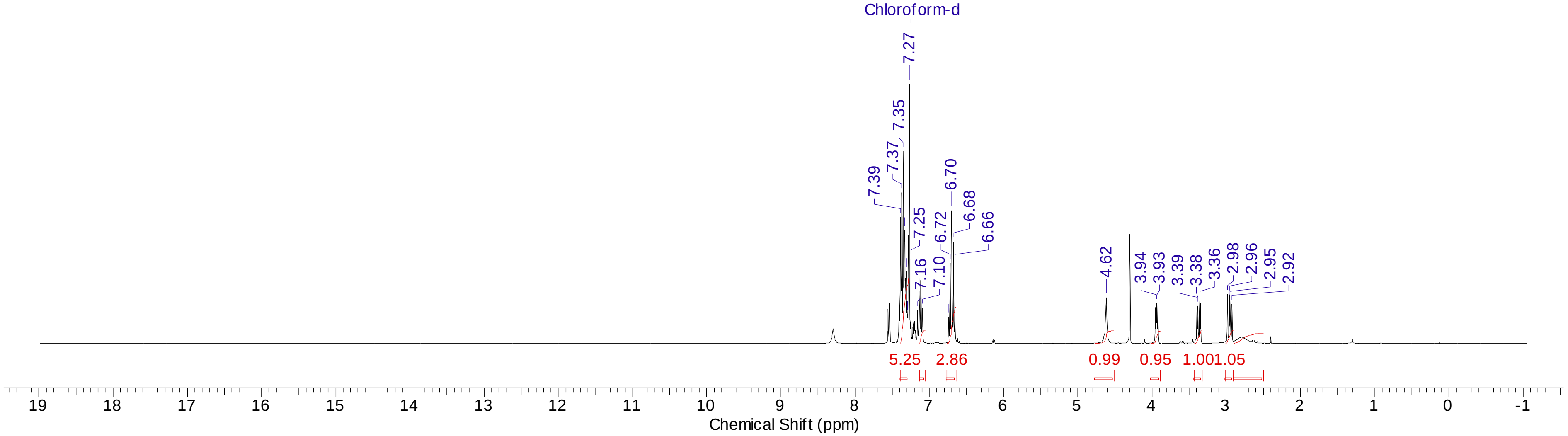

^1H NMR spectrum of **D** (400.1 MHz, $CDCl_3$)

19 Jun 2020

FW	568.5521	Formula	$C_{32}H_{26}F_6N_2O$

Acquisition Time (sec)	0.7340	Date	Dec 3 2019	File Name	C:\DOCS\OUTPUT_301\F19\2019.12.03\bm1816-r-f_20191203_01\FLUORINE_01		
Frequency (MHz)	376.31	Nucleus	19F	Number of Transients	100	Original Points Count	65536
Points Count	65536	Pulse Sequence	s2pul	Solvent	CHLOROFORM-D		
Sweep Width (Hz)	89285.71	Temperature (degree C)	22.000				

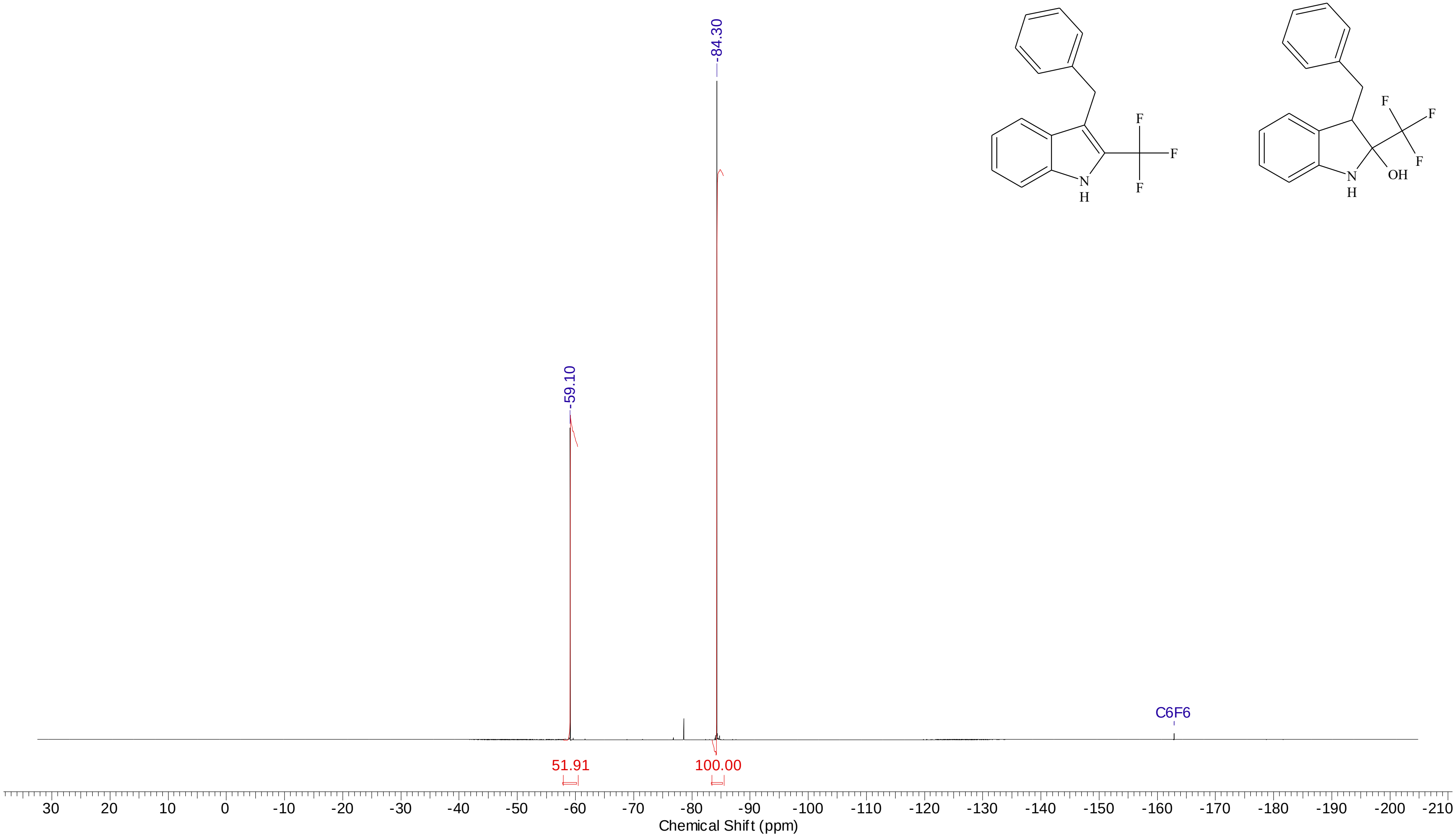

^{19}F NMR spectrum of **D** (376.5 MHz, $CDCl_3$)

19 Jun 2020

FW	568.5521	Formula	$C_{32}H_{26}F_6N_2O$

Acquisition Time (sec)	0.6783	Comment	Imported from UXNMR.			Date	02 Dec 2019 16:05:14
File Name	C:\DOCS\OUTPUT_301\2019\12.溴赅狃黒BM-1816-R.C_002001r					Frequency (MHz)	100.61
Nucleus	13C	Number of Transients	137	Original Points Count	16384	Points Count	131072
Pulse Sequence	zgpg30	Solvent	CHLOROFORM-D			Sweep Width (Hz)	24154.59
Temperature (degree C)	27.000						

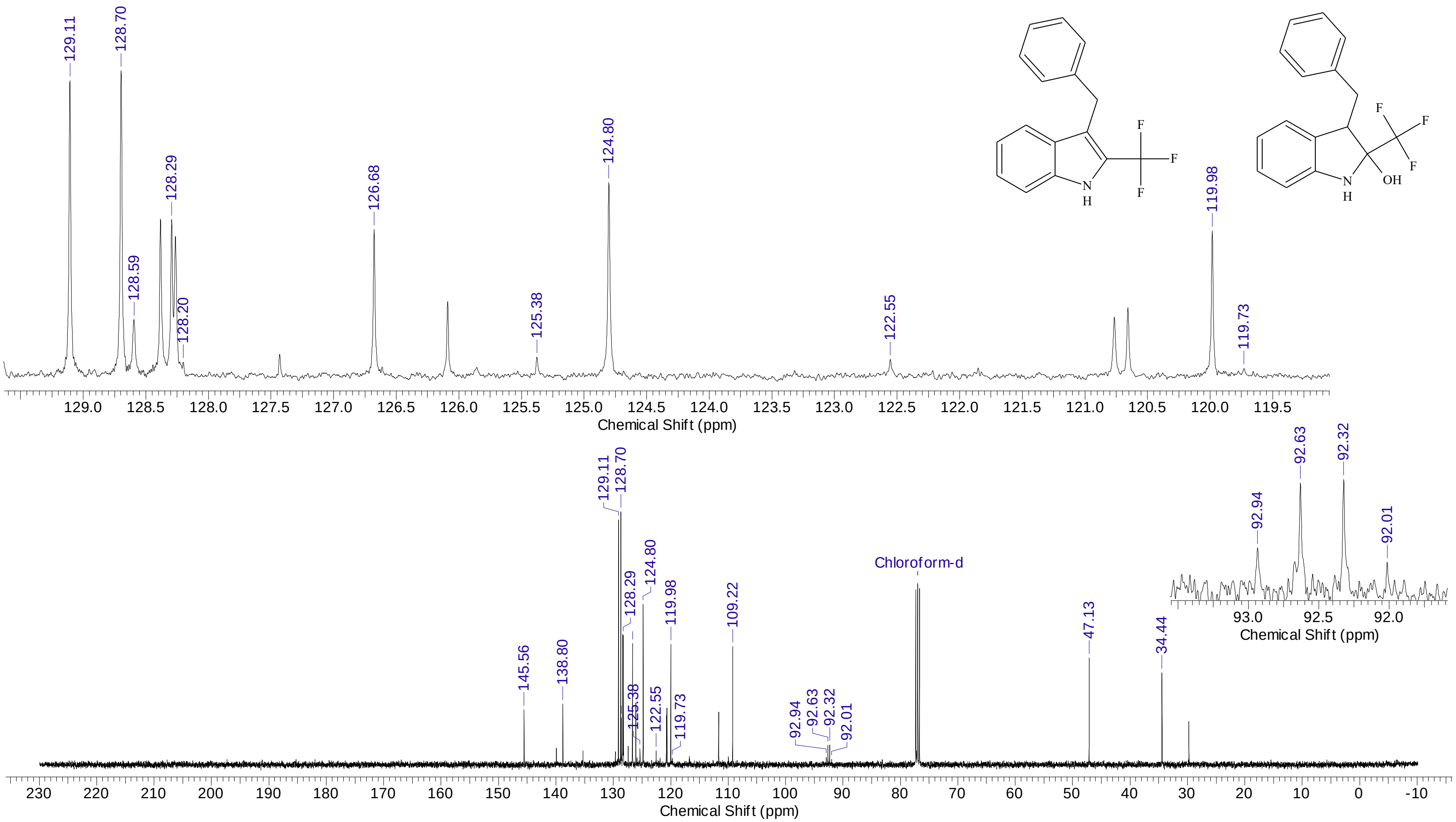

$^{13}C\{^1H\}$ NMR spectrum of **D** (100.6 MHz, $CDCl_3$)
